# Investigating the pathogenic SNPs in BLM helicase and their biological consequences by computational approach

**DOI:** 10.1038/s41598-020-69033-8

**Published:** 2020-07-23

**Authors:** Faisal A. Alzahrani, Firoz Ahmed, Monika Sharma, Mohd Rehan, Maryam Mahfuz, Mohammed N. Baeshen, Yousef Hawsawi, Ahmed Almatrafi, Suliman Abdallah Alsagaby, Mohammad Azhar Kamal, Mohiuddin Khan Warsi, Hani Choudhry, Mohammad Sarwar Jamal

**Affiliations:** 1 Department of Biochemistry, Faculty of Science, Stem Cells Unit, King Fahd Medical Research Center, King Abdulaziz University, Jeddah, 21589 Saudi Arabia; 2 Aston Medical Research Institute, Aston Medical School, Aston University, Birmingham, B4 7ET UK; 3 Department of Biochemistry, College of Science, University of Jeddah, Jeddah, 21589 Saudi Arabia; 4 University of Jeddah Centre for Scientific and Medical Research (UJ-CSMR), University of Jeddah, Jeddah, 21589 Saudi Arabia; 5 Department of Chemical Sciences, Indian Institute of Science Education and Research (IISER), Mohali, India; 6 King Fahd Medical Research Center, King Abdulaziz University, Jeddah, Saudi Arabia; 7 Department of Medical Laboratory Technology, Faculty of Applied Medical Sciences, King Abdulaziz University, Jeddah, Saudi Arabia; 8 Department of Computer Science, Jamia Millia Islamia, New Delhi, Delhi India; 9 Department of Biology, College of Science, University of Jeddah, Jeddah, 21589 Saudi Arabia; 10 Department of Genetics, Research Center, King Faisal Specialist Hospital, and Research Center, MBC-03, PO Box 3354, Riyadh, 11211 Saudi Arabia; 11 Department of Biology, Faculty of Science, University of Taibah, Medinah, Saudi Arabia; 12 Department of Medical Laboratories, Central Biosciences Research Laboratories, College of Science in Al Zulfi, Majmaah University, Al Majma’ah, Saudi Arabia; 13 Department of Biochemistry, Cancer Metabolism and Epigenetic Unit, Faculty of Science, Cancer and Mutagenesis Unit, King Fahd Center for Medical Research, King Abdulaziz University, Jeddah, Saudi Arabia; 14 Integrative Biosciences Center, Wayne State University, Detroit, MI 48202 USA

**Keywords:** Tumour biomarkers, Cancer prevention, Protein function predictions, Protein structure predictions

## Abstract

The BLM helicase protein plays a vital role in DNA replication and the maintenance of genomic integrity. Variation in the BLM helicase gene resulted in defects in the DNA repair mechanism and was reported to be associated with Bloom syndrome (BS) and cancer. Despite extensive investigation of helicase proteins in humans, no attempt has previously been made to comprehensively analyse the single nucleotide polymorphism (SNPs) of the BLM gene. In this study, a comprehensive analysis of SNPs on the BLM gene was performed to identify, characterize and validate the pathogenic SNPs using computational approaches. We obtained SNP data from the dbSNP database version 150 and mapped these data to the genomic coordinates of the “NM_000057.3” transcript expressing BLM helicase (P54132). There were 607 SNPs mapped to missense, 29 SNPs mapped to nonsense, and 19 SNPs mapped to 3′-UTR regions. Initially, we used many consensus tools of *SIFT, PROVEAN, Condel, and PolyPhen-2,* which together increased the accuracy of prediction and identified 18 highly pathogenic non-synonymous SNPs (nsSNPs) out of 607 SNPs. Subsequently, these 18 high-confidence pathogenic nsSNPs were analysed for BLM protein stability, structure–function relationships and disease associations using various bioinformatics tools. These 18 mutants of the BLM protein along with the native protein were further investigated using molecular dynamics simulations to examine the structural consequences of the mutations, which might reveal their malfunction and contribution to disease. In addition, 28 SNPs were predicted as “stop gained” nonsense SNPs and one SNP was predicted as “start lost”. Two SNPs in the 3′UTR were found to abolish miRNA binding and thus may enhance the expression of BLM. Interestingly, we found that BLM mRNA overexpression is associated with different types of cancers. Further investigation showed that the dysregulation of BLM is associated with poor overall survival (OS) for lung and gastric cancer patients and hence led to the conclusion that BLM has the potential to be used as an important prognostic marker for the detection of lung and gastric cancer.

## Introduction

The BLM gene encodes an important nuclear protein, BLM helicase, which is involved in DNA replication and the maintenance of genomic integrity. BLM is a 3′ to 5′ DNA helicase that belongs to the evolutionarily conserved RecQ helicase family. Most mammals have five RecQ helicases (RECQL1, BLM, WRN, RECQL4, and RECQL5). Helicases are crucial for unwinding duplex DNA to produce the transient single-stranded DNA (ssDNA) intermediates necessary for replication, recombination, and repair^1–3^. In a complex with topoisomerase Topo IIIa and Rmi1/Rmi2, BLM helicase repairs, double-strand DNA breaks (DSBs) through a homologous recombination (HR) pathway^4^. Consequently, cells lacking functional BLM show an about tenfold increase in chromatid breaks, mitotic recombination, and sister chromatid crossover formation^5^. Bloom syndrome (BS) is a rare autosomal recessive genetic disorder caused by pathogenic variants in the BLM gene. BS belongs to OMIM  entry 210900, which is characterized by genome instability that includes increased crossovers between homologous chromosomes^6^. The BLM gene is transcribed to a 97.93 kb-long precursor-mRNA with 21 exons, which encode a 1,417 amino acid protein. The literature shows that a large number of BS patients show insertion, deletion and missense mutations that change the amino acid sequence or nonsense mutations that introduce a premature stop codon in the BLM gene and thus inactivate the BLM helicase^7–9^. Symptoms of BS include low birth weight, dolichocephaly (long, narrow head), congenital short stature, growth retardation, sun-sensitive facial rash, an elevated risk of diabetes mellitus, reduced fertility and immune deficiency^6,10–12^. The absence of BLM protein activity causes a defect in DNA repair with a consequent increased rate of mutations and thus poses an elevated risk of cance^12–15^. The average life span of BS patients is approximately 27 years, with the most common cause of death being cancer (https://weill.cornell.edu/bsr/).

Single nucleotide polymorphisms (SNPs) are a common genetic variation contributing greatly to phenotypic variation in the general population^6^. SNPs can alter the functional consequences of proteins. In the coding region of genes, SNPs may be synonymous, non-synonymous (nsSNPs) or nonsense^16^. Synonymous SNPs change the nucleotide base residue but do not change the amino acid residue in the protein sequence due to the degeneracy of the genetic code. The nsSNPs, also called missense variants, alter amino acid residues in protein sequences and thus change the function of proteins through altering protein activity, solubility and protein structure. Nonsense SNPs introduce premature termination in the protein sequence.

The non-coding region of the gene contains several regulatory *cis*-elements, such as miRNA binding sites, that can also affect the regulation of gene expression^17–20^. SNPs have emerged as genetic markers for diseases, and there are many SNP markers available in public databases. Previous reports have shown the value of defining mutations as deleterious or non-deleterious and their connection with certain diseases, thus identifying pathogenic SNPs that are functionally compromised due to structure-damaging properties^21–27^.

In recent decades, the computational approach has become established as an effective method that streamlines time consuming, laborious experimentation and allows researchers to shortlist the most critical or pathological SNPs. This ability leads us to focus on selectively targeted SNPs instead of scanning full genes for the identification of pathological SNPs by experimental mutational analysis. Computational studies have also evaluated and analysed genetic mutations for their pathological effects and are effective in establishing the underlying molecular mechanism^26–34^.

With recent advances in high-throughput sequencing technology, hundreds of new SNPs have been mapped to human BLM genes. However, not all SNPs are functionally important. Despite extensive studies of helicase proteins in humans and the effect of their polymorphisms in cancer^11^, no attempt was previously made to comprehensively and systematically analyse and establish the functional consequences of SNPs of the BLM gene. The aim of this study was to identify the high-confidence pathogenic SNPs of the BLM gene and to determine their structural, molecular and functional consequences using computational approaches. This work may be useful in the development of precision medicine-based treatment for diseases caused by these genomic variations. In the future, this report can be used for biomarker discovery by establishing the importance of SNPs for the diagnosis of Bloom syndrome, as well as cancer, and will further aid in targeted therapies.

## Materials and methods

Application of computational methodological approaches for acquiring biological insight is well established^35–41^. Earlier studies support the notion that the application of various powerful tools and algorithms leads to increased prediction accuracy^42–45^. To ensure that the results are of the highest accuracy, we utilized several computational algorithms that can be used for the prediction of nsSNPs of BLM helicases because of their disease-related properties. For this purpose, we used tools such as SIFT^15^, PROVEAN^46^, Polyphen-2.0^47^, SNAP2-2^48^, Muscle^49^, Weblogo^50,51^, SNP&GO^52^, nsSNPAnalyzer^53^, and Mutpred-2^54^. This approach enabled greater accuracy for the prediction of most disease-associated mutations in BLM genes and their structural consequences.

### SNP dataset

**Figure 1:**
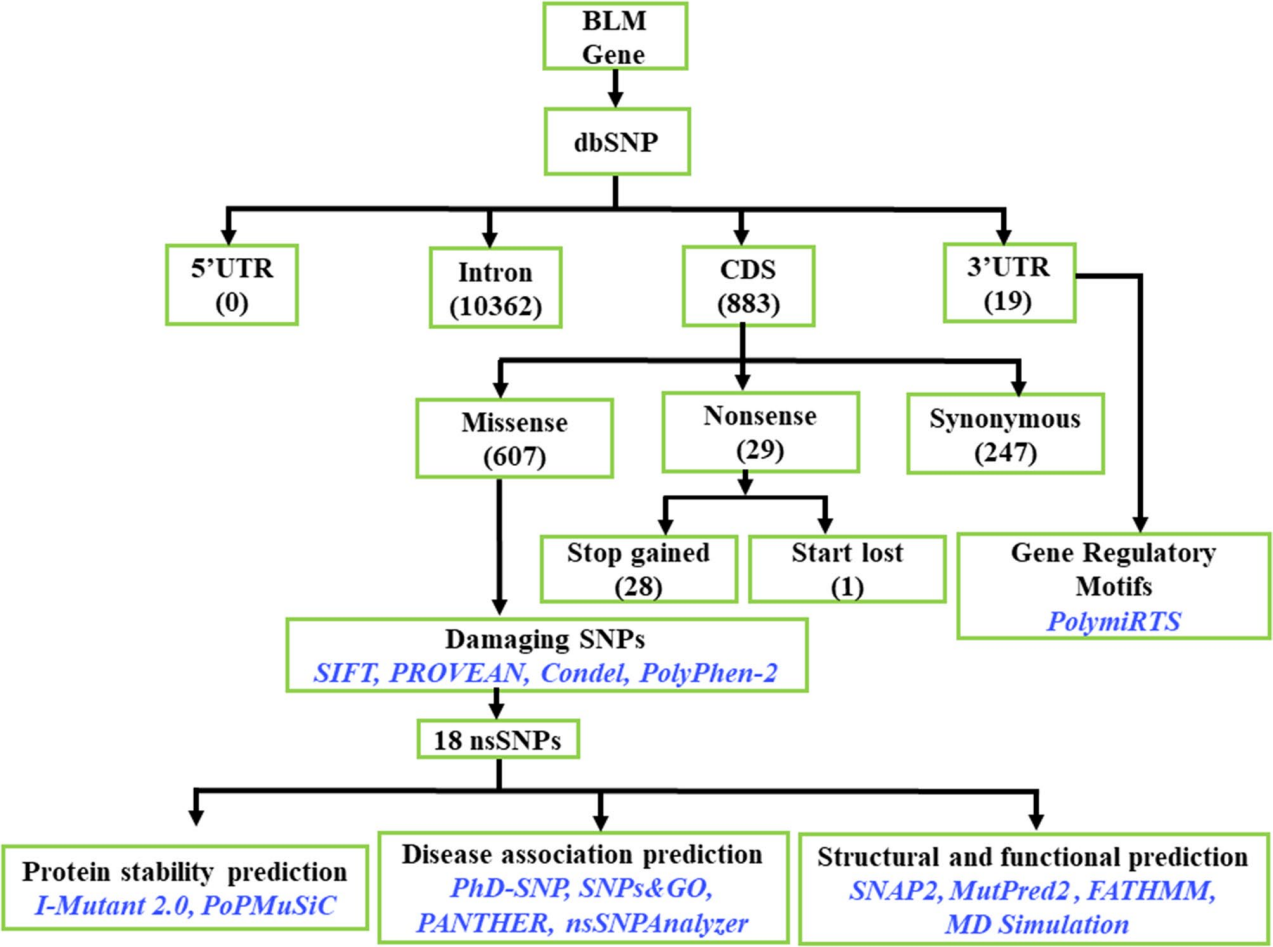
Flow chart of the in silico analysis of pathogenic SNPs in the BLM gene and their biological consequences.

The SNPs of the BLM helicases were retrieved from the dbSNP database build 150 and mapped on genome assembly GRCh38 using Variation Viewer^12^. We used “BLM” as our search term and filtered for SNPs (https://www.ncbi.nlm.nih.gov/variation/view/?q=BLM). Furthermore, we mapped these SNPs to the genomic coordinates of the “NM_000057.3” transcript expressing the BLM helicase (P54132) for computational analysis of the effect of missense variants and nonsense SNPs as well as SNPs in the UTR region. The protein sequences of the genes for BLM (P54132) were retrieved from the UniProt database (https://www.UniProt.org). We employed various sets of computational tools that together increased the accuracy and reliability of the identification of pathogenic SNPs and their effects on the structural and functional consequences of BLM (Fig. 1).

### Tools used to predict the SNP effects

#### Predicting deleterious and damaging nsSNPs

##### SIFT

An algorithm that predicts tolerant and intolerant coding base substitutions based upon amino acid properties and sequence homology^15^. The tool considered the vital positions in the protein sequence that have been conserved throughout evolution, and therefore, substitutions at conserved alignment positions are expected to be less tolerated and to affect protein function more than those at diverse positions. We used SIFT version 2.0 (https://sift.jcvi.org/), which predicted an amino acid substitution score from zero to one. SIFT predicted substituted amino acids to be damaging at a default threshold score < 0.05, while a score ≥ 0.05 was predicted to be tolerated.

##### PROVEAN

An online tool (https://provean.jcvi.org/) that uses an alignment-based scoring method for predicting the functional consequences of single and multiple amino acid substitutions and in-frame deletions and insertions^46^. The tool has a default threshold score, i.e., -2.5. If a protein variant is below the threshold, it is predicted as deleterious; above that threshold, a protein variant is considered neutral.

##### Condel

A tool (https://bbglab.irbbarcelona.org/fannsdb/) that predicts the consequences of non-synonymous SNPs as neutral or deleterious^55^. It uses a consensus deleteriousness (Condel) score calculated by integrating the normalized score of five predictive tools: SIF^15^, PolyPhen-2^47^, Logre^56^, MAPP^57^, and MutationAssessor^58^. The Condel score could vary between 0 to 1, where a higher score indicates SNPs as deleterious.

##### PolyPhen-2

A tool that predicts the structural and functional consequences of a particular amino acid substitution in a human protein^47^. The prediction of the PolyPhen-2 server (https://genetics.bwh.harvard.edu/pph2/) is based on a number of features, including structural and sequence comparison information. Its score varies between 0.0 (benign) and 10.0 (damaging). The PolyPhen-2 prediction output categorizes SNPs into three basic categories: benign (score < 0.2), possibly damaging (score between 0.2 and 0.96), and probably damaging (score > 0.96).

#### BLM helicase sequence retrieval and SNP position sequence logo

BLM helicase sequences were retrieved from the UniProt protein sequence database using a search option with the key words ‘BLM helicase’. The sequences containing the key words ‘Bloom’ and/or ‘Blm’ with the names were considered, and then the fragments and irrelevant sequences were removed. Finally, 153 sequences were obtained and considered for multiple sequence alignment. The multiple sequence alignment was performed by Muscle v.3.8.31^49^, and all the respective sites for human BLM helicase SNPs were extracted from the alignment using an in-house Perl script. The sequence logo for the SNP positions was prepared using Weblogo v.2.8.2^50,51^. A sequence logo is a graphical representation of a multiple sequence alignment, whereby a stack of amino acid symbols corresponds to a column position in the alignment. Within a stack, the varying heights of amino acid symbols show the relative frequency of each amino acid at that position.

#### Predicting disease-associated nsSNPs

##### SNPs&GO

A webserver that predicts whether an amino acid substitution is associated with a disease or not (https://snps.biofold.org/snps-and-go)^52^. It is a support vector machine (SVM)-based tool that considers protein sequence features, evolutionary information, and functional annotation according to Gene Ontology terms. We input the Swiss-Prot Code of BLM helicase (P54132) and provided the list of amino acid mutations. The results predicted whether helicase polymorphisms would be disease associated or not by three methods: (a) SNPs&GO, (b) PhD-SNP, and (c) PANTHER. A probability score > 0.5 is predicted as a disease-associated variation.

##### nsSNPAnalyzer

A random forest classifier developed using curated SNP datasets from SwisProt to predict the phenotypic effects of nsSNPs (https://snpanalyser.uthsc.edu/)^53^. It can predict (a) the structural environment of SNPs; (b) the normalized probability of substitution in the multiple sequence alignment; and (c) the similarity and dissimilarity between the variant and the original amino acid.

#### Predicting the molecular and phenotypic impact of nsSNPs

##### SNAP2

A tool used to predict the functional consequences of non-synonymous SNPs using a neural network (https://rostlab.org/services/snap/)^48^. SNAP2 incorporates various features, including evolutionary similarity from multiple sequence alignments and secondary structure and solvent accessibility, to predict whether a substitution is likely to alter the protein effect. It predicts a score from -100, considered strongly neutral, to + 100, considered as a strong effect. A threshold score > 0 is considered an effect. We input the protein sequences of BLM helicases and obtained the score of SNPs.

##### MutPred2

A neural network-based method to predict the molecular and phenotypic impact of amino acid variants as pathogenic or benign in humans (https://mutpred.mutdb.org/)^54^. It is programmed on 53,180 pathogenic and 206,946 putatively benign amino acid substitutions from HGMD, Swiss-Prot, dbSNP, and orthologous alignments. It also incorporated the impact of amino acid substitutions on over 50 different local structural and functional protein properties and thus helped to infer the molecular mechanisms of pathogenicity. The outcome of MutPred2 includes the following: (A) a general pathogenicity score (g), which is the likelihood that a substituted amino acid is pathogenic; (B) predicted molecular mechanism; (C) property score (pr) of molecular mechanism and its P-value (P); and (D) affected PROSITE and ELM Motifs. General scores vary from 0.0 (benign) to 1.0 (pathogenic), where with g ≥ 0.5 would suggest pathogenicity; however, g ≥ 0.68 yields a false positive rate (fpr) of 10%, whereas g ≥ 0.80 yields an fpr of 5%. The higher the property score (Pr), the more likely that the molecular mechanism of the disease involves the alteration of the property.

### Structural characterization of predicted nsSNPs

#### Modelling of mutant BLM helicase proteins

The coordinates of the native human BLM helicase were retrieved from the PDB database with PDB id ‘4O3M’^59,60^, referred to in the article as the wild type (WT). The WT structure consists of the BLM protein (640–1,290) bound to 1 ADP molecule, 1 Ca^2+^ ion, 1 Zn^2+^ ion, and a 3′-overhang DNA duplex. A few residues were missing in the crystal structure: 799–807, 1,011–1,013, 1,069–1,071, 1,093–1,104, 1,195–1,206, and 1,292–1,298. We modelled these residues using Modeller9v15^61^. The modelled structure was minimized and refined using simulations. Corresponding point mutations were introduced in the obtained structure using Modeller9v15^61^ to generate mutant structures. For the simulations, the protein and nucleic acids were represented by Amber forcefields: ff14SB^62^ and DNA (OL15)^63^, respectively. For bound ADP and two ions, GAFF charges provided with the AMBER forcefields were used. The modelled systems were solvated in a cubic box with TIP3P potential at a 10 Å marginal radius and neutralized with Na^+^ or Cl^−^ ions. Long-range interactions were modelled using particle mesh Ewald (PME) with a tolerance of 1e-05 and a grid spacing of 1.2 Å. A non-bonded cut-off of 12 Å was applied to the Lennard–Jones potential, and the cut-off for the direct-space part of the Coulomb forces was a switching function starting at a distance of 10 Å and reaching zero at 12 Å. A 2 fs time step was used for all simulations while constraining bonds involving hydrogens with the Lincs algorithm. Periodic boundary conditions were employed to eliminate surface effects. To reach the desired temperature and pressure, minimized systems were simulated for 5 ns while coupled to a *v-rescale* thermostat and Parinello-Rahman barostat implemented in Gromacs2016.5^64^. The equilibrated structures of the WT and 18 mutants were simulated further for 20 ns each.

#### Trajectory and structural analyses

Structural analyses were performed using built-in programs of Gromacs2016.5 and VMD-1.9.3^65^. Root mean square deviations (rmsd) and radius of gyration values for all backbone atoms were calculated with respect to the crystal structure. Root mean square fluctuations (rmsf) of Cα atoms in all trajectories were also calculated. The changes in the secondary structure of proteins during the course of simulation were analysed using the DSSP program as implemented in the Bio3D package written in R^66–68^. Domain-wise rmsd values were calculated for each domain after aligning the rest of the protein.

### Assessment of the effect of BLM genes on survival by Kaplan–Meier plots

Kaplan–Meier plots were analysed using online KM plotter software (https://kmplot.com/analysis/)^69^. The tool analyses the effect of 54,675 genes on the survival outcome of patients using 10,293 cancer samples from the Affymetrix microarray data in the Gene Expression Omnibus (GEO: https://www.ncbi.nlm.nih.gov/geo/), the European Genome-phenome Archive (EGA: https://ega.crg.eu/) and The Cancer Genome Atlas (TCGA: https://cancergenome.nih.gov/) databases. We analysed the potential effect of BLM gene expression on overall survival in a large number of cancer patients, including patients with gastric (1,065), ovarian (1816), lung (2,437), and breast (5,143) cancer. The hazard ratio (HR) with 95% confidence intervals and log rank *P*-value (below 0.05 were considered significant) were calculated. Biased arrays were excluded for quality control.

#### The GEPIA

A web-based tool for analysing the differential expression of mRNA in tumour and normal cells (https://gepia.cancer-pku.cn/index.html)^70^. The tool uses expression data from 8,587 normal and 9,736 tumour samples obtained from the TCGA and the Genotype-Tissue Expression (GTEx) projects. The mRNA expression on box plot was calculated using “Expression DIY” module using parameters: Gene “BLM”; |Log2FC| cut-off “1.5”; p-value cut-off “0.01”, and match TCGA normal and GTEx data.

#### PolymiRTS 3

A database of variants in miRNA and miRNA target sites (https://compbio.uthsc.edu/miRSNP/)^71^. It was used to examine the impact of SNPs on miRNA-target (BLM) binding and on BLM gene expression.

## Results and discussion

The application of computational biology in genome research is well established^18,72^ and is frequently used to filter for the most potential deleterious nsSNPs in target genes to understand the aetiology of various diseases^28,73–75^. Computational analysis can provide molecular insight into the changes in protein structure and function due to point mutations in the target genes. In the current study, we report nsSNPs in BLM genes, which are most likely associated with pathogenic conditions involving BLM and other BS-associated diseases, such as cancers. Our findings suggest that the application of multiple powerful algorithms to SNP datasets identifies the most deleterious SNPs, which might be associated with diseases. The accuracy of nsSNP prediction was also supported by various reports discussed below.

For this study, we retrieved 11,983 rsIDs of SNPs mapped in the human BLM gene from dbSNP (Table S1). However, these rsIDs fall in different molecular consequence classes. For instance, some rsIDs are associated with multiple SNPs and therefore belong to different classes. We mapped these SNPs to the genomic coordinates of the “NM_000057.3” transcript expressing BLM helicase (P54132). There were 883 SNPs (825 #rsID) mapped to the CDS region, 19 SNPs (17 #rsID) to the 3′UTR, and 10,362 SNPs (9,956 #rsID) mapped to introns. We did not find any SNPs in the 5′UTR of the NM_000057.3 transcript. In the CDS region, 608 SNPs (570 #rsID) were missense mutations, 28 SNPs (28 #rsID) were nonsense, and 247 SNPs (233 #rsID) belonged to the synonymous group (Table S2). For the study of the functional consequences of nsSNPs in BLM genes, we selected the “missense” and “nonsense” variant map to reference transcript “NM_000057.3”. In addition, we analysed the effects of SNPs in the 3′UTR of the “NM_000057.3” transcript on miRNA binding and poly (A) signals. The selection gave us a final dataset of 636 nsSNPs with 597 rsIDs and 19 SNPs (17 #rsID) mapped to the 3′UTR, which were then used for further analysis.

### Predicting deleterious and damaging nsSNPs

To predict the damaging nsSNPs, we employed multiple consensus tools. Initially, we used the online tool VEP (https://www.ensembl.org/Tools/VEP). VEP has advantages, such as using the latest human genome assembly, GRCh38.p10, and predicting thousands of SNPs from multiple tools, including *SIFT, PROVEAN, Condel, and PolyPhen-2,* to retrieve accurate results. We uploaded the 597 nsSNP accession numbers to the VEP tool, and the prediction results were used for further analysis.

We found 28 nsSNPs predicted as “stop gained” and one nsSNP as a “start lost” mutation (Table S3). The remaining nsSNPs were filtered on default scores of consensus tools based on sequence and structure homology methods: (a) SIFT (score < 0.5) and (b) PROVEAN (score < − 2.5) and *Condel* (score > 0.522), which showed 136 nsSNPs as damaging (Table S4). To obtain very high-confidence nsSNPs that impact the structure and function of the BLM helicase, we considered highly stringent scores across different consensus tools at parameters of *SIFT* (score = 0), *PROVEAN* (score < -8.0) and *Condel* (score > 0.9), we obtained 18 nsSNPs (Table 1). These 18 nsSNPs were further analysed by PolyPhen-2, which gave a score greater than 0.96 for all 18 nsSNPs and therefore placed them in the predicted category of probably damaging. Additional analysis of these 18 nsSNPs with SNAP2 showed that these amino acid substitutions have a damaging effect on protein structure with high accuracy (Detail information: Table S5).

**Table 1:** List of deleterious missense SNPs in the BLM gene using consensus bioinformatics tools. These SNPs are selected based upon the parameters *SIFT* (score = 0), *PROVEAN* (score < -8.0) and *Condel* (score > 0.9).

S.N	#rsID	VSc	Amino acid	SIFT	PROVEAN	Condel
1	rs761589072	C>T	Pro690Leu	0	− 9.32	0.935
2	rs770625327	C>T	Pro702Leu	0	− 9.24	0.945
3	rs148394770	T>C	Trp803Arg	0	− 13.05	0.945
4	rs761938011	G>T	Trp803Leu	0	− 11.92	0.935
5	rs766292814	A>T	His805Leu	0	− 10.26	0.935
6	rs145029382	A>G	Tyr811Cys	0	− 8.26	0.945
7	rs749632465	C>T	Pro825Leu	0	− 8.37	0.902
8	rs763471784	G>T	Gly891Val	0	− 8.8	0.906
9	rs367543034	G>T	Gly952Val	0	− 8.34	0.935
10	rs150475674	G>T	Gly972Val	0	− 8.15	0.911
11	rs1051102270	A>G	Tyr974Cys	0	− 8.35	0.945
12	rs750210123	G>T	Gly978Val	0	− 8.55	0.945
13	rs137853153	G>T	Cys1036Phe	0	− 10.13	0.945
14	rs747571272	A>G	Tyr1044Cys	0	− 8.29	0.902
15	rs746218707	T>C	Cys1055Arg	0	− 11.05	0.935
16	rs367543029	G>A	Cys1055Tyr	0	− 10.13	0.945
17	rs367543032	A>T	Asp1064Val	0	− 8.29	0.945
18	rs367543025	G>A	Cys1066Tyr	0	− 8.63	0.935

**Figure 2:**
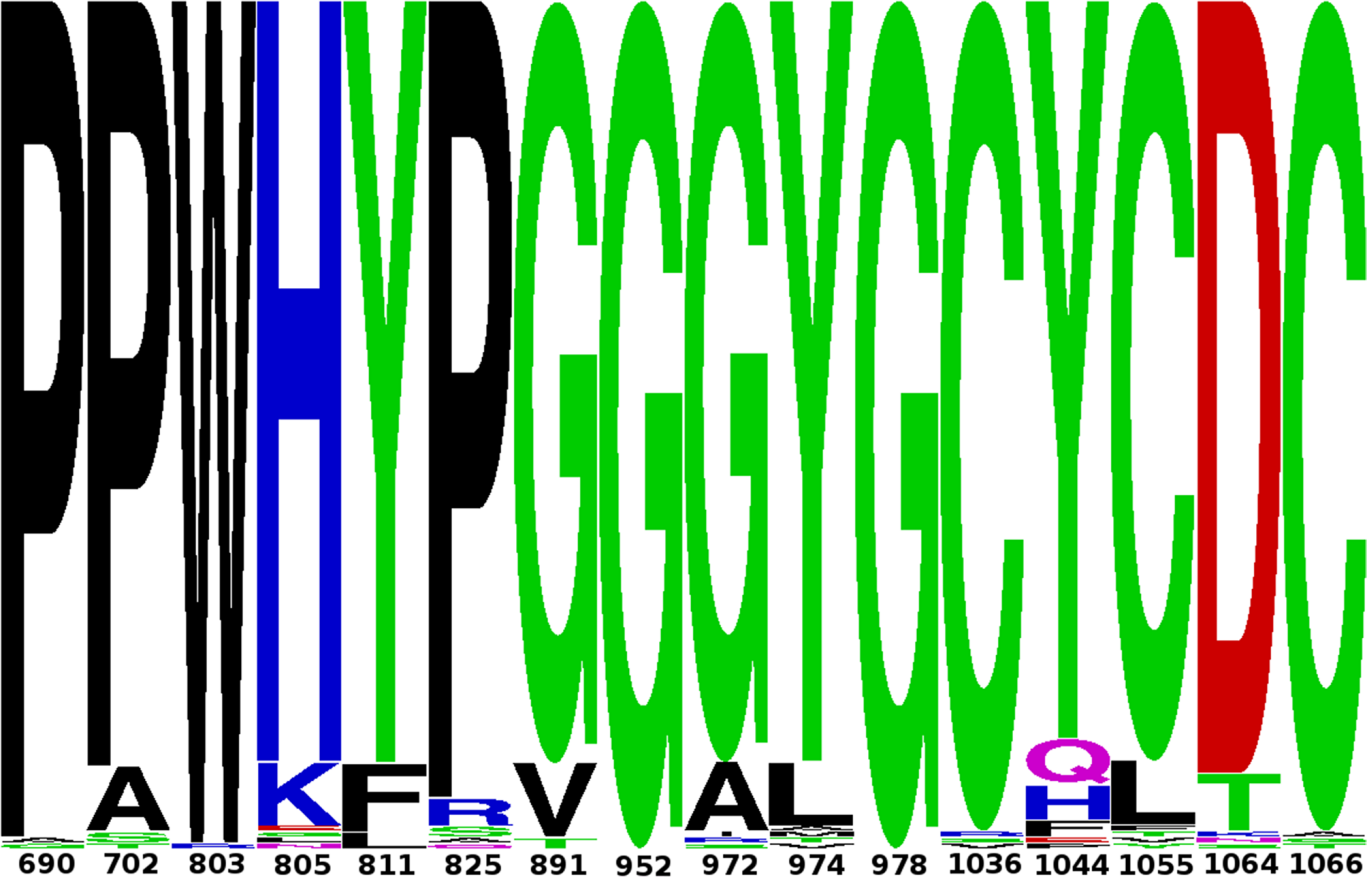
Sequence logo for 16 chosen SNP positions in BLM helicase sequence alignment. The height of the amino acid symbols shows the relative frequency of amino acids in the alignment. The amino acids are coloured according to their chemical properties. The polar amino acids (G, S, T, Y, C, Q, N) are green, basic (K, R, H) are blue, acidic (D, E) are red and hydrophobic (A, V, L, I, P, W, F, M) amino acids are black.

The sequence logo for nsSNP positions in BLM helicase sequences show that these positions are highly conserved among various species (Fig. 2). The reported 18 nsSNP variants were looked into the sequence logo, and three of them, W803R, H805L, and G891V, were found to naturally occur in other sequences; however, their frequency was very low. The two positions G952 and G978 were found to be 100% conserved, as shown in the sequence logo. The complete sequence alignment of BLM helicases is also provided in Supplementary_file1. Afterwards, these 18 high-confidence nsSNPs were further assessed for functional and structural consequences using different bioinformatics tools.

### Predicting the effect of 18 deleterious nsSNPs

#### Effect of nsSNPs on protein stability

Studies have shown that most disease-associated missense mutations change the stability of proteins^76,77^. Therefore, we analysed these nsSNPs in terms of amino acid substitutions and their effect on the stability of mutant BLM proteins by IMutant2 (https://folding.biofold.org/i-mutant/i-mutant2.0.html) and PoPMuSiC v3.1 (https://soft.dezyme.com) tools^78,79^.

Both IMutant2 and PoPMuSiC predict the effect of mutations on proteins based on protein 3D structures. Because the whole protein 3D structure of the BLM gene is not yet available, partial protein structures, such as PDB id 4O3M with a length of 640–1,290 amino acids, i.e., 613 amino acids long, were used for analysis. We submitted the 18 amino acid substitutions to IMutant2 and PoPMuSiC, which predicted the stability of the BLM protein variants. We found that three mutants (P690L, P702L, and P825L) and two mutants (P702L and G891V) were predicted to stabilize the mutant proteins by IMutant2 and PoPMuSiC, respectively (Table 2). However, twelve mutants (Y811C, G891V, G952V, G972V, Y974C, G978V, C1036F, Y1044C, C1055R, C1055Y, D1064V, and C1066Y) were predicted to destabilize the proteins by IMutant2; while thirteen mutants (P690L, Y811C, P825L, G952V, G972V, Y974C, G978V, C1036F, Y1044C, C1055R, C1055Y, D1064V, and C1066Y) were predicted to destabilize the proteins by PoPMuSiC. We found 12 mutants out of 15 predicted results showed consensus effect on the protein stability between IMutant2 and PoPMuSiC; while three mutants (W803R, W803L, H805L) were not predicted by both tools because the amino acid residues from 799 to 807 are missing in the BLM helicase crystal structure (PDB id: 4O3M) (Table 2).

**Table 2:** Effect of amino acid substitutions on the stability of mutant BLM protein using IMutant2 and PoPMuSiC v3.1 tools.

Substitutions	IMutant2		PoPMuSiC	
	DDG	Stability of variants	ΔΔG	Stability of variants
P690L	0.74	Increase	0.51	Destabilizing
**P702L**	0.70	Increase	− 1.68	Stabilizing
W803R	–	–	–	–
W803L	–	–	–	–
H805L	–	–	–	–
**Y811C**	− 0.74	Decrease	2.37	Destabilizing
P825L	0.50	Increase	1.43	Destabilizing
G891V	− 3.71	Decrease	− 1.09	Stabilizing
**G952V**	− 2.83	Decrease	2.20	Destabilizing
**G972V**	− 0.09	Decrease	0.56	Destabilizing
**Y974C**	− 1.56	Decrease	2.63	Destabilizing
**G978V**	− 1.53	Decrease	0.90	Destabilizing
**C1036F**	− 1.48	Decrease	1.32	Destabilizing
**Y1044C**	− 0.68	Decrease	1.36	Destabilizing
**C1055R**	− 2.29	Decrease	1.70	Destabilizing
**C1055Y**	− 1.49	Decrease	0.45	Destabilizing
**D1064V**	− 1.65	Decrease	0.16	Destabilizing
**C1066Y**	− 1.50	Decrease	1.09	Destabilizing

IMutant2: Gibbs free energy change value (DDG) in Kcal/mol, where DDG < 0 is decrease stability, while DDG > 0 is increase stability of mutant protein.

PoPMuSiC: The ΔΔG indicates change in folding free energy in Kcal/mol where ΔΔG < 0 is stabilizing mutation.

Consensus effect on amino acid substitutions between IMutant2 and PoPMuSiC are in bold.

The DDG/ΔΔG score for W803R, W803L, and H805L are absent because the amino acid residues from 799 to 807 are missing in the BLM helicase crystal structure (PDB id: 4O3M).

#### Identifying disease-associated nsSNPs

Eighteen selected amino acid substitutions in the BLM protein were used to analyse disease association. BLM protein ID “P54132” and its amino acid mutations were submitted to the “SNPs&GO” tool (https://snps.biofold.org/snps-and-go/snps-and-go.html). The predicted disease associations from three different tools were analysed. The output of (a) SNPs&GO and (b) PhD-SNP predicted that all the tested SNPs were associated with diseases, while (c) PANTHER predicted 16 SNPs as disease associated and two as neutral (Table S6). In addition, these nsSNPs were also analysed with the nsSNPAnalyzer tool to predict disease association. This tool also provides supplementary information about nsSNPs, such as secondary structure, structural environment, area buried and fraction polar. We uploaded the BLM protein sequence, list of 18 amino acid substitutions, and PDB structure (4O3M) to the nsSNPAnalyzer. The output of nsSNPAnalyzer reported 17 nsSNPs as disease associated and one nsSNP reported as neutral (Table 3).

**Table 3:** Predicting disease-associated amino acid substitution and phenotypic effect using nsSNPAnalyzer.

SNP	Phenotype	Environment	Area buried	Frac polar	Secondstr
P690L	Disease	P1C	0.472	0.458	C
P702L	Disease	B2H	0.526	0.417	H
W803R	Disease	–	–	–	–
W803L	Disease	–	–	–	–
H805L	Disease	–	–	–	–
Y811C	Disease	B2H	0.68	0.417	H
P825L	Neutral	P2C	0.432	0.708	C
G891V	Disease	ES	0.171	0.292	S
G952V	Disease	EC	0.165	0.333	C
G972V	Disease	EH	0.105	0.844	H
Y974C	Disease	B3H	0.713	0.448	H
G978V	Disease	EH	0.138	0.687	H
C1036F	Disease	P1C	0.221	0.24	C
Y1044C	Disease	B3H	0.615	0.615	H
C1055R	Disease	P1H	0.239	0.354	H
C1055Y	Disease	P1H	0.239	0.354	H
D1064V	Disease	P2H	0.385	0.594	H
C1066Y	Disease	P1H	0.227	0.458	H

Phenotype: Phenotype annotated by the Swiss-Prot DB. "Disease" or "Neutral".

Environment: The structural environment of the SNP B1, B2, B3, P1, P2 and E*

AreaBuried: Solvent accessibility score.

FracPolar: Environmental polarity score.

Secondstr: Secondary structure. H: alpha-helix, S: beta-sheet, C: coil.

*The first character denotes the solvent accessibility B: buried, P: partially buried, E: exposed. The second number (if exists) denotes different environmental polarity provided the solvent accessibility is the same, with a larger number corresponding to a larger polarity.

The structural features of last four columns for W803R, W803L, and H805L are absent because the amino acid residues from 799 to 807 are missing in the BLM helicase crystal structure (PDB id: 4O3M).

Eighteen amino acid substitutions were further analysed with the Fathmm server (https://fathmm.biocompute.org.uk/) at the default threshold for cancer-promoting mutations and disease-causing mutations^80^. The output results of these tools showed that two (G952V and G978V) were associated with cancer (Table 4), while three (G952V, P702L and G978V) were also associated with cancer (Table 5).

**Table 4:** Predicting cancer and disease-associated amino acid substitution and phenotypic effect using Fathmm cancer.

Substitution	Prediction	Score	HMM ID	HMM description	HMM Pos	HMM prob. W	HMM prob. M	HMM weights D	HMM weights O
P690L	PASSENGER/OTHER	1.79	41,442	P-loop containing nucleoside triphosphate hydrolases	118	0.148	0.011	1	4
P702L	PASSENGER/OTHER	1.49	41,442	P-loop containing nucleoside triphosphate hydrolases	130	0.346	0.070	1	4
W803R	PASSENGER/OTHER	1.92	41,442	P-loop containing nucleoside triphosphate hydrolases	231	0.088	0.033	1	4
W803L	PASSENGER/OTHER	2.01	41,442	P-loop containing nucleoside triphosphate hydrolases	231	0.088	0.092	1	4
H805L	PASSENGER/OTHER	1.99	41,442	P-loop containing nucleoside triphosphate hydrolases	233	0.092	0.086	1	4
Y811C	PASSENGER/OTHER	2.45	DEAD	DEAD/DEAH box helicase	140	0.050	0.026	5	28
P825L	PASSENGER/OTHER	2.41	DEAD	DEAD/DEAH box helicase	154	0.076	0.029	5	28
G891V	PASSENGER/OTHER	2.84	36,155	P-loop containing nucleoside triphosphate hydrolases	11	0.017	0.173	1	6
G952V	CANCER	− 1.49	Helicase_C	Helicase conserved C-terminal domain	46	0.798	0.006	8	14
G972V	PASSENGER/OTHER	3.06	36,155	P-loop containing nucleoside triphosphate hydrolases	83	0.002	0.282	1	6
Y974C	PASSENGER/OTHER	2.68	36,155	P-loop containing nucleoside triphosphate hydrolases	85	0.001	0.067	1	6
G978V	CANCER	− 1.12	41,442	P-loop containing nucleoside triphosphate hydrolases	263	0.885	0.001	1	4
C1036F	PASSENGER/OTHER	2.62	36,155	P-loop containing nucleoside triphosphate hydrolases	167	0.017	0.039	1	6
Y1044C	PASSENGER/OTHER	2.55	36,155	P-loop containing nucleoside triphosphate hydrolases	175	0.033	0.011	1	6
C1055R	PASSENGER/OTHER	2.61	36,155	P-loop containing nucleoside triphosphate hydrolases	207	0.012	0.027	1	6
C1055Y	PASSENGER/OTHER	2.61	36,155	P-loop containing nucleoside triphosphate hydrolases	207	0.012	0.029	1	6
D1064V	PASSENGER/OTHER	2.47	36,155	P-loop containing nucleoside triphosphate hydrolases	215	0.097	0.022	1	6
C1066Y	PASSENGER/OTHER	2.62	36,155	P-loop containing nucleoside triphosphate hydrolases	217	0.008	0.029	1	6

**Table 5:** Predicting cancer and disease-associated amino acid substitution and phenotypic effect using Fathmm Disease Ontology.

Substitution	Prediction	Score	HMM ID	HMM Description	HMM pos	HMM prob. W	HMM prob. M	HMM weights D	HMM weights O
C1036F	TOLERATED	− 0.97	36,155	P-loop containing nucleoside triphosphate hydrolases	167	0.017	0.039	12	6
C1055R	TOLERATED	− 0.98	36,155	P-loop containing nucleoside triphosphate hydrolases	207	0.012	0.027	12	6
C1055Y	TOLERATED	− 0.98	36,155	P-loop containing nucleoside triphosphate hydrolases	207	0.012	0.029	12	6
C1066Y	TOLERATED	− 0.97	36,155	P-loop containing nucleoside triphosphate hydrolases	217	0.008	0.029	12	6
D1064V	TOLERATED	− 1.11	36,155	P-loop containing nucleoside triphosphate hydrolases	215	0.097	0.022	12	6
G891V	TOLERATED	− 0.75	36,155	P-loop containing nucleoside triphosphate hydrolases	11	0.017	0.173	12	6
G952V	DAMAGING	− 3.19	Helicase_C	Helicase conserved C-terminal domain	46	0.798	0.006	26	14
G972V	TOLERATED	− 0.52	36,155	P-loop containing nucleoside triphosphate hydrolases	83	0.002	0.282	12	6
G978V	DAMAGING	− 4.12	41,442	P-loop containing nucleoside triphosphate hydrolases	263	0.885	0.001	8	4
H805L	TOLERATED	− 1.01	41,442	P-loop containing nucleoside triphosphate hydrolases	233	0.092	0.086	8	4
P690L	TOLERATED	− 1.21	41,442	P-loop containing nucleoside triphosphate hydrolases	118	0.148	0.011	8	4
P702L	DAMAGING	− 1.51	41,442	P-loop containing nucleoside triphosphate hydrolases	130	0.346	0.070	8	4
P825L	TOLERATED	2.41	DEAD	DEAD/DEAH box helicase	154	0.076	0.029	5	28
W803L	TOLERATED	− 0.99	41,442	P-loop containing nucleoside triphosphate hydrolases	231	0.088	0.092	8	4
W803R	TOLERATED	− 1.08	41,442	P-loop containing nucleoside triphosphate hydrolases	231	0.088	0.033	8	4
Y1044C	TOLERATED	− 1.03	36,155	P-loop containing nucleoside triphosphate hydrolases	175	0.033	0.011	12	6
Y811C	TOLERATED	2.45	DEAD	DEAD/DEAH box helicase	140	0.050	0.026	5	28
Y974C	TOLERATED	− 0.9	36,155	P-loop containing nucleoside triphosphate hydrolases	85	0.001	0.067	12	6

#### Predicting protein structural and functional consequences

MutPred2 was used to infer the structural, molecular and phenotypic impacts of amino acid variants. The results for MutPred2 are shown in Table S7. There are 18 nsSNPs reported with high confidence to affect the structure and function of BLM proteins.

#### Substitution effect on helicase structure of BLM via molecular dynamics simulations

Bloom syndrome proteins comprise three domains: ATPase domain (642–1,068), RecQ family-specific C-terminal (RQC) domain (1,074–1,194), and Helicase and RNase D C-terminal (HRDC) domain (1,208–1,290). The C-terminus of the ATPase domain, known as the Zn subdomain, consists of Zn-binding residues (994–1,068). One ADP molecule is observed to be bound to the inter-subdomain cleft between two ATPase subdomains, 1A (642–857) and 2A (858–1,068). In the BLM WT crystal structure, the duplex region of DNA is bound to the RQC domain surface via an unconventional winged-helix domain. This interaction differs from that known in conventional winged-helix domains, where the minor and major grooves of dsDNA are recognized by one recognition helix and β wing^81^. In the BLM RQC domain, dsDNA does not form direct interactions with the recognition helix. Instead, a loop connecting two helices serves as the prominent DNA-interacting entity of the RQC domain. The terminal region of the dsDNA duplex interacts with the β wing of the RQC domain, which seems to act as a scalpel for splitting the dsDNA duplex. The third domain of BLM, the HRDC domain, folds as a helical bundle of five α-helices and one 3_10_ helix connected by a short loop. However, much remains unknown regarding the functional aspects of the HRDC domain and how it occurs in two of the five human RecQ-family helicases (BLM and WRN), as well as in RecQ helicases in bacteria and yeast. BLM mutants lacking the HRDC domain possess core helicase and ATPase activities similar to those of the wild-type protein^82–84^ but are defective in both strand annealing^74^ and double Holliday junction dissolution^83^.

**Figure 3:**
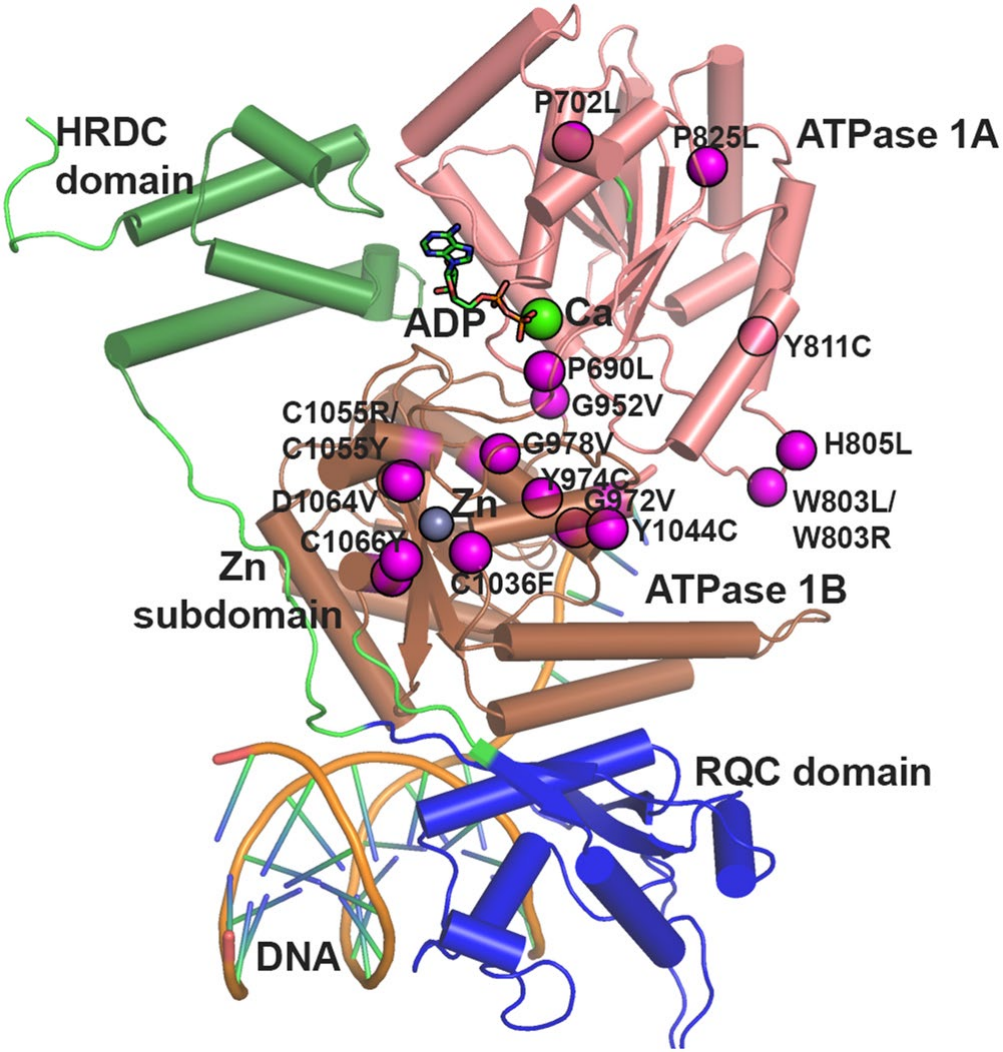
Various domains were observed in the BLM helicase crystal structure. (PDB id: 4O3M). The eighteen SNPs discussed in this study are shown by magenta spheres for Cα atoms. The bound ADP (in stick representation), Zn ion (grey sphere), Ca ion (green sphere) and DNA duplex with 3′ overhang are also shown.

**Figure 4:**
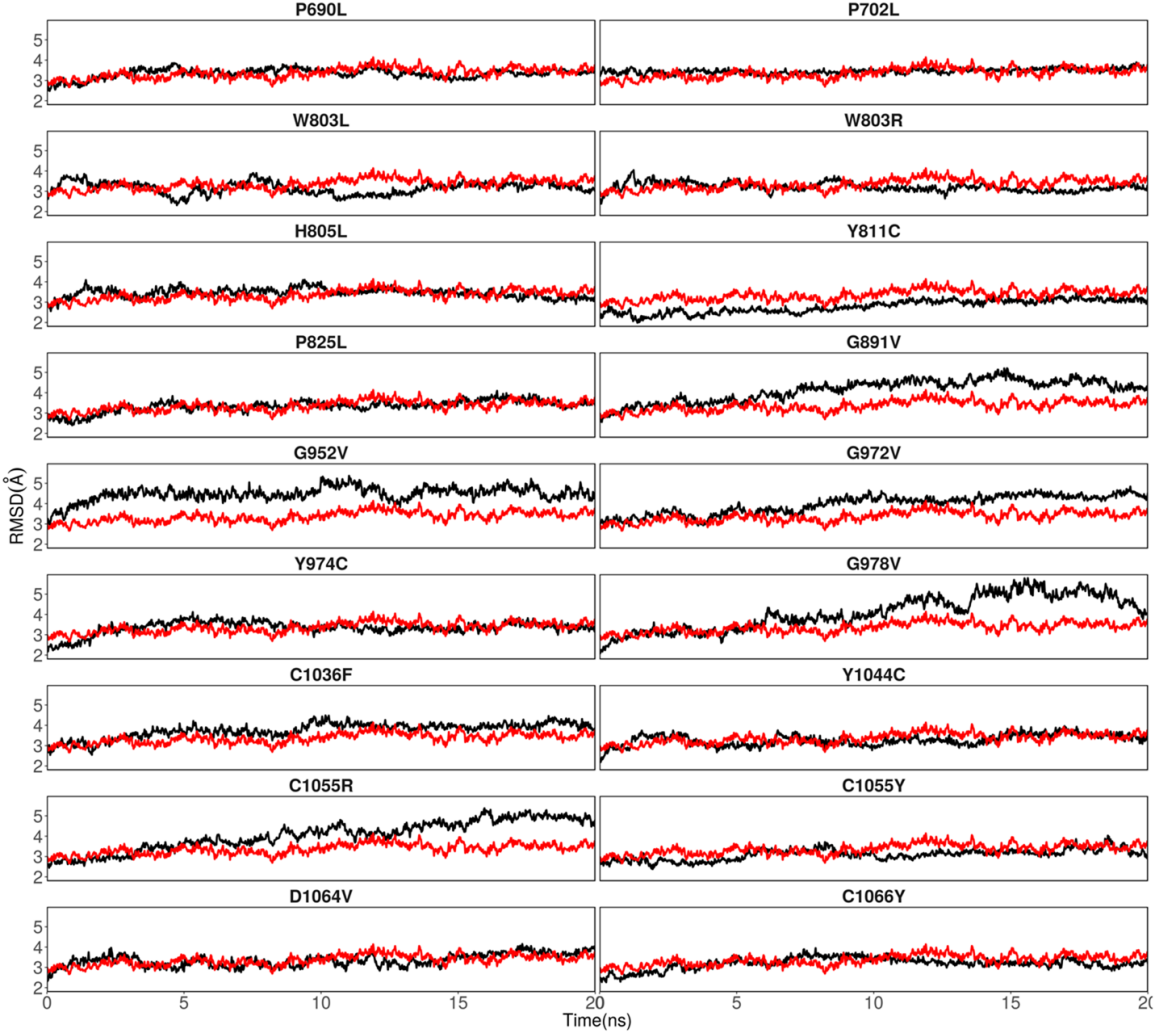
Variation in rmsd values with respect to time during simulations. Black lines correspond to mutant structures, and red lines correspond to WT or native structures.

**Figure 5:**
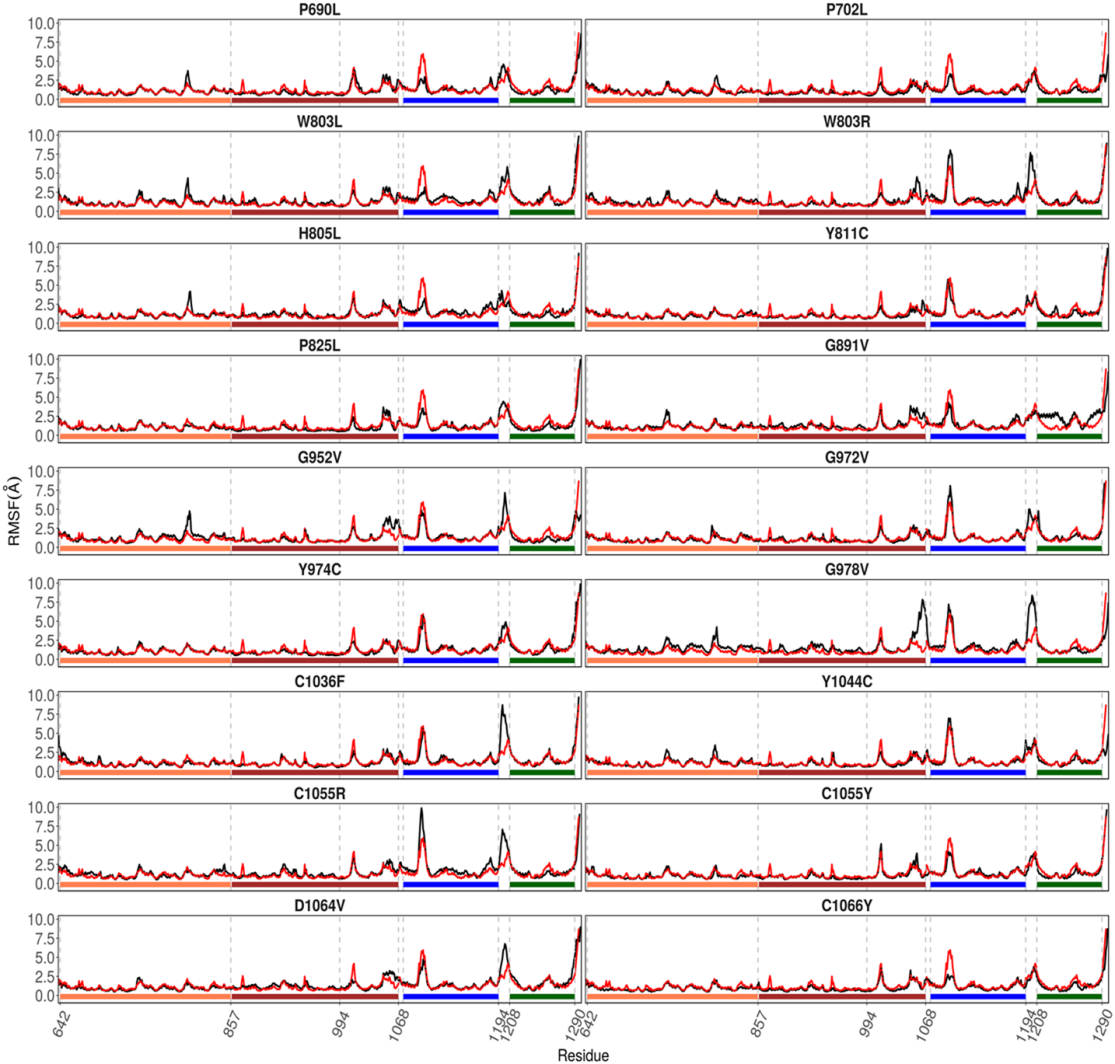
RMSF values calculated for each Cα atom with respect to residue. Black lines correspond to mutant and red lines correspond to WT or native structures. The bars below depict the domains, with coral for ATPase 1A, brown for ATPase 1B, blue for RQC and green for the HRDC domain.

**Figure 6:**
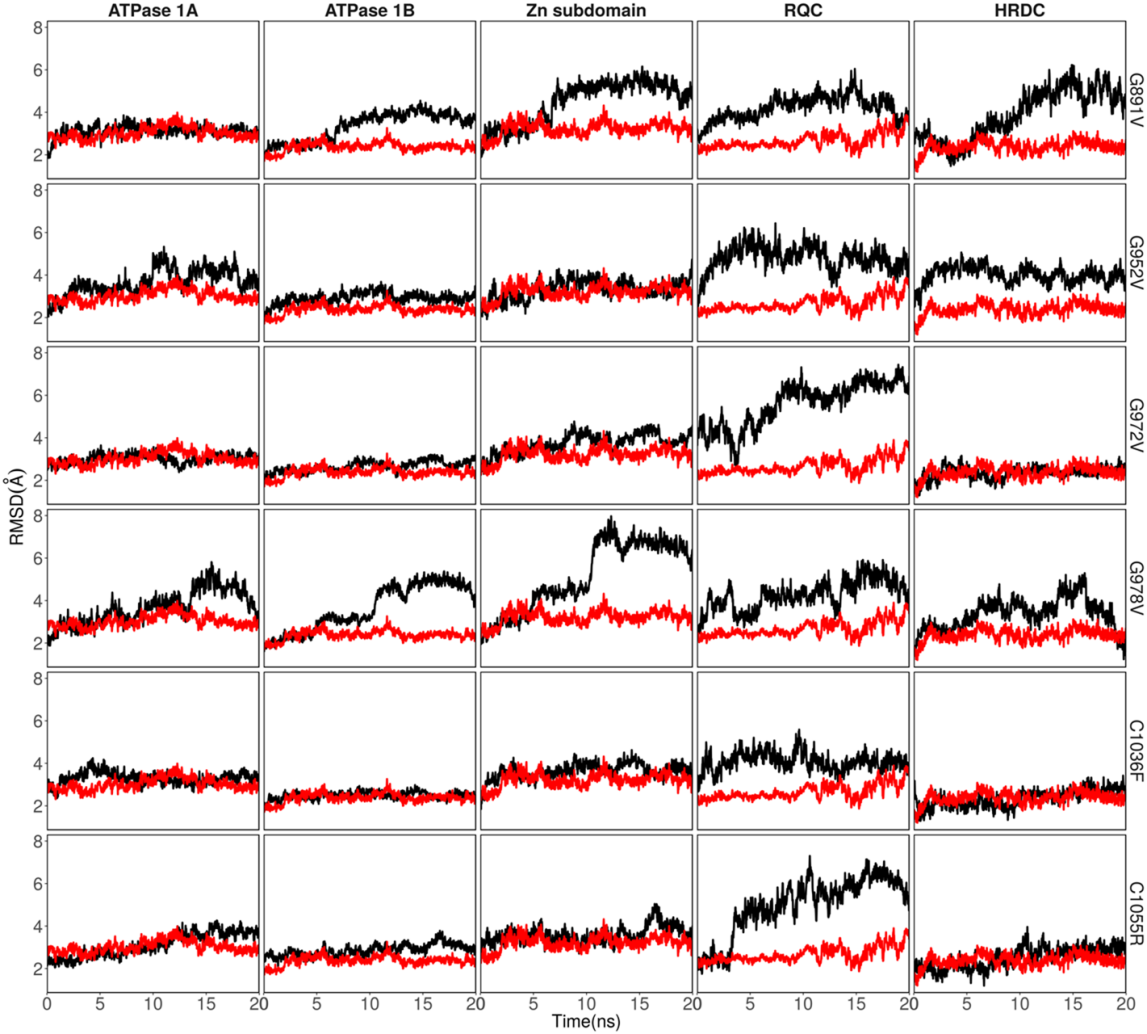
Domain wise rmsd plots for mutants destabilizing the complete BLM structure. Black line corresponds for mutants and red line corresponds for WT/native structure.

We evaluated the 18 predicted deleterious amino acid substitutions for possible damage to the 3D structure of the BLM helicase using molecular dynamics simulations. When these SNP positions were mapped onto the structure of the BLM helicase (Fig. 3), we found that the SNP positions were distributed in the helicases and coil structures. Three SNP positions, C1036, C1055, and C1066 (interestingly all cysteines), were in the Zn subdomain, directly interacting with the Zn atom. The SNP position, D1064, was also found close to the Zn atom. We then compared the deviations by rmsd (Fig. 4) and rmsf values (Fig. 5) observed during simulations within mutant proteins with those observed for native (or WT) proteins and classified the mutants into two categories: (a) mutants with considerably higher rmsd values, suggestive of instability induced in the protein; and (b) mutants with similar rmsd values, and thus, possessing significantly more or equally stable conformations compared to those of the WT. Mutants G891V, G952V, G972V, G978V, C1036F, and C1055R destabilize the BLM structure and belong to category (a), Mutants P690L, P702L, W803R, W803L, H805L, Y811C, P825L, Y974C, Y1044C, C1055Y, D1064V, and C1066Y belong to category (b). Interestingly, the mutation of C1055 seems to be residue specific. If C1055 is mutated to arginine, it destabilizes the complete BLM structure, whereas mutation to tyrosine is tolerated. The mutant C1055Y shows similar deviations from the WT. Below, we will describe the effects of each mutant in detail. For visual inspection, we compared the final structure obtained after 20 ns of sampling of each mutant with the final structure obtained for the WT or native state (Figs. S1, S2). We calculated rmsd values for each domain for these mutants (Figs. 6, S3). We further analysed the variation in secondary structure for mutants and WT with respect to time (Fig. S4).

### Mutations in ATPase domain 1A

Mutations P690L and P702L are present in ATPase domain 1A, near the ADP binding pocket. Our computational results suggest that their properties are similar to those of the WT. However, Mirzaei et al., reported six mutant residues (P690L, R717T, W803R, Y811C, F857L, G972V) that cause loss of function for the BLM protein^85^. Modelling studies of the BLM residues confirm that P690 along with R717, W803, and Y811 are located in the first lobes of the helicase domain. Among the point mutations of BLM, P690L does not affect the extremely conserved GK(T/S) residues of motif I (Walker A). Another report from Shashtri et al. showed, using a HU-based hypersensitivity-based study, that the P690L mutant of the BLM protein was unable to rescue the DNA damage response in the BS cell line compared to the WT^5^. The reason behind the loss of function could be due to leucine (hydrophobic) replacing proline (helix breaker), which may culminate in reduced flexibility and hydrophobicity, causing weakened dsDNA binding and altering the location of the dsDNA within the motif, which leads to failure to bind ATP and/or Mg^2+^^5,85^.

Studies from Mirzaei et al. showed that the W803 mutation leads to impaired BLM function associated with Bloom syndrome. This mutation site is located in the highly conserved region called the aromatic-rich (AR) loop and is crucial for ATP binding, hydrolysis, DNA binding, ssDNA binding and unwinding^86–89^. Because it is situated as a key residue in a loop region, it plays a crucial role in various functions. Another set of mutations, W803L, W803R, H805L, Y811C, P825L in ATPase domain 1A show similar dynamics as WT. W803, a residue that is conserved in all five human RecQ homologs (BLM, WRN, RecQL1, RecQL4, and RecQL5), suggests that this residue plays a key role in helicase activity. Hence, the W803R mutation might hamper helicase activity, which is one of the major causes of BS development. Though our molecular dynamics simulation-based study suggests similar dynamics to those of the WT, other studies suggest that these residues are crucial for the above-mentioned activities. Hence, we also speculate that this mutation may lead to a deterioration in BLM activity, which may lead to the development of BS. The downstream Y811C and P825L mutations showed no structural differences as WT. Though there is no evidence for P825 or Y811, it is identified as a key residue for the functioning of BLM proteins^85,91,93,96–98^. Another report^5^ suggested based on HU hypersensitivity that, similar to P602L, the Y811C mutant of the BLM protein was unable to rescue the DNA damage response in the BS cell line compared to the WT.

### Mutations in ATPase domain 1B

Mutations in ATPase domain 1B, destabilizing the BLM structure, are observed for glycines (G891V, G952V, G972V, G978V). The structural influence of these mutants can be observed by larger variations in domain-wise rmsd plots (Fig. 6). A report from Rong et al.^90^, also endorsed our results, which suggests that the G891 mutation along with Q672R, I841T and C901Y inactivates the helicase domain^7,8^. There are also point mutations, such as K803A, Q680P and I849T, in mouse BLM^7,8,90,91^. All these point mutations hamper ATPase and DNA unwinding activity. G972V mutation, in the N terminal region of BLM, may hamper the replicative role of BLM as reported by Selak et al.^92^, a major issue in Bloom Syndrome^93–97^. 972 is proximal to the nucleic acid overhang. Mutating G972 to valine pushes the nearby K968, which is directly interacting with the nucleic acid overhang, and this further affected the downstream RCQ domain and to some extent to the Zn subdomain, as observed from the domain-wise rmsd values. To depict that the nsSNP leads to full inactivation of the BLM protein, Mirzaei et al.^85^, exploited a yeast model system by designing a chimera BLM gene consisting of the yeast and human *BLM* genes. The introduction of nsSNP into the chimera BLM leads to impaired BLM function, which was examined and confirmed by a hypersensitivity assay of cells to hydroxyurea (HU), a DNA-damaging agent^85^. The hypersensitivity assay showed that six-point mutations (P690L, R717T, W803R, Y811C, F857L, G972V) cause total loss-of-function of BLM, and three cause partial loss-of-function (R791C, P868L, G1120R). The location of G972 is in close proximity to the arginine finger; therefore, this mutation might impair coordination among the ATP binding of lobe 1 with lobe 2. G978V mutation resulted in a change in the backbone orientation of the ATPase domain 1B, induced structural deviations in this interface area, as well as in the Zn subdomain, and influenced the downstream HRDC domain (Fig. 6). Since there is no report available for this mutation, in future studies, it may be informative to sequence this pathogenic mutation site, especially in BS patients and in other related diseases, and/or study it using mutational approaches using various models. We speculate that this mutation may also be a hypomorphic mutation in which, instead of causing BS, it may be connected with other BS-associated diseases, such as cancer or type 2 diabetes^5^.

Another set of mutations, Y974C and Y1044C in ATPase 1B domain, showed no significant destabilizing structural effects during the sub-nano seconds simulations. Y974 is present at the interface, pointing towards the cleft between ATPase domains 1A and 1B and forming stacking interactions with F1045 of the nearby helix. In the native structure, Y1044 forms hydrogen-bonding interactions with E971 of the nearby helix, and this interaction is maintained throughout the simulations of the WT. Mutating tyrosine to cysteine disrupts these hydrogen-bonding interactions but results in another set of stable interactions with S1025 of a nearby helix, thus maintaining the structural dynamics of the BLM. Although our computational results suggest that Y1044C is similar to the WT, other studies suggest that Y1044C is present along with S897C in Japanese male patients and white female patients with metachronous colon cancer in the C-terminal of BLM in CRC. This issue can be further explored in the future using BS cells and model organisms. Highly conserved BLM variants were reported to be found in the C-terminal helicase domain, which may lead to a predisposition to hereditary CRC^98^.

### Mutations in Zn subdomain

**Figure 7:**
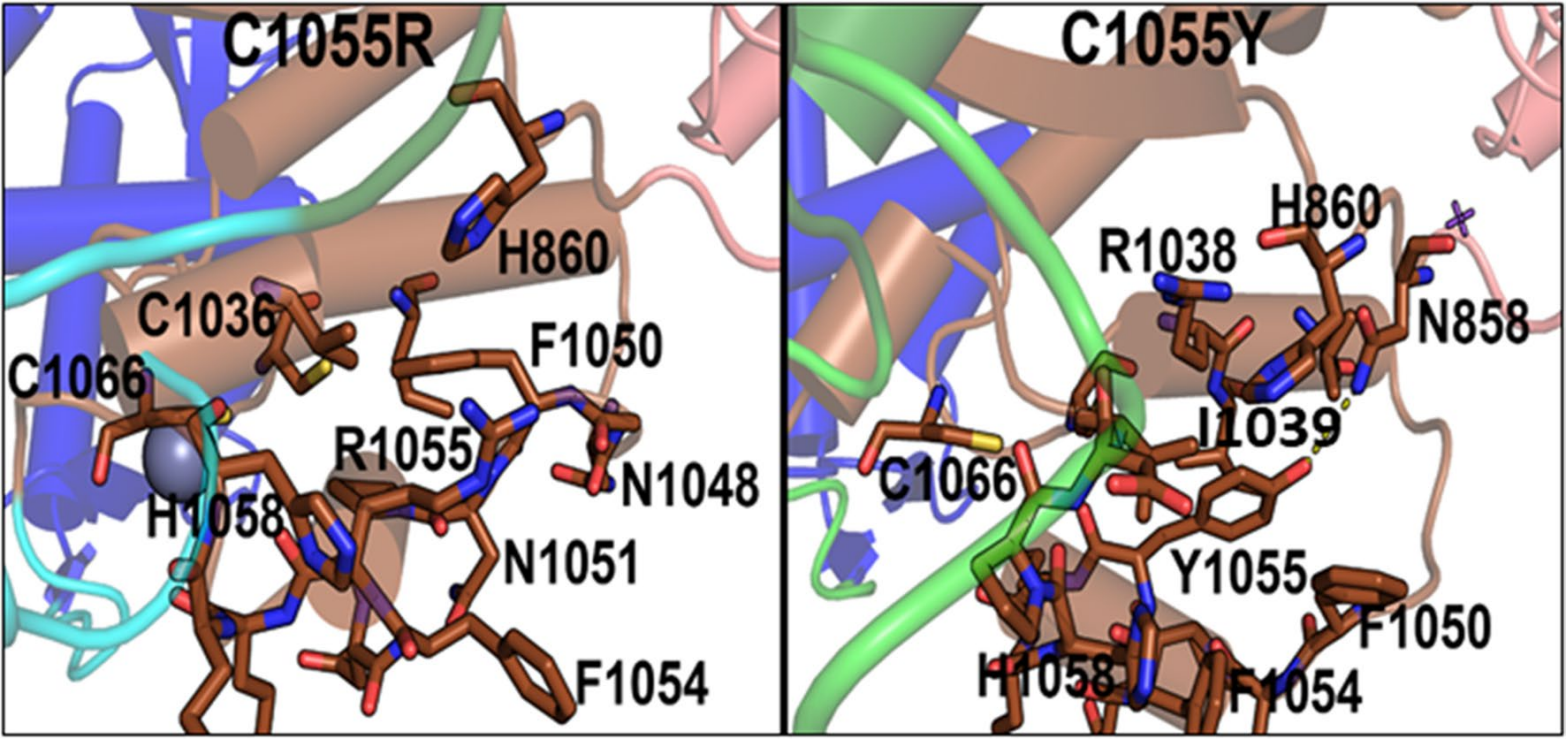
Interactions observed in the final conformations of mutants C1055R and C1055Y.

In the Zn subdomain, highly conserved residues C1036, C1055, C1063, and C1066 form interactions with the Zn atom in the BLM protein. C1036F mutation influenced the RQC domain. In the native structure, C1055 interacts with the Zn atom, along with C1063. For the C1055R mutation, when the conformations of mutants after simulation are visualized with respect to the native structure, arginine being positively charged shows repulsion with positively charged Zn and destabilization of the domain (Figs. 5, 6). This destabilization disrupts the C-terminus of ATPase domain 1A (Figs. 4, 5, 6). The destabilizing effect is indicated by the large variations observed in the rmsd and rmsf plots. We further calculated the domain-wise rmsd values for C1055R and observed that the mutation resulted in larger deviations within the Zn subdomain and the following RQC domain. However, the tyrosine mutant of C1055 is oriented towards the solvent and forms favourable hydrogen bonds with N858 and stacking interactions with I1039 and F1050, thus leading to less structural disruption than the arginine mutation, which completely disrupts the helical structure (Fig. 7). Therefore, we observe higher rmsd variations for C1055R than for C1055Y. Nonetheless, the loss of Zn ion binding is observed for both mutations. C1055 may form disulfide bridges with one of the cysteines present in close proximity to stabilize the helix structure of the BLM protein. This model predicts that the residues in the helix are important for ssDNA binding, contributing to the formation of the BLM–DNA complex. Consistent with this study, Guo et al. used a gel-shift assay to show that similar mutant C901Y leads to a significant reduction in dsDNA binding and an even greater loss of ssDNA binding^99^. This lower binding of BLM with counterparts of the DNA region may lead to disruption in the functional outcome. This may result in a predisposition to many unwanted mutations and thus to Bloom syndrome. A comprehensive site-directed mutational analysis by Guo et al.^100^, suggests that there is a significant reduction in zinc binding ability, which further indicates that these highly conserved cysteine residues are essential for Zn^2+^ ion binding and thus for BLM activity. The effect of cysteine residue mutation on DNA binding ability was shown by Guo et al.^100^, using a gel shift assay, which indicates that there was a significant reduction in DNA binding. This result suggests that conserved cysteine residues are key to the function of zinc binding domains, which are essential for the DNA helicase and ATPase activity of BLM. Therefore, the study suggests that DNA-binding ability is compromised due to this mutation^100^. Another study also showed, using the chimera mutational approach, that mutating C1055 leads to hypersensitivity to hydroxyurea (HU)^85,100^.

D1046 is present within the Zn subdomain and forms hydrogen-bonding interactions with the nearby R1037. This interaction is maintained intermittently during the simulations of the native structure. Though hydrophilic and negatively charged residues have been mutated to neutral and hydrophobic valines, the mutation occurred at the periphery of the protein, thus inducing local perturbations in the structure, and did not significantly affect the overall structural dynamics of the helicase except for the RQC domain (Fig. S3). The loss of the Zn-binding ability of such a mutant is observed as the Zn ion diffuses out of its pocket. Although our computational results suggest that its properties are similar to those of the WT, other studies suggest its crucial role in BS development^85^. C1066 is one of the four cysteines binding the Zn atom in the Zn subdomain. Our in silico results suggest that there is no effect because of the C1066Y mutation, but another report using site-directed mutational analysis suggests (Guo et al.) that there is a significant reduction in the zinc-binding ability, which further indicates that these highly conserved cysteine residues are essential for Zn^+2^ ion binding for subsequent BLM activity^100^. A report from Shashtri et al. noted that C1066Y is crucial for Zn coordination, which is required for helicase activity and consequently for the development of BS^5^.

For these 18 nsSNP mutations, we observed that few mutations disrupt the overall BLM structure within a sub-nanosecond timescale and thus disrupt the functioning of BLM helicases. These mutations are specifically present within the cleft of two ATPase subdomains, 1A and 1B, and thus hamper the binding of ADP to this site. These mutations were also supported by other experimental studies mentioned in earlier sections^5,85,100^. For some mutations, however, local disruptions are observed in subdomains but can result in a complete loss of function. For instance, the mutation of cysteines (C1036F, C1055Y, C1066Y) and glutamate (D1064V) in the Zn binding subdomain, which results in the loss of Zn binding upon mutation and thus hinders the functioning of BLM helicase, was also supported by other reports^100^. However, in all the mutations, we did not find considerable changes in the binding of DNA. This result may be attributed to the fact that the mutations are performed in the ATPase domain, and the simulation timescales in this study are not long enough to observe large allosteric effects. For the mutations G891V, G952V and G978V, however, we did find larger deviations in RCQ domain binding and in the C-terminal HRDC domain, as indicated by rmsd values, thus suggesting the possibility of allosteric effects of mutations in the ATPase domain in nucleic acid binding. This conclusion was further strengthened by reports suggesting a role for cysteine residues in the Zn-binding domain in helicase activity. The mutation of cysteine hampers BLM helicase activity, which may be responsible for the development of BS^100^. The overall secondary structure remained consistent throughout the simulations of mutants as well as of the native structure within the simulated timescale (Fig. S4).

### Relation between BLM dysregulation and survival analysis in cancer

**Figure 8:**
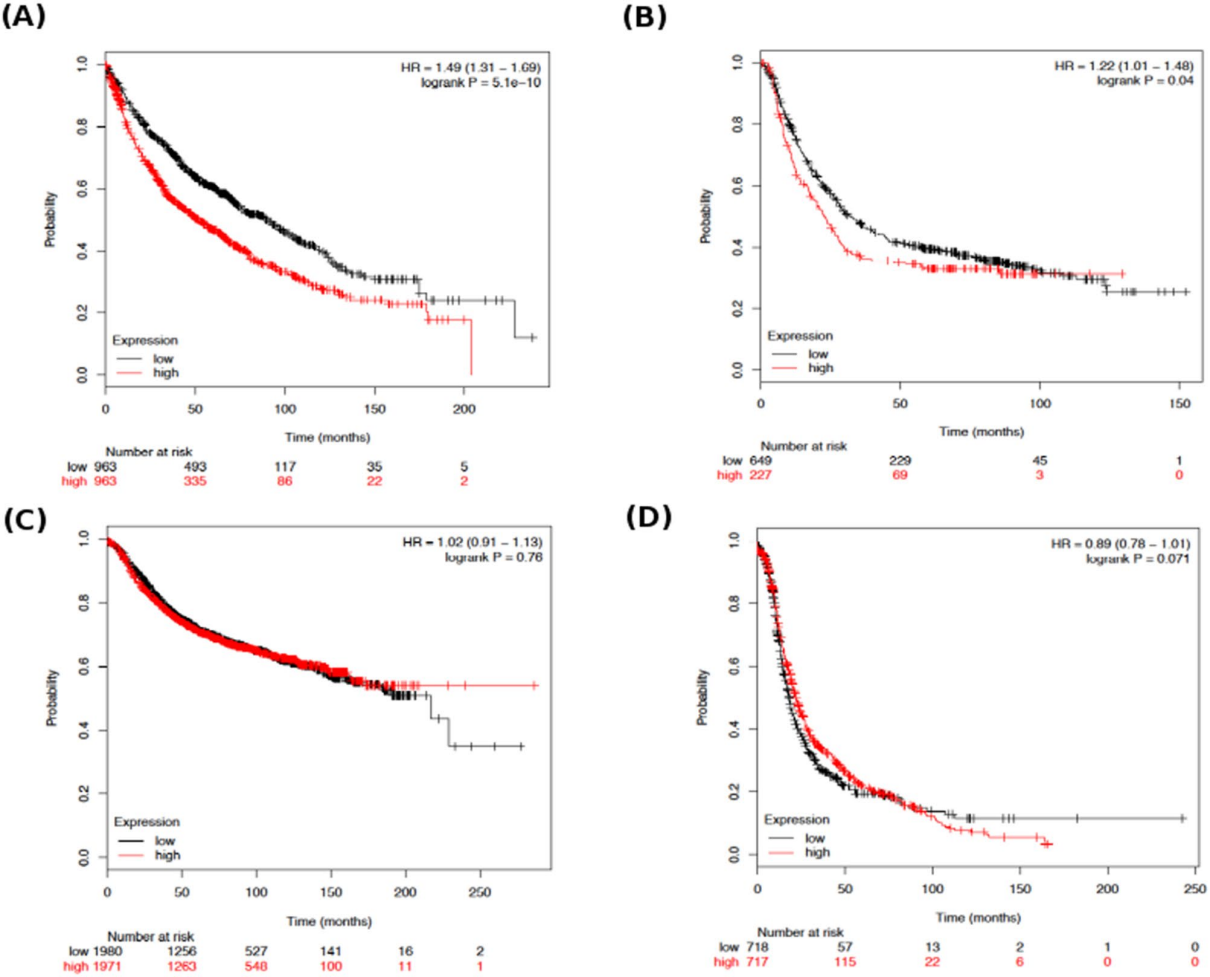
Kaplan**–**Meier curves showing the association of BLM mRNA expression and survival of patients in four cancers: (**A**) lung, (**B**) gastric, (**C**) breast, and (**D**) ovarian cancer.

To infer the functional consequences of BLM deregulation, we studied the relationship between the dysregulation of BLM and the clinical database of cancer patients. Using Kaplan–Meier plot analysis, we found that BLM dysregulation has distinct consequences in different types of cancers. High expression of the BLM gene (Affy probe id 205733_at) is associated with lower overall survival for lung and gastric cancer patients, whereas we did not find a significant association between expression of the BLM gene and overall survival for breast and ovarian cancer patients (Fig. 8).

Mutation in the BLM gene in BL cells showed a reduction in the expression of its mRNA and protein, resulting in excessive chromosome instability and a high frequency of sister chromatid exchanges (SCEs)^101^. However, another study found that BLM mRNA overexpression is associated with poor survival of breast cancer patients^102^. Interestingly, the same study reported that subcellular localization of the BLM protein is found to be high in the cytoplasm compared to the nucleus, although the analysis of normal breast tissue revealed that BLM protein is strongly localized in the nucleus and not the cytoplasm^102^. It might be possible that variants of the BLM gene express a truncated BLM, which lacks signals for protein localization.

Therefore, this study also indicates the importance of BLM gene expression as a better prognostic marker for the detection of gastric and lung cancers. Studies have found that nsSNPs can affect the functional activity of proteins; therefore, we expect that the 18 nsSNPs identified here have functional effects, as in BLM deregulation.

### Expression levels of BLM genes in different cancers

**Figure 9:**
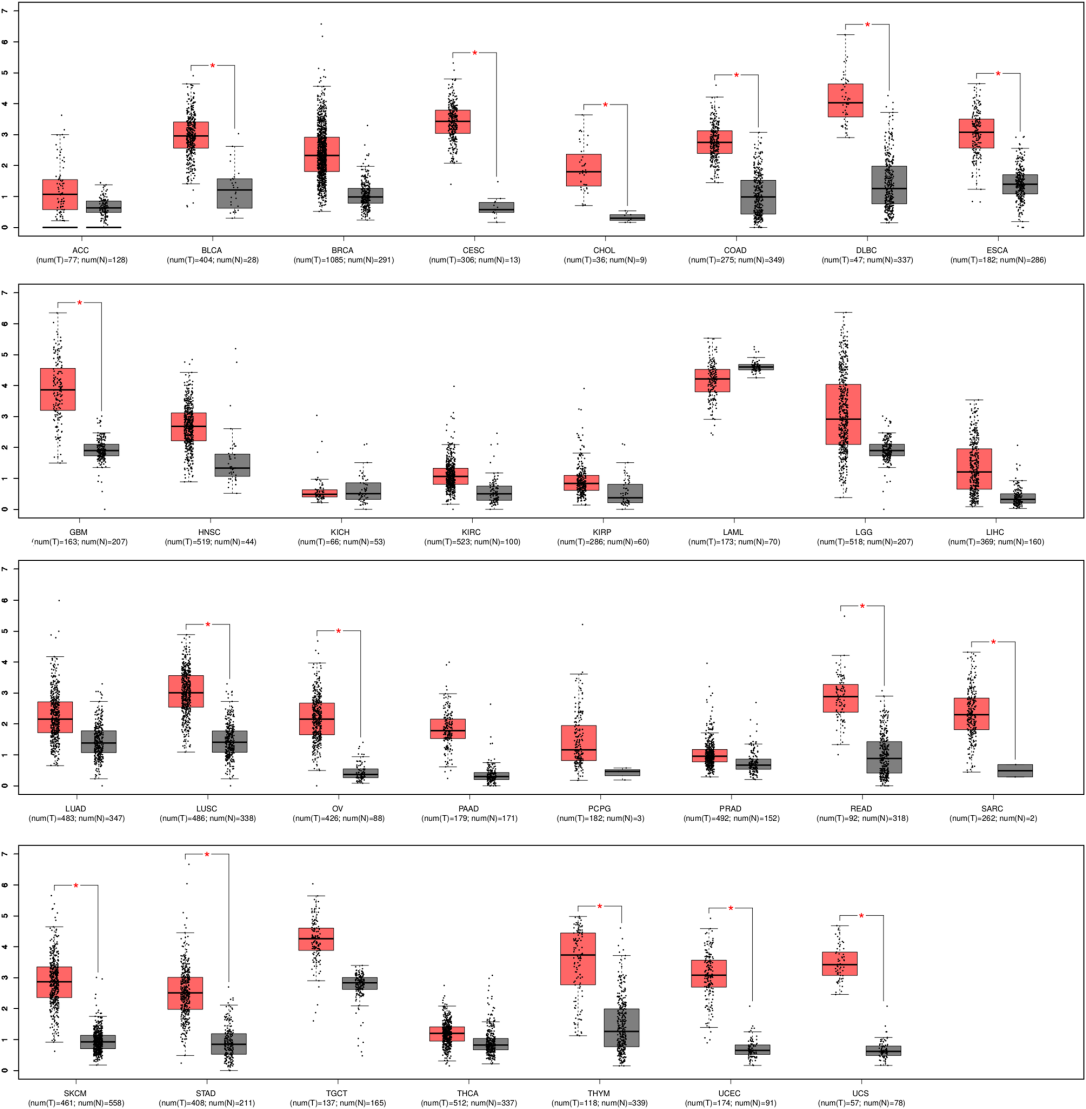
BLM mRNA expression level in normal tissues (N) and different cancer (T) samples. In each plot, y-axis indicates gene expression score calculated by mean value of log_2_(TPM + 1). The red box indicates the tumor samples while the gray box represents the normal samples, and the number of samples is given in brackets. The significant differential expression of mRNA between tumor and normal samples and indicates with symbol “*” with p-value < 0.01. *ACC* Adrenocortical, *BLCA* Bladder Urothelial Carcinoma, *BRC*A Breast invasive carcinoma, *CESC* Cervical squamous cell carcinoma and endocervical adenocarcinoma, *CHOL* Cholangio carcinoma, *COA*D Colon adenocarcinoma, *DLBC* Lymphoid Neoplasm Diffuse Large B-cell Lymphoma, *ESCA* Esophageal carcinoma, *GBM* Glioblastoma multiforme, *HNSC* Head and Neck squamous cell carcinoma, *KICH* Kidney Chromophobe, *KIRC* Kidney renal clear cell carcinoma, *KIRP* Kidney renal papillary cell carcinoma, *LAML* Acute Myeloid Leukemia, *LGG* Brain Lower Grade Glioma, *LIHC* Liver hepatocellular carcinoma, *LUAD* Lung adenocarcinoma, *LUSC* Lung squamous cell carcinoma, *OV* Ovarian serous cystadenocarcinoma, *PAA*D Pancreatic adenocarcinoma, *PCP*G Pheochromocytoma and Paraganglioma, *PRA*D Prostate adenocarcinoma, *READ* Rectum adenocarcinoma, *SARC* Sarcoma, *SKCM* Skin Cutaneous Melanoma, STAD Stomach adenocarcinoma, *TGCT* Testicular Germ Cell Tumors, *THCA* (Thyroid carcinoma, *THY*M Thymoma, *UCEC* Uterine Corpus Endometrial Carcinoma, *UCS* Uterine Carcinosarcoma.

To understand the role of the BLM gene in cancer, we studied the its expression levels in different cancers. A box plot was generated using GEPIA (|Log2FC| cut-off = 1.5; p-value cut-off = 0.01), which showed that the expression of BLM significantly increased in different cancer patients compared to normal expression (Fig. 9). Therefore, based upon this data, we propose the role of BLM as a diagnostic marker for several cancers.

### Impact of 3′UTR SNPs in BLM gene regulation

Earlier studies have shown the role of SNP in the 3′UTR of mRNA, which may lead to the partial or complete attenuation of complementary binding of miRNAs to the 3′UTR region^19^. On the other hand, SNPs in the 3′UTR region of mRNA can introduce new binding sites for other new miRNAs. We analysed the impact of gene regulation by miRNA due to SNPs in BLM mRNA using PolymiRTS v3.0. A list of 17 #rsIDs mapped to the 3′UTR and was fed into the PolymiRTS server, which yielded a list of binding miRNAs affected by these polymorphisms. The results showed that the binding of hsa-miR-6507-5p and hsa-miR-3976 to BLM mRNA was abolished due to polymorphisms rs116293756 and rs28363374, respectively (Table 6). Therefore, due to these SNPs, the BLM genes were not under the control of hsa-miR-6507-5p and hsa-miR-3976, which might result in higher expression of the BLM protein. In past decades, small interfering RNAs (siRNAs) have been widely used to silence the expression of target genes^103,104^. Designing allele-specific siRNAs using desiRm webserver (https://crdd.osdd.net/raghava/desirm/) against disease-causing SNPs would be a very promising therapeutic approach to suppress the overexpression of the BLM mutant gene^105^.

**Table 6:** Predicting miRNA-mRNA binding disruption due to SNPs in the 3′UTR of the BLM gene using PolymiRTS v3.0.

dbSNP ID	Variant type	Wobble base pair	Ancestral Allele	Allele	miR ID	Conservation	miRSite	Function class	Exp support	Context + score change
rs116293756	SNP	N	T	T	hsa-miR-6507-5p	2	tgtTAT**T**CTTgtt	D	N	-0.094
rs28363374	SNP	N	C	C	hsa-miR-3976	2	actcgT**C**TCTATt	D	N	-0.19

Function Class D: The derived allele disrupts a conserved miRNA site.

Exp Support N: Predicted target site with no experimental support.

### The possible mechanism of defective BLM and cancer predisposition

DNA damage during the replication process is corrected by the HR pathway. The HR starts with the degradation of 5′-terminal of DSBs, which generates 3′-overhang ssDNA^106^. Subsequently, RAD51 recombinase assembles at the ssDNA breakpoint and catalyzes the HR and DNA repair. During the HR process, invasion of ssDNA into the homologous sequence, typically a sister chromatid that works as a template, synthesizes the damaged DNA. At the last stage of HR, BLM forms a complex along with other enzymes and helps to separate the repaired DNA from the template DNA strand, and in this way BLM helps to maintain the genomic integrity^107^.

Biochemical studies showed that the deletion of BLM enhances the assembly of RAD51 at the ssDNA break site, while overexpression of BLM disrupts the RAD51 assembly through its helicase activity^108,109^. Therefore, BLM works as a ‘anti-recombinase’ at early and late stages of HR by: (i) preventing HR by disrupting the RAD51 assembly on the ssDNA of DSBs to make sure that HR only occurs between sequences with high homology; and (ii) preventing SCEs by separating the repaired DNA from the template DNA strand that arises at the final stages of HR^110,111^.

Previous studies showed that overexpression of BLM displaces the localization of RAD51 to the sites of DNA damage^109,112^. The loss of RAD51 leads to insufficient HR resulting in genomic instability and DNA damage. However, defective BLM increases the hyperaccumulation of RAD51 at DNA damage site resulting in a high level of HR and high frequency of SCEs^106,111,112^. Therefore, our study supports previous findings that an aberration in the activity of BLM through pathogenic nsSNPs or high expression of BLM (Figs. 8 and 9) could lead to genomic instability and predisposition to different cancer types.

## Conclusion

This study identifies eighteen highly deleterious and damaging nsSNPs for BLM inactivation using various powerful bioinformatic tools. Four of these mutations (P702L, W805L, P825L, and Y974C) have been reported in the literature to have a partial effect on helicase activity^76^. In support of this hypothesis, our MD studies of these mutations suggest that they maintained structural integrity during the simulations. Combining computational approaches with literature mining, we identified 14 more pathogenic mutations: P690L, W803R, W803L, Y811C, G891V, G952V, G972V, G978V, C1036F, Y1044C, C1055Y, D1064V, and C1066Y. Interestingly, we observed that only six of these mutations strongly destabilize the helicase structure within the simulation time: G891V, G952V, G972V, G978V, C1036F, and C1055R. The remaining mutations either showed weak destabilizing effects and maintained the structural integrity of the helicase structure or showed similar structural deviations to those observed in the WT/native structure. However, one cannot rule out the possibility that these mutations may contribute to the likely allosteric effect, which the limited time scales of our simulation studies could not capture. Visualizing the time-evolution of domain-wise rmsd values, as a general trend, we observed that the mutations observed in the Zn subdomain not only destabilized the Zn subdomain, but also destabilized the nearby RQC domain as observed from their higher rmsd values. This suggests the allosteric effect of these mutations. One probable reason can be that in the crystal structure, Zn subdomain interacts with the DNA 3′ overhang, while RQC domain interacts with the upstream duplex DNA, and thus, the nucleic acid may induce changes in RQC domain when Zn subdomain is mutated. Nonetheless, these observations warrant the detailed comparative studies in the future with longer sampling time to ascertain the allosteric effect of mutations.

**Figure 10:**
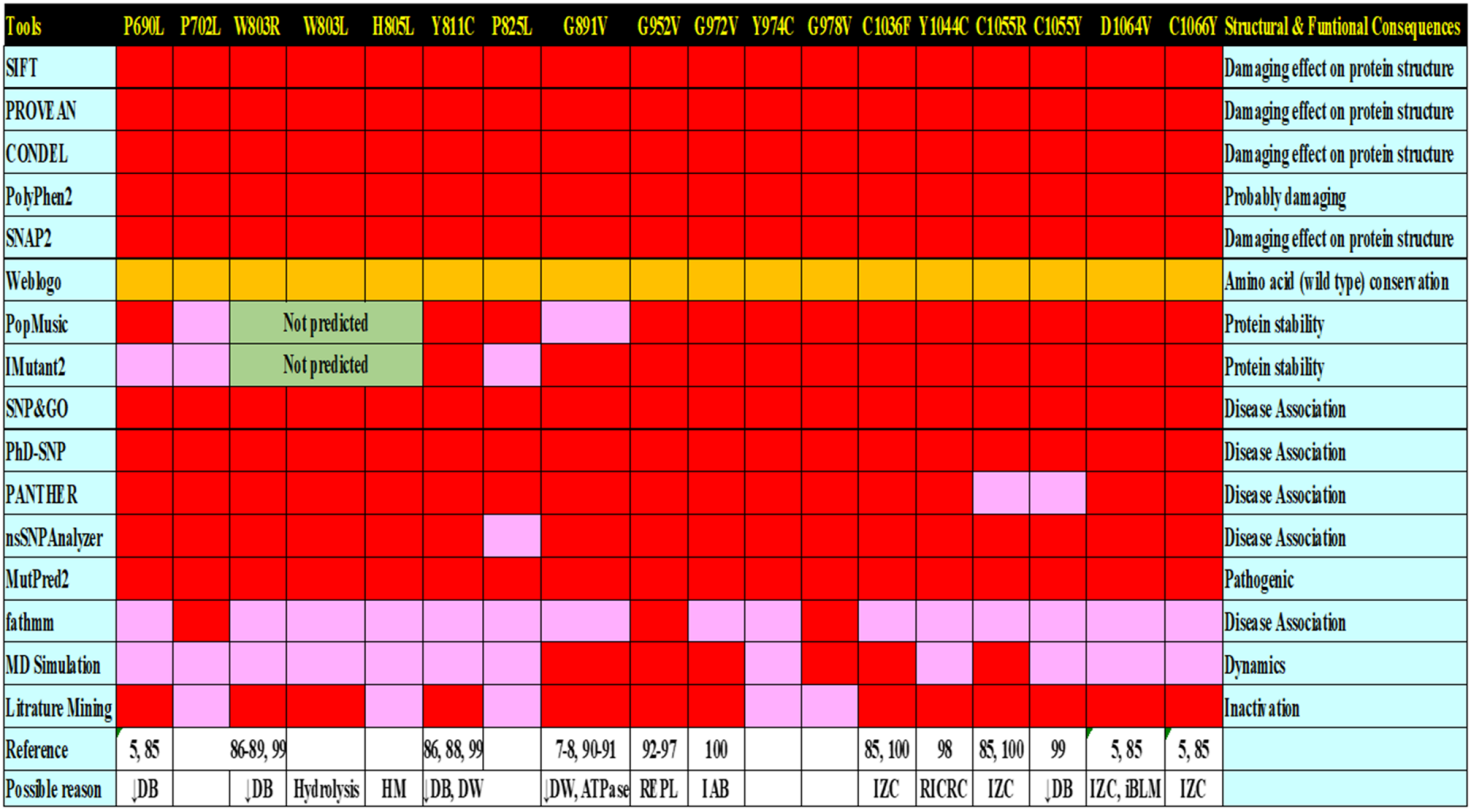
Structural functional consequences of nsSNPs on BLM helicase protein based on multiple computational tools prediction and literature mining. The first column represents the tool name, while the last column represents its use for predicting different structural and functional consequences. The mutant effects on W803R, W803L, and H805L was not predicted by IMutant2, and PoPMuSiC because the amino acid residues from 799–807 are missing in the BLM helicase crystal structure (PDB id: 4O3M). Red Box = damaging or disease associated, Green box = Not predicted by software because residue absent in crystal structure, Orange = highly conserved amino acid, Pink box = No damaging effect, DB = DNA Binding, IAB = Impaired ATP binding, DU = DNA Unwinding, IZC = Impaired Zn Coordination, AD = ATPase domain, RICRC = Reported in CRC patient, REPL = Replication, iBLM = deletion cause inactivation of BLM.

Since the MD simulation-based results suggest that G978V is pathogenic but no experimental evidence suggests its role in BS, future studies may sequence this pathogenic mutation site, especially in BS patients and other related diseases, and/or study it using mutational approaches using various models. Most of the pathogenic nsSNPs listed in the BLM protein suggest hampering the activity, such as ATP hydrolysis, DNA binding, and DNA unwinding, which is associated with various cellular procedures, such as replication, recombination, and DNA repair. BLM protein interacts with various factors for proper DNA replication and repair of DNA damage using the HR pathway. Turbulence during such a procedure always leads to the incorporation of erroneous copying of genomic information, giving rise to genomic instability and consequently leading to neoplastic transformation. Further future studies of BLM protein-related pathological nsSNPs will allow us to manage BS and its associated diseases. Figure 10 summarises our finding on pathogenic nsSNPs in BLM helicase.

Our study found 28 nsSNPs as “stop gained” and one nsSNP as “start lost” mutations, which can express a truncated form of BLM with the loss of important domains and nuclear signals and thus strongly localized BLM in the cytoplasm instead of the nucleus. As other studies have found, cancer cells have high cytoplasmic BLM, and it would be interesting to experimentally validate the nsSNPs with BLM protein localization in cancer cells. Furthermore, our investigation found that two SNPs, rs116293756 and rs28363374, at the 3′UTR of BLM abolished the binding affinity with the miRNAs hsa-miR-6507-5p and hsa-miR-3976, respectively, and thus enhanced the expression of BLM. Interestingly, we also found that BLM expression was significantly higher in some cancers including BLCA (Bladder Urothelial Carcinoma), COAD (Colon adenocarcinoma) and LUSC (Lung squamous cell carcinoma) (Fig. 9). Furthermore, Kaplan–Meier survival analysis suggested that high expression of the BLM gene is one of the reasons for the reduced survival of patients with lung or gastric cancer. Therefore, a deep understanding of how SNPs affect BLM transcription regulation and expression in cancer might be highly useful for the diagnosis and prognosis of disease.

In the future, our selected nsSNPs in the BLM gene can be further studied in different populations to explore and validate the contribution of these variants in BS and cancer, which may further lead to the design and development of potential drugs for the better management of BS and other associated diseases. Furthermore, our study provides key support to investigators to conduct future studies on pathological mutations and their structural consequences on BLM. The SNPs predicted from our study can be further validated by wet-lab scientists to investigate the evidence of BLM protein mutations in association to BS and develop a potential drug target for BS. These findings may enrich the available SNPs databases and then can be utilized for further research.

## Supplementary information

Supplementary information

Supplementary Figures

Supplementary Table S1

Supplementary Table S2

Supplementary Table S3

Supplementary Table S4

Supplementary Table S5

Supplementary Table S6

Supplementary Table S7
