# Supplementary material for: Investigating the pathogenic SNPs in BLM helicase and their biological consequences by computational approach
Source: Sci Rep. 2020 Jul 23;10:12377. doi: 10.1038/s41598-020-69033-8 (PMC7378827; doi:10.1038/s41598-020-69033-8)
Supplement: Supplementary file 1 — Supplementary information [file 41598_2020_69033_MOESM1_ESM.pdf]

Investigating the pathogenic SNPs in BLM helicase and their biological consequences by computational approach

Contributors of the manuscript in terms of data generation, manuscript drafting, editing

Faisal A. Alzahrani[#1,2], Firoz Ahmed[#*3,4], Monika Sharma[#5], Mohd Rehan[6,7], Maryam Mahfuz[8], Mohammed N. Baeshen[9], Yousef Hawsawi[10,] Ahmed Almatrafi[11], Suliman Abdallah Alsagaby[12], Mohammad Azhar Kamal[3,4], Mohiuddin Khan Warsi[3,4], Hani Choudhry[13], Mohammad Sarwar Jamal[*6,7,14]

# *Equal Contribution*
*** *Corresponding Author***

1. Department of Biochemistry, Faculty of Science, stem cells Unit, King Fahd Medical Research Center, King Abdulaziz University, Jeddah 21589, Saudi Arabia.
2. Aston Medical Research Institute, Aston Medical School, Aston University, Birmingham B4 7ET, UK.
3. Department of Biochemistry, College of Science, University of Jeddah, PO Box 80327, Jeddah, Saudi Arabia.
4. University of Jeddah Centre for Scientific and Medical Research (UJ-CSMR), University of Jeddah, Jeddah, Saudi Arabia.
5. Department of Chemical Sciences, Indian Institute of Science Education and Research (IISER), Mohali, India
6. King Fahd Medical Research Center, King Abdulaziz University, Jeddah, Saudi Arabia.
7. Department of Medical Laboratory Technology, Faculty of Applied Medical Sciences, King Abdulaziz University, Jeddah, Saudi Arabia
8. Department of Computer Science, Jamia Millia Islamia, New Delhi, Delhi, India
9. Department of Biology, College of Science, University of Jeddah, PO Box 80327, Jeddah, Saudi Arabia.
10. Department of Genetics, Research Center, King Faisal Specialist Hospital, and Research Center, MBC-03, PO Box 3354, Riyadh, 11211, Kingdom of Saudi Arabia.
11. Department of Biology, Faculty of Science, University of Taibah, Medinah, Saudi Arabia
12. Department of Medical Laboratories, Central Biosciences Research Laboratories, College of Science in Al Zulfi, Majmaah University, Kingdom of Saudi Arabia
13. Department of Biochemistry, Cancer Metabolism and Epigenetic Unit, Faculty of Science; Cancer and Mutagenesis Unit, King Fahd Center for Medical Research; King Abdulaziz University, Jeddah, Saudi Arabia.
14. Integrative Biosciences Center, Wayne State University, Detroit, MI 48202, USA

@ Corresponding Author:
Dr. Mohammad Sarwar Jamal (Email: sarwar4u@gmail.com & hd2149@wayne.edu)
Dr. Firoz Ahmed (Email: fahmed1@uj.edu.sa)

```
>P54132
--------------------------------MAAVPQNNLQEQL---------------
------------------------------------------------------------
--ERHS-ARTLNNKLS--LSKPKF--------SGFT------------------------
---------FKKKTSSDNN---VSVTNV--------SVAKTPVLR--------NKDVNVT
EDFSFSEPLP-NTTNQQ-----RVKDFFKNAPAGQETQRGGS------KSLLPDFLQT--
--PKEV-VCTT--Q---NTPTVKK----SRDTALKKLEFSSSPDSLSTI-NDWDDM-DDF
D--TSETSKSFVTP----PQSHF--VRVSTAQKSKKGKRNFF--------KA---QL---
YTTN-------------TVKTDLPPPS--SESEQIDLTEEQKDD---------SEW---L
SSDVICI-DDGPIAEVHINE----------DAQESDSLKTHLEDER-------------D
NSEKKKNLEE--------AELHSTEK------VPCIEF----DDDDYDTDFVP---PSP-
EEII-------SASSSSS---------------KCLSTLKDLDTSDRKEDVLS-TSKDLL
SKPEKMSMQELNP--ETSTDCDARQISLQQQLIH--------------------------
----------------------VMEHICK-------------------------------
------------------------------------------LIDTIPDDKLKLLD----
---------------------CGNELLQQRNIR----------------------RKLLT
--E-VDFNKSDAS-LLG----SL----WRYRPD---------SLDGPM------------
-------------EGDSCPTGN--SMKELN-----------FSHLPSNSVSPGDC-----
------------------LLTTTLG---------------------------------KT
GFSA-TRKNLFERPLFNT-----------------HLQKSFVSSNWAETPRLGKKN----
----------ESSYFPGNVLTSTAVK----DQNKHTASI----------------NDL-E
RETQPSYDIDNFDI---DDF---DD---------DDDWEDIM-HNLA-------------
--ASKSSTAAYQPIK--EGRPIKSVSE-RL---SSAKTD------------------CLP
VSSTAQNINFSESIQN--YTDKSAQNLAS--RNLKH-----ERFQSL-----S-FPH---
------TKEMMKIFHKKFGLHNFRTNQLEAINAALLG--EDCFIL---MPT--GGGKSLC
--------YQLPA---------------------CV----SPGV-TVVISPLRSLIVDQV
QKLTS------LDI-----------------PATYLTGD---KTDSEA---TNIYLQLSK
KDP--IIKLLYVTPE-KICAS---------------NRLISTLENLYERKLLARFVIDEA
HCV--SQWG------HDFRQDYKRMNMLRQKF--PS-VPVMALTATAN----------PR
VQKDILTQLKIL----RPQVFSM--SFNRHNLKYYVL---PK-------------KPKKV
AF--DCLEWIRKH-HPY----DSGIIYC-----LSRREC--------DTM---------A
DTL---QRDGLAALA-YHAGLSDSAR-DEVQQKW----INQDG-CQ----VICATIAFGM
GIDKPDVRFVIHASLPKS-VEGYYQESGRAG-RDGEISHCLLF-YTYHDVTRLKRLI---
--------MMEKDG--NHHTR--------ETHFNNLYSM--VHYCENITE----------
CRRI---QLLAYFGE--NGFNPDFCKKHPDV--SCDNC--CK----TKDYKTR--DVTDD
VKSIVRFVQEHSS-----SQGMRNIKHVGP-SGRFTMNML-VD-IFLGSKSAKIQS----
------GIFGKGSAYSRHNAERLFKKLILDKILDEDLYINAN-DQAI--AYVMLGNKAQT
VLNGN----L-------------K---VDFMETENSSSVKKQK----------ALVAKVS
Q------------------------------------------------------RE--E
MVKKCLGELTEVCKSLGKVFGV-HYFNIFNTVTLKKLA--ESLSSDPEVLLQIDGVTEDK
LEKYGAEVISVLQKYSE----------------WTSPAEDSSP--GISLSSSRGP---GR
SAAEE-----LDEEIPV-------SSHYFASKTRNERKRK--KMPASQRSKRRKTAS-SG
SK--AKG---------GSATCRKI--SSKTKSSSIIGSSSASHTS-----------QATS
---------------------GANSK----------------------------------
------------------------------------------------------------
------------------------------------------------------------
------------------------------------------------------------
```


--LGIMAPP-
--KP--------------
---------INRPFLKPSYAFS-----------------
>A0A2A6DYU3

--------------------MPILKPFSECIARPPEDGRTYP------------------

-------------------------LPDHLIHASETMQRVIA------------------
-----------------AHPVLGADHALVRLGELAGFCHDVP------------------

---------------------------------KCHPEWQEYAEGRRQRGPAHAPAAAF-

--LFAALGIELLDKLD----
--------------------------AWKRNQR----------------------RWLW-
----LVRDIADHHGLLKDAVEDRWIQQWKLG-----------------------------
-------------EWERMDLDGVTELVHLL-----------FPELTHVRLAPDRF-----

----------RSWCSLVRQRFDEVNKSLRKGRSDAEYSV------------LMDELDEWR
FLTSALIAADRFDI--

--TPTPDARFDDTSVD-------------------------GLLDAI-----RRFCE---
-------------ARRDSPFSDVRAEAMAAIVRAFDGAGADCQFFALEMPT--GYGKTVV
S-------FLLAALL-------------------CKRNGLSK---IIYAAPYLSVLEQNS
NVLRD-----DLGMRPVEHHSLAVWDKELAKRADDVDQDGDRRSRDSRHVHRSDWTDDRF
DDSIERASARMLAAE-SWAHEIVCTSFPQLARAVFPRRAQDTLRRFYLRDAV--LLIDEP
QIFENRGW-------NLFLVGLKSLAR-------QNNLRVIFLSATM-----------PP
FQYGLEEEKPF------PLVFRP--SPDDRKIRYTAQ------------------KIDRT
MDERELAEWVLGR-EER----TQVVILN-----TIRDACLVY-----DQM---------K
KAS---TPHELHLLHGLMVPLHKKVKISRLESLLKCLSQNGDR-CRLPPQIAVSTQVLEA
GVDVSFQHGVRAASI----LPAYVQTGGRIN-RHFEIRGGGTL--------SIVRFL---
-----------RGG--KRDTR--------HPIYESALTGLTDELLENGAE----------
-------WPEPEFEELARIFYERMFREDSRE--ACKAAIMDA----ASGYWTR-------
LGGYEPFESDDFR----------------------------LP-LFVPWR----------
--------------YPKRDEPFLPRTFL--------------------------------
---QLAERFG
M--RD---
-PHQIYECMVDRRYWEGRDFGERKQFMVLANYYILNIP------------VRFAVEWKND
YLQHRIPLLSDPDAYSDE---------------IGFTRVADVS-----------------

----------GDDVWII---

>A0A151VN02

--MPDVNPHEQIHVKS----
------------------------QLLLEKARD---------------------------

-----------------AAAKERKYDSAKT------------------------------

------RQDLTRLFREQSGNVPYDW-QLDVTEATLLG--LDSVII---AGT--GSGKTIP
--------FMLPL---------------------LL----HPKKLMLIISPLKVLQKDQV
RRFKK------MKI-----------------PAVAVNGETWSPSLGQS---------LAD
KKH----QAIFSSPE-MCLKH---------------PEFRKWLSDPETTKDMLGIIVDEA
HCI--SQWG------GDFRPLYADVQKLRALM--PSHIPVLLTSATLN----------PP
ALKEVCSKMEVD----LNSSFFLNRGNHRINITSSVIRMDGAK------------DFAAV
REHLPCPDQVTSP-EDF----KKTIIFT-NVVNDTQILC--------RDL---------R
KHYDNKYDDSIAY---LHANRTTKAK-RRIM-KW----FLKGK-IK----ILIATEAAGM
GADIPDIELIIQFGVPKS-LTIWTQRAGRGG-RSSDMQAEAKM-LVEKSMFETKRKKKPG
GKKPTTTKKTKKRRKTIEHDTQQANEAVANAEEEALIDAEEMEWVKNVDEDMRKYAGTEN
CRTD---VADSFFNNPPRAPVPGAVGCQ-----QCDNCLRKRQVLLSASMFD---DTQEQ

LSRPSTPTFPNSLPSTPSSSAPPSPSKLLNENGKRGMVSR-TP-IDLGETPLNDDPSSIK
PSTDRTDETVQATNTTTASAGSTSTELDLPGPEDAALTASAP------AKSTRRGEHLQA
IRSSLYAWRL-------------DIQRSRYSPSPFPAEVYLPEPVLTTLSSNRAIKTLDD
M--RV--A
LLKRPWIYLEEHGKEILDLIKV-TDQRFIEGRQQEAHAKKEARKQATAERRQAAEREKEQ
ATLNRRKMQGVHR--------------------RRSPLIDHTPLLNGPHLGLPLTSMSST
SAPIWPSHPAIPSNDQNTFGLSSTSHSYPRVHPIAGPS----LGPPQHYYQLQNMTPRGS
QVMHPSP------------YFMPLPYPPTPFSVPSTSPPCSVTNP---------------

--YELYFPP-

--------VYHAPSSRPPDS-------------------
>A0A2B4R558

-------MVSDEQKRW--

----------------------LVTIYALNCVLGPHLKLVVE------------------
--EGMKELYKFL
D------------------------DKLNGLVKPCALRSLTY------------------
-----------------DVVYSSPPLDFLQDLKFVNINRNFYRK----------------
---D
ESNYMYNVPK---------------------------------IEDLAKLYLPTDVPA--
---------------------------------KFSKFDKNMDVAVAIKLLGYEYQKEIF
SSEVQSSANDVRKNVRNECDHFREREWTESFFNK--------------------------
----------------------CFDKLCR-------------------------------
--LVVCLPLAEAEKKD----
-------------------------IEELSKWQ----------------------VEVMT
SKDNEDVLLNGEEYEEDLNDIPI-------------------------------------
--------DENEQNGESTQTGK-------------------DAMLPSHASLSGPS-----
---QAT
ALSSSEIAGLKEAILMLT-----------------QKHQNPLLGDNEPAKNKSTSE----
----------KHLADQRPNMAAPAKK----SKPDATDPE-----------------RPCS
MSTPNDCQILYDSV---LNY--------------TSDKDAAKTPLIA-------------
--TAKKRMISSPNSS--RMINAKSLNEEDC---QNMKKL------------------GLE
SMSLLTHESYKLNMQRRMLMRSDIGKEY---GSPCS----------------SQLPF---
------TEFLFGDDLQKRLTDIGDQNKIGAKITPNKG--KHRYDK---Y----GSGTSSC
NENYNAKKLERPE---------------------------SQAL-EAQKEPGQQVQQPSL
AYVKK------KRI-----------------KAAVVVGP---DSSQTK----------NK
EATEGKYNLVFTSPKALFGSH---------------QSTVLTLKN-----KIEAVFIDEV
HCV--AKWGHGSGNDEVFLDDYARLAEMRLFFK-QV-TPLIALTATAT----------DA

```
VRKVIMRDLMMR----DCVQFIT--VPNKQNIRFSVSSVDPD-----------------D
LE--TSFQWLIDDLKSKKINAQKVLIFC-----IKRAHLRELYETFHEVLGSNSYGPPTG
KEL---KDDRTRLFAMYHRKTHPLVK-ETVETEF----CKVNGSVR----VVFCTIAFGM
GIN----------------VEGAYIA------LHLGPSCCL---AAYSGCTRSKNIT---
------------------------------------NGM--KEYVRNTDV----------
RRHS---LLIQPFLN---------CQEKPDE--------ETR----IERHSCR--DVCAK
TCRCLCLCKGKCT----------------------------RD-----------------
---------------NVCNSEWFF------------------------------------
-----------------------------------------------------SPMEAIS
E------------------------------------------------------CNIVK
MIEQCSSDY---------------------------------------------------
------------------------------------------------------------
----------DTDDSDI-----------------------------YIRSRICHSQY---
------------------------------------------------------------
------------------------------------------------------------
------------------------------------------------------------
------------------------------------------------------------
------------------------------------------------------------
------------------------------------------------------------
------------------------------------------------------------
------------------------------------------------------------
------------------------------------------------------------
------------------------------------------------------------
---------------------------------------
>A0A0B7F353
------------------------------------------------------------
------------------------------------------------------------
------------------------------------------------------------
------------------------------------------------------------
------------------------------------------------------------
------------------------------------------------------------
------------------------------------------------------------
------------------------------------------------------------
------------------------------------------------------------
--------------------------------------------------------MPPT
SHTP--------------------------------------------------------
------------------------------------------------------------
------------------------------------------------------------
------------------------------------------------------------
-----------------------DATRFSRHQR---------------------------
------------------------------------------------------------
-----------------------------------------SDATPYSRPYAQEC-----
------------------------------------------------------------
------------------------------------------------------------
------------------------------------------------------------
------------------------------------------------------------
------------------------------------------------------------
```

---------------AKLGIKRLHRMQRGVIDDIVNNPEKDQFII---AAT--GSGKTLL
--------YELPG---------------------ILPS--SSGKTTIVFIPRVSIITVEH
KRLFD----CGVSV-----------------EKRHARNDNQQEREQQAQQNDRLFQAVNN
TEL--LPRFILATPNQLLYSD---------------SIFSKILSGLCERGLVYRFVFDEV
HML------------LDDHTVLSQFPILRQKY--PD-VPVTVLSASVA----------PA
TTSTLCRALSMG---GEPKVF----PLDRPNLYYQVL---PKLSQGEDDKLGVAATPKER
SQMSPILHLARNV-YPD----LAGLVYC-----RKKSTC--------ARF---------A
ELL---KKEGIAAEA-YDADSNRSVMGREIFRKW----QENDPDVR----ILISTNALSS
GVHKSDVRFVVHTSLPTSGIDGYMQETGRAG-RDGEPATCLLL-YAFGDAFQVPHHT---
-----------------------------QFQTNCILAL---LWLINSTN----------
CRRR---ALLSYYDD--NHFNYEATSHR-----CCDVC---------------------D
GMASGRPPLEITS--------------------------------LVGRVLEYIQD----
---------------GHEEKHGPVGRIRLTRILSDRFNNGEK------------------

---E
PTADMWDKLIQ---
---------------------------------WLVIEQYLEV-----------------
---------------------------HRPGEKAGGQIRV--------------------

>A0A1A8HPI0

--------------------FSLKHEQIEALKSFILK--KDVFAA---LPT--GYGKSLI
--------YQLAPVV-------------------AKRMGLSENPLVVIVSPLVALMEDQV
KEASK------LGL-----------------TAMQLG--------------VHTLKDIRE
ANC----QLIFGSPE-AWLMQ---------------SKWSEMLSTKAFRDNLLGIVVDEV
HLI--YKWGKADKGNKAFRECFAKLGELRSIV--KRGTPILALTATAD----------LD
SRAIIQNQLHFE----DATQVTI--SPNRKNIRFGLI------------------KVSSH
SF--DCLDWIVRELKERDTNMFQVIIYCRTLTAVGRVFCYLKAELGEDCW---------A
DKDPQRRAENLRIGM-FHSHTLPQNK-RRILNSF----GGDGP-CR----VVVATSALGM
GLNFPKISHVVMYGVPED-MEAILQEAGRAG-RDGSLSHAVIY--------RLNLNT---
--------NVDEGL---------------RTLLENSSKS---------------------
CFRK---ALFSHFENNVESVEPGHL--------CCTYCHSVC-------------SCTSA
GCDMAVPTYEQAP----------------------------LE-VSAPVQIREVTE----
--------------DQKDLIQNLLHNYKLSLIPETHLYTNST------------------
YCTGF---
---SD--E
LINSVL----EHCTEIFDLNFIMNNLPVFSKKHAQEIL----------------RIFFEV
FEDF---------KFTE----------------LDFPPDDFVV-----------------
----------PDPN----------YMGYFDEDTEGNVLSR--------------------
---------------------------CSSLESGLSALSTDSQNN---------------

>A0A1A8EW96

---MAAAPEQRSFETAL-----

-----SSTLEDLDL--

---GF-------------
---------------------SLKHEQIEALKSFILK--KDVFAA---LPT--GYGKSLI
--------YQLAPVV-------------------AKRMGLSENPLVVIVSPLVALMEDQV
KEASK------LGL-----------------TAMQLG--------------VHTLKDIRE
ANC----QLIFGSPE-AWLMQ---------------SKWSEMLSTKAFRDNLLGIVVDEV
HLI--YKWGKADKGNKAFRECFAKLGELRSIV--KRGTPILALTATAD----------LD
SRAIIQNQLHFE----DATQVTI--SPNRKNIRFGLI------------------KVSSH
SF--DCLDWIVRELKERDTNMFQVIIYCRTLTAVGRVFCYLKAELGEDCW---------A
DKDPQRRAENLRIGM-FHSHTLPQNK-RRILDSF----GGDGP-CR----VVVATSALGM
GLNFPKISHVVMYGVPED-MEAILQEAGRAG-RDGSLSHAVIY--------RLNLNT---
--------NVDEGL---------------RTLLKNSSKS---------------------
CFRK---ALFSHFENNVESVEPGHL--------CCTYCHSVC-------------SCTSA
GCDMAVPTYEQAP----------------------------LE-VSAPVQIREVTE----
--------------DQKDLIQNLLHNYKLSLIPETHLYTNST------------------
YCTGF---
---SD--E
LINSVL----EHCTEIFDLNFIMNNLPVFSKKHAQEIL----------------RIFFEV
FEDF---------KFTE----------------LDFPPDDFVV-----------------
----------PDPN----------YMGYFDEDTEGNVLSR--------------------
---------------------------CSSLESGLSALSTDSQNN---------------

>A0A1A8REU7


```
------------------------------------------------------------
------------------------------------------------------------
------------------------------------------------------------
------------------------------------------------------------
------------------------------------------------------------
------------------------------------------------------------
------------------------------------------------------------
------------------------------------------------------------
------------------------------------------------------------
------------------------------------------------------------
------------------------------------------------------------
------------------------------------------------------------
------------------------------------------------------------
------------------------------------------------------------
------------------------------------------------------------
------------------------------------------------------------
------------DL----------------------------------------------
------------------------------------------------------------
---------------------------------------------GF-------------
---------------------SLKHEQIEALKSFIFQ--KDVFAA---LPT--GYGKSLI
--------YQLAPVV-------------------AKRMGLSENPLVVIVSPLIALMEDQV
KEASK------LGL-----------------TAMQLG--------------VHTLKDIRK
ANC----QLIFGSPE-AWLMQ---------------SKWREMLSTKTFRDNLLGIVVDEV
HLI--YKWGKADKGQKAFRECFAKLGELRSIV--RRGTPILALTATAD----------LD
SRAIIQKQLHFE----DATQVTV--SPNRKNIRFGLI------------------KVSSH
TF--DCLDWIVRELKEHDTNMFQVIIYCRTLTAVGRVFCYLKAELGEDCW---------A
DKDPQRRAENLRIGM-FHSHTLPQNK-RRILDSF----GGDGP-CR----VVVATSALGM
GLNFPKISHVVMYGVPED-MEAILQEAGRAG-RDGSLSHAVIY--------RLNLNT---
--------NVDEGL---------------RTLLKNSSKS---------------------
CFRK---ALFSQFENNTDSVVPGHL--------CCTYCHSVC-------------SCTSA
GCDMAVPKYEQAP----------------------------LE-VSAPVQIREVTE----
--------------DEKDLIRNLLHTYKLSLIPETHLYTNST------------------
YCTGF-------------------------------------------------------
-------------------------------------------------------SD--E
LINSVL----EHCAEIFDLNFIMNNLPVFSKRHAQEIL----------------RILFEV
FEDF--------KFTE-----------------LDFPPEDFVV-----------------
----------PDPN----------YMGYFDEETEGNVLSQ--------------------
---------------------------CSSLESGLSALSTDSQNN---------------
------------------------------------------------------------
------------------------------------------------------------
------------------------------------------------------------
------------------------------------------------------------
------------------------------------------------------------
------------------------------------------------------------
------------------------------------------------------------
------------------------------------------------------------
------------------------------------------------------------
```

>A0A1A8RI11

--DVFAA---LPT--GYGKSLI
--------YQLAPVV-------------------AKRMGLSENPLVVIVSPLIARMEDQV
KEASK------LGL-----------------TAMQLG--------------VHTLKDIRK
ANC----QLIFGSPE-AWLMQ---------------SKWREMLSTKTFRDNLLGIVVDEV
HLI--YKWGKADKGHKAFRECFAKLGELRSIV--RRGTPILALTATAD----------LD
SRAIIQKQLHFE----DATQVTV--SPNRKNIRFGLI------------------KVSSH
TF--DCLDWIVRELKEHDTNMFQVIIYCRTLTAVGRVFCYLKAELGEDCW---------A
DKDPQRRAENLRIGM-FHSHTLPQNK-RRILDSF----GGDGP-CR----VVVATSALGM
GLNFPKISHVVMYGVPED-MEAILQEAGRAG-RDGSLSHAVIY--------RLNLNT---
--------NVDEGL---------------RTLLKNSSKS---------------------
CFRK---ALFSQFENNTDSVVPGHL--------CCTYCHSVC-------------SCTSA
GCDMAVPKYEQAP----------------------------LE-VSAPVQIREVTE----
--------------DEKDLIRNLLHTYKLSLIPETHLYTNST------------------
YCTGF---
---SD--E
LINSVL----EHCAEIFDLNFIMNNLPVFSKRHAQEIL----------------RILFEV
FEDF---------KFTE----------------LDFPPEDFVV-----------------
----------PDPN----------YMGYFDEETEGNVLSQ--------------------
---------------------------CSSLESGLSALSTDSQNN---------------

>A0A1A8MEN9

--------MQ--

------------------------------------SKWRELLSTKTFRDNLLGIVVDEV
HLI--YKWGKADKGHKAFRECFAKLGELRSIV--RRGTPILALTATAD----------LD
SRAIIQKQLHFE----DATQVTV--SPNRKNIRFGLI------------------KVSSH
TL--DCLDWIVRELKEHDTNMFQVIIYCRTLAAVGRVFCYLKAELGEDCW---------A
DKDPQRRAENLRIGM-FHSHTLPQNK-RRILDSF----GGDGP-CR----VVVATSAPGM
GLNFPKISHVVMYGVPED-MEAILQEAGRAG-RDGSLSHAVIY--------RLNLNT---
--------NVDEGL---------------RTLLKNSSKS---------------------
CFRK---ALFSQFENNTDSVEPGHL--------CCTYCHSVC-------------SCTSA
GCDMAVPKYEQAP----------------------------LE-VSAPVQIRKVTE----
--------------DEKDLIRSLLHTYKLSLIPETHLYTNST------------------
YCTGF---
---SD--E
LINSVL----EHCAEIFDLNFIMNNLPVFSKRHAQEIL----------------RILFEV

FEDF---------KFTE----------------LDFPPEDFVV-----------------
----------PDPN----------YMGYFDEDTEGNVLS---------------------

>A0A1A8PCT4

--T--GYGKSLI
--------YQLAPVV-------------------AKRMGLSENPLVVIVSPLIALMEDQV
KEASK------LGL-----------------TAMQLG--------------VHTLKDIRK
ANC----QLIFGSPE-AWLMQ---------------SKWRELLSTKTFRDNLLGIVVDEV
HLI--YKWGKADKGHKAFRECFAKLGELRSIV--RRGTPILALTATAD----------LD
SRAIIQKQLHFE----DATQVTV--SPNRKNIRFGLI------------------KVSSH
TL--DCLDWIVRELKEHDTNMFQVIIYCRTLAAVGRVFCYLKAELGEDCW---------A
DKDPQRRAENLRIGM-FHSHTLPQNK-RRILDSF----GGDGP-CR----VVVATSAPGM
GLNFPKISHVVMYGVPED-MEAILQEAGRAG-RDGSLSHAVIY--------RLNLNT---
--------NVDEGL---------------RTLLKNSSKS---------------------

CFRK---ALFSQFENNTDSVEPGHL--------CCTYCHSVC-------------SCTSA
GCDMAVPKYEQAP----------------------------LE-VSAPVQIREVTE----
--------------DEKDLIRSLLHTYKLSLIPETHLYTNST------------------
YCTGF---
---SD--E
LINSVL----EHCAEIFDLNFIMNNLPVF-------------------------------

>A0A1A8V8G3

---GF-------------
---------------------SLKHEQIEALKSFIFK--KDVFAA---LPT--GYGKSLI
--------YQLAPVV-------------------AKRMGLSENPLVVVVSPLVALMEDQV
KEASK------LGL-----------------TAMQLG--------------VHTLKDIRK
ANC----QLIFGSPE-AWLMQ---------------SKWRELLSTKAFRDNLLGIVVDEV

```
HLI--YKWGKADKGHKAFRECFAKLGELRSIV--RRGTPILVLTATAD----------LD
SRAIIQKQLHFE----DATQVTV--SPNRKNIRFGLI------------------TVSSH
TF--DCLDWIVRELKEHDTNMFQVIIYCRTLTAVGRVFCYLKAELGEDCW---------A
DKDPQRRAENLRIGM-FHSHTLPQNK-RRILDSF----GGDGP-CR----VVVATSALGM
GLNFPKISHVVMYGVPED-VEAILLEAGKAG-RDGSLSHAVIY--------RLNLNT---
--------NVDEGL---------------RTLLKNSSKS---------------------
CFRK---ALFSQFENDTDSVVPGHL--------CCTYCHSVC-------------SCTSA
GCDMAVPKYEQAP----------------------------LE-VSAPGQIREVTE----
--------------DEKDLIQSLLHTYQLSLNPGSRLYTNSS------------------
YCTGF-------------------------------------------------------
-------------------------------------------------------SD--E
LINAVL----EHCAEIFDLNFIMNNLPVFSKRHAQEIL----------------RILFEV
FEDF---------KFTE----------------LDFPPEDFVV-----------------
----------PDPN----------YTGYFDEDTEGTGLS---------------------
------------------------------------------------------------
------------------------------------------------------------
------------------------------------------------------------
------------------------------------------------------------
------------------------------------------------------------
------------------------------------------------------------
------------------------------------------------------------
------------------------------------------------------------
------------------------------------------------------------
------------------------------------------------------------
---------------------------------------
>A0A1A8B8R2
------------------------------------------------------------
------------------------------------------------------------
------------------------------------------------------------
------------------------------------------------------------
------------------------------------------------------------
------------------------------------------------------------
------------------------------------------------------------
------------------------------------------------------------
------------------------------------------------------------
------------------------------------------------------------
------------------------------------------------------------
------------------------------------------------------------
------------------------------------------------------------
------------------------------------------MAAALEQRSFEAAL----
------------------------------------------------------------
------------------------------------------------------------
------------------------------------------------------------
------------------------------------------------------------
------------------------------------------------------------
------------------------------------------------------------
-----SATLDDLDL----------------------------------------------
```

---GF-------------
---------------------SLKHEQIEALKSFIFK--KDVFAA---LPT--GYGKSLI
--------YQLAPVV-------------------AKRMGLSENPLVVVVSPLVALMEDQV
KEASK------LGL-----------------TAMQLG--------------VHTLKDIRK
ANC----QLIFGSPE-AWLMQ---------------SKWRELLSTKAFRDNLLGIVVDEV
HLI--YKWGKADKGHKAFRECFAKLGELRSIV--RRGTPILVLTATAD----------LD
SRAIIQKQLHFE----DATQVTV--SPNRKNIRFGLI------------------TVSSH
TF--DCLDWIVRELKEHDTNMFQVIIYCRTLTAVGRVFCYLKAELGEDCW---------A
DKDPQRRAENLRIGM-FHSHTLPQNK-RRILDSF----GGDGP-CR----VVVATSALGM
GLNFPKISHVVMYGVPED-VEAILLEAGKAG-RDGSLSHAVIY--------RLNLNT---
--------NVDEGL---------------RTLLKNSSKS---------------------
CFRK---ALFSQFENDTDSVVPGHL--------CCTYCHSVC-------------SCTSA
GCDMAVPKYEQAP----------------------------LE-VSAPGQIREVTE----
--------------DEKDLIQSLLHTYQLSLNPGSRLYTNSS------------------
YCTGF---
---SD--E
LINAVL----EHCAEIFDLNFIMNNLPVFSKRHAQEIL----------------RILFEV
FEDF---------KFTE----------------LDFPPEDFVV-----------------
----------PDPN----------YTGYFDEDTEGTGLS---------------------

>A0A1A8CX63

-----------LDL--

---GF-------------
---------------------SLKHEQIEALKSFIFK--KDVFAA---LPT--GYGKSLI
--------YQLAPVV-------------------AKRMGLSENPLVVIVSPLIALMEDQV
KEASK------LGL-----------------TAMQLG--------------VHTLKDIRK
ANC----QLIFGSPE-AWLMQ---------------SKWRELLSTKAFRDNLLGIVVDEV
HLI--YKWGKADKGHKAFRECFAKLGELRSIV--RRGTPILALTATAD----------LD
SRAIIQKQLHFE----DATQVTV--SPNRKNIRFGLI------------------TVSSH
TF--DCLDWIVRELKEHDTNMFQVIIYCRTLTAVGRVFCYLKAELGEDCW---------A
DKDPQRRAENLRIGM-FHSHTLPQNK-RRILDSF----GGDGP-CR----VVVATGALGM
GLNFPKISHVVMYGVPED-MEAILLEAGKAG-RDGSLSHAVIY--------RLSLNT---
--------NVDEGL---------------RTLLKNSSKS---------------------
CFRK---ALFSQFENNTDSVVPGHL--------CCTYCHSVC-------------SCTSA
GCDMAVPKYEQAP----------------------------LE-VSAPGQIREVTE----
--------------DEKDLIQSLLHTYQLSLNPGSRLYTNSS------------------
YCTGF---
---SD--E
LINAVL----EHCAEIFDLNFIMNNLPVFSKRHAQEIL----------------RILFEV
FEDF---------KFTE----------------LDFPPEDFVV-----------------
----------PDPN----------YMGYFDEDTEGNVLSQ--------------------
---------------------------CSSLESGLSALSTDSQNY---------------

>A0A1A8JIH7

--MAAALEQRSFEAAL----

-----SATLDDLDL--

---GF-------------
---------------------SLKHEQIEALKSFIFK--KDVFAA---LPT--GYGKSLI
--------YQLAPVV-------------------AKRMGLSENPLVVIVSPLIALMEDQV
KEASK------FGL-----------------TAMQLG--------------VHTLKDIRK
ANC----QLIFGSPE-AWLMQ---------------SKWRELLSTKAFRDNLLGIVVDEV
HLI--YKWGKADKGHKDFRECFAKLGELRSIV--RRGTPILALTATAD----------LD
SRAIIQKQLHFE----DATQVTV--SPNRKNIRFGLI------------------KVSSH
TF--DCLDWIVRELKEHDTNMFQVIIYCRTLTAVGRVFCYLKAELGEDCW---------A
DKDPQRRAENLRIGM-FHSHTLPQNK-RRILDSF----GGDGP-CR----VVVATSALGM
GLNFPKISHVVMYGVPED-MEAILLEAGKAG-RDGSLSHAVIY--------RLNLNT---
--------NVDEGL---------------RTLLKNSSKS---------------------
CFRK---ALFSQFENNTDSVVPGHL--------CCTYCHSVC-------------SCTSA
GCDMAVPKYEQAP----------------------------LE-VSAPVQIREVTE----
--------------DEKDLIQSLLHTYQLSLNPESRLYTNSS------------------
YCTGF---
---SD--E
LINAVL----EHCAEIFDLNFIMNNLPVFSKRHAQEIL----------------RILFEV
FEDF---------KFTE----------------LDFPPEDFVV-----------------
----------PDPN----------YMGYFDEDTEGTGLS---------------------

>A0A1A8K124


```
------------------------------------------------------------
------------------------------------------------------------
------------------------------------------------------------
------------------------------------------------------------
------------------------------------------------------------
------------------------------------------------------------
------------------------------------------------------------
------------------------------------------------------------
------------------------------------------------------------
------------------------------------------------------------
------------------------------------------------------------
------------------------------------------------------------
------------------------------------------------------------
------------------------------------------------------------
------------------------------------------------------------
------------------------------------------------------------
------------------------------------------------------------
-------------L----------------------------------------------
------------------------------------------------------------
---------------------------------------------GF-------------
---------------------SLKHEQIEALKSFIFK--KDVFAA---LPT--GYGKSLI
--------YQLAPVV-------------------AKRMGLSENPLVVIVSPLIALMEDQV
KEASK------FGL-----------------TAMQLG--------------VHTLKDIRK
ANC----QLIFGSPE-AWLMQ---------------SKWRELLSTKAFRDNLLGIVVDEV
HLI--YKWGKADKGHKDFRECFAKLGELRSIV--RRGTPILALTATAD----------LD
SRAIIQKQLHFE----DATQVTV--SPNRKNIRFGLI------------------KVSSH
TF--DCLDWIVRELKEHDTNMFQVIIYCRTLTAVGRVFCYLKAELGEDCW---------A
DKDPQRRAENLRIGM-FHSHTLPQNK-RRILDSF----GGDGP-CR----VVVATGALGM
GLNFPKISHVVMYGVPED-MEAILLEAGKAG-RDGSLSHAVI------------------
------------------------------------------------------------
------------------------------------------------------------
------------------------------------------------------------
------------------------------------------------------------
------------------------------------------------------------
------------------------------------------------------------
------------------------------------------------------------
------------------------------------------------------------
------------------------------------------------------------
------------------------------------------------------------
------------------------------------------------------------
------------------------------------------------------------
------------------------------------------------------------
------------------------------------------------------------
------------------------------------------------------------
------------------------------------------------------------
------------------------------------------------------------
------------------------------------------------------------
```


>A0A2D3IQF9

------------------MSTSKA------------------------------------

--P---

--LAD---------------
---------------------IGTPLL---------------------------------

---PPPFLNW------------

--FQAKGWSP------------

-----------------------RTHQLDLLRQAQQG--NSTLLI---APT--GAGKTLA
--------GFLPALVDLASRGKSN----------AT----SRGVHTLYISPLKALAVDIR
RNLEKPIDEMGLDI-----------------SLETRTGD------------TPAYKRQRQ
KLA--PPDILLTTPE-QLAL------------------LISAKDAGRFFEDLRYVIFDEL
HSLVTSKRG------HLLSLGLARLRRLQ-----PG-VQTIGLSATVADPDALRRWLLPQ
TGNSAMASLVTVEGGAKPHITIL--ETDDH-IPWA-----GH-------------SAVYA
LP--GIYDAIKRH--------GTTLIFV-----NTRSQA--------ERI---------F
HDLWMANDDNLPIAL-HHGSLDVSKR-RKVEQA-----MAANA-LR----AVVATSTLDL
GIDWGDVDLVIHVGAPKG-ASRLAQRIGRSNHRMDEPSRAILVPANRFEVMECQAALDAN
YIGAQDTPPLGEGSLDVLAQHVLGMACAEPFRADDLYEE--VTTALPYAA----------
VPRETFDRIVDFVAT--GGYALKSYERFAKIRQTVDGTWRVTHPRFAQQYRLNVGTIIEA
PSLNVRLTRQRGK-----GANARGGPVLGKVEEYFVEGLSPGDTFFFSGRVLRFEG----
-IRENECIVSNAEGFDAKIPAYAGGKFPLTTYLADQVRSML--GDPA--RWGALPDQVSD
WLRIQ----K-------------D---RSVLPQPGSLLVETFPRGERSYMVTYPFEGRLA
H--QTLGM
LLTRRLERAK--ARPLGFIATD-YALTIWGHRDMGRMF---KNGELPLAELFNEDMLGDD
LEAWLAESWMLKRTFRN----------------CALISGLVEKRHPGQEKSGRQVTVSTD
LIYDV-----LREHEPD----------HILIQATRADAAT--GLLDIRRLADMLSRIKGH
IVHKDLD-----------------RISPLAVPVMMEIGKERVPGE---------------
---------------------AHERL----------------------------------

```
------------------------------------------------------------
------------------------------------------------------------
------------------------------------------------------------
------------------------------------------------------------
------------------------------------------------------------
----------------------------------------------------LEEIADE-
------------------------------------------------------------
---------LTREMMDHR---------------------
>A0A1C7M4C7
------------------------------------------------------------
------------------------------------------------------------
------------------------------------------------------------
------------------------------------------------------------
------------------------------------------------------------
------------------------------------------------------------
------------------------------------------------------------
------------------------------------------------------------
------------------------------------------------------------
------------------------------------------------------------
------------------------------------------------------------
------------------------------------------------------------
------------------------------------------------------------
------------------------------------------------------------
------------------------------------------------------------
------------------------------------------------------------
------------------------------------------------------------
------------------------------------------------------------
------------------------------------------------------------
------------------------------------------------------------
------------------------------------------------------------
------------------------------------------------------------
------------------------------------------------------------
----------------------------------MLG--ADVLVV---APT--GMGKSLC
--------FQVPAIADDFSDAARSHRRDISPSGRVISLLLVHGF-LTEILANTALMKNQV
TKLRK------MHI-----------------PVASLTSE---TTSENK---NEIIEDLHS
ENP--EIRLLYVSPE-RFCKS---------------E-FNQIISAIYDQGQLNRLVVDEA
HCI--SEWG------HDFRQEYRRLGSFRDKF--PT-IPIMALTATAT----------NV
VQEDILRSLKIS----GEHLFKVIHPFNRDNLFYEVRYLSSS-------------NPAAH
IV--DIYEYISNL-HRRRKRPSSGIVYC-----RTRATC--------DDL---------S
NFL---RGKGLNARP-YHRGVKTTIL-NKTMEEWEQGGTGIDG-VD----IVCATIAFGM
GIDKADVRYIIHYDLPKS-FEGYYQETGRAG-RDGSPAKCILF-YSREDVVRVRRWV---
-SDSHSKRIIRAESDDGPVPS--------QRAMDSLTAL--INFAESVDV----------
CRHV---SICRYFGEDIDTDDPDILKRYCNG--MCDVC--KY----PDKTKGR-------
-----------------------------------KLELS-SE-ELVGSQTAKLQR----
--QVCHDDHDDG--HSKRSGSALGG--------DSNQYGHAKKAKPSPPAPLGMSAKLKQ
TLNKP----F-------------R---TPFKSPIIQPLLQEQRQD--------PFVSNSN
E------------------------------------------------------SSNID
```

```
KNEDAVIEISSDEED-GDVPTV---------VEVEKENLMHDFSPSPDVWLP---ETDVG
LDASFSQKIPISLRNDT----------------FTLIRRTLHKVFPHDHTGDAIWGRINK
AHSDS-----DTRTHVVAMVARELEFSALSLCTTHEGYKA--RSADKVQAVRLLAKAGAW
DKKLQKG---------------DEDFLDAEEVLDLMKRVCASHRK---------------
---------------------NKGRR----------------------------------
------------------------------------------------------------
------------------------------------------------------------
------------------------------------------------------------
------------------------------------------------------------
------------------------------------------------------------
------------------------------------------------------------
------------------------------------------------------------
------------------------------------------------------------
---------------------------------------
>A0A2C5X7S0
-----------------MTKNNLAHELKWLRQNPDIVRPNLQTQFVSP------------
------------------------------------------------------------
--VPSDFSQPAQSQTS--ILRSRL--------EGSQ------------------------
---------SQQRGALAIPPSQAPAPPPHLQARPVAKSSGDSTLTSLTTDAAASDKTNGD
DMVTMHNLPTRTSPMKLPQYSPFLSESTTPSLPHAKNDATCSKDKALHQPSTSNMGRFER
AYASQAASSNT-AGVDLKTPAQKSLYVQPPDSQTKPQQPNMTEEDLSLI-ELEDDYIPSS
D--DLGVDDEELQLWNEAHDPQSRPVSPIHKKRKSSDISTLK--------GPRIEAVKDD
SGDE-YPDVYEILGTEPPPSTRKTPSSRVAKQNSSQSSLESTST-----------RSGKR
SKLLLCQQEAYLHTPTKSKL-------------DVHTLKTPGKTKRLM-----------T
SSPSRKRIAT--------PDRISPKKRLSEERDLGPATTPQSSQREHENEHVV---QDSE
DECMTPP----SNSGSPL---------------TIPSTIAKPAAPSAPPPPPPPPPLFND
TSQDDSIMEISNPTVVPLTAHPPASSFIPQPPAP--------------------------
----------------------VIDNTPQ-------------------------------
------------------------------------------LPEATEANSVGSLI----
---------------------IKNPILL--SLK----------------------EKAIA
--AEVQRNQKDFEMALRVRK-------AGAKETRDRVKAEGVTLAGHLKEIQSLQAILGR
HKAMSDDMEYLLNQITKAYSQGISTDEDEN-----------RLDEITENIKAIET-----
-------------EFCQALAKSIVT---------------------------------DT
EVYELNAQYLRDTIVASTIPPESEIKDQKFAGAGGPKDMASVYETTTQAAAENPAV----
----------TPAQRSRSGLNSLLQT----SNDRKSSSG---------VICETPSNRRQT
GHSSPGLELDDIDDSILMELDVVDS---------SSIFHHGQRKNTT-------------
--ASLNTPSNIQPNR---QTPASRSYHTPC---NGPKSGIGPSFSDCEDDDFDDDLIALA
DSFEASSLPANQRTAE-ATLDNNASTKSAPKKTPSK-----PEKRKLPTSVKE-NPWPPE
QMCYPWSADVKQILKDRFRMQNFRHNQLQAINATLAG--KDAFVL---MPT--GGGKSLC
--------YQLPA---------------------VIRTGKTKGI-TLVISPLLSLMHDQV
NHLKN------LGI-----------------HAVMYNGE---MTKDAK---DMVMNMFQE
RNPEGYVEILYTTPE-MLNKS---------------DRFLKAMSSLYQRNKLARIVIDEA
HCV--SQWG------HDFRPDYKEIGKTRLAF--PR-VPVIALTATAT----------SN
VIIDIKHNLRLQ----NCETFAQ--SFNRTNLYYEVR---RK-------------SPKTI
FD--DIREIIEE--HRG----KSGIVYT-----LSKNTA--------ETV---------A
QKL---RDAGIMAER-YHAGVEPHEK-VQVQKNW-----QSGK-TQ----VVVATIAFGM
GIDKPDVRFVIHYAIPKS-LEGYYQETGRAG-RDGKPSKCYCF-YGKADAVLLRKMI---
```

```
------RGDFDDGG--RGRTQITTSKEQIDRQIAMLNRV--VTFCDNESD----------
CRRV---EILRYFNE--S-FDAADCNR------QCDNCLYDK----NVSEPLDFTHIAAA
ALGAVRICPE-----------------------SLTMGQC-AD-ILMGKGDKHMEKLNKK
FELEPFSNAAKG--LKKLDVERIIDRLSVENALMSGHKMNHRVGFAV--DYLKCGPEANN
FINGRKKLMLSINLGADKPKLKAKGKKKEPTSTMVSSPVRKGKGK--------EVIINVS
DESFFESETEPEKPHSKRGMLETDEGDDDAAFDPPGPSTSRAHRPTAQQQSLDPIHE--D
VIANFVDEAQRLEEGLRNTSGL--RKALFSQNDFREMA--TRWTTTLNAMSKIPGIDKTK
VRSHGDKFLPMLRMFREQYETMMDGDGFNEEDIVDLISDEECD--DGGNEDSKFFQSASR
NGS-------GNGNPGFASALPSVATASSTTGPSAPRTKS--SVEAWHDKLNSLTYNPGE
GGSGSRGGNGSSAYSRMGSSSSRGGHKRSFKGRSKSSWGSGGASA-----------SGSS
---------------------ARGWK----------------------------------
------------------------------------------------------------
------------------------------------------------------------
------------------------------------------------------------
------------------------------------------------------------
------------------------------------------------------------
------------------------------------------------------------
----------------------------------------------------KGYGGVS-
--------------------------------------------KSRGGG----------
---------SRKSSTKASGASSGGNVGGGSGGGIRPMPL
>D3BIJ9
------------------------------MTSNKVINNNYQDQL---------------
------------------------------------------------------------
--------KLYNNTLVDFDNNSNN--------NNSN------------------------
---------NRNNNDTNVVDDFISLKDINNHNINNKTLVTSLGAFKDIFKLIPKKQPNYS
NNYSSNIKFNASTNNNI----ASISSAPAASSSSTSSTYSPN-----NNSTSPGIKPI--
--TLKLQPKSK------TSTSTTNTNTNTNSTSNMSTNNNNNNNNNNNNDNGYDDFLRDF
D--GDNTSKVSSSK-----------VSDNSATRSSAYNNNNN--------NDRYVDLSAT
PKST------------PAPSRNVSSTPLMPLKLSSTTTSSLASS---------------K
STTVTNFNTRNSFTTSPRTM-------------TTDDLNASFDDSN-------------N
NNNDDYNDNG--------------------------------IDFENDMDFKR---QSTS
SSSS-------SYLSST----------------RVSAYNNNNNNNNNNNDNNNNNSNSNG
SESIEIQILRVKA--EFCDISMEKTKVKESKPVT--------------------------
----------------------DSERLAR-------------------------------
-------------------------------------------YDKLDDLKKKKKA----
---------------------ITKRLAELEQTR---------------------------
--DSQAKQRQDITSTTTATSTTN----YSTSST---------------------------
------YNNNNNNNYSSTFNNNNNNNNNNY-----------TSSLSSSTYNNSNN-----
----------------------------------------------------------NN
NYSSSTYNNNNNNNNNNN--------------------NNYSASTNYSKPMDNDTS----
----------IDAHDVDDFEFGNTLD----DNNDYESNN--------------NNNDNSS
MLFDDDYYDTGMTISAPSTTTTTST---------SSNSNNSNSNNHQ-------------
--QNLMNAEDAIDLDFDYNDFDLSTTTTTM---TSNNNN------------------NNN
NDSIFNSSVLNSSTSSMVSVAPPSISVQVDERHL-------AKWKAK-------FPW---
------SEKINSTNRNIFGNRSFRLNQEEIINASMAG--HDIFVL---MPT--GGGKSLC
--------YQIPA---------------------LL----ADGL-TIIISPLISLIQDQV
MLLQN------LSY-----------------PTAALTGS---ISSEEQ---TRIFKELRS
```

DNP--TIKLLYLTPE-KVVQS---------------TSIISLFHQLNSRGKLIRAVIDEA
HCV--SQWG------HDFRPDYKQLAILKREF--PK-LPILALTATAT----------ER
VKQDVIFNLSMK----DSITFKQ--SFNRPNLIYAVV----K-------------KKKSI
ID--DIIEFITANGYKQ----KSGIIYC-----FSTFEC--------EKV---------A
QEL---NSKGLKVKF-YHAKMTPEDR-QRTQENW-----TRDR-VK----IIVSTIAFGM
GINKPDVRFVIHHSLPKS-LEGYYQESGRAG-RDSQTAHCILY-YSYADKYRIDSLL---
--------ESSSGQGSTYQSI--------RENKDNLNKM--VSYCENSVD----------
CRRQ---LQLQYLGE--Q-FDRTICAK------TCDNC--KC----DDPIITK--DLTED
AHNLYKIFQSVDE----------------------PLGTI-LE-IFKGSQAKKITE----
KHYQNHKQHGSGKNVDKNDVDRLVHKMVTLNILKE-VHVANA-YRTI--TQTKSVNNTTF
FSSGR----R-----------------IKIECRAKSSGSSKDKGQSVISNI--SVNGNKN
K--GD--D
GPEKLRLYLIDVRAQIAEDAGL-PPHMIIQNSAFDDLV--EKRPTTLAELRNCSYFTNTR
VEKYGEQFIQAVKDFNN----------------GTMKKIATV------------------
----------LDDDDEF--------------QPTGSQDDG--ASPYFQKKSKRNTIKE--

--LLTQQHP-
--TP--------------
--------------TNIKYNLL-----------------
>F4Q985

--MMIT-AKSSSSTLW----------------KSLL------------------------

----------------------------YQSFTSMNLNSHHSLQPFTNYASSSSDG-IDY
EWSTTFKKKKGLSL----TKDKSVLTGKERLDKKDTNSLGFL------------------
NKYG-------------LISQGINAKDLLSSSSSDQNDPSKSSS----------------

--------------------------------IQEEENIIMLNEDGEMIE--P---PKKK
KSSS-----------------------------KKKDIIKQEEEEEEEEDGRR---VELF
EKPKKIRKKKMDL--------------EQDQLKV--------------------------
----------------------DEDGIQR-------------------------------
--VMDDTEVKKQRKSK----
-----------------------------KSDH----------------------QMMLL
--DFADITP---
---PPSLFDPQIYTTPL-----

-----------------------------------DLSDMVQDPTLASTSIHDEVE----
----------TSSSYLNSVNTPKKTR-----KKKLTTII---------------------

-ESMPNYNQDSQDS---NNNNLQQQ---------QQQSISILNQELL-------------
--RSYNQWVIEKK----ELLQQSTTKQRKI---------------------------SLP
NIPTGKKYEVTHSGG----------------HTEDN-----TLFSQE-------FPW---
------DDLVNSCAKTVFGVGELRALQKDAINAILYR--RDTFVS---LPT--GGGKSLC
--------FQLPA---------------------LI----DAGL-TVVVSPLLALMNDQV
TKLRQ------RGI-----------------PAAVLNSG---ISVSER---TRTMEELEN
PQG--SIKLLYVTPE-RLVSE---------------D-FAKRMARWHYQGRLRRLVIDEA
HCI--SEWG------HDFRSDYRKLSSFRKTF--PH-VPIVALTATAT----------DY
VEHDIKNQLGIS----RDDVVNVRGTFQRNNLRYAVRD-KPA-------------SPIGV
AM--DIESFIKAR-YPT----SSGIVYC-----ATSVEC--------ENL---------A
SHL---RDVGLSAHH-YYASLSTPTR-LEIQDNW----IK-GK-IK----VICTTTAFGM
GIDKPDTRFVIHHSMPQS-IESYYQHTGRAG-RDGQLSDSILY-FSDIDKKRMEKLMKPT
TSTQDQYENISNDK--VGDIY--------ERKLAALDSL--VSLLKD-TG----------
CRHQ---KLTNYFGD--TKNKPTSCGR------MCDNC----------------------
---ID-KYSGKPSKKQKN----
-----------------DHTERIYTK----------------------------------
---------------------------YDYSPTKKKLSSKK-------------------

>Q9VGI8
--------------------------------MSKKPVAQRKQL----------------

--TLSSFIGLDGNSQSQPKSRAAS------------------------------------
---VRS
KPPAVYNPIFLDASSSDDETTEISSQSNNGTIATKKSSRDPRTAKLKKHTYLDLSVSP--
--LAKLSAKKYARDSPKKPTSLDLSVSPLAELLAKKSDRDSPKKPVQNENSYTYRGLSES
PVENKSIGDTLRKPPQKERKTSIVWLSDSPEKKVTQNERKIL-----------DSPLQRF
SFEDFPNKENGNRHHLLTLPDSPPPPQPVKKPEKTMWQNETKTI----------------
------QDKDSPANPL--------------VSNNLASISTLLDSSRAPNTYKGSSRNLFE
DSPEKSGSGE--------------------------QGYKLGSAKENEIPTKPATASLER
NSVT-------------------------------SSPSPAAPLKPRYSVAFDNSLADYL
KDLAQNDNFSIDPNKQNTETLKSTLGFFRNTYVE--------------------------
----------------------LMEKYCS-------------------------------
--LIDQIPAMHFNEIA----

-------------------GFQPNTFLKLKVMR----------------------QKFKA
RTQLVQNSLDKKESQLKAEQEALEKEEIEMQAEQAQQTVLSSSSPEKS------------
------RPIMPLPKVQEIKDEKIPNRNQLIHDLCGEPDNFSPPSSPRDTQLIPKR-----
--QQ
LINDLCGEPDDFSPPSKQNDPHLLRKCEELVHDLCEEPDDYLAQSMMLDGDLEE------
----------EQLNGPTQGTTTSGMD----DDEDDLEGL------------LAEIEDEHQ
KMQGRRSEFNGYSYKELEAVKVK-----------EKHKETPINISLD-------------
----DDGFPEYDEAMFEQMHSQAAANKSRV---------------------------SSA
GPSTSKSVVPTKQTSA-----LHSQKLSGNFHANVHNDGITGEFDGQ-----K-FEH---
------STRLMHGLSYSFGLKSFRPNQLQVINATLLG--NDCFVL---MPT--GGGKSLC
--------YQLPA---------------------IL----TEGV-TIVISPLKSLIFDQI
NKLAS------LDI------------------CAKSLGE---QKMADV---MAIYRDLES
QPP--MVKLLYVTPE-KISSS---------------ARFQDTLDTLNSNNYISRFVIDEA
HCV--SQWG------HDFRPDYKKLGVLKKRF--PN-VPTIALTATAT----------PR
VRLDILAQLNLK----NCKWFLS--SFNRSNLRYRVL---PK-------------KGVST
LD--DISRYIRSK-PQH----FSGIIYC-----LSRKEC--------DET---------S
KKM---CKDGVRAVS-YHAGLTDTDR-ESRQKDW----LT-GK-MR----VICATVAFGM
GIDKPDVRFVLHYSLPKS-IEGYYQEAGRAG-RDGDVADCILY-YNYSDMLRIKKML---
--------DSDKAL--QYNVK--------KIHVDNLYRI--VGYCENLTD----------
CRRA---QQLDYFGE---HFTSEQCLENRET--ACDNC--IN----KRAYKAV--DALEH
ARKAARAVKDLCS-----------------GRSRFTLLHI-AD-VLKGSKIKKIID----
FNHHKTPHHGVLKDWDKNDVHRLLRKMVIDGFLREDLIFTNDFPQ----AYLYLGNNISK
LMEGT--------------------PNFEFAVTKNAKEAKAAVGSVSDGATSSTADGQSG
M--RE---
IHERCYTDLLDLCRTIASQRNV-TMASIMNIQALKSMA--ETLPITEKDMCSIPHVTKAN
FDKYGAKLLEITSNYASEKLLM-----------QAVLDEEEEQAAAKQRPSTSGW---NN
ESVDWDMAVASQGNANT-------SGASGFNSFRAGKRKKIYKSGASKRYKTSTTSPAAR
KTTSARG--------RGGRAGAKRAESSASSASGWKSKKTGNSFG---------------

--FDLMPLP-

---------GSK---------------------------
>W8BFP7
-------------------------------MNHNRTNLTYKKQL---------------

--SLSSFLEGKKECSS--
---------SNSDEASNTLSKKLLCEESVRNDKNIKSLAFAPLAK--------QQSIKML
NPIFLDSSSDEEKDEEQCSPNRNSRNSIGKAIVQRYTFDECS------------------
-----------------QSPLAPLESKENIKLVNRTINTKACNESSSLINQKDRYLEKNN
A--HGKVARNKYLPSQRDTKSEEERSKFYNSDIDSPKTSTSF------------------
-----------EEQQKQDCSSKYIAITKVSTSSKVPTPEKQKTP---------------D

```
SRKHFEWDGYEKMIEKKLST---------------------------------------D
YDEYFSQFEDSHQMSVTKTKPISSESPIKSINAYETVSPLKNQSKEYDNSSII-------
-----------------------------------KQCTGNKDATSSLKVTFDSKLTDYL
SAVEQDSTFKISIKDATDLELKTSNTFFKSTYNE--------------------------
----------------------LLEKLVQ-------------------------------
------------------------------------------IFDQVPVSFFSGIE----
-------------------GFDTKCYLRLKTMR----------------------NKLRA
RSKLVQTELEKREKKTRIIKDDPCDLDYDALEAEEMLMREECKNQDSE------------
-------------KITRTPENI-------------------WSNSLMSRISSDET-----
------------------------------------------------------------
GKSSSKTPDLSENILTVD-----------------DTDDEEMEQAYLTQAIALDNE----
----------ELDSLPKEKLNANNNKKLEFEDEDDLEAL------------IADIKDQED
LLRGKKSEYDNYSY---RDFEEETT---------PKQQMITKTTIAAAAKVSPDDLQQWS
TQTDDDGWQVYDPEKFEIAASQAILLDSPVSKSSVPNKKEVFSAMNITISEDENDNMPST
SAASLRKLNAPITITSSPSPLKTAQKVDGNFHANTHNDGITGEFDGF-----K-YPH---
------SQNVKEALSSNFGLKSFRPNQLQVINAALLG--HDCFVL---MPT--GGGKSLC
--------YQLPA---------------------LL----TEGV-TLVISPLKSLILDQV
NKLES------LDI-----------------YAKSLSGE---QTLQEI---RAIFCDLKT
SPP--RIKILYVTPE-KISSS---------------PQFQDLLDDLYASNYISRFVIDEA
HCV--SQWG------HDFRPDYKRLGILRKRF--PK-VPTMALTATAT----------PR
VRHDILRQLNLT----HTKWFLS--SFNRSNLKYSVL---PK-------------RGVST
LE--DMKAFINAR-PSN----WSGIVYC-----LSRKEC--------EEV---------A
NKL---HMAGIKSLA-YHAGLKDSLR-ETRQKDW----LT-GK-VR----VICATVAFGM
GIDKPDVRFVLHYSLPKA-IEGYYQEAGRAG-RDGEMAHCILY-YNYSDMMRMKKLL---
--------DLDNTV--AMDVK--------KIHLENLNRI--VGYCENVTD----------
CRRA---QQLDYFGE---HFTSEQCLERRET--ACDNC--LK----KQSYQEI--NVLEQ
SRKVARAVKDICG-----------------GRARFTLLYI-AD-VLKGSLIKRIVE----
SGHDKTPHHGAFKEWDKTDIQRLMRKMVLEGYLREELIFSND----IPQSYIYLGPKIQT
LMQES--------------------VTLKFAISRKESSKRALPGFSSGIVVSTSKTTSAG
S------------------------------------------------------TQLRS
IYERCYADLLDLCRTIAAARSV-TMASIMNMQALKAMS--EALPESETDMLSIPHVTKAN
FDKYGGKMLEITRNYAAEKL-------------CLLMDLEDAHLLSQKANSEKAAKTNSF
ASCDS-----DEDGDSWAT-----AAVSQGSKQNGGKRKRAWRGGGAKRTKKATGWASSG
KSPAKRTYTKARSGSTTSPKPGRRKAVGSISSRGRGGGKSGNNAW---------------
----------------ICKKSSSTGA----------------------------------
------------------------------------------------------------
------------------------------------------------------------
------------------------------------------------------------
------------------------------------------------------------
------------------------------------------------------------
------------------------------------------------------------
----------------------------------------------------FELMPLP-
------------------------------------------------------------
---------KSK---------------------------
>A0A034W9E7
------------------------------------------------------------
------------------------------------------------------------
```

```
----MSQTKSNKSSVFKKQLSLSS------------------------------------
-----------------------------------------------------FLDGNTT
RSGSINSDTGQISTEQKNTIIEGSLQSFRGVRNVDINNKSCK------------------
----------------------NLAYAPVRKQQTTKMLNPIFVDSSSEDENDDESHTNCT
KINNAGVTRAIVHRYTFDDCSQSPIAPLENKKNTKTDNSIIK---------------PIS
NGYSIYSNKKEKYSDKIDEHKNIYQEKDYKNGSKECIDGQSNAI----------------
----------------------------NGQKKHGDGMSNGFDDGS--------------
------------------------------------MYEVRSNHSSKESPPKKIISPSDT
KKTPLYREYAISG--------------------AIQKSKENETVARKPKITIDTALSDQL
NMIEQDSTFKICTKDATDLELKASSTFYKSSYYD--------------------------
----------------------LLEKFAQ-------------------------------
------------------------------------------IIDQVPVNYFDGID----
-------------------GFDKCIYLRLKTMR----------------------NKLKA
RSKLVQTELEKRQKQTRTLQSPALHLDYDALEAEEMQMREECKWQ---------------
-------------TLEKSPTNTPQSIYSNVS----------ITPSSSSVSKTPDY-----
------------------------------------------------------------
-----------------------------------HKNEAILTIDDTDEEDLQKTYL---
----------TQAAALDDEETEKSISQNSKPDEDELEAL------------LADIKDHDN
LLRGCKSNYNNYSY---KDFEDKAT---------PKQLTNAKAVAAPAAEISPEDLQQWS
TQTDDDGWQVYDPEKFEIAASQAILLDSPVSKSSIPNKSSVVPITNITLSEDECDDMPST
SAASMRKLNTSIATTSNLSPVKPAQKIDGNFHADTHNDGITGEFDGT-----K-YTH---
------SQAVKEALSSNFGLKSFRPNQLQVINAALLG--HDCFVL---MPT--GGGKSLC
--------YQLPA---------------------LL----SEGV-TLVISPLKSLILDQV
NKLES------LDI-----------------YAKNLSGT---QTLQEV---RAIYCDLKT
SPP--RIKILYVTPE-KISSS---------------PQFQDLLDDLYAKNYISRFVIDEA
HCV--SQWG------HDFRPDYKRLGILRKRF--PK-VPTMALTATAT----------PR
VRIDILRQLNLT----HTKWFLS--SFNRSNLKYSVL---PK-------------RGAST
LD--EIKAFISAR-PSS----WCGIVYC-----LSRKEC--------EDV---------A
NKL---HASGIKSLA-YHAGLTDPMR-ETRQKDW----LT-GK-VR----VICATIAFGM
GIDKPDVRFVLHFSLPKS-IEGYYQEAGRAG-RDGDIAHCVLY-YNYSDMMRMKKLL---
--------DLDNTV--AFDVK--------KIHLDNLNRI--VGYCENVTD----------
CRRA---QQLDYFGE---HFTSEQCLERRET--ACDNC--LK----KNTYQEI--NVLEQ
SRKVARAVKEICS-----------------GRSRFTLLHI-AD-VLKGSLIKRIVE----
CGHDKTAHHAIFKDWDKSDIQRLLRKMVLEGYLREDLIFSNDIPQ----AYIYLGPKVQA
LMQET--------------------ITFNFAISRKESAKRPLPGISNGITVTKDSAAEAT
Q------------------------------------------------------LR--S
IYERCYSDLLDLCRTIAAARSV-TMASIMNMQALKAMS--EELPESEKDMLSIPHVTKAN
FEKYGGKMLEITRNYAAEKL-------------CLLMDLEDAHQQQQLQIANKNKTTKAD
TRPPTLVSDDFDEDDGTGWAAAAASQGSMNSSGRGGKRKRAWRGGGAKRAKKTTGWATSG
KSPAKRGVAKGGGGSSTTPKAARRKAVASTSIRGRGGASKNGNNA---------------
---------------WLCKKSSSNGV----------------------------------
------------------------------------------------------------
------------------------------------------------------------
------------------------------------------------------------
------------------------------------------------------------
------------------------------------------------------------
------------------------------------------------------------
```

```
----------------------------------------------------FELMPLP-
------------------------------------------------------------
---------KSK---------------------------
>A0A0K8U0V1
------------------------------------------------------------
------------------------------------------------------------
------------------------------------------------------------
------------------------------------------------------------
------------------------------------------------------------
------------------------------------------------------------
------------------------------------------------------------
------------------------------------------------------------
------------------------------------------------------------
------------------------------------------------------------
------------------------------------------------------------
------------------------------------------------------------
------------------------------------------------------------
-------------------------------------------MDQVPVNYFDGID----
-------------------GFDKCIYLRLKTMR----------------------NKLKA
RSKLVQTELEKRQKQIRTLQSPALDLDYDALEEEEMQMREECKWQ---------------
-------------NLEKSPTNTPQSIYSNVS----------ITPSSSNVSKTPDC-----
------------------------------------------------------------
-----------------------------------HKNDAILTIDDTDEEDLQKIYL---
----------TQAAALDDEATEKSISQNSKADEDELEAL------------LADIKDHDN
LLRGCKSNYNNYSY---KDFEDKAT---------PKQFTNAKAVALPSAEISPEDLQQWS
TQTDDDGWQVYDPEKFEIAASQAILLDSPVSKSSIPNKSSVVPITNITLSEDECDDMPST
SAASMRKLNTSIAITSNLSPVKPAQKIDGNFHADTHNDGITGEFDGT-----K-YAH---
------SQAVKEALSNNFGLKSFRPNQLQVINAALLG--HDCFVL---MPT--GGGKSLC
--------YQLPA---------------------LL----SEGV-TLVISPLKSLILDQV
NKLES------LDI-----------------YAKNLSGT---QTLQEV---RAIYCDLKT
SPP--RIKILYVTPE-KISSS---------------PQFQDLLDDLYAKNYISRFVIDEA
HCV--SQWG------HDFRPDYKRLGILRKRF--PK-VPTMALTATAT----------PR
VRIDILRQLNLT----HTKWFLS--SFNRSNLKYSVL---PK-------------RGAST
LD--DIKSFISAR-PSS----WCGIVYC-----LSRKEC--------EDV---------A
NKL---HASGIKSLA-YHAGLTDPMR-ETRQKDW----LT-GK-VR----VICATIAFGM
GIDKPDVRFVLHFSL---------------------------------------------
------------------------------------------------------------
------------------------------------------------------------
------------------------------------------------------------
------------------------------------------------------------
------------------------------------------------------------
------------------------------------------------------------
------------------------------------------------------------
------------------------------------------------------------
------------------------------------------------------------
------------------------------------------------------------
------------------------------------------------------------
```


>O18017

--------MIKNREIE--VAPPRR----TIQFGGYT------------------------

--FVE---PDL-
-----------NFKAPIF---------------SCCGSIRDPSCEER-------EEEYID
NGHDEEPPVEVNR---

--IQESTSFDEPVSSP----

---------------------------PRYRPSE--------------------------
---NPGPSSSSYEPGHY-----

--SFNEYQ------------
----------QFPSRPQKRLVDPPIV----DLDEEPPIVD--------------------
----LDDSFDNFHV--------------------GSTSEEVVSGDIA-------------
--PEEEEEEGHDSFDDFESVPAQPPSKNTL---ASLQKS------------------DSE
IALNQQ-------------------------RHDMH-----GRFRGFLQDDSEEFSDEVG
LLGADMNKELYDTLKSKFGFNQFRHRQKQCILSTLMG--HDTFVL---MPT--GAGKSLC
--------YQLPA---------------------VI----LPGV-TVVVSPLRSLIEDQK
MKMKE------LGI-----------------GCEALTAD---LGAPAQ---EKIYAELGS
GNP--SIKLLYVTPE-KISAS---------------GRLNSVFFDLHRRGLLARFVIDEA
HCV--SQWG------HDFRPDYTKLSSLREKYANPP-VPIIALTATAT----------PK
IVTDARDHLKMQ----NSKLFIS--SFVRDNLKYDLI---PK-------------AARSL
-I--NVVEKMKQL-YPG----KSGIVYC-----LSRKEC--------ETV---------Q
MML---TKAGLSAEV-YHAGLNDNLR-VSVQRSW----IA-NK-FD----VICATIAFGM
GIDKPDVRFVIHYSLPKS-IEGYYQETGRAG-RDGMPSYCLML-YSYHDSIRLRRMI---
-----------EEGNTTTGVR--------SMHLNNVLQV--VAYCENVSV----------
CRRK---MLVEHFGE--V-YDEQSCRN-SKT--PCDICERQR----KNAEAIRLFDVSTD
ALSILK-----CL----------------PRMQKATLKYI-SE-LYRGALIKKSQEQAMR
LGHTKLPFYSKGQGMSEQDALRFVRKLVIEGYIHERLYSVPN-QAAAVFAYAELTEAGRD
LANGK----KTAKVYLHIVTCERKRKNAGLIELSNMNIVSEAQA---------LKERHMV

```
K------------------------------------------------------HG--D
VFTRCLQDLTHLITAVAESSGLSGPYSIVSREGIEQIA--ALLPRTNSDLLRIDSMTQIK
VTKYGRLIMELLATY------------------WKQVDEREEE--E------------MR
NQLDK-----LKSGEIV-------MGGFATLQSD----------PGFPSVPYMKPLG-GG
GGCRGRG---------KKRAFSGFSSGRATKKPRATAPSARGKTS---------------
---------------------GRGGA----------------------------------
------------------------------------------------------------
------------------------------------------------------------
------------------------------------------------------------
------------------------------------------------------------
------------------------------------------------------------
------------------------------------------------------------
------------------------------------------------------------
--------------------------------------------KPAT------------
---------SLKRNMYPATSM------------------
>A0A0X3P9R3
------------------------------------------------------------
------------------------------------------------------------
--MSSDGRGSSSKRFI--FRPVKP------------------------------------
------------------------------------------------------------
---------------------PVASPAVTSTPNPPRTLFTPS-----PSVPTPKILPT--
---------------AKVIPLTSKLNRKSPSPELELTDVENSPDPKRTSG----------
---APQKCPMVFSP----TQPPTVRCTLATASTHTSAPNVSP--------SWIDDNPQGT
SFTN-------------GVISPLFTPQLTSTQSPSPRVQNSPKG----------------
------------------------------VSPVKMSLKSLLQAQ--------------N
QQKPIPSISG----------------------------------------------PPSV
PCSI-------SRTVIS----------------KAPQANRQLKLENMSTRSLT-------
--PKPPTKQQPKG--KKTTHIKPDGVCISKELFK--------------------------
----------------------LLEEVCD-------------------------------
------------------------------------------IIQELPLQRLLT------
---------------------C--------------------------------------
----FGSKVNTVSSLLA----ER----TKHREQ---------------------------
--------------TACIAYDTTPTSMDTV-----------FPEDQDSRLSDAST-----
------------------------------------------------------------
-----------------------------------TLDEPPPEPPESSAP----------
----------QAFSNDGGLGFATDEE----DQDNGAPLN---------------------
-------DWNEFDF---------------------ETQEAPP----Q-------------
--TAASGPPQVELIR--RPKPNEPKFEAD----DGEGNA------------------FLP
LTNNQSKWAPDFDTVD--CKDQP--------DDGST-----GEFDGT-----DKFPH---
------SKAMMDAFKRIFGLRTFRRNQLQAINATLLG--KDCFVI---MPT--GGGKSLC
--------YQLPA---------------------VV----QSGL-TLIVSPLKALILDQV
TKLQS------LGV-----------------RAASMSGD---VSQSEM---HRIYTLIHS
QPL--QLDLLFLTPE-KIAAS---------------EKLKSCLEHLYKRNLLARFVIDEA
HCV--SQWG------HDFRPDYRNLKILRVNF--PT-VPMMAMTATAT----------PR
VRQDILHQLLMK----DTKWFIQ--SFNRPNLKFEVR---PK-------------KLKNC
SK--EVIDLIRSQ-FSG----LAGIVYC-----LSRNEC--------DRL---------A
EEL---TAGGIVARA-YHAGMGDAAR-KRVQEGW----LQEER-FK----VVCATIAFGM
```

GIDKPDVRFVIHFSIPKS-IEGYYQEAGRAG-RDGLPATCVLY-YHWQDVIRLRKLI---
--------YSASTHPFNDPSV--------KLHEDALFKM--VSYCDNVVD----------
CRRA---LILAHFGE---AFNAADCSLVVGC--LCDNCQFAE----RRKLAQR--DLTQD
AKMIVEAVAEFVC-----------------RRRNVTLNYC-VE-IFRGAQTAQIQRN---
-NDSGNPLYGKGANYSKIDAERLIHRLLADRILLEEFVVTTV-DTVA--AYLRLGPKADS
LLSGQ----Q-------------K---IMLPVAVNLKPRGTEP----------VIAGEEP
V--DKVAN
IRNDCYEALVRTAKQLTTKQGISNYATVFPNEMLLEIA--DQLPTTPEELLRIPQCTEYK
LNRFNAT-----QAFLD----------------VTLNFLSILG--G--------------
------------------------KYSLPTS------------------------IF---
---CT---------------

--KTLLTDD-

-------------ILNEH---------------------
>A0A0X3P2Y7

--MSSDGRGSSSKRFI--FRPVKP------------------------------------

---------------------PVASPAVTSTPNPPRTLFTPS-----PSVPTPKILPT--
---------------AKVIPLTSKLNRKSPSPELELTDVENSPDPKRTSG----------
---APQKCPMVFSP----TQPPTVRCTLATASTHTSAPNVSP--------SWIDDNPQGT
SFTN-------------GVISPLFTPQLTSTQSPSPRVQNSPKG----------------
------------------------------VSPVKMSLKSLLQAQ--------------N
QQKPIPSISG--PPSV
PCSI-------SRTVIS----------------KAPQANRQLKLENMSTRSLT-------
--PKPPTKQQPKG--KKTTHIKPDGVCISKELFK--------------------------
----------------------LLEEVCD-------------------------------
--IIQELPLQRLLT------
---------------------C--------------------------------------
----FGSKVNTVSSLLA----ER----TKHREQ---------------------------
--------------TACIAYDTTPTSMDTV-----------FPEDQDSRLSDAST-----

-----------------------------------TLDEPPPEPPESSAP----------
----------QAFSNDGGLGFATDEE----DQDNGAPLN---------------------
-------DWNEFDF---------------------ETQEAPP----Q-------------
--TAASGPPQVELIR--RPKPNEPKFEAD----DGEGNA------------------FLP
LTNNQSKWAPDFDTVD--CKDQP--------DDGST-----GEFDGT-----DKFPH---
------SKAMMDAFKRIFGLRTFRRNQLQAINATLLG--KDCFVI---MPT--GGGKSLC
--------YQLPA---------------------VV----QSGL-TLIVSPLKALILDQV

TKLQS------LGV-----------------RAASMSGD---VSQSEM---HRIYTLIHS
QPL--QLDLLFLTPE-KIAAS---------------EKLKSCLEHLYKRNLLARFVIDEA
HCV--SQWG------HDFRPDYRNLKILRVNF--PT-VPMMAMTATAT----------PR
VRQDILHQLLMK----DTKWFIQ--SFNRPNLKFEVR---PK-------------KLKNC
SK--EVIDLIRSQ-FSG----LAGIVYC-----LSRNEC--------DRL---------A
EEL---TAGGIVARA-YHAGMGDAAR-KRVQEGW----LQEER-FK----VVCATIAFGM
GIDKPDVRFVIHFSIPKS-IEGYYQEAGRAG-RDGLPATCVLY-YHWQDVIRLRKLI---
--------YSASTHPFNDPSV--------KLHEDALFKM--VSYCDNVVD----------
CRRA---LILAHFGE---AFNAADCSLVVGC--LCDNCQFAE----RRKLAQR--DLTQD
AKMIVEAVAEFVC-----------------RRRNVTLNYC-VE-IFRGAQTAQIQRN---
-NDSGNPLYGKGANYSKIDAERLIHRLLADRILLEEFVVTTV-DTVA--AYLRLGPKADS
LLSGQ----Q-------------K---IMLPVAVNLKPRGTEP----------VIAGEEP
V--DKVAN
IRNDCYEALVRTAKQLTTKQGISNYATVFPNEMLLEIA--DQLPTTPEELLRIPQCTEYK
LNRFNAT-----QAFLD----------------VTLNFLSILG--ALKEEEKEALKHEEQ
LTMHY-----GPSTSHL-------KHGIPAQRPTVKSQYF--KKSRAGKAANRRKIF-GN
FAYKVKS---------GVRGGSAVGPTAGWKRKASAPSRPTASSS---------------

--SSTFTPK-

-------------LLKLSTRME-----------------
>A0A0X3NLA4

---------MVFSP----TQPPTVRCTLATASTHTSAPNVSP--------SWIDDNPQGT
SFTN-------------GVISPLFTPQLTSTQSPSPRVQNSPKG----------------
------------------------------VSPVKMSLKSLLQAQ--------------N
QQKPIPSISG--PPSV
PCSI-------SRTVIS----------------KAPQANRQLKLENMSTRSLT-------
--PKPPTKQQPKG--KKTTHIKPDGVCISKELFK--------------------------
----------------------LLEEVCD-------------------------------
--IIQELPLQRLLT------
---------------------C--------------------------------------
----FGSKVNTVSSLLA----ER----TKHREQ---------------------------
--------------TACIAYDTTPTSMDTV-----------FPEDQDSRLSDAST-----

-----------------------------------TLDEPPPEPPESSAP----------

----------QAFSNDGGLGFATDEE----DQDNGAPLN---------------------
-------DWNEFDF---------------------ETQEAPP----Q-------------
--TAASGPPQVELIR--RPKPNEPKFEAD----DGEGNA------------------FLP
LTNNQSKWAPDFDTVD--CKDQP--------DDGST-----GEFDGT-----DKFPH---
------SKAMMDAFKRIFGLRTFRRNQLQAINATLLG--KDCFVI---MPT--GGGKSLC
--------YQLPA---------------------VV----QSGL-TLIVSPLKALILDQV
TKLQS------LGV-----------------RAASMSGD---VSQSEM---HRIYTLIHS
QPL--QLDLLFLTPE-KIAAS---------------EKLKSCLEHLYKRNLLARFVIDEA
HCV--SQWG------HDFRPDYRNLKILRVNF--PT-VPMMAMTATAT----------PR
VRQDILHQLLMK----DTKWFIQ--SFNRPNLKFEVR---PK-------------KLKNC
SK--EVIDLIRSQ-FSG----LAGIVYC-----LSRNEC--------DRL---------A
EEL---TAGGIVARA-YHAGMGDAAR-KRVQEGW----LQEER-FK----VVCATIAFGM
GIDKPDVRFVIHFSIPKS-IEGYYQEAGRAG-RDGLPATCVLY-YHWQDVIRLRKLI---
--------YSASTHPFNDPSV--------KLHEDALFKM--VSYCDNVVD----------
CRRA---LILAHFGE---AFNAADCSLVVGC--LCDNCQFAE----RRKLAQR--DLTQD
AKMIVEAVAEFVC-----------------RRRNVTLNYC-VE-IFRGAQTAQIQRN---
-NDSGNPLYGKGANYSKIDAERLIHRLLADRILLEEFVVTTV-DTVA--AYLRLGPKADS
LLSGQ----Q-------------K---IMLPVAVNLKPRGTEP----------VIAGEEP
V--DKVAN
IRNDCYEALVRTAKQLTTKQGISNYATVFPNEMLLEIA--DQLPTTPEELLRIPQCTEYK
LNRFNAT-----QAFLD----------------VTLNFLSILG--ALKEEEKEALKHEEQ
LTMHY-----GPSTSHL-------KHGIPAQRPTVKSQYF--KKSRAGKAANRRKIF-GN
FAYKVKS---------GVRGGSAVGPTAGWKRKASAPSRPTASSS---------------

--SSTFTPK-

-------------LLKLSTRME-----------------
>A0A291S6U0

-------MPSSRGKLASVGGDLQI--------CGVM------------------------
---------FSARIPGKIEDPPES------------DIANGSTKL---------EETAMA
CDSLMREDSFDYGPDEAVEKRDSIISIHSTDSSFTSPQKARK-----NNSGQSEPIYI--
-----------------DDSLEDRPLEGSSKIDSEKENCTPPMEPSKPKPNDVSIS-DET
Q--TPQDSNKF-TPKKPSLKLKSLSLKLNSAKKDVKTEYPII-------------RPQPR
QVRP-------------RFLEDSSDEEDIPTSKKSPPKKSPTDS---------------P
RQLTPKKSQPRPHESSPRSP--------RWKGPDPKVNLTPLGLDQ-------------K
LTPWIASVKKNPAMTSTPTDASHLQDQKTSLQGLEIEI---LDKFYTAMEKIP---TQVL
EKFP-------QFDAKVFSELRILRQHVKAKSRRVSKQLESVDLNTKGKTPLNDSSQDIE
ASPMKPKAQKPVLEDDFPTEDELEPSPIKRPL----------------------------
----------------------ALS-----------------------------------

--RFENPKLSDTKTMR----
---------------------RNTAVVAQSSV-----------------------DDYPS
DDDMSSYFPSTSSENREACKLKD----TRVNGFVVKAESKRASPLTPV------------
-----------SPRFEPESDGTLTPEGTSR-----------RPGLDSPSPSPAPV-----
---KKS
GFQVKRPVKAQMAPQTKQ-----------------QLEALWEKRQPLKTPESPQTA----
----------PSPSKPERKFSTPAPR----EETPAKKPT------------IDIDEEIRA
YEMMKHMSVDLADV--STSF---------------QYPGDSPELPEG-------------
--KSKPERLSSEQVE--RFPSVAFAYEDK----NPPKSS-----------------QIS
TASSSRSDTSKKSAKDIFEMGDFMGQVH---NDGVT-----GDFDGL-----A-YSH---
------SPEMLKIFRKTFGLFDFRPNQLQAINAAMLN--HDCFIL---MPT--GGGKSLC
--------YQLPA---------------------LL----TRGI-TIVISPLKSLINDQV
QKLVS------LDV-----------------PAAHMLGG---ASEKHM---NGVYRELAL
ENP--SLKLLYVTPE-KIAAS---------------EKFMSILTRLYQLKLLSRFVIDEA
HCV--SQWG------HDFRPDYKKLKVLRTKY--PQ-VPVMALTATAT----------PR
VRTDILHQLGMT----NPKWFLS--SFNRPNLKYTIA---EK--------------KGKEG
IP--KIIALIKEK-YKN----ECGIVYC-----LSKKEC---------DDY---------A
DQM---RMNGIKAMS-YHAGLGDNKR-SEVQGRW----IAEE--IK----VVCATIAFGM
GIDKPNVRFVIHAVIPKS-IEGYYQESGRAG-RDLESSDCILF-YSYADVHRLRKMM---
--------QLDRP---PADVY--------ETHMENLYKM--VAFCENKTD----------
CRRS---LQLNYFGE---KFDRSKCGAVRGA--ICDNC--RN----TDTFTEI--DVTED
VKAIITAVRDISK-----------------TRGNITAIQL-GD-VFKGADLKKIRDS---
-GTNKLALYGRGKSWNKSDVERLIHKLVVDGLLEEEMVIK-N-DMTA--AYLRLTKHSGE
FMRSN----D-------------K---IMFPMRQSTKAQLTVTV---------SASTKPG
N--TDMKN
LQDECFAALMRDIKGYAGAIEV-SATSVMNPIAVRAMS--QQMPTTKEAMLQIPHVTESN
FEKIGKALLHILKDFAE----------------KKAVLEAIEA--AQEE---------EV
NDESD-----FEDGWS--------AATSSTTGSGGGRKRK--SFGAKGNAAKR---YKRN
TSGSGRG------------------RYSGKRGGRAARGKAQPKG----------------

--PGLVDFT-

---------QNKQYLADPLRYGSI---------------
>A0A2B4SZU5
---------------------MSSHDNKSYISPGNPPINNLAQQLGKLRRENLSSAANKT
STSALSSSKQKHKTQTKGECAEENGTEVPGSRQIARVTPFLSTSNCVFKTSGSGTLNSRS
AGEARRPVAPLKKELV--FQNPKHNANNEDDENDFQVTAEFPITKKRKLNGNSVFKNGKN
HMEKNNNQDYKKKSCSRKNSTNSSTGPVI-------DLTGTEGQNEGIDEQYWTRNREDA
DIGDCSDVADLLFSTDFQPTSYDASDIPPKNSVEDPGNLSSD-----GKPFAQSQGLMNS
FTPLESVSPVHSPPPQNDLPLTNAPSAPEDDLPMNSPPLPEYFPQVDSP-PPLEYV-QTM
D--SPSSQENLHPMESPPPPEYFPPMDSPQLQEDGPAVNSLPPPEYFPPVDSSQLQKDGP

AVNS-PPPVDSPQFPNVGLSVNSPPPPEYFPGVKSLSHQEDFPSMDSPPLPEFFSPRDSP
PPPEDVLPVNSPPPQETFPAVNSPPPPEYFSPPQAPKIPSAAETEFGSGIPCEDHANSSV
SPPPPEYFPG-VKSLSHQEDFPSMDS------PPLPEFFSPRDSPPPPEDVLPVNSPPPQ
ETFP-------AVNSPPPPEYFSPPQAPKIPSAAETEFGSGIPCEDHANSSVR-SNEISH
TLQQPVSQQIRSPETHGNTSQLDNLISKHNFLFEDSYEGLQGLEAHITIKKGAKPVFFKP
RKVPYALKDAVESELDKLEKNGVIKKVER-------------------------------
-------------------------------------SEWASPIVVVPKADKITKDIARE
TQHDPAMSKVLHFVMSGWPDRCEDETLKPYHNR----------------------RLELS
CEQSCILWGSRVIIPPSLRVKMLKELHWKH-PEHGLPEELVSDTGPQFVSHEFSEFMRKN
GIKHTLVPPYHPPSNGAAERTTGVTPAELMVKLKLRTRLSLVKPDLEQAVQHKQTKQAVY
KDPKVDAERFSQVYDRVRVRNTRPKSQTPSIKVQLHLEEMHSMHYQTLENICSLLDKADP
TIICSLPKSIKEAQNLRQSIRSKILQLENMLQQRGESKDCTFKICSRQSPRINNASLSNT
SFIPCGESFYEGYRSVSSSLSSRLNK----ENNFSTTSENFIRLYSGSTSLPDGGNDYIV
TKTQNSVSMNSASI--IDDVKKDSLLLTELLDSSDDEWANA--GILD-------------
--SNGQQKKSISPAT--ATHNSSASRQQPI---PTLSSK------------------YKP
GATQSNNLNSSFNSPNRINTIP---------NNGND-----DDLKRR-----D-LPY---
------HREMWKILKNVYNIRSLRTNQLEAINAAMMK--HDCFIL---MPT--GGGKSLC
--------FQLTA---------------------LM----GAGA-SFVISPLRSLIQDQV
QRLQS------MKL-----------------PAVHLLGD---AGSGSTRHLNSVYQDLSL
RNP--TIKLVYVTPE-KLSAS---------------DKLDSVMKSLYNRGLLDRIVIDEA
HCV--SQWG------HDFRPDYKKLSVLRNKF--PK-LPFMALTATAT----------PR
VQKDILHQLKMT----KPKWFTQ--TFNRPNLRFIVK---PK---------------KKV
MD--EILQYIKTK-QPA----SSGIVYC-----LSRNDC--------ERV---------A
DSL---NEAGIKAIA-YHAGLSDDQR-TQCQEAW----IN-NR-YQ----VVCATIAFGM
GIDKATVRYVIHHSMPKS-LEGYYQECGRAG-RDGQLAECILF-YLYGDTNRIKRMI---
--------QNNSDR--TADSK--------QVDMDNLFRV--VQYCENVSE----------
CRRV---QLLHYFGE--MSFDPSQCRARSDS--TCDTC--AT----SATFDNK--EITSD
AKAFVENVNSIV------HCGKRNWRKPVK---SFTLNHF-ID-IFKGSGGSRVTSE---
-GHDRCSLYGLGKSYHRNDAERLGHLLVLQRVLAEHIVIGNH-ENVI--SYVKLGPKGMD
FLQGR----V-------------KLPPLPVREKEKTVSKSKRK----------ELVDQTS
D--SN--Y
ISSDCATDTGD--NYDGSS---
--NR
SHYWQ-----QDEKAPV------------------GRKRK--RKPAGSTARPNSTSG-TS
TGTFASA----------QQTKKNLQNFRRNKSETSFNRKGTAANQ-----------RSG-

--LALMAPP-
--QP--------------
----------VKRFTR-----------------------
>F6THN7
--------------------------------MAALPQNNLQKQL---------------

--ELFS-AKGTANKLS--LQKPKS--------SVFT------------------------
---------FKKKCSPNGSLSTGFV-----------EVKQNVALK--------DKNVNVK
QNDVQTNLTVLPKSAER----NKINGFF--------------------TPVYTKSTQL--
--PQTVAVKDH-----VHINDSANKHPRVEDSASKKTGINTSFGSVTSL-EEWDDL-DDF
D--TSVSP----------PKSQAGKIKITPQNCKNTSPVSS---------KIQCLSPKGP
TIEK------------QMCAKKPYENNEIASEPQKLVHAQTAEM---------------P
DKSLVCLGNKSNNNKYICIK----------DSTENSSTYSDITAEK-------------D
TCRNIDYSGT--------DDIEHDQE--------TLSQALIENEDDCEPDFIP---PSPS
DDLT-------MISEHDSL--------------HCFSGSAEKDTCRN--IDYS-GTDDIE
HDQETLSQALI----ENEDDCEPDYSKVPSHLLS--------------------------
----------------------VMLEICD-------------------------------
--LVDKIPISELHVLS----
---------------------CGMDLKKKRDMRYVLSTKNIYWSVDLWNTELCWLSRGVK
--DNSVLRSSPADSSTV----SL----TSCPS----------------------------
---------QNMEFDVSAPKDSENFSGSSV-----------NKIFKFNKLVADEF-----
------------------GTKGNDR---------------------------------AS
DFAPNFREKMENKTSFSL-----------------QAGGDSVMEDSFSFQSNVLST----
----------SQFNTPQNEKPVSSST----CARPISQPID-----------DMDNPDL--
-----DFDIDNFDI---EDF---DD----------QLMDSPAAPPVS-------------
--SK--NAPQYPTIC--ETQFDSGSKEKVT--------R------------------HNV
GDKTNTELSEYQYYRN--SLLKPQI------VNPAL-----ERFKSF-----N-FPH---
------SKEMMKIFHKKFGLHRFRTNQLEAINACLLG--EDCFIL---MPT--GGGKSLC
--------YQLPG---------------------CV----SPGV-TIVISPLRSLIVDQV
QKLTS------LDI-----------------PATYLTGD---KTDAEA---ASIYLQLSK
KDP--IIKLLYVTPE-KVCAS---------------TRLISTMENLYERQLLARFVIDEA
HCV--SQWG------HDFRPDYKRLNMLRQKF--QS-VPMMALTATAN----------PR
VQKDILNQLKMT----KPQIFTM--SFNRDNLKYDVL---PK-------------KPKRV
AL--DCVEWIKKY-HPN----DSGIIYC-----LSRHEC--------DTM---------A
DTL---QKGGLAALA-YHAGLADSNR-DYVQHKW----INQDD-CQ----VICATIAFGM
GIDKPDVRYVIHASLPKS-VEGYYQESGRAG-RDGEISHCLLF-YSYSDVTRIRRLI---
--------QMEKDG--NSHTK--------QTHFNNLYSM--VHYCENVVE----------
CRRM---QLLAYFGE--NNFNPNFCKENTRV--SCDNC--LG----RKEFKSR--NVTDD
VRNIVRFVQENCS-------SARGKGRSNS--SRLTLNMM-VD-IFLGSKSAKIQT----
------GLFGKGAAYSRHNAERLFRKLVLDRILDEELYITAN-DQAV--AYVKTGERAQA
VLNGF----L-------------T---VEFQDTENASSLRKQKA---------SVVANTS
Q--RE--E
MVKKCQAELTELCKRLGKIFGV-HYFNIFNTATIRRIA--ESLSPEPDVLLQIDGVTEDK
LEKYGAELIDVLQKYSE----------------WTLPVEDIHQ--KSSGPANFSA---RR
YDSDD-----DDGERGE-------KSSYFSTKSNKGTKRK--NAPTFRKSKKRKTGG-DG
QQSHPKN---------ATGSFSRNNSTAKTSSSYYSGSKT--------------------
---------------------GADKR----------------------------------


```
------------------------------------------------------------
----------------------------------------------------PGFMAPP-
--------------------------------------------LPQ-------------
---------PNRRFLKPSYTLF-----------------
>Q9DEY9
--------------------------------MAALPQNNLQKQL---------------
------------------------------------------------------------
--ELFP-AKGTSNKLS--LQKTKS--------SVFT------------------------
---------FKKKCSPNVSASTGFI-----------PFQQHV-LK--------DKNVNVK
QDGTH---TALPKATER----NKINCFF--------------------TPVYTKSGQP--
--PQVVALKDH-----VHGNDSANKPPSTEDAASKKTGINTSFGSVTSL-EEWDDL-DDF
D--TSVSP----------PKSHAGKGGKTPQKCKNTSPVASF--------KIQSISPEGP
TTEK------------HDCAKLLYDNNEVASEPRKNLHAKTAES---------------P
DQSLVCLASVEPTNL-----------------------------ER-------------D
MCRNTDYLGT--------DDLEHDQE--------TLSQVLIEEEDDCEPDFIP---PSPS
DE---------SLSSPPVL--------------KVISAQRKHKVSSL--TD----VNDCE
NTTDHLQGQSV----STSLD-----SKVPSQLLT--------------------------
----------------------LMLEICD-------------------------------
------------------------------------------LVDKIPISELHVLS----
---------------------CGLDLKKKRDMR----------------------KRLLS
--NDSVFRSSPADSSTV----SL----TSCTSS---------------------------
--------TQNRDFNVNAPKGAESLSGSSV-----------SKVFKFNKLAVHDI-----
------------------GTKESEN---------------------------------SA
NSAPNFMEKIGNKTSFSF-----------------RAGGDSIMENSFNFHSSVLSN----
----------SRFNTPQNEKPISSST----CTRPYSQPID-----------DMDNPDL--
-----DFDIDNFDI---EDL---DD---------IHCLDSPAAPSVS-------------
--SK--NVPQYPTIR--EAQLDSRNKEKNT--------R------------------NNT
GDTTNPSL-----LSD--SLLKPQI------ENPAH-----ERFRGF-----N-FPH---
------SKEMMKIFHKKFGLHRFRTNQLEAINACLCG--EDCFIL---MPT--GGGKSLC
--------YQLPG---------------------CI----SPGV-TIVISPLRSLIVDQV
QKLTS------LDI-----------------PATYLTGD---KTDAEA---ASIYLQLSK
KDP--IIKLLYVTPE-KVCAS---------------TRLISTMENLYERQLLARFVIDEA
HCV--SQWG------HDFRPDYKRLNVLRQKF--QS-VPMMALTATAN----------PR
VKKDILNQLKMT----KPQIFTM--SFNRDNLKYEVL---PK-------------KPKRV
AL--DCVEWIKKH-HPN----DSGIIYC-----LSRHEC--------DTM---------A
DTL---QKEGLAALA-YHAGLADSNR-DYVQHKW----INQDD-CQ----VICATIAFGM
GIDKPDVRYVIHASLPKS-VEGYYQESGRAG-RDGETSHCLLF-YSYHDVTRIRRLI---
--------QMEKDG--NSHTK--------QTHFNNLYSM--VHYCENVVE----------
CRRM---QLLSYFGE--NNFNPNFCKEHTQV--ACDNC--LG----KKNYKSR--DVTDD
VGNIVRFVQDNCS-----LVQGRGKGRSNN--TRLTLNMM-VD-IFLGSKSAKIQT----
------GLFGKGAAYSRHNAERLFRKLVLDRIIDEELYITFN-DQAV--AYVKMGERAQA
VLNGF----L-------------K---VDFQDTESASSIRKQKA---------SVVTNTS
Q------------------------------------------------------RE--E
MVKKCQAELTELCKRLGKIFGV-HYFNIFNTATIRRIA--ESLSPEPEVLLQIDGVTEDK
LDKYGAELIDVLQKYSE----------------WTLPVEDICQ--KSGGPANVSA---RR
SNSDH------DDESCD-------KSSYFSSNNKKGPKRK--NSSYFGKSKKRKTGG-DG
QQSRSKN---------GNSSYARKNSTAKTSSSYISGSKT--------------------
```

---------------------GADKR----------------------------------

--PGFMAPP-
--MPQ-------------
---------PNRRFLKPSYSMF-----------------
>A0A1L8GSC3
--------------------------------MAALPQNNLQKQL---------------

--ELFS-AKGTSNKLS--LQKTKS--------SVFT------------------------
---------FKKKCSPNVSASTGFI-----------PFQQHV-LK--------DKNVNVK
QDGTH---TALPKATER----NKINCFF--------------------TPVYTKSGQP--
--PQVVALRDH-----VHGNDSANKPPSTEDAASKKTGINTSFGSVTSL-EEWDDL-DDF
D--TSVSP----------PKSHAGKSGKTPQKCKNTSPVTSF--------KIQTISPEGP
TTEK------------HDCAKLLYDNNEVASEPRKNLHAKTAES---------------P
DQSLVCLASVEPTNLG--------------------------SAER-------------D
MCRNTDYLGT--------DDLEHDQE--------TLSQVLIEEEDDCEPDFIP---PSPS
DE---------SLSSPPVL--------------KVISAQRKHKVSSL--TD----VNDCE
NTTDHLQGQSV----STSLD-----SKVPSQLYT--------------------------
----------------------LMLEICD-------------------------------
--LVDKIPISELHVLS----
---------------------CGLDLKKKRDMR----------------------KRLLS
--NDSVFRSSPADSSTV----SL----TSCTSS---------------------------
--------TQNRDFNVNAPKGAESLSGSSV-----------SKVFKFNKLAVHDI-----
------------------GTKESEN---------------------------------SA
NSAPNFMEKIGNKTSFSF-----------------RAGGDSIMENSFNFHSSVLSN----
----------SQFNTPQNEKPISSST----CTRPYSQPID-----------DMDNPDL--
-----DFDIDNFDI---EDL---DD---------IHCLDSPAAPSVS-------------
--SK--NVPQYPTIR--EAQIDSRNKEKNT--------R------------------NNT
GDTTNPSL-----LSD--SLLKPQI------ENPAH-----ERFRGF-----N-FPH---
------SKEMMKIFHKKFGLHRFRTNQLEAINACLCG--EDCFIL---MPT--GGGKSLC
--------YQLPG---------------------CI----SPGV-TIVISPLRSLIVDQV
QKLTS------LDI-----------------PATYLTGD---KTDAEA---ASIYLQLSK
KDP--IIKLLYVTPE-KVCAS---------------TRLISTMENLYERQLLARFVIDEA
HCV--SQWG------HDFRPDYKRLNVLRQKF--QS-VPMMALTATAN----------PR
VKKDILNQLKMT----KPQIFTM--SFNRDNLKYEVL---PK-------------KPKRV
AL--DCVEWIKKH-HPN----DSGIIYC-----LSRHEC--------DTM---------A
DTL---QKEGLAALA-YHAGLADSNR-DYVQHKW----INQDD-CQ----VICATIAFGM
GIDKPDVRYVIHASLPKS-VEGYYQESGRAG-RDGETSHCLLF-YSYHDVTRIRRLI---
--------QMEKDG--NSHTK--------QTHFNNLYSM--VHYCENVVE----------
CRRM---QLLSYFGE--NNFNPNFCKEHTQV--ACDNC--LG----KKNYKSR--DVTDD
VGNIVRFVQDNCS-----LVQGRGKGRSNN--TRLTLNMM-VD-IFLGSKSAKIQT----
------GLFGKGAAYSRHNAERLFRKLVLDRIIDEELYITFN-DQAV--AYVKMGERAQA

VLNGF----L-------------K---VDFQDTESASSIRKQKA---------SVVTNTS
Q--RE--E
MVKKCQAELTELCKRLGKIFGV-HYFNIFNTATIRRIA--ESLSPEPEVLLQIDGVTEDK
LDKYGAELIDVLQKYSE----------------WTLPVEDICQ--KSGGPANVSA---RR
YNSDH-----DDDESCD-------KSSYFSSNNKKGPKRK--NSAYFGKSKKRKTGG-DG
QQSRSKN---------GNSSYARKNSTAKTSSSYISGSKT--------------------
---------------------GADKR----------------------------------

--PGFMAPP-
--MPQ-------------
---------PNRRFLKPSYSMF-----------------
>H3ACE6
--------------------------------MTAVPQNNLQKQL---------------

--ELHS-AKGAQNKLS--LSKSKP--------GGFT------------------------
---------FKKKTPPGNNDATTFS-----------EVKLTNVLK--------DKDVNIH
ENISLAKPLTSSLTKHNDQQLKIGHFFEKNCKSQPQVTQMFS-----GQPFLNCSESV--
---RQTSKILPVFTRPEETIKEPKQPSAKPEESSKKSELDCSFSTVISI-EDWDDL-DDF
D--TSWQAKHYKSTPSSHQSKINLSSGTFNDSLSTRNSKNVL------------SPQKSI
QDTMDKDNQVGYANSFEISERSLTCVDLVSLNSTGSVVIDEEET----------------
---N
CIKENERRDTHRYSETQNRELKWRETDGDSNLVHQSNENFVFENDDFDIDFIP---PSPQ
DPMSSP---------------------------SFIQHKSNLKQSTCCSEGLLGMTISDP
VISEHNQDILRQPPQWSNSNREAEAFGSQDKLYR--------------------------
----------------------VMEEICR-------------------------------
--LVDTIPLDELKALS----
---------------------CGSQLLQQRQHR---------------------YFKLFF
HLIVNNIKRAIISGPNEKLLTNQNEPSLSSVPTLAASVHKRSLDPTPP------------
--------SSFPTDQMLSSSAEGTSTKAFK-----------FKKSSSTSFSPASC-----

-------------------------------GKSFFSDADFSIASELQTPELSGKSHS--
----------LSFTWLDPQKDQNQSNNCKTDLDGRNPPL---------------------
TEDADGFDVDDFDI---DDFYDDDI-----------VCSDSPSILGA-------------
--AEKLSTVLVQPIK--EGGPTKSFLEKVSYAMKK----------------------CEI
ILSHQPSLSLSCLQIF-----RLSIQHKPSFLNPSH-----DHFRGF-----Q-FPH---
------SKEMMKIFHKKFGLHQFRTNQLEAINCTLLG--EDCFIL---MPTDKGGGKSLC
--------YQLPA---------------------CL----SPGV-TIVVSPLRSLIVDQV
QKLTT------LDI-----------------PATYLTGD---KTDAEA---GSIYMQLSK
KDP--IVKLLYVTPE-KLCAS---------------NRLLSTLTNLYERKMLTCFVIDEA
HCV--SQWG------HDFRPDYKRLNELRQKF--PT-VRMMALTATAN----------PR
VQKDILNQLKML----RPQVFTM--SFNRHNLKYEVL---PK-------------KPKKV
AE--DCMEWIKKH-YPH----DSGIIYC-----LSRREC--------DTM---------A

```
DTL---KKAGLAALA-YHAGLGDSDR-DLVQQKW----INQDG-CQ----VICATIAFGM
GIDKPDVRYVIHASLPKS-VEGYYQESGRAG-RDGDISHCVLF-YTYNDVTRIKRLI---
--------QMEKDG--NYHTK--------QTHFNNLYSM--VHYCENVVD----------
CRRT---QLLAYFGE--NNFNPNFCKAHPEV--ICDNC--SM----KKDYKSR--NVTDE
VKAIIRFVQEHCT-----QSGGGKGKGTAQLRTRLTLNML-VD-IFLGTKSARIQS----
------GIFGRGAAYSRHNAERLFRKLVLDRILDEELYITAN-GQAI--AYETIGEKAQA
VLNGY--------------------LQVEFHDTKSASSVRKKA----------AVMKNVS
K------------------------------------------------------RD--E
MVQKCLGAITELCKKLGKVFGV-HYFNIFNTATIKKLA--ETLSSDPEVLLQIDGVTEDK
LDKYGAELIDVLQKYSE----------------WQLPEDESQI-----SDGSNTWIDTRR
HEEEK-----NGNKANT-------ASRYFNNINNKGGKRK--SAPSFKKSKKRKTVSGNQ
QLCSKSH-----MFSDYDTEYSRASSGFNSKPQSTTAFNSRSNTKK--------------
------------------------------------------------------------
------------------------------------------------------------
------------------------------------------------------------
------------------------------------------------------------
------------------------------------------------------------
------------------------------------------------------------
------------------------------------------------------------
----------------------------------------------------PGFMAVP-
--------------------------------------------VPRN------------
---------NQRSFLKPSFSLL-----------------
>W5NAF7
--------------------------------MQALPQNNLQKQL---------------
------------------------------------------------------------
--ELHS-SKAVQNRLS--LNKPRS--------GTFS------------------------
---------FKKKSASGITKVAVLP-----------KVTVSNALV--------DRDVNAF
HHSKVTKPLTSLNKPEEQQLTHKAKS----------------------QNFTPVVTRP--
-----------------AVSNIKSALVNTDVGKKDDSEKTALNNSLSVSIDDWDDF-DDF
DFGTPVKDKSVTRLSQSNSGENFKKESASNLNKPCLKTSRAA-------------EKILS
ESSD-HNISNGLQNQEEPLEAQNGPAALPVQGSLIVLIDESKQI----------------
------------------------------------------------------------
-------------------------------DTPIAEIENRSENDNEELDFVP---PSPE
SETI------------------------------SPLLMKQPFSDAAGIFSMFYTRNQIL
KLLYYLSIQNHST---SPRLYAVSGTDSENQLFS--------------------------
----------------------IMEEICQ-------------------------------
------------------------------------------LVDSIPEHELTALS----
---------------------CGRDLLFQRARR----------------------RRFFA
ESCRTPQAPRWTGSHEASNAKTDNQWSHFKTPVSHSSSFKTIPSDGDG------------
---------SVFEESDLSDTGGIQSPSPSY-----------RPRGVPNVSFNGES-----
------------------------------------------------------------
-----DSRNTF------------------------YSKFPSFSSASNQMPNLEETD----
----------DCVCLADTPSLRIQNK----TNTPASDSY---------------GVDPSL
DDIDNEFTFDNFDI---DDFDEEDM---------GDYFEGNDSA----------------
--SKKQGTEGVQPVR--EGHPSKSLWEKKSPSSSFSSSA------------------AKP
SKPTSECSLPEPIN-----------------RNPAH-----DRFRGC-----N-FPY---
------SQEMMKIFNKRFGLRQFRTNQLEAINATLHC--EDTFVL---MPT--GGGKSLC
```

--------YQLPA---------------------CV----SPGV-TIVISPLRSLIVDQV
QKLTT------LDI-----------------PAAGLTGF---KSDSES---ARVYMQLSK
KDP--IIKLLYCTPE-KVCAS---------------NKFISALRNLYERSLLSRIVIDEA
HCV--SQWG------HDFRPDYKRLHELRRMF--PS-VPIMALTATAN----------PR
VQKDILNQLQML----KPQVFTM--SFNRHNLKYAVL---PK-------------KPKKI
AE--DCLEWIKKY-HPR----DSGIIYC-----LSRNNC--------DSM---------A
ESL---QRGGITALA-YHAGLKDSDR-DYVQQKW----IEQDE-CQ----VMCATIAFGM
GIDKPDVRFVIHASLPKS-VEGYYQESGRAG-RDGEISHCVLF-YSYNDAIRIKRLI---
--------NMERDG--NKETK--------QTHITNLYSM--VHFCENVVE----------
CRRT---QLLAYFGE--RTFNPNFCKEHPDV--TCDNC--AR----AKLYKSR--NVTEE
VKNVVRFVQEHCA-----KVGVRQAKSAQL--NRLTLNML-VD-IFIGTKSARIQT----
------GLYGKGAAYSRHNAERLFRKLVLENVLEESLYITAN-DQAV--AYISAGQKAAA
VLSGF----M-------------Q---IEFYDTESASSIKKQRA---------SVTKNMS
K--RE--E
MVQNCLQELNDLCKKLGKIFGI-HYYNIFSTATLKKIA--ETLSADPEVLLQIDGVTEDK
LEKYGAELIQLLQKYSD----------------WQLPVEEHTE----KPVGNDGWIDVER
NRREEQEL--CDEDAG--------GSSYFKSTSERGGKRK--KPSYFNKAKRRKGGYNSS
TKGSNSN------NARASSTASKGGSKDRGQDSAGSSKPAMATKR---------------

--PGFMAPP-
--VPRS------------
---------NQRPFLKPAFSHM-----------------
>W5NAF6
--------------------------------MQALPQNNLQKQL---------------

--ELHS-SKAVQNRLS--LNKPRS--------GTFS------------------------
---------FKKKSASGITKVAVLP-----------KVTVSNALV--------DRDVNAF
HHSKVTKPLTSLNKPEE--QQARINSFFELTHKAKSQNFTPV------------------
-------------VTRPAVSNIKSALVNTDVGKKDDSEKTALNNSLSVSIDDWDDF-DDF
DFGTPVKDKSVTRLSQSNSGENFKKESASNLNKPCLKTSRAA--------EKILSESSDA
FAAV-EKPQGCVEDKEEEDDVPIKFARKRHLSQIAQILSDSEDD----------------
-----CVEEGDFPN---K
KKGKSKQIDT-----------------------PIAEIENRSENDNEELDFVP---PSPE
SETI-----------------------------SPLLMKQPFRKPNSEAFILSFYTKYST
SPRLNICIFSGTD--------------SENQLFS--------------------------
----------------------IMEEICQ-------------------------------
--LVDSIPEHELTALS----
---------------------CGRDLLFQRARR---------------------KMLIS
HSSSFKTIPSGKDQSSAKS--------FRFRKPSSVPFADGDGS----------------
----------VFEESDLSDTGGIQSPSPSY-----------RPRGVPNVSFNGES-----
--DS

RKEKLGVGNNGKDSRSDTF----------------YSKFPSFSSASNQMPNLEETD----
----------DCVCLADTPSLRIQNK----TNTPASDSY---------------GVDPSL
DDIDNEFTFDNFDI---DDFDEEDM---------GDYFEGNDSA----------------
--SKKQGTEGVQPVR--EGHPSKSLWEKKSPSSSFSSSA------------------AKP
SKPTSECSLPEPIN-----------------RNPAH-----DRFRGC-----N-FPY---
------SQEMMKIFNKRFGLRQFRTNQLEAINATLHC--EDTFVL---MPT--GGGKSLC
--------YQLPA---------------------CV----SPGV-TIVISPLRSLIVDQV
QKLTT------LDI-----------------PAAGLTGF---KSDSES---ARVYMQLSK
KDP--IIKLLYCTPE-KVCAS---------------NKFISALRNLYERSLLSRIVIDEA
HCV--SQWG------HDFRPDYKRLHELRRMF--PS-VPIMALTATAN----------PR
VQKDILNQLQML----KPQVFTM--SFNRHNLKYAVL---PK-------------KPKKI
AE--DCLEWIKKY-HPR----DSGIIYC-----LSRNNC--------DSM---------A
ESL---QRGGITALA-YHAGLKDSDR-DYVQQKW----IEQDE-CQ----VMCATIAFGM
GIDKPDVRFVIHASLPKS-VEGYYQESGRAG-RDGEISHCVLF-YSYNDAIRIKRLI---
--------NMERDG--NKETK--------QTHITNLYSM--VHFCENVVE----------
CRRT---QLLAYFGE--RTFNPNFCKEHPDV--TCDNC--AR----AKLYKSR--NVTEE
VKNVVRFVQEHCA-----KVGVRQAKSAQL--NRLTLNML-VD-IFIGTKSARIQT----
------GLYGKGAAYSRHNAERLFRKLVLENVLEESLYITAN-DQAV--AYISAGQKAAA
VLSGF----M-------------Q---IEFYDTESASSIKKQRA---------SVTKNMS
K--RE--E
MVQNCLQELNDLCKKLGKIFGI-HYYNIFSTATLKKIA--ETLSADPEVLLQIDGVTEDK
LEKYGAELIQLLQKYSD----------------WQLPVEEHTE----KPVGNDGWIDVER
NRREEQEL--CDEDAG--------GSSYFKSTSERGGKRK--KPSYFNKAKRRKGGYNSS
TKGSNSN------NARASSTASKGGSKDRGQDSAGSSKPAMATKR---------------

--PGFMAPP-
--VPRS------------
---------NQRPFLKPAFSHM-----------------
>W5NAF4
---------------------VLHLFTAIIYTMQALPQNNLQKQL---------------

--ELHS-SKAVQNRLS--LNKPRS--------GTFS------------------------
---------FKKKSASGITKV---------------------------------------
-----------AVLPKPEEQQARINSFFELTHKAKSQNFTPVVTRPAVSNIKLSLSTT--
------------------KASPQSALVNTDVGKKDDSEKTALNNSLSVSIDDWDDF-DDF
DFGTPVKDKSVTRLSQSNSGENFKKESASNLNKPCLKTSRAA--------EKILSESSDH
NISN-GLQNQEEPLEAQNGPAALPVQCRPLPDAFAAVEKPQGCV--------------ED
KEEILSDSEDDCVEEGDFPN----------KKKEQGSLIVLIDESK--------------
-----------------------------QIDTPIAEIENRSENDNEELDFVP---PSPE
SETI-----------------------------SPLLMKQPFRQNELVSKQQKHETADDG
DSEEIKNSTSPRA---------VSGTDSENQLFS--------------------------

```
----------------------IMEEICQ-------------------------------
------------------------------------------LVDSIPEHELTALS----
---------------------CGRDLLFQRARR----------------------RRFFA
ESCRTPQAPRWTGSHEASNAKTDNQWSHFKTPVSHSSSFKTIPSGKDQSSAKSFRFRKPS
SVPFADGDGSVFEESDLSDTGGIQSPSPSY-----------RPRGVPNVSFNGES-----
----------------------------------------------------------DS
RKEKLGVGNNGKDSRSDTF----------------YSKFPSFSSASNQMPNLEETD----
----------DCVCLADTPSLRIQNK----TNTPASDSY---------------GVDPSL
DDIDNEFTFDNFDI---DDFDEEDM---------GDYFEGNDSA----------------
--SKKQGTEGVQPVR--EGHPSKSLWEKSFSSSAAKPSK------------------PTS
ECSLPEPIN----------------------RNPAH-----DRFRGC-----N-FPY---
------SQEMMKIFNKRFGLRQFRTNQLEAINATLHC--EDTFVL---MPT--GGGKSLC
--------YQLPA---------------------CV----SPGV-TIVISPLRSLIVDQV
QKLTT------LDI-----------------PAAGLTGF---KSDSES---ARVYMQLSK
KDP--IIKLLYCTPE-KVCAS---------------NKFISALRNLYERSLLSRIVIDEA
HCV--SQWG------HDFRPDYKRLHELRRMF--PS-VPIMALTATAN----------PR
VQKDILNQLQML----KPQVFTM--SFNRHNLKYAVL---PK-------------KPKKI
AE--DCLEWIKKY-HPR----DSGIIYC-----LSRNNC--------DSM---------A
ESL---QRGGITALA-YHAGLKDSDR-DYVQQKW----IEQDE-CQ----VMCATIAFGM
GIDKPDVRFVIHASLPKS-VEGYYQESGRAG-RDGEISHCVLF-YSYNDAIRIKRLI---
--------NMERDG--NKETK--------QTHITNLYSM--VHFCENVVE----------
CRRT---QLLAYFGE--RTFNPNFCKEHPDV--TCDNC--AR----AKLYKSR--NVTEE
VKNVVRFVQEHCA-----KVGVRQAKSAQL--NRLTLNML-VD-IFIGTKSARIQT----
------GLYGKGAAYSRHNAERLFRKLVLENVLEESLYITAN-DQAV--AYISAGQKAAA
VLSGF----M-------------Q---IEFYDTESASSIKKQRA---------SVTKNMS
K------------------------------------------------------RE--E
MVQNCLQELNDLCKKLGKIFGI-HYYNIFSTATLKKIA--ETLSADPEVLLQIDGVTEDK
LEKYGAELIQLLQKYSD----------------WQLPVEEHTE----KPVGNDGWIDVER
NRREEQEL--CDEDAG--------GSSYFKSTSERGGKRK--KPSYFNKAKRRKGGYNSS
TKGSNSN------NARASSTASKGGSKDRGQDSAGSSKPAMATKR---------------
------------------------------------------------------------
------------------------------------------------------------
------------------------------------------------------------
------------------------------------------------------------
------------------------------------------------------------
------------------------------------------------------------
------------------------------------------------------------
----------------------------------------------------PGFMAPP-
--------------------------------------------VPRS------------
---------NQRPFLKPAFSHM-----------------
>W5NAF0
--------------------------------MQALPQNNLQKQL---------------
------------------------------------------------------------
--ELHS-SKAVQNRLS--LNKPRS--------GTFS------------------------
---------FKKKSASGITKVAVLP-----------KVTVSNALV--------DRDVNAF
HHSKVTKPLTSLNKPEE--QQARINSFFELTHKAKSQNFTPVVTRPAVSNIKLSLSTT--
------------------KASPQSALVNTDVGKKDDSEKTALNNSLSVSIDDWDDF-DDF
```

DFGTPVKDKSVTRLSQSNSGENFKKESASNLNKPCLKTSRAA--------EKILSESSDH
NISN-GLQNQEEPLEAQNGPAALPVQCRPLPDAFAAVEKPQ------------------G
CVEDKEEEDDVPIKFARKR-----------HLSQIAQILSDSEDDCVEEG---------D
FPNKKKGLQS------------------KQIDTPIAEIENRSENDNEELDFVP---PSPE
SETI-------------------------------SPLLMKQPFSGAETHESSYNGHFSS
KELVSKQQKHETADDGDSEEIKISGTDSENQLFS--------------------------
----------------------IMEEICQ-------------------------------
--LVDSIPEHELTALS----
---------------------CGRDLLFQRARR----------------------RRFFA
ESCRTPQAPRWTGSHEASNAKTDNQWSHFKTPVSHSSSFKTIPSGKDQSSAKSFRFRKPS
SVPFADGDGSVFEESDLSDTGGIQSPSPSY-----------RPRGVPNVSFNGES-----
---DSRKEKL
GVGNNGKDSRSDTF---------------------YSKFPSFSSASNQMPNLEETD----
----------DCVCLADTPSLRIQNK----TNTPASDSY---------------GVDPSL
DDIDNEFTFDNFDI---DDFDEEDM---------GDYFEGNDSA----------------
--SKKQGTEGVQPVR--EGHPSKSLWEKKSPSSSFSSSA------------------AKP
SKPTSECSLPEPIN-----------------RNPAH-----DRFRGC-----N-FPY---
------SQEMMKIFNKRFGLRQFRTNQLEAINATLHC--EDTFVL---MPT--GGGKSLC
--------YQLPA---------------------CV----SPGV-TIVISPLRSLIVDQV
QKLTT------LDI-----------------PAAGLTGF---KSDSES---ARVYMQLSK
KDP--IIKLLYCTPE-KVCAS---------------NKFISALRNLYERSLLSRIVIDEA
HCV--SQWG------HDFRPDYKRLHELRRMF--PS-VPIMALTATAN----------PR
VQKDILNQLQML----KPQVFTM--SFNRHNLKYAVL---PK-------------KPKKI
AE--DCLEWIKKY-HPR----DSGIIYC-----LSRNNC--------DSM---------A
ESL---QRGGITALA-YHAGLKDSDR-DYVQQKW----IEQDE-CQ----VMCATIAFGM
GIDKPDVRFVIHASLPKS-VEGYYQESGRAG-RDGEISHCVLF-YSYNDAIRIKRLI---
--------NMERDG--NKETK--------QTHITNLYSM--VHFCENVVE----------
CRRT---QLLAYFGE--RTFNPNFCKEHPDV--TCDNC--AR----AKLYKSR--NVTEE
VKNVVRFVQEHCA-----KVGVRQAKSAQL--NRLTLNML-VD-IFIGTKSARIQT----
------GLYGKGAAYSRHNAERLFRKLVLENVLEESLYITAN-DQAV--AYISAGQKAAA
VLSGF----M-------------Q---IEFYDTESASSIKKQRA---------SVTKNMS
K--RE--E
MVQNCLQELNDLCKKLGKIFGI-HYYNIFSTATLKKIA--ETLSADPEVLLQIDGVTEDK
LEKYGAELIQLLQKYSD----------------WQLPVEEHTE----KPVGNDGWIDVER
NRREEQEL--CDEDAG--------GSSYFKSTSERGGKRK--KPSYFNKAKRRKGGYNSS
TKGNNAR---------ASSTASKGGSKDRGQDSAGSSKPAMATKR---------------

--PGFMAPP-
--VPRS------------
---------NQRPFLKPAFSHM-----------------
>A0A2D0QTF4

```
--------------------------------MPTLPLNNLKEQL---------------
------------------------------------------------------------
--ERHSSVAARSKSLAKAKSGSYC------------------------------------
-----------------------------------------------------FKKKSTP
DSAKAEVPQKLLCKAKTQTFNPVANPFAVSKPSLVEPSISRL------------------
--------------------QKPRNNRITGTTAGKAVALAEDYILSAIPLDDWDRY--DF
ETPVKGEG----------------------------------------------------
---------------------AGPDKDPSPKGAGAQTCNTENGD----------------
------------------------------------------------------------
--------------------------------LPLVHSFGTSDEACVVSTEILAVDPGAQ
SGHT-----------------------------DGESPINTIGRLISVKHKLLLSDKSIA
TGIKPTDTIEGNC---------NCTTGFDDTLFS--------------------------
----------------------VMESICC-------------------------------
------------------------------------------LMDTIPEHELASLS----
---------------------CGTELLLLRAHR----------------------KRIIT
GAALVEKRKSEGGTNPHPRSLHKASSAVTSKGDHSSIHGQLSKSNASV------------
---------MSQDYNNCIFKDSDCIISDVIKGD--------ACSLNPSIIHAVNQ-----
----------------------------------------------------------HK
GKISGLNNGNGNIQTGEC-----------------SLSLSFNKSNVQTVNQE--------
----------KTLHYISPKNTVEVDRKNVDSKDTLVGGA--------------GLQDHRE
SDDMGDFCIGDFDI---DDFSENDM---------PDYCEEPAALSVS-------------
------GSSSALPQQFCEGGHSNSCDKKSV---------------------------ANP
TAVVRSTKPCFPEVTS---------------RNPAH-----GCFWGF-----N-FPH---
------SPEMMKIFHKKFGLHQFRFNQLEAINATLLG--EDTFVL---MPT--GGGKSLC
--------YQLPA---------------------CV----SAGV-TVVISPLRSLIVDQV
QKLTT------LDV-----------------PATSLSGE---KSDYEA---GRIYMQLSK
NDP--VIKLLYATPE-KICAS---------------GRMISALQNLYERGLLARFVIDEA
HCV--SQWG------HDFRPDYKRMHELRQKF--PK-VPIMALTATAT----------PR
VQKDILNQLSMT----RPQVFSM--SFNRHNLKYSIV---PK--------------KPKKV
DE--DCISWIKKH-YPY----DSGIVYC-----LSRNDC--------DSM---------A
DSF---QRAGIAALA-YHAGINDSDR-EYVQNKW----INQDG-CQ----VICATIAFGM
GIDKPDVRYVIHASLPKS-VEGYYQESGRAG-RDGEISHCILF-YSYTDVIRIKRLI---
--------AMDKEG--NHQSK--------ATHINNLHSM--VHFCENVTE----------
CRRI---QLLAYFGE--HKFNTDFCKQHPEV--ICDNC--AR----PQQYSAR--NVTED
VKKIVRFIQENCE-----KVGGKRVRSFQQ--SRLTLNML-VD-IFLGLKSARIKS----
------GMYGIGAAYSKHNAERLFKKLVLDHILMEDLYITNN-GQAV--AYISAGLKAMD
VLSGI--------------------MQVNFHETESASSIRKQKA---------TVTKSVS
K------------------------------------------------------RE--E
MVKKCLEELNELCKKLGKVFGL-HYYNIFSTATLKKIA--ETLSADPEVLLQIDGVTEDK
LVKYGSELIELLQKYSK----------------WQLTVEEQAE---------------GS
HSIDY-----YNEDGSM--------SAYFRSSKGQGEKRK--KTHLSKNPKRRRGYNNQN
SSKR------------GNWSSSKGRDLGTDRVSKHAPSAPAKRK----------------
------------------------------------------------------------
------------------------------------------------------------
------------------------------------------------------------
------------------------------------------------------------
------------------------------------------------------------
```


--PGFMALP-
--LPQS------------
---------VERPFLRPTFSQVG----------------
>A0A2D0QSS8

--MNNITNTA
RSYCFKKKSTPDSAKAEVPQKLLCKAKTQTFNPVANPFAVSK-----PSLVEPSISRL--
--------------------QKPRNNRITGTTAGKAVALAEDYILSAIPLDDWDRY--DF
E--TPVKGEGAG--
-----------------------PDKDPSPKGAGAQTCNTENGD----------------

--------------------------------LPLVHSFGTSDEACVVSTEILAVDPGAQ
SGHT-----------------------------DGESPINTIGRLISVKHKLLLSDKSIA
TGIKPTDTIEGNC---------NCTTGFDDTLFS--------------------------
----------------------VMESICC-------------------------------
--LMDTIPEHELASLS----
---------------------CGTELLLLRAHR----------------------KRIIT
GAALVEKRKSEGGTNPHPRSLHKASSAVTSKGDHSSIHGQLSKSNASV------------
---------MSQDYNNCIFKDSDCIISDVI-----------KGDACSLNPSIIHA-----
---VNQHK
GKISGLNNGNGNIQTGEC-----------------SLSLSFNKSNVQTVNQEKT------
----------LHYISPKNTVEVDRKN----VDSKDTLVG------------GAGLQDHRE
SDDMGDFCIGDFDI---DDFSENDM---------PDYCEEPAALSVS-------------
------GSSSALPQQFCEGGHSNSCDKKSV---------------------------ANP
TAVVRSTKPCFPEVTS---------------RNPAH-----GCFWGF-----N-FPH---
------SPEMMKIFHKKFGLHQFRFNQLEAINATLLG--EDTFVL---MPT--GGGKSLC
--------YQLPA---------------------CV----SAGV-TVVISPLRSLIVDQV
QKLTT------LDV-----------------PATSLSGE---KSDYEA---GRIYMQLSK
NDP--VIKLLYATPE-KICAS---------------GRMISALQNLYERGLLARFVIDEA
HCV--SQWG------HDFRPDYKRMHELRQKF--PK-VPIMALTATAT----------PR
VQKDILNQLSMT----RPQVFSM--SFNRHNLKYSIV---PK-------------KPKKV
DE--DCISWIKKH-YPY----DSGIVYC-----LSRNDC--------DSM---------A
DSF---QRAGIAALA-YHAGINDSDR-EYVQNKW----INQDG-CQ----VICATIAFGM
GIDKPDVRYVIHASLPKS-VEGYYQESGRAG-RDGEISHCILF-YSYTDVIRIKRLI---
--------AMDKEG--NHQSK--------ATHINNLHSM--VHFCENVTE----------
CRRI---QLLAYFGE--HKFNTDFCKQHPEV--ICDNC--AR----PQQYSAR--NVTED
VKKIVRFIQENCE-----KVGGKRVRSFQQ--SRLTLNML-VD-IFLGLKSARIKS----
------GMYGIGAAYSKHNAERLFKKLVLDHILMEDLYITNN-GQAV--AYISAGLKAMD
VLSGI----M-------------Q---VNFHETESASSIRKQKA---------TVTKSVS
K--RE--E
MVKKCLEELNELCKKLGKVFGL-HYYNIFSTATLKKIA--ETLSADPEVLLQIDGVTEDK
LVKYGSELIELLQKYSK----------------WQLTVEEQAE---------------GS
HSIDY-----YNEDGSM--------SAYFRSSKGQGEKRK--KTHLSKNPKRRRGYNNQN

```
SSKR------------GNWSSSKGRDLGTDRVSKHAPSAPAKRK----------------
------------------------------------------------------------
------------------------------------------------------------
------------------------------------------------------------
------------------------------------------------------------
------------------------------------------------------------
------------------------------------------------------------
------------------------------------------------------------
----------------------------------------------------PGFMALP-
--------------------------------------------LPQS------------
---------VERPFLRPTFSQVG----------------
>A0A1W4ZRM4
-----------MLKLNLPATSVFFSGVFLIFMMPTVPANNLKEQL---------------
------------------------------------------------------------
--ERHTGQNKMSLSKAKAGTFAFK------------------------------------
-----------------------------------------------------KKSESCV
AKPAVPSEVNANTLVNRNIKVLYHKTVFNTLTSLTETERQPAKSSSFFTTPKQQVMAP--
-TVDRSPVCSSTPAFSTSKVPSARSKPHIASTANSCSQNIELDSSYIIDIDSWDDF-DDF
ETPVRGKTSTLLTSVDLQRTSNAGDLSTVSKKDTETDKMARN------------------
-----------------CHYSPLDSKAGKSLQKGDDLHLTLSHP--------------TE
VESLNCENASGCNAVSKTKNLKGSKIWKQGMSLMTPTKKSSFVPPELLLSPREETFTADA
KCCDRKTVDG--------------------STMMDADVADVPMDFPEDVDFVP---PSP-
---------------------------------EAGRLSSPLSFENRSKVAIQDASDISC
ISPKQLVSSILNNKSHLVVKDKTRSESEDELIV---------------------------
----------------------VMETICC-------------------------------
------------------------------------------LVDSIPEHELISLA----
---------------------CGTELLLQRAHR----------------------KRILA
QSVDLSKKCYKERRAVSETKMKKSQESYEYTQQHAVSAMKAEDSGKTI------------
---------QFRESLSVLSGDGENTVFDDSNCNISGIQTVPAFQRTSSLFSPSGT-----
------------------------------------------------------------
-----------------------------------IEKELGFTTKSYERPTSDGKD----
----------GQKCKPDNSVLAQTSS------------------------------DAQV
DTEQYDFYFDDFDI---DDFDESDI---------PNCSDGPPHQPIN-------------
--------TVSQPLR--EGGQSRTAYE------------------------------NTS
SHSYSTTKPAKFGLAEPVVRNH-------------------DRFRGF-----N-FPH---
------SPEMMKIFHKRFGLHQFRFNQLEAINASLLG--EDTFVL---MPT--GGGKSLC
--------YQLPA---------------------CV----SQGV-TVVVSPLRSLIVDQV
QKLTT------LDI-----------------PAASLSGD---KSDSEA---GRIYMQLSK
KDP--LIKLLYSTPE-KICAS---------------GRLMSALQNLYERGLLARFVIDEA
HCV--SQWG------HDFRPDYKRMYELRQKF--PK-VPIMALTATAT----------PR
VQKDILTQLQMS----KPQVFTM--SFNRHNLKYSVL---PK-------------KPKKV
DE--DCIQWIKKN-YPR----DSGIVYC-----LSRNDC--------DNL---------A
ASL---QRAGIAALS-YHAGLNDTDR-EYVQNKW----INQDD-CQ----VICATIAFGM
GIDKPDVRYVIHASLPKS-VEGYYQESGRAG-RDGEISHCVLF-YSYADAIRIKRLI---
--------SMDREG--NQHTK--------RTHYNNLHSM--VHFCENLMD----------
CRRV---QLLAYFGE--HDFDPDFCKNYPEV--ICDNC--AK----PNQYKLQ--NVTED
VKKIVRFVQDNCE-----KIGVKRNSCAQQ--NRLTLNML-VD-IFLGSKSARIQT----
```

------GMFGMGAAYSRHNAERLFKKLVLDCILQEDLYITAN-AQAV--GYISAGEKAGS
VLYGS--------------------MQVEFYQTESASSIRKNKA---------SVTKSTS
Q--RE--E
MVHKCLQELNELCKKLGKVFGI-HYYNIFSTATLKKIA--ETLSADPDVLLQIDGVTEDK
LEKYGVELIELLQKYSE----------------WQLPAGEQMESHSCTRSLKSTGRNRGR
QDKDE-----QDGIDE--------ASTYFKSRRVRGQKRK--QNSYFKNSKKQRTYPNQQ
SSTKSNE---------KPWISGSFQGRSKGRTRTATGSSTLSAQA---------------
---------------------PVARR----------------------------------

--PGFMTLP-
--VPHT------------
---------NQRPFLKPSFSHLV----------------
>A0A1S3KNL0
-------------------------------MMSSLPQNNLKEQL---------------

--ERHS--NAAQRKLS--LSKPKP--------GTFC------------------------
---------FKKKSTSGITKVEVPTKPERPLAKINFSSVNPKSKY--------EAISPLL
NPFSVRKPIPAVSAAKISPALTKSDSLITGSNGSKPTGLDSS------------------
---FGISLDGWDDF-DDF
E--TPVKGKSTLPSPGI-SGRSAKVTPVSSVEQLDTNTHRSD--------KDVPLQALNT
KTNS-RHRNKCSLDGTEQCTLTMEDSQDASESASSAPRNNPIQD---------------P
AEWKHWEVEDSPIKMVRRRP----------SASQAKSVLSDSEDESI------------T
APGPIEKTDH-----------------QSKWNETDVIDLDDKSELDGDLDLIS---PSPS
PEDF------------------------------SHSTSTKEARLKSAGSALRKDISPVS
TRGSVSSLQRVSD--GPLKERKVTNPTSEDQLLS--------------------------
----------------------IMESICA-------------------------------
--LVDSIPDHELLGLS----
---------------------CGTELLLQRAHR----------------------KRIIA
--TGSSDTYFRTQQPDSTVLESR----FSEKTTFSFATPSVMSSTTCLPKGKGDSSMKAL
PFRRSSVISVDYDSSVFEDSDCMINVQTPGISW--------RPSTQENPIFTGDK-----

KKASTTGRSNCDSP----------------------SNYGIFKKQIGDVAQS--------
----------NRFFSPKSPAAAVQMT----GESSTQASV----------------AQAPA
VTEPDDFFIDDFDI---DDFNDSDI---------PDYFEPPSSSV---------------
--SRQDSSSVSKAVR--EGGPSTSTWQKKP---ATPAPV------------------AKP
PKITSPEPTY---------------------RNPAH-----DRFRGF-----N-FPH---
------CPEMMKIFYKRFGLHQFRFNQLEAINATLLG--EDTFVL---MPT--GGGKSLC
--------YQLPA---------------------CV----CTGV-TVVISPLKSLIVDQV
QKLTT------LDI-----------------PATSLSGE---KTDSEA---ARIYLQLSK
KDP--IIKLLYATPE-KVCAS---------------GRMISALQNLYERGLLARFVIDEA
HCV--SQWG------HDFRPDYKRMYELRQKF--PK-VPIMALTATAT----------PR
VQKDILNQLQMT----QPQVFTM--SFNRNNLKYAVL---PK-------------KPKKV

DE--DCIDWIKKN-YPR----DSGIVYC-----LSRNDC--------DNM---------A
DSL---QRAGILALA-YHAGINDKDR-EYVQTKW----INQDG-CQ----VICATIAFGM
GIDKPDVRYVIHASLPKS-VEGYYQESGRAG-RDGEISHCILF-YSYSDVIRIKRIL---
--------TMDKDG--NHHTK--------ATHLNNLHSM--VHFCENVMD----------
CRRI---QLLAYFGE--HKFNPGFCKEHPDV--ICDNC--AR----PNQYKAR--NVTED
VKNIVRFVQENCE-----KVGSRYNKSANQ--NRLTLNML-VD-IFLGAKTARVQS----
------GMFAIGKAYSKHNADRLFKKLVLDHLLVEDLYITAN-SQAV--AYISAGPKAIN
VLGGH----M-------------Q---VEFYETESASSIRKHKT---------AVAKDVS
K--SE--E
MVQKCLQELTDLCKRLGKVFGI-HYYNIFSTATLKMIA--ETLSADPDVLLTIDGVTEDK
LEKYGADVIALLQKYSE----------------WKLPVEEQSD--TTEGSSTMAWIDPRR
GRGDD-----EEDCYDDAG-----SSSYFNNNNGRGAKRK--KAPAFKKSKKRKGYTNSN
SKGGYSN--------NGSWTGSNSRGGSKGRGSAAGRGSAAGRGS---------------
---------------RPSAAGTVGRR----------------------------------

--PGFMALP-
--TPQT------------
---------NSRPFLKPAFSVL-----------------
>A0A1S3KNZ2
-------------------------------MMSSLPQNNLKEQL---------------

--ERHS--NAAQRKLS--LSKPKP--------GTFC------------------------
---------FKKKSTSGITKVEVPT-----------KVNSSNGLT--------NRSVNVP
HDNAVIKPPLTFSTKPERPLAKINFSSVNPKSKYEAISPLLNPFSVRKPIPAVSAAKI--
-----------------SPALTKSDSLITGSNGSKPTGLDSSFGISL---DGWDDF-DDF
E--TPVKGKSTLPSPGI-SGRSAKVTPVSSVEQLDTNTHRSD--------KDVPLQALNT
KTNS-RHRNKCSLDGTEQCTLTMEDSQDASESASSAPRNNPIQD---------------P
AEWKHWEVEDSPIKMVRRRP----------SASQAKSVLSDSEDESI------------T
APGPIEKTDH---------QSKWNET--------DVIDLDDKSELDGDLDLIS---PSPS
PEDF------------------------------SHSTSTKEARLKSAGSALRKDISPVS
TRGSVSSLQRVSD--GPLKERKVTNPTSEDQLLS--------------------------
----------------------IMESICA-------------------------------
--LVDSIPDHELLGLS----
---------------------CGTELLLQRAHR----------------------KRIIA
--TGSSDTYFRTQQPDSTVLESR----FSEKTTFSFATPSVMSSTTCLPKGKGDSSMKAL
PFRRSSVISVDYDSSVFEDSDCMINVQTPGISW--------RPSTQENPIFTGDK-----

KKASTTGRSNCDSP----------------------SNYGIFKKQIGDVAQS--------
----------NRFFSPKSPAAAVQMT----GESSTQASV----------------AQAPA
VTEPDDFFIDDFDI---DDFNDSDI---------PDYFEPPSSSV---------------
--SRQDSSSVSKAVR--EGGPSTSTWQKKP---ATPAPV------------------AKP
PKITSPEPTY---------------------RNPAH-----DRFRGF-----N-FPH---

------CPEMMKIFYKRFGLHQFRFNQLEAINATLLG--EDTFVL---MPT--GGGKSLC
--------YQLPA---------------------CV----CTGV-TVVISPLKSLIVDQV
QKLTT------LDI-----------------PATSLSGE---KTDSEA---ARIYLQLSK
KDP--IIKLLYATPE-KVCAS---------------GRMISALQNLYERGLLARFVIDEA
HCV--SQWG------HDFRPDYKRMYELRQKF--PK-VPIMALTATAT----------PR
VQKDILNQLQMT----QPQVFTM--SFNRNNLKYAVL---PK-------------KPKKV
DE--DCIDWIKKN-YPR----DSGIVYC-----LSRNDC--------DNM---------A
DSL---QRAGILALA-YHAGINDKDR-EYVQTKW----INQDG-CQ----VICATIAFGM
GIDKPDVRYVIHASLPKS-VEGYYQESGRAG-RDGEISHCILF-YSYSDVIRIKRIL---
--------TMDKDG--NHHTK--------ATHLNNLHSM--VHFCENVMD----------
CRRI---QLLAYFGE--HKFNPGFCKEHPDV--ICDNC--AR----PNQYKAR--NVTED
VKNIVRFVQENCE-----KVGSRYNKSANQ--NRLTLNML-VD-IFLGAKTARVQS----
------GMFAIGKAYSKHNADRLFKKLVLDHLLVEDLYITAN-SQAV--AYISAGPKAIN
VLGGH----M-------------Q---VEFYETESASSIRKHKT---------AVAKDVS
K--SE--E
MVQKCLQELTDLCKRLGKVFGI-HYYNIFSTATLKMIA--ETLSADPDVLLTIDGVTEDK
LEKYGADVIALLQKYSE----------------WKLPVEEQSD--TTEGSSTMAWIDPRR
GRGDD-----EEDCYDDAG-----SSSYFNNNNGRGAKRK--KAPAFKKSKKRKGYTNSN
SKGGYSN--------NGSWTGSNSRGGSKGRGSAAGRGSAAGRGS---------------
---------------RPSAAGTVGRR----------------------------------

--PGFMALP-
--TPQT------------
---------NSRPFLKPAFSVL-----------------
>W5LCA5
--------------------------------MFSLPQNNLKEQL---------------

--ERHSKVSAAQNKLS--LAKPKP------------------------------------
---GAFCF
RKTSTSTTSKAAVPEKKPERAPPKFNFFSAPSKPKTQSINPS------------------
--ANPFAVTKPTLIQSVIKTSAKSETIPPDTHEDKSAESLVGNDSFAIPVDDWDDL-DDF
E--TPSKVRASQSPQIPVKKTSGLDKNVSPKAASAKSADTTE------------------
-----------------NGSLPVGPSFGACGEALEIPTDTSGAD--------------RH
DAISAGEPDDSPIKLTKRRL----------SAVKQKPLLSDSEDESIT-----------D
NIKPHNKADENFKYSEENEQKCKSPK------IIDIDDSQEEDVLEDDFDFIP---PSPI
PEDQ---------------------------------SPSVPTLEKRKPDLIKNEVASVA
SSRCSVSSLSGQNVPAKDLKGPDDALFS--------------------------------
----------------------IMESICC-------------------------------
--LVDKIPEHELIALT----
---------------------CGTELLLQRAHR----------------------KRILA
EIASSGKSVSVSGAACD----------FRLLHRGSFGVPTPLISGKGDSALKGFQYRKSF
VSTMSVDYNNSVFEDSDCIINGVETPGGSW-----------NPNPSLKSAAKGDQ-----

ENTCLKKNNVHI-----------------------ESSSLLLKQTDDQTEDLLQT-----
----------NLFFSPKKRVGTIKKNKEDKVENNTAGSV---------------YVDSEM
DTVPDDFYDDDFDI---DDFNESDI---------PKYNEEPVDSLA--------------
--TKKSSSATPKPIY--EGGPSKSCEKRTV---------------------------TTP
TAVVKPTKPASPEVTY---------------RNPAH-----DRFRGF-----S-FPH---
------SSEMMKIFHKRFGLHQFRFNQLEAINATLLG--EDTFVL---MPT--GGGKSLC
--------YQLPA---------------------CV----SAGV-TVVISPLRSLIVDQV
QKLTT------LDI-----------------PATSLSGD---KSESEA---GRIYMQLSK
KDP--VIKLLYATPE-KVSAS---------------GRMISALQNLYERNLLARFVIDEA
HCV--SQWG------HDFRPDYKRLHELRQKF--PN-VPIMALTATAT----------PR
VQKDILNQMAMN----RPQVFTM--SFNRHNLKYSVL---PK-------------KPKKV
DE--ECINWIKKY-YPR----DSGIIYC-----LSRNDC--------DTM---------A
DSL---QRAGIAALA-YHAGLNDKDR-EYVQNKW----INQDR-CQ----VMCATIAFGM
GIDKPDVRYVIHASLPKS-VEGYYQESGRAG-RDGEISHCVLF-YSYSDVIRIKRIL---
--------AMDKDG--NHHSR--------ATHTNNLHSM--VHFCENVSE----------
CRRI---QLLAYFGE--NKFNADFCKEHPEV--ICDNC--AR----PHQYKAR--NVTED
VKKIVRFVQENCQ-----KVGGRYSKSSQQ--NRLTLNML-VD-IFIGAKSARIQT----
------GMFGHGAAYSRHNADRLFKKLVLDNVLMEDLYITNN-GQAV--AYISAGQKAMN
VLNGS--------------------MQVVFHETESASSIRKHKA---------SVIKNVS
K--RE--E
MVTKCLEELNELCKKLGKVFGL-HYYNIFSTSTLKTIA--ETLSADPEVLLQIDGVTEDK
LDKYGAELIELLQKYTE----------------WQLPVEEQVE--------SSGWIDTAR
DHLEDDV---YDDEEDS-------SSSYFRSSTGRGEKRK--KTPYSRKPKRRKSYNNQN
TYKSGNS---------GGWSSSKGGYKAAGRGAGRGAGRGSARPA---------------
----------------AAAAAPAARR----------------------------------

--PGVMALP-
--TPQT------------
---------AARPFLKPAFSHLG----------------
>H3DPJ8
--------------------------------MSTLPQNNLKEQL---------------

--ARHSNAAQSKLASTKPKTGPFC------------------------------------
---------FKKKSSSVTARVDIPTKVTEVFQ---------------------SRDVNVF
RNNLVTNSPLTFSNKLERSQTSKISTFFAVNSKTRSDSTSQI------------------
--------------------DNLASSGQISSATAKPDGSTSLDASLGFPEDNWDDL-DDF
EIPVKEPEEILGEKEQSSAETGAPGHSVQDVAAVSPGPDPVQ------------------
-------------EDCVLGDSPVRPSRRRRNVPKNRVLSESDED----------------
---N
ADEPCDEAKG-----------DVFLETKSLWIEPKVIELDDNSEPEDDFNFIP---PSPI
SDEM------------------------------------SSALSARFVHLIQSILAGPK

```
GQDSPPLQPHGPSVEDHEPLGSSAKAKTNEQLFS--------------------------
----------------------IMESICA-------------------------------
------------------------------------------LVDSVPEHELIALS----
---------------------CGNELLLKRAQR----------------------KRILA
TGGCSLFRMQLPDSTVVNEVDSKENPRFSTDAPGVR------------------------
-------------RSSSVFADCSSEHSDSI-----------IPAAPVSSKKSTTA-----
------------------------------------------------------------
----QADYERCDSPSAR------------------TVKNAHLEFSDAGADLGGL------
----------DLFFSPKRQETSLKNT----PRTSASEDA---------------------
--ERDDFYDDDFDI---DDLNDYDF---------PSYYEEPPPSPV--------------
--TSPNPRTLDRTIR--EGGPTTSPWEKKP---ATPPSA------------------PKP
SIVSSPEPTF---------------------RNPAH-----DRFRGF-----N-FPY---
------SQEMMKIFHKRFGLHQFRFNQLEAINATLGG--EDTFVL---MPT--GGGKSLC
--------YQLPA---------------------CV----SPGV-TVVISPLKSLIVDQI
QKLTT------LDI-----------------PATSLSGD---LGDREA---GRIYMQLSR
KEP--IVKLLYVTPE-KVSAS---------------NKLISALQNLYERGLLARFVIDEA
HCV--SQWG------HDFRPDYKKLHELRKKF--PQ-VPMMALTATAT----------PR
VQKDIHNQLNMR----QPQVFTM--SFNRTNLKYAVL---PK-------------KPKKV
DE--DCISWIKKH-YPR----DSGIIYC-----LSRNDC--------DTM---------A
ESL---QRAGLLALS-YHAGLRDGER-EYVQTKW----INQDG-CQ----VICATIAFGM
GIDKPDVRYVIHASLPKS-VEGYYQESGRAG-RDGEISQCILF-YSYTDVHRIKRII---
--------SMDREG--DGHTK--------ATHYNNLHSM--VHFCENAME-----------
CRRI---QLLAYFGE--LKFNRSFCKEHADV--SCDNC--AR----PNKYKLR--NVTDD
VKKIVRFVQENCE-----KVGSRFGKTAQQ--NRLTLNML-VD-IFLGTKSAKVQT----
------GMYGMGGAYSRHNADRLFKRLVLDNILMEDLYITNG-GQAV--CYISAGARATS
VLAGH--------------------MQVEFYETESTSSIRKHKA---------AVAKNVS
Q------------------------------------------------------RE--E
KVKECLEELTSLCKQLGKAFGI-HYYNMFSTSTLKKIA--EKLSCEPEELLQIDGVTEDK
LEKYGAEVIQVLQKYSE----------------WKLPVDDPTD------NDGDGWIDTRA
GRTNY-----GEEDDT--------ESTYFSSRPVQGQKRK--KAPFFKYSKR-KAYGNTG
YNSKGRG----------YGTSKSRSSSAAGRGDWGSAGEAAASRR---------------
------------------------------------------------------------
------------------------------------------------------------
------------------------------------------------------------
------------------------------------------------------------
------------------------------------------------------------
------------------------------------------------------------
------------------------------------------------------------
----------------------------------------------------PGFLSLP-
--------------------------------------------TPQS------------
---------HQRPFLKPSFSSF-----------------
>G3P9V9
--------------------------------MSCLPQNNLKEQL---------------
------------------------------------------------------------
--ARHSNAAQSKLSLAKPKPGAFS------------------------------------
----------FKKSSSTTPRVEVPT-----------KVIGANVLA--------NRNVNVS
KNNLVTKSHLTFPNKLERPQKSQISNFVPVNSKSN-------------------------
```

```
-----------------RAPAAVSSIPHNQFTGGNGSKSRSLDTSLGFPMDDWDDL-DDF
EIPVKNDSGMSANQ-----------VSLFNEERAQSSLTKPP------------------
------------------LSNCLENGNGLSRSEQSFMETDELEH----------------
---QVRNAAASPEQCLNNEP----------AEFELDVSPFILDDET--------------
-----------------------------KWTDPKVIQLYDNSDPEDDLDYIP---PSPI
PDEI--------------------------------SYTTSALETRSKSVDAKSRDSCVQ
SKGPIATLHASSDNHSNDNTIFSCDYVTDEQLFS--------------------------
----------------------IMQCICS-------------------------------
------------------------------------------LVDSIPEHEVIGLS----
---------------------CGSELLLKRAQR----------------------KRILA
TGGCNSLSMQQPDSTVISDCSHKIRSSFSCDTSSVMS-----------------------
-------------SSGSGPSDSKKPPQIRRSSIISVDDGCDHSDGITSTKPSYSR-----
----------------------------------------------------------KS
GADHATNQSICDSPSTH------------------RLTGPSFNSSKKSTTHADGS-----
----------DRLFSPKKPETVVQSKSNTPAASTATEDV---------------------
--ELDDFYNDYFDI---DDFNDSDV---------PSYFDEPS------------------
-----NSSTVASTAK--DLRSCESPWGNKP---------------------------TTP
APAPKPAKICSPEPTY---------------RNPAH-----DRFRGF-----N-FPH---
------SQEMMKIFHKRFGLHQFRFNQLEAINATILA--EDTFVL---MPT--GGGKSLC
--------YQLPA---------------------CV----SPGV-TVVISPLKSLIVDQV
QKLTT------LDI-----------------PATSLSGS---KSDSEA---GRIYMQLSR
KDP--IIKLLYVTPE-KVSAS---------------QKLISAMQNLYERGLLVRFVIDEA
HCV--SQWG------HDFRPDYKRLHELRQKF--PS-VPMMALTATAT----------PR
VQKDILNQLNMT----RPQVFTM--SFNRTNLKYAVL---PK-------------KPKKV
AE--DCIDWIKKH-YPR----DSGIIYC-----LSRNDC--------DAM---------A
EGL---QRAHISALS-YHAGLSDGDR-EYVQSKW----INQDG-CQ----VICATIAFGM
GIDKPDVRYVIHASLPKS-VEGYYQESGRAG-RDGEISHCILF-YSYTDVHRIKRII---
--------GMDKEG--DRHTK--------ATHFNNLHSM--VHFCENVME----------
CRRL---QLLAYFGE--LKFNKMFCKEHANV--SCDNC--AK----PNQYKMR--NVTED
VKKIVKFVQENCE-----KVGSRFGRTAQQ--NRLTLNML-VD-VFVGSKSAKVQT----
------GMFGMGGAYSRHNADRLFKKLVLDNILEEDLYITIN-GQAV--SYISAGPKAMN
VLSGH--------------------MQVEFYETESASSIRKHKA---------AVTTNVS
Q------------------------------------------------------RE--Q
KVQECLKELTDLCKQLGKAFGI-HYYNIFSTAALKKIAGTEKLSPDPEVLLQIDSVTEDK
LEKYGAEVIQVLEKYSE----------------WQLPEEPPET-------TGDGWIDTAQ
GRSYG-----DDDGDAE-------SSAYFRGQSAQGQKRK--KAPFFKYSKKRKASGNSK
GRGYSSN-------KSWSSSGSRGGSSAAGRGSRSSTGDFSASRK---------------
------------------------------------------------------------
------------------------------------------------------------
------------------------------------------------------------
------------------------------------------------------------
------------------------------------------------------------
------------------------------------------------------------
------------------------------------------------------------
----------------------------------------------------PGFLNVP-
--------------------------------------------APQT------------
---------NQRPFLKPTFSHLG----------------
```

>A0A0F8AI96
-------------------------------MSARLPQNNLQEQL---------------

--ARHSNAAPAKVSQAKPKAGAFS------------------------------------
---------FKKKSSSGTTKVE--------------------------------------
-----------VQTKLERPQKSKISTFFPVSSKGSSVRSAGNHAPAGQSTSAASAVKV--
-----------------------TTTPVHQFTSGTRSSSASLDASLGFPLDDWDDF-DDF
ETPVKAKNDSFSSEKSQKSTNPVSSPSEETTQGELIHNASRM------------------
---------------TPDLNISVGSKNGLSGSEPSCMETDEADG------AEASAAPSLN
QDSAECEFEDSPVKMTKRHA----------AARLKSVISDSEEDND-------------V
EFEPFKGMSD------------------NKNKGVEPKVKEFKSEDDDDLDYIP---PSPT
PDEISY---------------------------TPSALQTRSKTAGTERKESPVQPRELI
KTLHEPSDHHSKD-------------KTNEQLFS--------------------------
----------------------IMESICA-------------------------------
--LVDSVPEHELIALS----
---------------------CGNELLVKRAQR----------------------KRILA
VGGGCLPKIQQPDSTVISEPSVKEKSSLSCDTSNVMSSSCSLPMDLKK------------
--------PLQIRRSSVISIDHDSDHSDSI-----------ISGKPMHSEHSRTA-----

---HEENEIMCDSPSTH------------------SFTNSYFDSSKKSTTKMEDS-----
----------DLFFSPKKPETVVQNK----AKPSAAEDI---------------------
--EPDDFYLDDFDI---DDFNDSDI---------PDYFEKPS------------------
-------EQGQRSTTVKEGGPSKSSWEKKP---TTPASK------------------PKP
STICSPEPNF---------------------RNPAH-----DRFRGF-----E-FSY---
------SQEMMKIFHKRFGLHQFRFNQLEAINATLQG--EDTFIL---MPT--GGGKSLC
--------YQLPA---------------------CV----SPGV-TVVISPLKSLIVDQV
QKLTT------LDI-----------------PATSLSGD---KSDSEA---GRIYMQLSR
KDP--IIKLLYVTPE-KVSAS---------------NRLISALQNLYERGLLGRFVIDEA
HCV--SQWG------HDFRPDYKKLHELRQKF--PN-VPMMALTATAT----------PR
VQKDILNQLNMT----RPQVFTM--SFNRTNLKYSVM---PK-------------KPKKV
DE--DCMSWIKKH-YPR----DSGIIYC-----LSRNDC--------DAM---------A
ESL---QRAGILALS-YHAGLSDGDR-EYVQSKW----INQDG-CQ----VICATIAFGM
GIDKPDVRYVIHASLPKS-VEGYYQESGRAG-RDGEISHCILF-YSYTDVQRIKRII---
--------NMDREG--DRHTK--------ATHFNNLHSM--VHFCENVME----------
CRRI---QLLAYFGE--LKFNKSFCKDHADV--TCDNC--AK----PNQYKLR--NVSED
VKKIVRFVQENCE-----KVGARFGRTAQQ--NRLTLHML-VD-IFVGSKSARVQT----
------GMFGMGGAYSKHNADRLFKKLVLDNVLVEDLYITNG-GQAV--SYISAGPKAMN
VLSGH--------------------MQVEFYETESASSIRKHK----------AVAKNVS
Q--RE--E
KVQECLKELTDLCKQLGKAFGL-HYFNIFSTATLKKIA--EKLSPDPEVLLQIDGVTEDK
LEKYGAEVIQVLEKYSQ----------------WQLPAEEQVD------NGGGKWIDTTR
GRTWE-----DDEDNTE-------SSTYFHNQAAQGQKRK--KAPFFKYSKKRKGYNNTS
SSSKGRG-----------YSSNKSWSSSNSRGGSKAAGRGSRSSA---------------
------------------GDAPAGRR----------------------------------


```
------------------------------------------------------------
------------------------------------------------------------
------------------------------------------------------------
----------------------------------------------------PGFLTVP-
--------------------------------------------TPQT------------
---------NQRPFLKPAFSHLS----------------
>A0A1A7XSC7
--------------------------------MSSLPQNNLKEQL---------------
------------------------------------------------------------
--ARHNNAAQSKLSLARPKTGPFM------------------------------------
---------FKKKSTSSTTKVEFPT-----------KVISSNVLA--------NRNVNLA
QSGLVTEHSLTFSNKLERSQKSKINSFFPVSSKCKSDSTVSA------------------
----------GNPSSSCRTPLEKSDGKRSPVPTGDRISSTDLDVSVFPV-DDWDDF-DDF
E--SPVKTKNDSFSSDKSGKVTKLPSSADEEFTEKQSSDALL------------------
EKSGLNKDEDQICTKTDEIEESVNKNAASPETSLFQEPSEFEVE----------------
---------DSPVKRTRRRC----------PPPHVTSVLSDSDEDVCAMF---------E
TSVDKTSGKK-------------------KWLDPKVIELNDDSEPEGDLDYIP---PSPV
PDETSD---------------------------TVSTLEPRLQSGDSESRDVPLLVNSLA
TSLPKHSDDHSKD-------------KTKDQLFS--------------------------
----------------------LMESICS-------------------------------
------------------------------------------LVDSIPEHELIALS----
---------------------CGNELLLKRAQR----------------------KRILA
SGADSLFRMQQPDSTVL----------SEPSFKETSSSCSTTNSFVPF------------
-------------DSKKLLQPRRSSVISVD-----------SDIDLSDRTNLKPW-----
-------------------------------------------------------NNKHS
RTISVKNESICDSPSAH------------------SLTKPTFRHFEETTYESDP------
----------DLSFSPKKSEAVLQN-----CPKPHTAAA---------------------
EADVDDFYNDDFDI---DDFNDSDI---------PDYFDEPSTSSVS-------------
--SQNLSSAVSTRAT--GGGPNKSTWEIKP---------------------------VTP
VSAPKPAKIYSPEPTF---------------KNPAH-----DRFRGF-----N-FSY---
------SQEMMKLFHKRFGLHQFRFNQLEAINATLQG--EDTFVL---MPT--GGGKSLC
--------YQLPA---------------------CV----SPGV-TVVVSPLKSLIVDQI
QKLTA------LDI-----------------PATSLSGD---KSDSEA---ARIYMQLSR
KDP--IVKLLYATPE-KVSAS---------------NRLISALQNLYERGLLARFVIDEA
HCV--SQWG------HDFRPDYKKLHELRQKF--PK-VPMMALTATAT----------PR
VQKDILSQLNMT----RPLVFTM--SFNRVNLKYAVL---PK-------------KPKKV
DE--DCISWIKKH-YPR----DSGIVYC-----LSRNDC--------DAM---------A
ESL---QRAGILALS-YHAGLRDGER-EHVQSKW----INQDG-CQ----VICATIAFGM
GIDKPDVRYVIHASLPKS-VEGYYQESGRAG-RDGEVSHCILF-YSYADVHRIKRII---
--------SMDREG--DRHTK--------TTHYNNLHSM--VHFCEDVME----------
CRRI---QLLAYFGE--LKFNRNFCKEHSDV--SCDNC--AK----PNQYKMR--DVTDD
VKKIVRFVQENCE-----KVGARFGKTAQQ--NRLTLNML-VD-IFIGSKSAKVQT----
------GMFGMGAAYSRHNADRLFKKLVIDNVLAEDLYVTNH-GQTV--VYISAGPKAMN
ILSGH--------------------MQVEFCETESASSIRKHK----------AVTKSIS
K------------------------------------------------------RE--Q
MVQECLTELIDRCKQLGKAFGL-HYYNIFSTATMKKIA--ERLSSDPEVLLQIDGVTEDK
LEKYGAEVIQVLQKYSE----------------WQLPEEEQVA------TSGNEWIDTAH
```

GRSH------DDGDDLE-------SSSYFCNQTAQGQKRK--KAPFFKYSKKRKGYGSSG
STARGRG------------YSSNRSWSSSSRGGAHSAGRGSGSSV---------------
--------------------GNVGRR----------------------------------

--PGFMSVP-
--TPQS------------
---------VQRPFLKPSFSHMS----------------
>A0A1A7XXN6

---MQLSR
KDP--IVKLLYATPE-KVSAS---------------NRLISALQNLYERGLLARFVIDEA
HCV--SQWG------HDFRPDYKKLHELRQKF--PK-VPMMALTATAT----------PR
VQKDILSQLNMT----RPLVFTM--SFNRVNLKYAVL---PK-------------KPKKV
DE--DCISWIKKH-YPR----DSGIVYC-----LSRNDC--------DAM---------A
ESL---QRAGILALS-YHAGLRDGER-EHVQSKW----INQDG-CQ----VICATIAFGM
GIDKPDVRYVIHASLPKS-VEGYYQESGRAG-RDGEVSHCILF-YSYADVHRIKRII---
--------SMDREG--DRHTK--------TTHYNNLHSM--VHFCEDVME----------
CRRI---QLLAYFGE--LKFNRNFCKEHSDV--SCDNC--AK----PNQYKMR--DVTDD

VKKIVRFVQENCE-----KVGARFGKTAQQ--NRLTLNML-VD-IFIGSKSAKVQT----
------GMFGMGAAYSRHNADRLFKKLVIDNVLAEDLYVTNH-GQTV--VYISAGPKAMN
ILSGH----M-------------Q---VEFCETESASSIRKHK----------AVTKSIS
K--RE--Q
MVQECLTELIDRCKQLGKAFGL-HYYNIFSTATMKKIA--ERLSSDPEVLLQIDGVTEDK
LEKYGAEVIQVLQKYSE----------------WQLPEEEQVA------TSGNEWIDTAH
GRSHD-----DGDDLE--------SSSYFCNQTAQGQKRK--KAPFFKYSKKRKGYGSSG
STARGRG------------YSSNRSWSSSSRGGAHSAGRGSGSSV---------------
--------------------GNVGRR----------------------------------

--PGFMSVP-
--TPQS------------
---------VQRPFLKPSFSHMS----------------
>A0A1A8HHU7
--------------------------------MSSLPQNNLKEQL---------------

--ARHNHVAQSKLSLAKPKTGSFL------------------------------------
---------FKKKSTSGTTNVEFPT-----------KVISSNVLA--------NRNVNLP
HSSLVTEPSLTFSNKLERSQKSKINSFFPVSSKCKSDSTASA------GNSSPSS-----
-----------------QTPLAKSDSKGSPAPPGDRIGSTDLDASVFPV-DDWDDF-ADF
E--SPVKTKNDSFSSDKSGKVTKLLSSADEEFTEKPSSAALL-------------EKSGL
NKDG-DQICTKTDETGESVNKKAASPEPSLFQEPSEFEV---------------------
--------EDSPVKRTRRRC----------PPPHVTSVLSDSDEDVDAMF---------E
TSEDKTNEKK-------------------NWLDPKVIELNDDSEPEDDLDYIP---PSPV
SDETSY---------------------------AVSAVDTRIKSEDSPKGVPLLENSSAT
SLPKPSDHHSKDK--------------TKDQLFS-------------------------
----------------------LMESICS-------------------------------
--LVDSIPEHELIALS----
---------------------CGDELLLKRAQR----------------------KRILA
SGADSLFRMQQPDSTVL----------SEPGFKETSSSCSTSNSSVPF------------
-------------DSKKLPQPRRSSVIYVG-----------SDTDFSDRTDLNSL-----
---NKKHS
RTISVENESICDSPSAH------------------SLTKPTFSHFEQTTYENDP------
----------DLYFSPKRSEAVLQNS----TKPQTAAAE---------------------
-ADLDDFYNDDFDI---DDFNDSDI---------PDYFDEPATSS---------------
--VSSQNLSSAVSTRGTGGGPNKSTWENKP---VTPVSA------------------PKP
AKIYSPEPTV---------------------KNPAH-----DRFRGF-----N-FPY---
------SQEMMKIFHKRFGLHQFRFNQLEAINATLQG--EDTFVL---MPT--GGGKSLC
--------YQLPA---------------------CV----SAGV-TVVVSPLKSLIVDQI
QKLTA------LDI-----------------PATSVSGD---KSDSEA---ARIYMQLSR
KDP--TVKLLYATPE-KVSAS---------------NRLISALQNLYERGLLARFVIDEA
HCV--SQWG------HDFRPDYKKLHELRQKF--PK-VPMMALTATAT----------PR

VQKDILSQLNMT----RPLVFTM--SFNRVNLKYAVL---PK-------------KPKKV
DE--DCIDWIKKH-YPR----DSGIVYC-----LSRNDC--------DAM---------A
ESL---QRAGILALS-YHAGLGDAER-EHVQSKW----INQEG-CQ----VICATIAFGM
GIDKPDVRYVIHASLPKS-VEGYYQESGRAG-RDGEISHCILF-YSYTDVHRIKRII---
--------SMDKEG--DRHTK--------TTHYNNLHSM--VHFCEDVME----------
CRRI---QLLAYFGE--LNFNRNFCKEHPDV--SCDNC--AK----PNQYKMR--NVTED
VKKIVRFVQENCE-----KVGARFGKTAQQ--NRLTLNML-VD-IFIGSKSAKVQT----
------GMFGMGAAYSRHNADRLFKKLVIDHVLAEDLYVTNH-GQTV--VYISAGPKAMN
ILSGH----M-------------Q---VEFCETESASSIRKHK----------AVTKSVS
K--RE--Q
MVQECLMELSDLCKQLGKAFGL-HYYNIFSTATMKKIA--ERLSSDPEVLLQIDGVTEDK
LEKYGAEVIQVLQKYSE----------------WQLPEEQVAS-------SGNEWIDMSE
GRS-------HDDGDDL-------ESSYFCNQPAQGQKRK--KAPFFKYSKKRKGYGSSG
SSARGRG------------YSSNKSWSSNSRGGAQSAGRGSGSSV---------------
--------------------GNAGRR----------------------------------

--PGFMSVP-
--TPQS------------
---------VQRPFLKPSFSHMS----------------
>A0A1A8MH17
--------------------------------MSSLPQNNLKEQL---------------

--ARHN--HVAQSKLS--LAKPKT--------GSFL------------------------
---------FKKKSTFGTTNVEFPT-----------KVTSSNALA--------NRNVNLP
HSSLVTEPSLTFSNKLERSQKSKINSFFPISSKCKSDSTASA------GNPSPSS-----
-----------------QTPLAKSDSKRSPAPPGDRIGSTDLDASVFPV-DDWDDF-ADF
E--SPVKTKNDSFSSDKSGKITKLLSSADEEFTEKQSSGALL-------------EKSGL
NKDG-DQISTKTDETGESVNKKVASPEPSLFQEPSEFEV---------------------
--------EDSPVKRTRRRC----------PPPHVMSVLSDSDEDVCTMF---------E
TSEDKTNEKK-------------------KWLDPKVVELNDDSEPEDDLDYIP---PSPV
SDETSY---------------------------TVSAVDTRIKSEDSHKGVPLLDNSSAT
SLPKPSDHHSKDK--------------TKDQLFS--------------------------
----------------------LMESICS-------------------------------
--LVDSIPEHELIALS----
---------------------CGNELLLKRAQR----------------------KRILA
TGADSLFGMQQPDSTVL----------SEPGFKETSSSCSTSNSSVPF------------
-------------DSKKLPQPRRSSVISVG-----------SDTDFSDRTDLKPL-----
---NKMHS
RTISVENESICDSPSAH------------------SLTKPTFSHFEPSTYESDP------
----------DLDFSPKRSEAVLQNS----TKPQTAAAE---------------------
-ADLDDFYNDDFDI---DDFNDSDI---------PDYFDEPATSSVS-------------
--SQNLSSAVSTRGT--GGGPNKSTWENKP---VTPVSA------------------PKP

AKIYSPEPTF---------------------KNPAH-----DRFRGF-----N-FPY---
------SQEMMKIFHKRFGLHQFRFNQLEAINATLQG--EDTFVL---MPT--GGGKSLC
--------YQLPA---------------------CV----SAGV-TVVVSPLKSLIVDQI
QKLTA------LDI-----------------PATSLSGD---KSDSEA---ARIYMQLSR
KDP--IVKLLYATPE-KVSAS---------------NRLISALQNLYERGLLARFVIDEA
HCV--SQWG------HDFRPDYKKLHELRQKF--PK-VPMMALTATAT----------PR
VQKDILSQLNMT----RPLVFTM--SFNRVNLKYAVL---PK-------------KPKKV
DE--DCIDWIMKH-YPR----DSGIVYC-----LSRNDC--------DAM---------A
ESL---QRAGILALS-YHAGLRDGER-EHVQSKW----INQDG-CQ----VICATIAFGM
GIDKPDVRYVIHASLPKS-VEGYYQESGRAG-RDGEVSHCILF-YSYTDVHRIKRII---
--------SMDKEG--DRHTK--------TTHYNNLHSM--VHFCEDVME----------
CRRI---QLLAYFGE--LNFNRNFCKEHPDV--SCDNC--AK----PSQYKMR--DVTED
VKKIVRFVQENCE-----KVGARFGKTAQQ--NRLTLNML-VD-IFIGSKSAKVQT----
------GMFGMGAAYSRHNADRLFKKLVIDHVLAEDLYVTNH-GQTV--VYISAGPKAMN
ILSGH----M-------------Q---VEFCETESASSIRKHK----------AVTKSVS
K--RE--Q
MVQECLMELIDLCKQLGKAFGL-HYYNIFSTATMKKIA--ERLSSDPEVLLQIDGVTEDK
LEKYGAEVIQVLQKYSE----------------WQLPEEHVAS-------SGNEWIDTSQ
GRSHD-----DRDDLE--------SSSYFCNPPARGQKRK--KAPFFKYSKKRKGYGSSG
SSARGRG------------YSSNKPWSSNSRGGAQSAGRGSGSSV---------------
--------------------GNAGRR----------------------------------

--PGFMSVP-
--TPQS------------
---------VQRPFLKPSFSHMS----------------
>A0A1A8P9C1

--HF
E---

--PSTY
ESDP--

--DLDFSPKRSEAVLQ----

---NSTKPQTAAAEADL-----

----DDFYNDDFDI---DDFNDSDI---------PDYFDEPATSSVS-------------
--SQNLSSAVSTRGT--GGGPNKSTWENKP---VTPVSA------------------PKP
AKIYSPEPTF---------------------KNPAH-----DRFRGF-----N-FPY---
------SQEMMKIFHKRFGLHQFRFNQLEAINATLQG--EDTFVL---MPT--GGGKSLC
--------YQLPA---------------------CV----SAGV-TVVVSPLKSLIVDQI
QKLTA------LDI-----------------PATSLSGD---KSDSEA---ARIYMQLSR
KDP--IVKLLYATPE-KVSAS---------------NRLISALQNLYERGLLARFVIDEA
HCV--SQWG------HDFRPDYKKLHELRQKF--PK-VPMMALTATAT----------PR
VQKDILSQLNMT----RPLVFTM--SFNRVNLKYAVL---PK-------------KPKKV
DE--DCIDWIMKH-YPR----DSGIVYC-----LSRNDC--------DAM---------A
ESL---QRAGILALS-YHAGLRDGER-EHVQSKW----INQDG-CQ----VICATIAF--

---------VDKEG--DRHTK--------TTHYNNLHSM--VHFCEDVME----------
CRRI---QLLAYFGE--LNFNRNFCKEHPDV--SCDNC--AK----PSQYKMR--DVTED
VKKIVRFVQENCE-----KVGARFGKTAQQ--NRLTLNML-VD-IFIGSKSAKVQT----
------GMFGMGAAYSRHNADRLFKKLVIDHVLAEDLYVTNH-GQTV--VYISAGPKAMN
ILSGH----M-------------Q---VEFCETESASSIRKHK----------AVTKSVS
K--RE--Q
MVQECLMELIDLCKQLGKAFGL-HYYNIFSTATMKKIA--ERLSSDPEVLLQIDGVTEDK
LEKYGAEVIQVLQKYSE----------------WQLPEEHVAS-------SGNEWIDTSQ
GRSHD-----DRDDLE--------SSSYFCNPPARGQKRK--KAPFFKYSKKRKGYGSSG
SSARGRG------------YSSNKPWSSNSRGGAQSAGRGSGSSV---------------
--------------------GNAGRR----------------------------------

--PGFMSVP-
--TPQS------------
---------VQRPFLKPSFSHMS----------------
>A0A1A8B9K8
--------------------------------MSSLPQNNLKEQL---------------

--ARHNHVAQSKLSLAKPKTGPFL------------------------------------
---------FKKKSTSGTTNVEFPT-----------KVISSNVLA--------NRNVNLP
RSSLVTEPSLTFSNKLERSQKSKINNFFPVSSKCKSDSTASA------GNSSPSS-----
-----------------QTPLAKSDSKRSPAPPGDRIGSTDLGASVFPV-DDWDDF-GDF
E--SPVKTKNDSFSSDKSGKITKLLSSANEEFTEKQSSGALL-------------EKSGL
NKDG-DQICTKTDETGESVNKKVASPEPSLFQEPSEFEV---------------------
--------EDSPVKRTRRRC----------PPPHVTSVLSDSDEDVGTMF---------E
TSEDKTNEKK-------------------KWLDPKVIELNDDSEPEDDLDYIP---PSPV

SDETSY---------------------------TVSAVDTRIKSEDSHKGVPLLDNSSAT
SLPKPSDHHSKDK--------------TKDQLFS--------------------------
----------------------LMESICS-------------------------------
--LVDSIPEHELIALS----
---------------------CGNELLLKRAQR----------------------KRILA
TGADSLFRMQQPDSTVL----------SEPGFKETSPSCSTSNSSVPF------------
-------------DSKTLPQPRRSSVISVG-----------SDTDFSDRTDLKPL-----
---NKKHN
RTISVENESICDSPSAH------------------SLTKPTFSHFEQTTYESDP------
----------DLDFSPKRSEAVLQNS----TKPQTAAAE---------------------
-ADLDDFYNDDFGI---DDFNDSDI---------PDYFDEPATSSVS-------------
--SQNLSSAVSTRGT--GGGPNKSTWENKP---VTPVSA------------------PKP
AKIYSPEPTF---------------------KNPAH-----NRFRGF-----N-FPY---
------SQEMMKIFHKRFGLHQFRFNQLEAINATLQG--EDTFVL---MPT--GGGKSLC
--------YQLPA---------------------CV----SAGV-TVVVSPLKSLIVDQI
QKLTA------LDI-----------------PATSLSGD---KSDSEA---ARIYMQLSR
KDP--IVKLLYATPE-KVSAS---------------NRLISALQNLYERGLLARFVIDEA
HCV--SQWG------HDFRPDYKKLHELRQKF--PK-VPMMALTATAT----------PR
VQKDILSQLNMT----RPLVFTM--SFNRVNLKYAVL---PK-------------KPKKV
DE--DCIDWIKKH-YPR----DSGIVYC-----LSRNDC--------DAM---------A
ESL---QRAGILALS-YHAGLRDGER-EHVQSKW----INQDG-CQ----VICATIAFGM
GIDKPDVRYVIHASLPKS-VEGYYQESGRAG-RDGEVSHCILF-YSYTDVHRIKRII---
--------SMDKEG--DRHTK--------TTHYNNLHSM--VHFCEDVME----------
CRRI---QLLAYFGE--LNFNRNFCKEHPDV--SCDNC--AK----PSQYKMR--DVTED
VKKIVRFVQENCE-----KVGARFGKTAQQ--NRLTLNML-VD-IFIGSKSAKVQT----
------GMFGMGAAYSRHNADRLFKKLVIDHVLAEDLYVTNH-GQTV--VYISAGPKAMN
ILSGH----M-------------Q---VEFCETESASSIRKHK----------AVTKSVS
K--RE--Q
MVQECLMELIDLCKQLGKAFGL-HYYNIFSTATMKKIA--ERLSSDPEVLLQIDGVTEDK
LEKYGAEVIQVLQKYSE----------------WQLPEEQVAS-------SGNEWIDTSQ
GRSHD-----DRDDLE--------SSSYFCNQPARGQKRK--KAPFFKYSKKRKGYSSSG
SSARGRG------------YSSNKSWSSNSRGGAQSAGRGSGSSV---------------
--------------------GNAGRR----------------------------------

--PGFMSVP-
--TPQS------------
---------VQRPFLKPSFSHMS----------------
>A0A1A8V9I1
--------------------------------MSSLPQNNLKEQL---------------

--ARHNHVAQSKLSLAKPKTGPFL------------------------------------
---------FKKKSTSGTTNVEFPT-----------KVISSNVLA--------NRNVNLP

RSSLVTEPSLTFSNKLERSQKSKINNFFPVSSKCKSDSTASA------GNSSPSS-----
-----------------QTPLAKSDSKRSPAPPGDRIGSTDLGASVFPV-DDWDDF-GDF
E--SPVKTKNDSFS----SDKSGKITKLLSSANEEFTEKQSS--------GALLEKSGLN
KDGD-------------QICTKTDETGESVNKKAASPEPSLFQE----------------
--PSEFEVEDSPVKRTRRRC----------PPPHVTSVLSDSDEDVGTMF---------E
TSEDKTNEKK-------------------KWLDPKVIELNDDSEPEDDLDYIP---PSPV
SDETSY---------------------------TVSAVDTRIKSEDSHKGVPLLDNSSAT
SLPKPSDHHSKDK--------------TKDQLFS--------------------------
----------------------LMESICS-------------------------------
--LVDSIPEHELIALS----
---------------------CGNELLLKRAQR----------------------KRILA
TGADSLFRMQQPDSTVL----------SEPGFKETSPSCSTSNSSVPF------------
-------------DSKTLPQPRRSSVISVG-----------SDTDFSDRTDLKPL-----
---NKKHN
RTISVENESICDSPSAH------------------SLTKPTFSHFEQTTYESDP------
----------DLDFSPKRSEAVLQNS----TKPQTAAAE---------------------
-ADLDDFYNDDFDI---DDFNDSDI---------PDYFDEPATSSVS-------------
--SQNLSSAVSTRGT--GGGPNKSTWENKP---VTPVSA------------------PKP
AKIYSPEPTF---------------------KNPAH-----NRFRGF-----N-FPY---
------SQEMMKIFHKRFGLHQFRFNQLEAINATLQG--EDTFVL---MPT--GGGKSLC
--------YQLPA---------------------CV----SAGV-TVVVSPLKSLIVDQI
QKLTA------LDI-----------------PATSLSGD---KSDSEA---ARIYMQLSR
KDP--IVKLLYATPE-KVSAS---------------NRLISALQNLYERGLLARFVIDEA
HCV--SQWG------HDFRPDYKKLHELRQKF--PK-VPMMALTATAT----------PR
VQKDILSQLNMT----RPLVFTM--SFNRVNLKYAVL---PK-------------KPKKV
DE--DCIDWIKKH-YPR----DSGIVYC-----LSRNDC--------DAM---------A
ESL---QRAGILALS-YHAGLRDGER-EHVQSKW----INQDG-CQ----VICATIAFGM
GIDKPDVRYVIHASLPKS-VEGYYQESGRAG-RDGEVSHCILF-YSYTDVHRIKRII---
--------SMDKEG--DRHTK--------TTHYNNLHSM--VHFCEDVME----------
CRRI---QLLAYFGE--LNFNRNFCKEHPDV--SCDNC--AK----PSQYKMR--DVTED
VKKIVRFVQENCE-----KVGARFGKTAQQ--NRLTLNML-VD-IFIGSKSAKVQT----
------GMFGMGAAYSRHNADRLFKKLVIDHVLAEDLYVTNH-GQTV--VYISAGPKAMN
ILSGH----M-------------Q---VEFCETESASSIRKHK----------AVTKSVS
K--RE--Q
MVQECLMELIDLCKQLGKAFGL-HYYNIFSTATMKKIA--ERLSSDPEVLLQIDGVTEDK
LEKYGAEVIQVLQKYSE----------------WQLPEEQVAS-------SGNEWIDTSQ
GRSHD-----DRDDLE--------SSSYFCNQPARGQKRK--KAPFFKYSKKRKGYSSSG
SSARGRG------------YSSNKSWSSNSRGGAQSAGRGSGSSV---------------
--------------------GNAGRR----------------------------------

--PGFMSVP-
--TPQS------------

```
---------VQRPFLKPSFSHMS----------------
>A0A1A8RAG5
------------------------------------------------------------
------------------------------------------------------------
------------------------------------------------------------
------------------------------------------------------------
------------------------------------------------------------
------------------------------------------------------------
------------------------------------------------------------
------------------------------------------------------------
------------------------------------------------------------
------------------------------------------------------------
------------------------------------------------------------
------------------------------------------------------------
------------------------------------------------------------
------------------------------------------------------------
------------------------------------------------------------
------------------------------------------------------------
---------------------------------------------LSSAVSTRGT-----
------------------------------------------------------------
------------------------------------------------------------
------------------------------------------------------------
------------------------------------------------------------
-----------------GGGPNKSTWENKP---VTPVSA------------------PKP
AKIYSPEPTF---------------------KNPAH-----DRFRGF-----N-FPY---
------SQEMMKIFHKRFGLHQFRFNQLEAINATLQG--EDTFVL---MPT--GGGKSLC
--------YQLPA---------------------CV----SAGV-TVVVSPLKSLIVDQI
QKLTA------LDI-----------------PATSLSGD---KSDSEA---ARIYMQLSR
KDP--IVKLLYATPE-KVSAS---------------NRLISALQNLYERGLLARFVIDEA
HCV--SQWG------HDFRPDYKKLHELRQKF--PK-VPMMALTATAT----------PR
VQKDILSQLNMT----RPLVFTM--SFNRVNLKYAVL---PK-------------KPKKV
DE--DCIDWIKKH-YPR----DSGIVYC-----LSRNDC--------DAM---------A
ESL---QRAGILALS-YHAGLRDGER-EHVQSKW----INQDG-CQ----VICATIAFGM
GIDKPDVRYVIHASLPKS-VEGYYQESGRAG-RDGEVSHCILF-YSYTDVHRIKRII---
--------SMDKEG--DRHTK--------TTHYNNLHSM--VHFCEDVME----------
CRRI---QLLAYFGE--LNFNRNFCKEHPDV--SCDNC--AK----PSQYKMR--DVTED
VKKIVRFVQENCE-----KVGARFGKTAQQ--NRLTLNML-VD-IFIGSKSAKVQT----
------GMFGMGAAYSRHNADRLFKKLVIDHVLAEDLYVTNH-GQTV--VYISAGPKAMN
ILSGH----M-------------Q---VEFCETESASSIRKHK----------AVTKSVS
K------------------------------------------------------RE--Q
MVQECLMELTDLCKQLGKAFGL-HYYNIFSTATMKKIA--ERLSSDPEVLLQIDGVTEDK
LEKYGAEVIQVLQKYSE----------------WQLPEEQVAS-------SGNEWIDTSQ
GRSHD-----DRDDLE--------SSSYFCNPPTRGQKRK--KAPFFKYSKKRKGYGSSG
SSARGRG------------YSSNKPWSSNSRGGAQSAGRGSGSSV---------------
--------------------GNAGRR----------------------------------
------------------------------------------------------------
------------------------------------------------------------
```


--PGFMSVP-
--TPQS------------
---------VQRPFLKPSFSHMS----------------
>A0A1A8E874

----------------SQTPLAKSDSKRSPAPPGDRIGSTDLGASVFPV-DDWDDF-GDF
E--SPVKTKNDSFSSDKSGKITKLLSSANEEFTEKQSSGALL-------------EKSGL
NKDG-DQICTKTDETGESVNKKAASPEPSLFQEPSEFEV---------------------
--------EDSPVKRTRRRC----------PPPHVTSVLSDSDEDVGTMI---------E
TSEDKTNEKK-------------------KWLDPKVIELNDDSEPEDDLDYIP---PSPV
SDETSY---------------------------TVSAVDTRIKSEDSHKSVPLLDNSSAT
SLPKPSDHHSKDK--------------TKDQLFS--------------------------
----------------------LMESICS-------------------------------
--LVDSIPEHELIALS----
---------------------CGNELLLKRAQR----------------------KRILA
TGADSLFRMQQPDSTVL----------SEPGFKETSPSCSTSNSSVPF------------
-------------DSKTLPQPRRSSVISVG-----------SDTDFSDRTDLKPL-----
---NKKHS
RTISVENESICDSPSAH------------------SLTKPTFSHFEQTTYESDPDL----
----------DFSPKRSEAVLQNSIK----PQTAAAEAD---------------------
---LDDFYNDDFDI---DDFNDSDI---------PDYFDEPATSSVS-------------
--SQNLSSAVSTRGT--GGGPNKSTWENKP---VTPVSA------------------PKP
AKIYSPEPTF---------------------KNPAH-----NRFRGF-----N-FPY---
------SQEMMKIFHKRFGLHQFRFNQLEAINATLQG--EDTFVL---MPT--GGGKSLC
--------YQLPA---------------------CV----SAGV-TVVVSPLKSLIVDQI
QKLTA------LDI-----------------PATSLSGD---KSDSEA---ARIYMQLSR
KDP--IVKLLYATPE-KVSAS---------------NRLISALQNLYERGLLARFVIDEA
HCV--SQWG------HDFRPDYKKLHELRQKF--PK-VPMMALTATAT----------PR
VQKDILSQLNMT----RPLVFTM--SFNRVNLKYAVL---PK-------------KPKKV
DE--DCIDWIKKH-YPR----DSGIVYC-----LSRNDC--------DAM---------A
ESL---QRAGILALS-YHAGLRDGER-EHVQSKW----INQDG-CQ----VICATIAFGM
GIDKPDVRYVIHASLPKS-VEGYYQESGRAG-RDGEVSHCILF-YSYTDVHRIKRII---
--------SMDKEG--DRHTK--------TTHYNNLHSM--VHFCEDVME----------
CRRI---QLLAYFGE--LNFNRNFCKEHPDV--SCDNC--AK----PSQYKMR--DVTED
VKKIVRFVQENCE-----KVGARFGKTAQQ--NRLTLNML-VD-IFIGSKSAKVQT----
------GMFGMGAAYSRHNADRLFKKLVIDHVLAEDLYVTNH-GQTV--VYISAGPKAMN
ILSGH----M-------------Q---VEFCETESASSIRKHK----------AVTKSVS
K--RE--Q
MVQECLVELIDLCKQLGKAFGL-HYYNIFSTATMKKIA--ERLSSDPEVLLQIDGVTEDK

```
LEKYGAEVIQVLQKYSE----------------WQLPEEQVAS-------SGNEWIDTSQ
GRSHD-----DRDDLE--------SSSYFCNQPARGQKRK--KAPFFKYSKKRKGYSSSG
SSARGRG------------YSSNKSWSSNSRGGAQSAGRGSGSSV---------------
--------------------GNAGRR----------------------------------
------------------------------------------------------------
------------------------------------------------------------
------------------------------------------------------------
------------------------------------------------------------
------------------------------------------------------------
------------------------------------------------------------
----------------------------------------------------LGFMSVP-
--------------------------------------------TPQS------------
---------VQRPFLKPSFSHMS----------------
>A0A1A8D0H8
--------------------------------MSSLPQNNLKEQL---------------
------------------------------------------------------------
--ARHNHVAQSKLSLAKPKTGPFL------------------------------------
---------FKKKSTSGTTNVEFPT-----------KVISSNVLA--------NRNVNLP
RSSLVTEPSLTFSNKLERSQKSKINNFFPVSSKCKSDSTASA------GNSSPSS-----
-----------------QTPLAKSDSKRSPAPPGDRIGSTDLGASVFPV-DDWDDF-GDF
E--SPVKTKNDSFSSDKSGKITKLLSSANEEFTEKQSSGALL-------------EKSGL
NKDG-DQICTKTDETGESVNKKAASPEPSLFQEPSEFEV---------------------
--------EDSPVKRTRRRC----------PPPHVTSVLSDSDEDVGTMI---------E
TSEDKTNEKK-------------------KWLDPKVIELNDDSEPEDDLDYIP---PSPV
SDETSY---------------------------TVSAVDTRIKSEDSHKSVPLLDNSSAT
SLPKPSDHHSKDK--------------TKDQLFS--------------------------
----------------------LMESICS-------------------------------
------------------------------------------LVDSIPEHELIALS----
---------------------CGNELLLKRAQR----------------------KRILA
TGADSLFRMQQPDSTVL----------SEPGFKETSPSCSTSNSSVPF------------
-------------DSKTLPQPRRSSVISVG-----------SDTDFSDRTDLKPL-----
-------------------------------------------------------NKKHS
RTISVENESICDSPSAH------------------SLTKPTFSHFEQTTYESDP------
----------DLDFSPKRSEAVLQNS----TKPQTAAAE---------------------
-ADLDDFYNDDFDI---DDFNDSDI---------PDYFDEPATSSVS-------------
--SQNLSSAVSTRGT--GGGPNKSTWENKP---VTPVSA------------------PKP
AKIYSPEPTF---------------------KNPAH-----NRFRGF-----N-FPY---
------SQEMMKIFHKRFGLHQFRFNQLEAINATLQG--EDTFVL---MPT--GGGKSLC
--------YQLPA---------------------CV----SAGV-TVVVSPLKSLIVDQI
QKLTA------LDI-----------------PATSLSGD---KSDSEA---ARIYMQLSR
KDP--IVKLLYATPE-KVSAS---------------NRLISALQNLYERGLLARFVIDEA
HCV--SQWG------HDFRPDYKKLHELRQKF--PK-VPMMALTATAT----------PR
VQKDILSQLNMT----RPLVFTM--SFNRVNLKYAVL---PK-------------KPKKV
DE--DCIDWIKKH-YPR----DSGIVYC-----LSRNDC--------DAM---------A
ESL---QRAGILALS-YHAGLRDGER-EHVQSKW----INQDG-CQ----VICATIAFGM
GIDKPDVRYVIHASLPKS-VEGYYQESGRAG-RDGEVSHCILF-YSYTDVHRIKRII---
--------SMDKEG--DRHTK--------TTHYNNLHSM--VHFCEDVME----------
```

CRRI---QLLAYFGE--LNFNRNFCKEHPDV--SCDNC--AK----PSQYKMR--DVTED
VKKIVRFVQENCE--------------------------------KVGARFGKTAQ----
--------------QNRLTL--

>A0A1A8R1X9

--FVIDEA

HCV--SQWG------HDFRPDYKKLHELRQKF--PK-VPMMALTATAT----------PR
VQKDILSQLNMT----RPLVFTM--SFNRVNLKYAVL---PK-------------KPKKV
DE--DCIDWIKKH-YPR----DSGIVYC-----LSRNDC--------DAM---------A
ESL---QRAGILALS-YHAGLRDGER-EHVQSKW----INQDG-CQ----VICATIAFGM
GIDKPDVRYVIHASLPKS-VEGYYQESGRAG-RDGEVSHCILF-YSYTDVHRIKRII---
--------SMDKEG--DRHTK--------TTHYNNLHSM--VHFCEDVME----------
CRRI---QLLAYFGE--LNFNRNFCKEHPDV--SCDNC--AK----PSQYKMR--DVTED
VKKIVRFVQENCE-----KVGARFGKTAQQ--NRLTLNML-VD-IFIGSKSAKVQT----
------GMFGMGAAYSRHNADRLFKKLVIDHVLAEDLYVTNH-GQTV--VYISAGPKAMN
ILSGH----M-------------Q---VEFCETESASSIRKHK----------AVTKSVS
K--RE--Q
MVQECLMELIDLCKQLGKAFGL-HYYNIFSTATMKKIA--ERLSSDPEVLLQIDGVTEDK
LEKYGAEVIQVLQKYSE----------------WQLPEEQVAS-------SGNEWIDTSQ
GRSHD-----DRDDL---------ESSYFCNPPARGQKRK--KAPFFKYSKKRKGYGSSG
SSARGRG------------YSSNKPWSSNSRGGAQSAGRGSGSSV---------------
--------------------GNAGRR----------------------------------

--PGFMSVP-
--TPQS------------
---------VQRPFLKPSFSHMS----------------
>A0A1A8JGA9
--------------------------------MSSLPQNNLKEQL---------------

--ARHNHVAQSKLSLAKPKTGPFL------------------------------------
---------FKKKSTSGTTNVEFPT-----------KVISSNVLA--------NRNVNLP
RSSLVTEPSLTFSNKLERSQKSKINNFFPVSSKCKSDSTASA------GNSSPSS-----
-----------------QTPLAKSDSKRSPAPPGDRIGSTDLNASVFPV-DDWDDF-GDF
E--SPVKTKNDSFSSDKSGKITKLLSSANEEFTEKQSSGALL-------------EKSGL
NKDG-DQICTKTDETGESVNKKAASPEPSLFQEPSEFEV---------------------
--------EDSPVKRTRRRC----------PPPHVTSVLSDSDEDVGTIF---------E
TSEDKTNEKK-------------------KWLDPKVIELNDDSEPEDDLDYIP---PSPV
SDETSY---------------------------TVSAVDTRIKSEDSHKGVPLLDNSSAT
SLPKPSDHHSKDK--------------TKDQLFS--------------------------
----------------------LMESICS-------------------------------
--LVDSIPEHELIALS----
---------------------CGNELLLKRAQR----------------------KRILA
TGADSLFRMQQPDSTVL----------SEPGFKETSSSCSTSNSSVPF------------
-------------DSKTLPQPRRSSVISVG-----------SDTDFSDRTDLKPL-----
---NKKHS
RTISVENESICDSPSAH------------------SLTKPTFSHFEQTTYESDP------
----------DLDFSPKRSEAVLQNS----TKPQTAAAE---------------------
-ADLDDFYNDDFDI---DDFNDSDI--------PDYFDEPATSSVS-------------

--SQNLSSAVSTRGT--GGGPNKSTWENKP---VTPVSA------------------PKP
AKIYSPEPTF---------------------KNPAH-----DRFRGF-----N-FPY---
------SQEMMKIFHKRFGLHQFRFNQLEAINATLQG--EDTFVL---MPT--GGGKSLC
--------YQLPA---------------------CV----SAGV-TVVVSPLKSLIVDQI
QKLTA------LDV-----------------PATSLSGD---KSDSEA---ARIYMQLSR
KDP--IVKLLYATPE-KVSAS---------------NRLISALQNLYERGLLARFVIDEA
HCV--SQWG------HDFRPDYKKLHELRQKF--PK-VPMMALTATAT----------PR
VQKDILSQLNMT----RPLVFTM--SFNRVNLKYAVL---PK-------------KPKKV
DE--DCIDWIKKH-YPR----DSGIVYC-----LSRNDC--------DAM---------A
ESL---QRAGILALS-YHAGLRDGER-EHVQSKW----INQDG-CQ----VICATIAFGM
GIDKPDVRYVIHASLPKS-VEGYYQESGRAG-RDGEVSHCILF-YSYTDVHRIKRII---
--------SMDKEG--DRHTK--------TTHYNNLHSM--VHFCEDVME----------
CRRI---QLLAYFGE--LNFNRNFCKEHPDV--SCDNC--AK----PSQYKMR--DVTED
VKKIVRFVQENCE-----KVGARFGKTAQQ--NRLTLNML-VD-IFIGSKSAKVQT----
------GMFGMGAAYSRHNADRLFKKLVIDHVLAEDLYITNH-GQTV--VYISAGPKAMN
ILSGH----M-------------Q---VEFCETESASSIRKHK----------AVTKSVS
K--RE--Q
MVQECLMELTDLCKQLGKAFGL-HYYNIFSTATMKKIA--ERLSSDPEVLLQIDGVTEDK
LEKYGAEVIQVLQKYSE----------------WQLPEEQVAS-------SGNEWIDTSQ
GRSHD-----DRDDLE--------SSSYFCNQPARGQKRK--KAPFFKYSKKRKGYGSSG
SSARGRG------------YSSNKSWSSNSRGGAQSAGRGSGSSV---------------
--------------------GNAGRR----------------------------------

--LGFMSVP-
--TPQS------------
---------AQRPFLKPSFSHMS----------------
>A0A1A8JZ51

----------------DFRPDYKKLHELRQKF--PK-VPMMALTATAT----------PR
VQKDILSQLNMT----RPLVFTM--SFNRVNLKYAVL---PK-------------KPKKV
DE--DCIDWIKKH-YPR----DSGIVYC-----LSRNDC--------DAM---------A
ESL---QRAGILALS-YHAGLRDGER-EHVQSKW----INQDG-CQ----VICATIAFGM
GIDKPDVRYVIHASLPKS-VEGYYQESGRAG-RDGEVSHCILF-YSYTDVHRIKRII---
--------SMDKEG--DRHTK--------TTHYNNLHSM--VHFCEDVME----------
CRRI---QLLAYFGE--LNFNRNFCKEHPDV--SCDNC--AK----PSQYKMR--DVTED
VKKIVRFVQENCE-----KVGARFGKTAQQ--NRLTLNML-VD-IFIGSKSAKVQT----
------GMFGMGAAYSRHNADRLFKKLVIDHVLAEDLYITNH-GQTV--VYISAGPKAMN
ILSGH----M-------------Q---VEFCETESASSIRKHK----------AVTKSVS
K--RE--Q
MVQECLMELTDLCKQLGKAFGL-HYYNIFSTATMKKIA--ERLSSDPEVLLQIDGVTEDK
LEKYGAEVIQVLQKYSE----------------WQLPEEQVAS-------SGNEWIDTSQ
GRSHD-----DRDDLE--------SSSYFCNQPARGQKRK--KAPFFKYSKKRKGYGSSG
SSARGRG------------YSSNKSWSSNSRGGAQSAGRGSGSSV---------------
--------------------GNAGRR----------------------------------

--LGFMSVP-
--TPQS------------
---------AQRPFLKPSFSHMS----------------
>A4UTP8
--------------------------------MSSLPQNNLEEQL---------------

--ARHNNANQSKLSLAKPKTGAFL------------------------------------
---------FKKKSTSGTTNVEYPA-----------KVTASSVLA--------NRNVNVP
KNSLLTKSSVTFSNKLERTQKSKINSFFTVNSKCQSDSVIPA------------------
-----------CRNPPAVSGNNGAITQTKTDNHSINATNAPSFDPSQFPLDDWDDF-DDF
ETPVKTKNDSFSSGKVTKPSSPDEEFPEFTGKQSSDTSHVTP------------------
----------------ELIIEDDQPCMEIKEPEHIVSNNSTVSP-----------GPGIL
QETVQFEVEDSPVRGTRRRP------------FEHKSVLSDSEDDT--------------

---------------NTVVEKIETKSDGKKWIDPKVIELQDNSEPEVDFDCIP---PSPD
TDET-----------------------------SYTSSALQTRSKTGKTQSRFFSAQSKS
SAPTRPEFSLCLS-----------KNSTREQLYS--------------------------
----------------------IMESICA-------------------------------
--LVDSIPEHELISLS----
---------------------CGNELLLRRAHR----------------------KRILA
TGGDFSFRMQQPDSTVI----------SDASFKETSSCCVLTPSSSAVL-----------
-------------DSRKSQQPRRPSVISLD-----------YDSDHSDRTAFNPL-----
---SSKSS
RAICVEDESICDSLSTPR-----------------LLKQPCFSESTSRLDEDD-------
----------AHFFSPKKPVSVEQNK----SKTPTCTTA--------------------D
NTETDDFYFDDFDI---DDFDESDI---------PEYFEEAPTSSVP-------------
----RQNSSTATAVK--EGGPSKSSWDKKP---SSSVST------------------PKP
PKLCSPEPTF---------------------RNPAH-----DRFRGF-----N-FPH---
------SQEMMKIFHKRFGLHQFRFNQLEAINAALLG--EDAFVL---MPT--GGGKSLC
--------YQLPA---------------------CV----SPGV-TVVVSPLKSLIVDQI
QKLTT------LDI-----------------PATSLSGD---KSDSEA---GRIYMQLSR
KDP--LIKLLYVTPE-KVSAS---------------GRLISALQNLYERGLLARFIIDEA
HCV--SQWG------HDFRPDFKRLHELRQKF--PS-VRMMALTATAT----------PR
VQKDILNQLNMM----RPQVFTM--SFNRSNLKYSVL---PK-------------KPKKV
DE--DCIGWIKKH-YPR----DSGIVYC-----LSRNDC--------DAM---------A
ESL---KRAGIQALS-YHAGLSDGDR-EYVQSKW----INQDG-CQ----VICATIAFGM
GIDKPDVRYVIHASLPKS-VEGYYQESGRAG-RDGEISHCILF-YSYTDVHRIKRII---
--------SMDREG--DSHTK--------ATHFNNLHSM--VHFCENVME----------
CRRI---QLLAYFGE--LNFNRNFCKDHPDV--SCDNC--AK----PNQYQMK--NVTED
VKKIVRFVQENCE-----KVGSRFGKTAQQ--NRLTLNML-VD-IFIGSKSAKIQT----
------GMFGMGGAYSRHNADRLFKKLVLENILVEDLYITNN-GQAV--SYISAGPKAMN
VLSGY--------------------MQVDFYETESASSIRKQKA---------AVSKSVS
Q--RD--E
MVQACLKELVDLCKELGKAFGL-HYYNIFSTATLKKIA--ERLSSDPEVLLQIDGVTEDK
LEKYGAEVIKVLQKYSE----------------RQLPVEEQAE------GAGEGWIDTTR
GRTQD-----EDDE----------SSTYFSNRSAQGQKRK--KAPFFKYSKKRKGFGNAS
SNSRGNS---------------KSWSSSSSRGSSQAAGRGSRNSA---------------
------------------GDPPAGRR----------------------------------

--PGILAAP-
--TPQS------------
---------KQRPFLKPSFSHLS----------------
>I3KDC9
--------------------------------MSSLPQNNLKEQL---------------

--ARHSNAAQSKQSVAKPKPGAFS------------------------------------

```
---------FKKKSSSGTTKVEVPT-----------KVISSNALA--------NRNVNVP
KSSLVTKSPLTFSNKLERPQKSKINNYFSVSSKCKPDTISPADIASPASQTPSVVSDI--
------------------KSAPAPAKSNSQSCSSNSFNSTALDVSRSFPMDNWDDL-DDF
ETPAKGKNNSFSSEVSEKSSKLLPSPTGEKAESTGKLNYDSL------------FKTSKL
TSKNGKQTCMETDEPEENVNTTAVSPGPSLNQEPADWSDEEELA----------------
-------------------------------MKMNRKHPAHLKHIM-------------S
DSEEDNNAEL--------EPFKGGTGNKKIWIDPKTIEIDDNSEPEDNLDCIP---PSPI
PDEITYTG-------------------------STLETRAKTADAEIGDNAVKSKSPVTL
HEPSELCLKD-----KTSECCALKKKKKNEKLFA--------------------------
----------------------IMESICA-------------------------------
------------------------------------------LVDSVPEHELIALS----
---------------------CGDELLLKRAQR---------------------CVSFVC
SFGIIHGVLSNGVSSNSSVPV---------------------------------------
-------------DSKKPPQHRRSSVISVD-----------YDSDDSDSVIDLKP-----
------------------------------------------------------LDNKDS
GAIGVETDSICDSPSAH------------------RLTKPSFNISEKSSIDLDGS-----
----------DLFFSPKESESGVQKK----SNNTPTFVA-------------------AE
DEEMDDFYNDDFDI---DDFNDSDI---------PDYFDEPQ------------------
-----TSSAVTTAVK--EGGPSKSSWEKKP---TTPTSA------------------PKP
STICSPGKSTNLRHLDCLHITKPTF------KNPAH-----DRFRGF-----N-FPH---
------SQEMMKIFHKRFGLHQFRFNQLEAINAALLG--EDAFVL---MPT--GGGKSLC
--------YQLPA---------------------CV----SLGV-TVVVSPLKSLIVDQV
QKLTT------LDI-----------------PATSLSGD---KSDSEA---SRIYMQLSR
KDP--IIKLLYVTPEKKLSAS---------------NRLISALQNLYERGLLARFIIDEA
HCV--SQWG------HDFRPDYKKLHELRQKF--PN-VAMMALTATAT----------PR
VQKDILNQLNMS----RPQVFTM--SFNRTNLKYAVL---PK-------------KPKKV
DE--DCTSWIKKH-YPR----DSGIVYC-----LSRNDC--------DAM---------A
ESL---QRAGLSALS-YHAGLSDSDR-EYVQSKW----INQDG-CQ----VICATIAFGM
GIDKPDVRYVIHASLPKS-MEGYYQESGRAG-RDGEISHCILF-YSYTDVQRIKRII---
--------SMDREG--DRHTK--------ATHYNNLHSM--VHFCENVME----------
CRRI---QLLAYFGE--LKFNRNFCKDHPDV--SCDNC--TK----PNQYKMR--NVTED
VKKIVRFVQENCE-----KVGARFGKTANQ--NRLTLNML-VD-IFIGSKAAKVQT----
------GMFGIGAAYSRHNADRLFKKLVLDNVLVEDLYITNG-GQAV--SYVSAGTKAMN
VLSGH--------------------MQVEFYETESASTIRKHKA---------GTAKNVS
R------------------------------------------------------RE--E
MVQECLKELLDLCKQLGKAFGL-HYYNIFSTATLKKIA--EKLSSDPEVLLQIDGVTEDK
LDKYGAEVIQVLQKYSE----------------WQLPEEQADS-------GGDGWIDTAR
GRTRG-----NDEDDTE-------SSTYFRNKSAQGQKRK--KAPFFKYSKKKKGCGNTS
SSSKGSV------EFSRGYSSNKSWSSSSSRGGGRGSRSSAGDAS---------------
----------------------ATRR----------------------------------
------------------------------------------------------------
------------------------------------------------------------
------------------------------------------------------------
------------------------------------------------------------
------------------------------------------------------------
------------------------------------------------------------
----------------------------------------------------PGLLAAP-
```

--MPQS------------
---------SQRPFLKPVFSHFS----------------
>A0A146W5V2
--------------------------------MSGLPQNNLKEQL---------------

--ARHNNAAQSKLSLAKPKTGAFL------------------------------------
---------FKKKSSSGTTRVEYPT-----------KVNSPNGLA--------NRNVNVP
TNSLVTKSPVTFSNKLERTQKSKINSFFPPSSKCASDFTSPAGNSPSARQSPPVEAGV--
-----------------RMTAAPAEPDNQPSCGGKGIGSPSLDVSSFPM-DDWDDF-EDF
E--TPAKSKNDSVCSEKSARVAMAMSSSDEFPEFRGKKSPGP--------VRLQAESKKK
SSDP-------------SCMEMEESRVGKTVSPEPSLAHEPSEE----------------
------EVEDSPVKRTRRRC----------PPPHVTSVLSDSDDEVA------------V
ASEPLKDKTG----------------EKGKWVDPKVIDLNDSSEPEEDVDYIP---PSPP
PDAT--LYTTSPAETRTQSG
KVQSRDNTFQPNGPATTLPSTHLSSDKTKEQLFN--------------------------
----------------------AMEAICG-------------------------------
--LVDSIPEHELIGLS----
---------------------CGNELLLKRAQR----------------------KRILA
CGAESLSRMMQQPDSTVISDASFIDTFSSCG-----------------------------
-------------ASSLSPSGSLPVDSKKN-----------LPPQRSSLNYHSDC-----
------------------FDGSSLN----------------------------PQHRTDS
KTIYAESESVCDSPSPP------------------SFTKPAYNFSDKTSSHADEA-----
----------DLFFSPKKSKDVEQDK----SKAPASAAA--------------------A
DFEADDFYDDDFDI---DDLNDSDI---------PDYFDEPPTSSML-------------
-----SQNRSTAPAAVKEGGPNKSSWDKKP---VTPVSA------------------PKP
QKIYSPEPNF---------------------KNPAH-----DRYRGF-----N-FPH---
------SQEMMKIFHKRFGLHQFRFNQLEAINASLLG--EDAFVL---MPT--GGGKSLC
--------YQLPA---------------------CI----SAGV-TVVISPLKSLIVDQI
QKLTT------LDI-----------------PATSLSGD---KSDSEA---GRIYMQLSR
KDP--IIKLLYVTPE-KVSAS---------------NRLISALQNLYERGLLARFVIDEA
HCV--SQWG------HDFRPDYKKLHELRHKF--PN-VRMMALTATAN----------PR
VQKDILNQLNMS----RPQVFTM--SFNRANLKYSVL---PK-------------KPKKV
DE--DCIGWIKKH-YPR----DSGIVYC-----LSRNEC--------DAL---------A
ESL---KRAGIQALA-YHAGLSDGDR-EYVQSKW----INQDD-CQ----VICATIAFGM
GIDKPDVRYVIHATLPKS-VEGYYQESGRAG-RDGEISHCILF-YSYNDVHRIKRII---
--------SMDREG--DRHTK--------ATHYNNLHSM--VHFCENMMD----------
CRRI---QLLAYFGE--LTFNRNFCKDHPDV--SCDNC--AR----PNQYKLR--KVTDD
VKKIVRFVQENCE-----KVGARFGKTPQQ--NRLTLNML-VD-IFIGAKSAKVQT----
------GMYGMGAAYSRHNADRLFKKLVLDNVLVEDLYITVN-GQAV--SYISAGPKATN
ILSGH--------------------MEVEFYETESASSVRKQK----------AXXXXXX
X--XXXXX
XXXXXXXXXXXXXXXXXXXXXXXXXXXXXXXXXXXXXX----------------------
---------------------------------XXLSPRASPR-----------------
------------------------ERPWFSSV----------------------------

>A0A146ZYQ1
--------------------------------MSGLPQNNLKEQL---------------

--ARHN--NAAQSKLS--LAKPKT--------GAFL------------------------
---------FKKKSSSGTTRVEYPT-----------KVNSPNGLA--------NRNVNVP
TNSLVTKSPVTFSNKLERTQKSKINSFFPPSSKCTSDFTSPAGNSPSTRESPPAEAGV--
-----------------RMTAAPAEPDNQPSCGGKGIGSPSLDVSSFPM-DDWDDF-DDF
E--TPAKSKNDSVCSEKSARVAMAMSSSDEFPEFRGKKSPGP------------------
VRLQ-AESKKKSSDQSCMETEESRVGKTVSPEPSLAHEHSEEEV----------------
--------EDSPVKRTRRRC----------PPPHVTSVLSDSDDEVA------------V
ASEPLKDKTG--------EKGKWV--------DPKVIDLNDSSEPEEDVDYIP---PSPP
PDAT--SYTTSPAETRTQFG
KVQSRDNTFQPNGPATTLPSTHLSSDKTKEQLFS--------------------------
----------------------AMEAICA-------------------------------
--LVDSIPEHELIGLS----
---------------------CGNELLLKRAQR----------------------KRILA
CGAESSSRMMQQPDSTVISDASFIDTFSSCGAS---------------------------
---------------SLSPSGSL--DSKRN-----------LPPQRSSLNYHSDC-----
------------------FDGSSLN----------------------------PRHRTDS
KTIYAESESVCDSPSPP------------------SFTKPAYNSSEKTSSHADDA-----
----------DLFFSPKKSKDVEHDK----SKAPASAAA--------------------A
DFEADDFYDDDFDI---DDLNDSDI---------PDYFDEPPTSSML-------------
-----SQNRSTAPAAMKEGGPNKSSWDKKP---VTPVSA------------------PKP
QKIYSPEPNF---------------------KNPAH-----DRYRGF-----N-FPH---
------SQEMMKIFHKRFGLHQFRFNQLEAINASLLG--EDAFVL---MPT--GGGKSLC
--------YQLPA---------------------CI----SAGV-TVVISPLKSLIVDQI
QKLTT------LDI-----------------PATSLSGD---KSDSEA---GRIYMQLSR
KDP--IIKLLYVTPE-KVSAS---------------NRLISALQNLYERGLLARFVIDEA
HCV--SQWG------HDFRPDYKKLHELRHKF--PN-VPMMALTATAN----------PR
VQKDILNQLNMS----RPQVFTM--SFNRANLKYSVL---PK-------------KPKKV
DE--DCIGWIKKH-YPR----DSGIVYC-----LSRNEC--------DAL---------A
ESL---KRAGIQALA-YHAGLSDGDR-EYVQSKW----INQDD-CQ----VICATIAFGM
GIDKPDVRYVIHATLPKS-VEGYYQESGRAG-RDGEISHCILF-YSYNDVHRIKRII---
--------SMDREG--DRHTK--------ATHYNNLHSM--VHFCENMMD----------
CRRI---QLLAYFGE--LTFNRNYCKDHPDV--SCDNC--AR----PNQYKLR--KVTDD
VKKIVRFVQENCE-----KVGARFGKTPQQ--NRLTLNML-VD-IFIGAKSAKVQT----
------GMYGMGAAYSRHNADRLFKKLVLDNVLVEDLYITVN-GQAV--SYISAGPKATN
ILSGH----M-------------E---VEFYETESASSVRKQKA---------SVSKSVS
K--RE--A

```
MVQQCLKELIDLCKQLGKAFGI-HYYNIFSTATVKKIA--ERLSSDPEVLLQIDGVTEDK
LEKYGAEVIKVLQKYSE----------------WQLPVDEQTE------STGDGWIDPRC
----------PEDDDTE-------SSTYFSNQSAAGRKRK--KAPFFKYSKKRKGYGNQG
YSSRGRG------------YSNNKSSSSGSRGGSQSAGRGSASSA---------------
------------------GAAPGGR-----------------------------------
------------------------------------------------------------
------------------------------------------------------------
------------------------------------------------------------
------------------------------------------------------------
------------------------------------------------------------
------------------------------------------------------------
----------------------------------------------------PGFMSVP-
--------------------------------------------MPQS------------
---------NQRPFLKSSFSYMS----------------
>A0A146W2U0
--------------------------------MSGLPQNNLKEQL---------------
------------------------------------------------------------
--ARHN--NAAQSKLS--LAKPKT--------GAFL------------------------
---------FKKKSSSGTTRVEYPT-----------KVNSPNGLA--------NRNVNVP
TNSLVTKSPVTFSNKLERTQKSKINSFFPPSSKCASDFTSPAGNSPSARQSPPVEAGV--
-----------------RMTAAPAEPDNQPSCGGKGIGSPSLDVSSFPM-DDWDDF-EDF
E--TPAKSKNDSVCSEKSARVAMAMSSSDEFPEFRGKKSPGP------------------
VRLQ-AESKKKSGDQSCMEMEESRVGKTVSPEPSLAHEPSEEEV----------------
--------EDSPVKRTRRRC----------PPPHVTSVLSDSDDEVA------------V
ASEPLKDKTG--------EKGKWV--------DPKVIDLNDSSEPEEDVDYIP---PSPP
PDAT------------------------------------------SYTTSPAETRTQFG
KVQSRDNTFQPNGPATTLPSTHLSSDKTKEQLFS--------------------------
----------------------AMEAICALPEEDVDYIPPSPPPDATLYTTSPAETRTQS
GKVQSRDNTFQPNGPATTLPSTHLSSDKTKEQLFNAMEAICGLVDSIPEHELIGLS----
---------------------CGNELLLKRAQR----------------------KRILA
CGAESLSRMMQQPDSTVISDASFIDTFSSCGAS---------------------------
---------------SLSPSGSLPVDSKKN-----------LPPQRSSLNYHSDC-----
------------------FDGSSLN----------------------------PQHRTDS
KTIYAESESVCDSPSPP------------------SFTKPAYNFSDKTSSHADEA-----
----------DLFFSPKKSKDVEQDK----SKAPASAAA--------------------A
DFEADDFYDDDFDI---DDLNDSDI---------PDYFDEPPTSSML-------------
-----SQNRSTAPAAVKEGGPNKSSWDKKP---VTPVSA------------------PKP
QKIYSPEPNF---------------------KNPAH-----DRYRGF-----N-FPH---
------SQEMMKIFHKRFGLHQFRFNQLEAINASLLG--EDAFVL---MPT--GGGKSLC
--------YQLPA---------------------CI----TAGV-TVVISPLKSLIVDQI
QKLTT------LDI-----------------PATSLSGD---KSDSEA---GRIYMQLSR
KDP--IIKLLYVTPE-KVSAS---------------NRLISALQNLYERGLLARFVIDEA
HCV--SQWG------HDFRPDYKKLHELRHKF--PN-VRMMALTATAN----------PR
VQKDILNQLNMS----RPQVFTM--SFNRANLKYSVL---PK-------------KPKKV
DE--DCIGWIKKH-YPR----DSGIVYC-----LSRNEC--------DAL---------A
ESL---KRAGIQALA-YHAGLSDGDR-EYVQSKW----INQDD-CQ----VICATIAFGM
GIDKPDVRYVIHATLPKS-VEGYYQESGRAG-RDGEISHCILF-YSYNDVHRIKRII---
```

--------SMDREG--DRHTK--------ATHYNNLHSM--VHFCENMMD----------
CRRI---QLLAYFGE--LTFNRNFCKDHPDV--SCDNC--AR----PNQYKLR--KVTDD
VKKIVRFVQENCE-----KVGARFGKTPQQ--NRLTLNML-VD-IFIGAKSAKVQT----
------GMYGMGAAYSRHNADRLFKKLVLDNVLVEDLYITVN-GQAV--SYISAGPKATN
ILSGH----M-------------E---VEFYETESASSVRKQKA---------SVSKSVS
K--RE--A
MVQQCLKELIDLCKQLGKAFGI-HYYNIFSTATVKKIA--ERLSSDPEVLLQIDGVTEDK
LEKYGAEVIKVLQKYSE----------------WQLPVDEQTE------STGDGWIDPRC
----------PEDDDTE-------SSTYFSNQSAAGRKRK--KAPFFKYSKKRKGYGNQG
YSSRGRG------------YSNNKSSSSGSRGGSQSAGRGSASSA---------------
------------------GAAPGGRR----------------------------------

--PGFMSVP-
--MPQS------------
---------NQRPFLKS----------------------
>E7EZY7
--------------------------------MSSLPQNNLKEHL---------------

--ERHN--NAAQNKLS--LLKPKP--------GGFC------------------------
---------FKKKSSSGISKV---------------------------------------
-----------EVPQKPERVAPKVNFFTAPSKPKTNTVNPLA------NPFALNKTPLVQ
STIKALPKSAA-----------------LPKDSTNDQTVELKHDISQLSFNEWDDL-DDF
E--TPVKSRVASPVAGTSTKKPSVSDQNTSSSCSSKCEETKV------------------
------------NEESQATETITAPKDVLSAANGVSTETAEREP----------------
--------EDSPIKKSKRPK----------KSVQHTALLSDTEDEEI------------I
HCVSPDTNQKNFKSVAVAEKDKWGEPN-----VIDSDDCEENNHYEGFEDFIP---PSPI
PEEI------------------------------------SLSVSDKEKSSSEPVTPANK
KESSRSASGLPAPLDQSAKGLKGA----DDALFS--------------------------
----------------------VMESICC-------------------------------
--LVDTIPEHELIALT----
---------------------CGTELLLQRAHR----------------------KRILA
KGGSSRTSHSDSVSTPG----------FLNRPTFGVTPSNLTSLTPVT--SGKREGVKTG
FSFRKSIASVMSVGDESVFEDSDCIINGVE-----------TPGGTWNPNSSTKI-----
---SAG
RDTFNGSIQTLSKPES-------------------KTDKCYSRLSFNESSNQT-------
----------DLFYSPKRVDSGSRNA----DSSVEINIA-------------GSSSLRTG
AEPVDDFLLDDFDI---DDFDENDI---------PDYYEEPPSVLE--------------
--SRNNSGVKTPSVQ--EGGSSKSFERKTFTP-------------------------PAP
KSIKTPNPEPLY-------------------RNPAH-----DRFRGF-----N-FPH---
------SPEMMKIFHKKFGLHQFRFNQLEAINASLLG--EDTFVL---MPT--GGGKSLC
--------YQLPA---------------------CV----SAGV-TVVISPLRSLIVDQV
QKLTT------LDI-----------------CATSLSGD---KKDSEA---ARIYMQLSR

KDP--AIKLLYATPE-KVCAS---------------GRMISALQNLYERGLLARLVIDEA
HCV--SQWG------HDFRPDYKRLHELRRMF--PN-VPIMALTATAT----------PR
VQKDILNQLAMT----RPQVFTM--SFNRNNLKYSVL---PK-------------KPKKV
DE--ECIQWIKKY-YPR----DSGIVYC-----LSRNDC--------DTL---------A
DSL---QRAGIAALA-YHAGLSDSDR-EYVQNKW----INQDG-CQ----VMCATIAFGM
GIDKPDVRYVIHASLPKS-VEGYYQESGRAG-RDGEISHCVLF-YSYSDVIRIKRLI---
--------AMDKDG--NQQSK--------ATHINNLHSM--VHFCENVAE----------
CRRI---QLLAYFGE--HTFNTSFCKEHPEV--ICDNC--AR----PNKYKSR--NVTDD
VKKIARFVQDNCE-----KVGNRYGKSAQQ--NRLTLNML-VE-IFLGSKSARIQT----
------GMFGVGAAYSKHNAERLFKKLVLDNVLMEDLYITNS-GQAV--AYISAGPKAMS
VLNGC----M-------------Q---VEFHETESASSIRKHRA---------SVCEKVS
K--RE--E
MVKKCLGELNDLCKKLGKVFGI-HYYNIFSTATLKKIA--ETLSADPEVLLQIDGVTEDK
LEKYGAEFIELLQKYSE----------------WQLPAEAQAE--------SSGWIDTTR
GHQND-----EDDNDDDDEGGGDVTSTYFRSNSGRGAKRK--QGSYSRKPKRRKGSSGQN
SSAKGGYSSNWSSSRGGGRGGGYRGGSRGAGRGSRSAPSGSAAKR---------------

--PGFMSLP-
--TPQA------------
---------AARPFLKPSFSHL-----------------
>E9QIQ5
--------------------------------MSSLPQNNLKEHL---------------

--ERHN--NAAQNKLS--LLKPKP--------GGFC------------------------
---------FKKKSSSGISKVEVPQ-----------KVTGSSVLA--------NRSVNIP
SNFEVTKNPVTFSQKPERVA-PKVNFFTAPSKPKTNTVNPLA------NPFALNKTPLVQ
STIKALPKSAA-----------------LPKDSTNDQTVELKHDISQLSFNEWDDL-DDF
E--TPVKSRVASPVAGTSTKKPSVSDQNTSSSCSSKCEETKV------------------
------------NEESQATETITAPKDVLSAANGVSTETAEREP----------------
--------EDSPIKKSKRPK----------KSVQHTALLSDTEDEEI------------I
HCVSPDTNQKNFKSVAVAEKDKWGEPN-----VIDSDDCEENNHYEGFEDFIP---PSPI
PEEI------------------------------------SLSVSDKEKSSSEPVTPANK
KESSRSASGLPAPLDQSAKGLKGA----DDALFS--------------------------
----------------------VMESICC-------------------------------
--LVDTIPEHELIALT----
---------------------CGTELLLQRAHR----------------------KRILA
KGGSSRTSHSDSVSTPG----------FLNRPTFGVTPSNLTSLTPVT--SGKREGVKTG
FSFRKSIASVMSVGDESVFEDSDCIINGVE-----------TPGGTWNPNSSTKI-----
---SAG
RDTFNGSIQTLSKPES-------------------KTDKCYSRLSFNESSNQT-------
----------DLFYSPKRVDSGSRNA----DSSVEINIA-------------GSSSLRTG

AEPVDDFLLDDFDI---DDFDENDI---------PDYYEEPPSVLE--------------
--SRNNSGVKTPSVQ--EGGSSKSFERKTFTP-------------------------PAP
KSIKTPNPEPLY-------------------RNPAH-----DRFRGF-----N-FPH---
------SPEMMKIFHKKFGLHQFRFNQLEAINASLLG--EDTFVL---MPT--GGGKSLC
--------YQLPA---------------------CV----SAGV-TVVISPLRSLIVDQV
QKLTT------LDI-----------------CATSLSGD---KKDSEA---ARIYMQLSR
KDP--AIKLLYATPE-KVCAS---------------GRMISALQNLYERGLLARLVIDEA
HCV--SQWG------HDFRPDYKRLHELRRMF--PN-VPIMALTATAT----------PR
VQKDI---

>A0A087XHE6
--------------------------------MSSLPQNNLKEQL---------------

--ARHN--NATHNKLS--LAKPKT--------GAFS------------------------
---------FKKKSSLGTTKVE--------------------------------------
-----------YPTKLERTQKSKINNFFSPSTNSKSNFSLAGISTSTCEEPPADTG----
-----------------GKVIAAPAKPNNQSSCGGKGIGSAGLDLSDLPVDDWDDF-EDF
E--TPAKTKNDSLCSEKSARVTETVSSSDEEFPEFTGKQSHD------------------
---------------------SGHLKSELSEKNREQSCMEKEEPESRIGKTTVSLEPSLV
DGLPDDEVEDTPVKRTRRRC----------PPPHVTSVLSDSDEEIS------------T
MSEPSKDKTD----------------EKGKWIDPKVINLNDNSEPEEDVDYIP---PSPP
PDET---------------------------------FYRPSSAETRRTHSDIVQSRDKL
FQSNSPATTLLEPSNHLSSD------KTKEQLFS--------------------------
----------------------IMESICT-------------------------------
--LVDSIPEHELIALT----

---------------------CGNELLLKRAHR----------------------KRILA
CGVDSLRMPQPDSTVISDTSYKETFSSYSASSLTSPSSSLFLDTKKPL------------
-----------------NSQRSSVISVDCD-----------SDYLDSASSKPWNK-----

-----REISICDSPSAQ------------------SFTKPHFSLSEKTNSDVDDTD----
----------HFFAKKSEQLKSKTPT-----------------------------HAITT
DFEEDDFDVDDFDI---DDFNDSDI---------PDYFDEPPTSST--------------
--WSENPNTTCAAVT--KGGPNKASWDKKP---LTPVSA------------------PKP
QKIYSPEPNF---------------------KNPAH-----DRFRGF-----N-FPH---
------SQEMMKIFHKRFGLHQFRFNQLEAINAALLG--EDAFVL---MPT--GGGKSLC
--------YQLPA---------------------CL----SPGV-TVVVSPLKSLIVDQI
QKLTT------LDI-----------------PATSLSGD---KSDSEA---GRIYMQLSR
KDP--IIKLLYVTPE-KVSAS---------------NRLISALQNLYERGLLARFVIDEA
HCV--SQWG------HDFRPDYKKLHELRQKF--PN-IPMMALTATAT----------PR
VQKDILNQLNMS----KPQVFTM--SFNRTNLKYAVL---PK-------------KPKKV
DE--DCISWIKKH-YPR----DSGIVYC-----LSRNDC--------DAL---------A
ESL---KRAGIQALS-YHAGLSDGNR-EYVQSKW----INQDG-CQ----VICATIAFGM
GIDKPDVRYVIHASLPKS-MEGYYQESGRAG-RDGEISHCILF-YSYTDVHRIKRII---
--------SMDREG--DKHAK--------TTHFNNLHSM--VHFCENVME----------
CRRI---QLLAYFGE--LKFNRNFCKDHPDV--SCDNC--AK----PNEYKLR--KVTDD
VNKIVRFVQENCE-----KVGARFGKTPQQ--NRLTLNML-VD-IFIGAKSAKIQT----
------GMFGMGAAYSRHNADRLFKKLVLDNVLVEDLYITNN-GQAV--SYISAGPKAMN
ILSGH----M-------------E---VEFYETESASSIRKQR----------AAVKSIS
K--RE--E
MVQNCLKELIDLCKQLGKAFGL-HYYNIFSTAALKKIS--EKLSSDPEVLLQIDGVTEDK
LEKYGAEVIKVLQKYSE----------------WQLPVDEQTE------SDGNGWIDTRR
T---------EDDDTE--------SSTYFSNQSARGQKRK--KAPFFKYSKKKAYGNQGS
SS--------------KGRGFGYNKSSSGSRGGSQSSGRGSTSSA---------------
----------------------AGKK----------------------------------

--LGFLSAP-
--TPQS------------
---------NQRPFLKPTYSHFS----------------
>A0A096LSR1
-----------------------------TTTMSSLPQNNLKEQL---------------

--ARHNNATHNKLSLAKPKTGAFS------------------------------------
---------FKKKSSLGTTKVEYPT-----------KVNSPNILA--------NRNVNVL
GNSLVTKSSLTFSNKLERTQKSKINNFFSPSTNSKSNFSLAGISTSTCEEPPADTG----
-----------------GKVIAAPAKPNNQSSCGGKGIGSAGLDLSDLPVDDWDDF-EDF
ETPAKTKNDSLCSEKSARVTETVSSSDEEFPEFTGKQSHDSG------------------
-----------------------HLKSELSEKNREQSCMEKEEPESRIGKTTVSLEPSLV

DGLPDDEVEDTPVKRTRRRC----------PPPHVTSVLSDSDEKG--------------
-----------------------------KWIDPKVINLNDNSEPEEDVDYIP---PSPP
PDET------------------------------FYRPSSAETRRTHSDIVQSRDKLFQS
NSPATTLLEPSNHLSSDKTIGFTSFIPAEEQLFS--------------------------
----------------------IMESICT-------------------------------
--LVDSIPEHELIALT----
---------------------CGNELLLKRAHR---------------------------
----FVHLYSAGDVLMSYLVESKVDSLRMPQPDSTVISDTSYKE----------------
-------------TFSSYSASSLTSPSSSL-----------FLDTKKPLNSQNSA-----
--SS
KPWNKREISICDSPSAQ------------------SFTKPHFSLSEKTNSDVDDTD----
----------HFFAKKSEQLKSKTPT-----------------------------HAITT
DFEEDDFDVDDFDI---DDFNDSDI---------PDYFDEPPTSST--------------
--WSENPNTTCAAVT--KGGPNKASWDKKP---------------------------LTP
VSAPKPQKIYSPGKAK---CNQYFKTQQPNFKNPAH-----DRFRGF-----N-FPH---
------SQEMMKIFHKRFGLHQFRFNQLEAINAALLG--EDAFVL---MPT--GGGKSLC
--------YQLPA---------------------CL----SPGV-TVVVSPLKSLIVDQI
QKLTT------LDI-----------------PATSLSGD---KSDSEA---GRIYMQLSR
KDP--IIKLLYVTPE-KVSAS---------------NRLISALQNLYERGLLARFVIDEA
HCV--SQWG------HDFRPDYKKLHELRQKF--PN-IPMMALTATAT----------PR
VQKDILNQLNMS----KPQVFTM--SFNRTNLKYAVL---PK-------------KPKKV
DE--DCISWIKKH-YPR----DSGIVYC-----LSRNDC--------DAL---------A
ESL---KRAGIQALS-YHAGLSDGNR-EYVQSKW----INQDG-CQ----VICATIAFGM
GIDKPDVRYVIHASLPKS-MEGYYQESGRAG-RDGEISHCILF-YSYTDVHRIKRII---
--------SMDREG--DKHAK--------TTHFNNLHSM--VHFCENVME----------
CRRI---QLLAYFGE--LKFNRNFCKDHPDV--SCDNC--AK----PNEYKLR--KVTDD
VNKIVRFVQENCE-----KVGARFGKTPQQ--NRLTLNML-VD-IFIGAKSAKIQT----
------GMFGMGAAYSRHNADRLFKKLVLDNVLVEDLYITNN-GQAV--SYISAGPKAMN
ILSGH--------------------MEVEFYETESASSIRKQR----------AAVKSIS
K--RE--E
MVQNCLKELIDLCKQLGKAFGL-HYYNIFSTAALKKIS--EKLSSDPEVLLQIDGVTEDK
LEKYGAEVIKVLQKYSE----------------WQLPVDEQTE------SDGNGWIDTRR
T---------EDDDTE--------SSTYFSNQSARGQKRK--KAPFFKYSKKKAYGNQGS
SS--------------KGCGCSRGFGYNKSSSGSRGGSQSSGRGS---------------
------------------TSSAAGKK----------------------------------

--LGFLSAP-
--TPQS------------
---------NQRPFLKPTYSHFS----------------
>M3ZL77
--------------------------------MSSLPQNNLKEQL---------------

```
--ARHNNAAHSKLSLAKPKTGAFS------------------------------------
---------FKKQSSSGTTKVE--------------------------------------
-----------YPTKLERTQKSKINNFFSPSTNSKSNFSLAGNSTSACQAPPADTG----
-----------------GKVIAAPAKPDNPSSCGGKGNSSTGLDLSDLPFDDWDDF-QDF
ETPTKTKNDSLCSEKSVRVTKTVSSSDEEFPEFTGKQSHGHL------------------
-------------------------KSELSKKNREQSCMEKEEPEPGVGKTAVSLEPSLV
DGLPDDEVEDTPVKRTRRRC----------PPPHVTSVLSDSDEEIS------------T
MSEPLKDKTD----------------EKAKWIDPKVIKLNDNSEPKEDVDYIP---DSPP
PDET---------------------------------FYRPSSAETRRTHSGIVQSRDKL
FQSNIPAATLLEP------SNNLSSDKTKEQLFS--------------------------
----------------------IMESICT-------------------------------
------------------------------------------LVDSIPEHELIALT----
---------------------CGNELLLKRAYR----------------------KRILA
CGVDSLRMPQPDSTVISDTSYKETFSSCSASSLTSPSSSLFLDTKKPL------------
-----------------NSQRLSVISVDYD-----------SDYLDSASLKPGNK-----
------------------------------------------------------------
-----TESSICDSPSAQ------------------SFTKPHFSLSEKTSSDVND------
----------TDPFFSAKKLEDEQLK----SKTPTHAIT--------------------T
DFEADDFDVDDFDI---DDFNDSDI---------PDYFDEPPTSST--------------
--LSENPNTTCAAVT--KGGPNKPPWDKKP---ATPVSA------------------PKP
QKIYSPEPNF---------------------KNPAH-----DRFRGF-----D-FPH---
------SQEMMKIFHKRFGLHQFRFNQLEAINATLLR--EDAFVL---MPT--GGGKSLC
--------YQLPA---------------------CL----SSGV-TVVVSPLKSLIVDQI
QKLTT------LDI-----------------PATSLSGD---KSDSEA---GRIYMQLSR
KDP--IIKLLYATPE-KVSAS---------------NRLISALQNLYERGLLARFVIDEA
HCV--SQWG------HDFRPDYKKLHELRQKF--PN-IPMMALTATAT----------PR
VQKDILNQLNMS----KPQVFTM--SFNRTNLKYAVL---PK-------------KPKKV
DE--DCISWIKKH-YPR----DSGIVYC-----LSRNDC--------DAL---------A
ESL---KRAGIQALS-YHAGLSDGNR-EYVQSKW----INQDG-CQ----VICATIAFGM
GIDKPDVRYVIHASLPKS-MEGYYQESGRAG-RDGEISHCILF-YSYTDVHRIKRII---
--------SMDQEG--NKHSK--------TTHYNNLHSM--VHFCENVME----------
CRRI---QLLAYFGE--LKFNRNFCKDHPDV--SCDNC--AK----PNEYKLR--KVTDD
VKKIVRFVEENCE-----KVGARFGKTPQQ--NRLTLNML-VD-IFIGAKSAKIQT----
------GMFGMGAAYSRHNADRLFKKLVLDNVLVEDLYITNN-GQAV--SYISAGPKAMN
ILSGH--------------------MEVEFYETESASSIRKQR----------AAVKSIS
K------------------------------------------------------RE--E
MVQNCLKELIDLCKQLGKAFGL-HYYNIFSTATLKKIS--EKLSSDPEALLQIDGVTEDK
LEKYGAEVIKVLQKYSE----------------WQLPVDEQTE------SAGNGWIDTRR
T---------EDDDTE--------SSTYFSNQPAQGQKRK--KAPFFKYSKKMKAYGNQG
YSSKGRG-------------YSYNKSSSGSRGGSQSLGRGSTTSA---------------
----------------------AGKK----------------------------------
------------------------------------------------------------
------------------------------------------------------------
------------------------------------------------------------
------------------------------------------------------------
------------------------------------------------------------
------------------------------------------------------------
```

--IGFLSAP-
--TPQS------------
---------NQRPFLKPTYSHIS----------------
>A0A0S7JFW9

--------MQLSC---

KDP--IIKLLYVTPE-KVSAS---------------HRLISALQNLYERGLLARFVIDEA
HCV--SQWG------HDFRPDYKKLHELRQKF--PN-IPMMALTATAT----------PR
VQKDILNQLNMS----KPQVFTM--SFNRTNLKYAVL---PK-------------KPKKV
DE--DCISWIKKH-YPR----DSGIIYC-----LSRNDC--------DGL---------A
ESL---KRAGIQALS-YHAGLSDGDR-EYVQSKW----INQDG-CQ----VICATIAFGM
GIDKPDVRYVIHASLPKS-MEGYYQESGRAG-RDGEISHCILF-YSYADVHRIKRII---
--------SMDREG--DRHAK--------ATHYSNLHSM--VHFCENVME----------
CRRI---QLLAYFGE--LKFNRNFCKDHPDV--SCDNC--AK----PSEYKLR--KVTDD
VKKIVRFVEEKCE-----KVGARFGKTPQQ--NRLTLNML-VD-IFIGAKSAKIQT----
------GMFGMGAAYSRHNADRLFKKLVLDSVLVEDLYITNN-GQAV--SYISAGPKAMN
ILSGH---
---ME---

---------------------------------VTGPSSSYT------------------

>A0A0S7JFR5

---------------------------------------MMALTATAT----------PR
VQKDILNQLNMS----KPQVFTM--SFNRTNLKYAVL---PK-------------KPKKV
DE--DCISWIKKH-YPR----DSGIIYC-----LSRNDC--------DGL---------A
ESL---KRAGIQALS-YHAGLSDGDR-EYVQSKW----INQDG-CQ----VICATIAFGM
GIDKPDVRYVIHASLPKS-MEGYYQESGRAG-RDGEISHCILF-YSYADVHRIKRII---
--------SMDREG--DRHAK--------ATHYSNLHSM--VHFCENVME----------
CRRI---QLLAYFGE--LKFNRNFCKDHPDV--SCDNC--AK----PSEYKLR--KVTDD
VKKIVRFVEEKCE-----KVGARFGKTPQQ--NRLTLNML-VD-IFIGAKSAKIQT----
------GMFGMGAAYSRHNADRLFKKLVLDSVLVEDLYITNN-GQAV--SYISAGPKAMN
ILSGH---

```
-------------------------------------------------------ME---
------------------------------------------------------------
---------------------------------VTGPSSSYT------------------
------------------------------------------------------------
------------------------------------------------------------
------------------------------------------------------------
------------------------------------------------------------
------------------------------------------------------------
------------------------------------------------------------
------------------------------------------------------------
------------------------------------------------------------
------------------------------------------------------------
------------------------------------------------------------
------------------------------------------------------------
---------------------------------------
>A0A0S7JI41
------------------------------------------------------------
------------------------------------------------------------
------------------------------------------------------------
------------------------------------------------------------
------------------------------------------------------------
------------------------------------------------------------
------------------------------------------------------------
------------------------------------------------------------
------------------------------------------------------------
------------------------------------------------------------
------------------------------------------------------------
------------------------------------------------------------
------------------------------------------------------------
------------------------------------------------------------
------------------------------------------------------------
------------------------------------------------------------
------------------------------------------------------------
------------------------------------------------------------
------------------------------------------------------------
------------------------------------------------------------
------------------------------------------------------------
------------------------------------------------------------
------------------------------------------------------------
------------------------------------------------------------
------------------------------------------------------------
------------------------------------------------------------
------------------------------------------------------------
---------------------------------------MMALTATAT----------PR
VQKDILNQLNMS----KPQVFTM--SFNRTNLKYAVL---PK-------------KPKKV
DE--DCISWIKKH-YPR----DSGIIYC-----LSRNDC--------DGL---------A
ESL---KRAGIQALS-YHAGLSDGDR-EYVQSKW----INQDG-CQ----VICATIAFGM
```

GIDKPDVRYVIHASLPKS-MEGYYQESGRAG-RDGEISHCILF-YSYADVHRIKRII---
--------SMDREG--DRHAK--------ATHYSNLHSM--VHFCENVME----------
CRRI---QLLAYFGE--LKFNRNFCKDHPDV--SCDNC--AK----PSEYKLR--KVTDD
VKKIVRFVEEKCE-----KVGARFGKTPQQ--NRLTLNML-VD-IFIGAKSAKIQT----
------GMFGMGAAYSRHNADRLFKKLVLDSVLVEDLYITNN-GQAV--SYISAGPKAMN
ILSGH----M-------------E---VEFYETESTSSIRKQR----------AAVKSIS
K--RE--E
MVQNCLKELIDLCKQLGKAFGL-HYYNIFSTATLKKIS--EKLSSDPDALSCRS------
---------------------------------WR-------------------------

>A0A0S7JFT6

--------MQLSC---

KDP--IIKLLYVTPE-KVSAS---------------HRLISALQNLYERGLLARFVIDEA
HCV--SQWG------HDFRPDYKKLHELRQKF--PN-IPMMALTATAT----------PR
VQKDILNQLNMS----KPQVFTM--SFNRTNLKYAVL---PK-------------KPKKV
DE--DCISWIKKH-YPR----DSGIIYC-----LSRNDC--------DGL---------A
ESL---KRAGIQALS-YHAGLSDGDR-EYVQSKW----INQDG-CQ----VICATIAFGM
GIDKPDVRYVIHASLPKS-MEGYYQESGRAG-RDGEISHCILF-YSYADVHRIKRII---
--------SMDREG--DRHAK--------ATHYSNLHSM--VHFCENVME----------
CRRI---QLLAYFGE--LKFNRNFCKDHPDV--SCDNC--AK----PSEYKLR--KVTDD
VKKIVRFVEEKCE-----KVGARFGKTPQQ--NRLTLNML-VD-IFIGAKSAKIQT----
------GMFGMGAAYSRHNADRLFKKLVLDSVLVEDLYITNN-GQAV--SYISAGPKAMN
ILSGH----M-------------E---VEFYETESTSSIRKQR----------AAVKSIS
K--RE--E
MVQNCLKELIDLCKQLGKAFGL-HYYNIFSTATLKKIS--EKLSSDPDALLQIDGVTEDK
LEKYGAEVMKVLQKYSE----------------WQLSVDEQTD------SAGNGWIDTRR
----------TEDDTE--------SSTYFSNRSAPGQKRK--KGPFFKYSKKMKAHGNQG
YSSKGRG-------------YSYNKSSSGSRGGSQSSGRGSTSSA---------------
----------------------AGKT----------------------------------

--LGFLSAP-
--TPQS------------
---------NQRPFLKGTYSHIS----------------
>A0A0S7JHH7

---------------------------------------MMALTATAT----------PR
VQKDILNQLNMS----KPQVFTM--SFNRTNLKYAVL---PK-------------KPKKV
DE--DCISWIKKH-YPR----DSGIIYC-----LSRNDC--------DGL---------A
ESL---KRAGIQALS-YHAGLSDGDR-EYVQSKW----INQDG-CQ----VICATIAFGM
GIDKPDVRYVIHASLPKS-MEGYYQESGRAG-RDGEISHCILF-YSYADVHRIKRII---
--------SMDREG--DRHAK--------ATHYSNLHSM--VHFCENVME----------
CRRI---QLLAYFGE--LKFNRNFCKDHPDV--SCDNC--AK----PSEYKLR--KVTDD
VKKIVRFVEEKCE-----KVGARFGKTPQQ--NRLTLNML-VD-IFIGAKSAKIQT----
------GMFGMGAAYSRHNADRLFKKLVLDSVLVEDLYITNN-GQAV--SYISAGPKAMN
ILSGH----M-------------E---VEFYETESTSSIRKQR----------AAVKSIS
K--RE--E
MVQNCLKELIDLCKQLGKAFGL-HYYNIFSTATLKKIS--EKLSSDPDALLQIDGVTEDK
LEKYGAEVMKVLQKYSE----------------WQLSVDEQTD------SAGNGWIDTRR
TEDDTE------------------SSTYFSNRSAPGQKRK--KGPFFKYSKKMKAHGNQG
YSSKGRG-------------YSYNKSSSGSRGGSQSSGRGSTSSA---------------
----------------------AGKT----------------------------------

--LGFLSAP-
--TPQS------------
---------NQRPFLKGTYSHIS----------------
>H0ZI69

---LEEDDLDIIP---PSPE
EELP-----------------------------AFSPSVQSASVFRDSPTGGRCTAGSSE
ARQEKVSTVHPGG--DPSAGDAGEAVPVAPGLYR--------------------------
----------------------VMEAICE-------------------------------

```
------------------------------------------LVDAIPLQELQALR----
---------------------CARALLQHRELR----------------------RKLLA
NSCHLNQNGTNTTFPRS----------WKSPVEQSPRVCPGAGPGSVP------------
---------------SRSFGSDRNSPKSTN-----------LPSVLSVISSSFSA-----
------------------------------------------------------STNRTL
GSSGASKQSAEEMPCPGAAELPCPTGGGRQGASL-RLSPRSCNSSWDPSPGARNGI----
-----------------QPLCSTALRAPSTAATASPAGS-------------------SL
AADHPDLELDAFDI---DDL--------------DEDWESLLPAAAA-------------
---EAPATPLCPPVP--RGPPAQSLLAKIMSRATGPAGG---------------------
SSPAAPKPGLPMATKS--HPDPVV-------HNPAL-----ERFRGM-----K-FPH---
------SAEMMNIFHRKFGLHCFRTNQLEAINAALLG--EDCFIL---MPT--GGGKSLC
--------YQLPA---------------------CV----SAGV-TIVISPLRSLIIDQV
QKLKT------LDI-----------------AATYLTGD---RTDADA---SKIYMQLSK
KDP--VIKLLYVTPE-KVCAS---------------NRLMSTLENLYDRKLLARFVIDEA
HCV--SQWG------HDFRQDYKRLNMLRRKF--GS-VPMMALTATAN----------PR
VQKDILNQLEML----KPQVFTM--SFNRHNLKYDVL---PK-------------KPKKV
AL--DCLEWIKKY-HPH----DSGIIYC-----LSRHEC--------DTT---------A
AIL---QKEGLAALA-YHAGLTDSNR-DLVQQKW----VNQEG-CQ----VICATIAFGM
GIDKPDVRYVIHASLPKS-IEGYYQESGRAG-RDGEMSHCLLF-YSYSDVTRLRRLI---
--------LMEKDG--NSHTR--------QTHFNNLYSM--VHYCENVVE----------
CRRV---QLLAYFGE--TNFNPTFCKDHPEV--ICDNCSRKK----VNTQWCQTEFVFSC
IKKWSKHLGLCKL-----SSSSRREGSCGGQQGRVLISFVPVD-IVSQFLFTQQSC----
-----------TSLYTDNPREIIYSQLDWFCVITSRIPVTVS-LKVLTPTKMAKKRKKME
LALVK----------------------VEFHETESASAIRRQR----------ASMAKMS
Q-----------------------------------------------------RE--E
MVKQCLSELTDTCKTLGKVFDV-HYFNIFSTSTLKKIA--ETLSSDVEVLLQIDGVTEDK
LEKYGAEIIKVMDKYSE----------------CSIPEDAACP------GGDTATGSTGS
LGSDG-----EAEDAGT-------TSSYFGNDTNQRRKRK--RPPTSRDSKRKKTGG-GS
QQFHPRG------------RYRRTKKLQSSKAPASSGASSGAAGK---------------
------------------------------------------------------------
------------------------------------------------------------
------------------------------------------------------------
------------------------------------------------------------
------------------------------------------------------------
------------------------------------------------------------
------------------------------------------------------------
----------------------------------------------------LAMMALP-
--------------------------------------------KP--------------
---------KSRSFLQPSYSIL-----------------
>U3K8X3
------------------------------------------------------------
------------------------------------------------------------
------------------------------------------------------------
------------------------------------------------------------
------------------------------------------------------------
-------------------------------------------GPLIAVGQEWDDI-DDF
D--LSGIEKKFSRPPVVSPRGLRAPRTEPPRPSPRPAPALPP------------------
```

------------------LPGPVSVPRPASQGSVICLGDSSPRP----------------
-------------------------------ALDSAGNGCAGYSAHTWAAGAEGGQEPQG
AGDSPPDLAG------------------------DTWPLAGAELEEDDLDIIP---PSPE
EELP------------------------------PFSPSVQSASSVFRDTPTGGRCSAGS
SEARQEKDPTTHPAADPGAEDSGEAVPVAQQLLR--------------------------
----------------------VTEAICE-------------------------------
--LVDAIPLQELQALG----
---------------------CARALLQHRDRR----------------------RKLLA
NYNSVSVNQNGTTPPFPRS--------WKPPVEQSPPVCPGAGPGSGP------------
-------------SWSFSSDRNSPKPTNLPSVL--------SLHSSGSSAKTNQT-----
---L
AAPCASRQGAEELPCARGGGREGAAP---------RLSLQSCSSSWEQAAGAPRAQ----
----------STAASASPAGSSLGTR----------------------------------
---HPDLDLHLFDI---DDL--------------DEDWESPGAAAAT-------------
-----------------EGPPAKSLCSKLLSRAKG----------------------SVG
SGPAAPKPGLLMATKSPPDPVV---------HNPAL-----ERFRGM-----K-FPH---
------SAEMMNIFHKKFGLHCFRTNQLEAINAALLG--EDCFVL---MPT--GGGKSLC
--------YQLPA---------------------CV----SAGV-SVVISPLRSLIIDQV
QKLKT------LDI-----------------AATYLTGD---RTDADA---SKIYMQLSK
KDP--VIKLLYVTPE-KVCAS---------------SRLMSALENLYDRKLLARFVIDEA
HCV--SQWG------HDFRQDYKRLNVLRRRF--HR-VPMMALTATAT----------PR
VQKDILNQLEML----RPQVFTM--SFNRHNLKYDVL---PK-------------KPKKV
AM--DCLEWIKKY-HPH----DSGIIYC-----LSRHEC--------DTT---------A
AIL---QKEGLAALA-YHAGLTDSSR-DLVQQKW----VNQEG-CQ----VICATIAFGM
GIDKPDVRYVIHASLPKS-IEGYYQESGRAG-RDGEMSHCLLF-YSYSDVTRLRRLI---
--------LMEKDG--NSHTR--------QTHFNNLYSM--VHYCENVVE----------
CRRV---QLLAYFGE--TNFNPTFCKDHPEV--ICDNC--SR----KKDYRCR--DVTED
VRSIVRFVRQHCG--------QAGHGRGSPASGRYTLNMM-VD-IFLGSTSAKIQS----
------GIFGKGAAYSRHNAERLFRKLVLDKILDEDLYITAN-DQAV--AYVILGERAQA
VLDGS--------------------LQVEFHETESASAIRRQR----------ASMAKMS
Q--RE--E
VVKQCLSELTDTCKTLGKVFDV-HYFNIFSTSTLKKIA--ESLSSDMEVLLQIDGVTEDK
LEKYGAEIIKVMDKYSE----------------CSFPEDAACSGVDTATGSTGSLGS---
----------DAEDAGT-------TSSYFGNNTNQRRKRK--RPPNSRDCKRKKTSGGGS
QQFQPRG---------GYSRYRRTKKLPSSKAAASSSASSVSYSA---------------
-----------------GGTQGAAGK----------------------------------

--LAMMALP-
--KP--------------
---------KSRSFLQPSFSVF-----------------
>A0A218UFA3

```
------------------------------------------------------------
------------------------------------------------------------
------------------------------------------------------------
------------------------------------------------------------
------------------------------------------------------------
------------------------------------------------------------
------------------------------------------------------------
------------------------------------------------------------
------------------------------------------------------------
------------------------------------------------------------
------------------------------------------------------------
-----------------------MEAICE-------------------------------
------------------------------------------LVDAIPLQELQALR----
---------------------CARALLQHRELR----------------------RKLLA
--NSVSLNQNGMNTTFP----RS----WKSPVEQSPRVCPYAGPGSGP------------
-------------SWSFGSDRN--SPKSTN-----------LPSVLS--VHSSSF-----
----------------SAKTNRTLG-----------------------------------
-SSCASERSAEEMPCPGAAERPCPKGGGRAGASL-GLSPQSCDSSWDRSPAA-RNG----
----------I------QPLCSTALR----APSTTAAASP-----------AGSSLAA--
--DHPDLELDAFDI---DDL--------------DEEWENLL-PAAA-------------
--TEAPATPLCPPVP--RGPPAQSLLSKIM---SRATGS------------------AVG
SSPAAPKPGLPMATKS--HPDPVV-------HNPAL-----ERFRGM-----K-FPH---
------SAEMMNIFHRKFGLHCFRTNQLEAINAALLG--EDCFIL---MPT--GGGKSLC
--------YQLPA---------------------CV----SAGV-TIVISPLRSLIIDQV
QKLKT------LDI-----------------AATYLTGD---RTDADA---SKIYMQLSK
KDP--VIKLLYVTPE-KVCAS---------------NRLMSALENLYNRKLLARFVIDEA
HCV--SQWG------HDFRQDYKRLNMLRRKF--GS-VPMMALTATAN----------PR
VQKDILNQLEML----KPQVFTM--SFNRHNLKYDVL---PK-------------KPKKV
AL--DCLEWIKKY-HPH----DSGIIYC-----LSRHEC--------DTT---------A
AIL---QKEGLAALA-YHAGLTDSNR-DLVQQKW----VNQEG-CQ----VICATIAFGM
GIDKPDVRYVIHASLPKS-IEGYYQESGRAG-RDGEMSHCLLF-YSYSDVTRLRRLI---
--------LMEKDG--NSHTR--------QTHFNNLYSM--VHYCENVVE----------
CRRV---QLLAYFGE--TNFNPTFCKDHPEV--ICDNC--SR----KKDYKSR--DVTED
VKGIVRFVREHCG-----RAGRMDGRRNPG-SGRYTLNMM-VD-IFLGSTSAKIQS----
------GIFGKGAAYSRHNAERLFRKLVLDKILDEDLYITAN-DQAV--AYVILGERAQA
VLDGS----L-------------Q---VEFHETESASAIRRQR----------ASMAKMS
Q------------------------------------------------------RE--E
MVKQCLSELTDTCKTLGKVFDV-HYFNIFSTSTLKKIA--ETLSSDVEVLLQIDGVTEDK
LEKYGAEIIKVMDKYSE----------------CAISEDAACP--GGDTATGST----GS
LGSDG-----EAEDAGT-------TSSYFGNDTNQRRKRK--RLPTSMDSKRKKT-GGGS
QQFHPRG---------GYSRYRRTKKRPSSKAPASSGASSVSYSV-----------GGTQ
---------------------GAAWK----------------------------------
------------------------------------------------------------
------------------------------------------------------------
------------------------------------------------------------
------------------------------------------------------------
------------------------------------------------------------
```

--LAMMALP-
--KP-------------
---------KSRSFLQPSYSVL-----------------
>A0A1V4JDR7
--------------------------------MAAVPQNNLREQL---------------

--RLHS-ARGGLGKAA--PPRPRP--------TGFT------------------------
---------FKKLSPVGRGPAAGALRDK-------------------------DVNAALG
PGPPAAAARRTQIQHFF----PAAGGQRGLGPGCGLRDPPGG-----PGGSEGLRGAP--
--ARGAGPG-P--A---AAPAAPA-------------------------GDQWDDI-DDF
D--LSG-TQRFGRVPALSATGQPPRGANPRPDQPPGASPGTG-------------PGPRS
PPEH-------------GEASPEREPR--PLSQQSLICLDDLAPCSGDPAAGGGDW---E
NSPAGLV-LDNDGEELHPGA----------EDQSGQRQQP-------------------S
GGGSSSGGSS--------RPPGRDDD------SVDLEL----DEDDE-LDVVP---PSPE
KP-P-------SCSPSVK---------------SVSNIFKEFPP-----GGRS-TPSSTE
SQPGKVVTMQPVADSDPGVEDAGKGLR------S--------------------------
----------------------VMEEMCR-------------------------------
--LLDTVPLRELQALS----
---------------------CARDLLRLRDLR----------------------RKLLA
--GSVTRSVSGTSDTFP----MS----RRACAEQEPLTQPGAAPCAGT------------
-------------SSSFVSNRN--SPRPTH-----------LPPVRPGTVHPSNF-----
----------------STKTNQTLD-----------------------------------
-TSCASKPSVEELGCLSTTALPIPIPKANGTDG----------SGWCEEPRV-GTA----
----------G--------------------PASRAAAATP-----------TAGISAA--
--NDTDFDLDYFDI---DDF--------------DEDWENSV-NVSA-------------
--PQTPSAPSYQPIG--EGPPTKSLSSKIL---SRVKGA------------------AAV
SSAAAPKSSFVMATKN--SSDQPV-------NNPAL-----ERFRGT-----K-FPH---
------SEEMMNTFHKKFGLHCFRTNQLEAINAALLG--EDCFIL---MPT--GGGKSLC
--------YQLPA---------------------CV----SAGV-TIVISPLRSLIIDQV
QKLKT------LDI------------------AATYLTGD---RTDADA---SKIYMQLSK
KDP--VIKLLYVTPE-KVCAS---------------SRLMSALENLYDRRLLARFVIDEA
HCV--SQWG------HDFRQDYKRLNMLRKKF--RS-VPMMALTATAN----------PR
VQKDIQNQLEML----KPQVFTM--SFNRQNLKYDVL---PK-------------KPKKV
AT--DCLEWIKKY-HPH----DSGIIYC-----LSRHEC--------DTT---------A
AIL---QKEGLAALA-YHAGLPDSNR-DLVQKKW----VNQEG-CQ----VICATIAFGM
GIDKPDVRYVIHASLPKS-LEGYYQESGRAG-RDGEMSHCLLF-YSYSDVTRLRRLI---
--------LMEKDG--NSHTR--------QTHFNNLYSM--VHYCENVVD----------
CRRI---QLLAYFGE--TNFNPNFCKDHPEV--ICDNC--SR----KKDYKSR--NVTDE
VKSIIRFVQEHCG-----QMGRINAKRNPG-SGRYTLNMM-VD-IFLGTKSAKIQS----
------GIFGKGAAYSRHNVERLFRKLVLDKILDEDLYITAN-DQAV--AYVVLGEKAPA
VLNGS----L-------------Q---VEFHETESASAIRKQR----------ASVAKMS
Q---RE--E
MVKKCLGELTDTCKMLGKVFDV-HYFNIFSTSTLKKIA--ETLSSDAEVLLQIDGVTEDK
LEKYGAEIIKVMEKYAE----------------WTLPEDAACQ--SADTATGST----GT
PESDE-----DTEDVGT-------TSSYFCNNANQRRKRK--RPPNFRESKRKKTGHGGS
QQFHYKG---------GYSKYRRTRRPLGSKAPAGSGSGSAPHSV-----------PELR

VLLTPLRAGRRLPQKRLLSGGGTGGKRLRLERPPERPTGTGPGSPADSGSDGAGDGDGDS
DGSDAGDAARDGCSQLSAYERKRLKNITENAKFFAALKLHESAARLYQIASRKQPHVTKR
AKPKKAEDEPVRRRSMRLQRVEPSGIPVPEPEAEELPRVPAGPVPMVPEDQESSKLTQEL
LATWMRISEMKADGTEKLTYDMKRYQDSLSSMVLSEENIRKVVKYRVCSMAIHPSQSVVL
VAAGDKSGQVGLWNVDGESAGTCVFVPHSSMVSCMHFSPWHPAHLLSLSYDTLRCGDVTR
AIFDEICRSEDDNFSSFDFLDDNGSTVVMGHWPGDVAVVDIRTPGTSPELSADIGFKRTR
TVHVHPVNKQYFMAAGSVDVCIYDVRYLKSKGNKPVSSLKGHTKSVASAYFSPVTGNRVV
TVCADDKLRVYDTSSLSSTLAALSTISHNCNTGRWLTRFRAVWDPKQERCFAVGSMARP-
--RQVQLFRDTGALLHTF
CNPDCLGSVCSINVLHPTRNILVGGNSSGRLHVFKE---
>U3IGZ2

-----------------------QYASLSATNDKSGGSQKLS------------------
------------------------NSEKNSQPEVDEISNELLAN----------------

--IELEEDDYLDVVP---PSPE
EELP-------SFSPSVK---------------SISNIFKESPTGGRS------TANGIE
SKPEKITAKQPVAESDPRAEDADEGPHLEQELYS--------------------------
----------------------VMDDICK-------------------------------
--LVDAIPIHELKSLS----
---------------------CAKELLQQRDLR----------------------RKLLA
SSVTLNTSSTNNTFPRN----------WKACVQQDSSAHSGTALCSGP------------
---------------NRSFVRN--SPKSTN-----------LSSMLSGTVNSSNF-----
----------------STENNQTLD---------------------------------DL
ATSYVSKQTAQEIVCLETAELSSSKLNSKDRTSLSHPSEMSFNNSWCEKTVVRNG-----
----------EHWHLPERPITSTALK----GPSKAPAGT---------------PASECL
DVNDPDFDLDNFDI---DDF--------------DEGWEENPVTVSA-------------
--PETPSTPLYQPLR--EGPPAKSLLSKII---STAKGS------------------PLV
SNPVAPKSSFLTATKNQTEPLV---------NNPAL-----ERFRGM-----K-FSH---
------SEEMMTIFHRKFGLHTFRTNQLEAINAALLG--EDCFIL---MPT--GGGKSLC
--------YQLPA---------------------CV----SAGV-TIVISPLRSLIIDQV
QKLNT------LDI-----------------AATYLTGD---RTDTDA---SNIYMQLSK
KDP--IIKLLYVTPE-KVCAS---------------NRLMAALENLYNRKLLARFVIDEA
HCV-SQKWG------HDFRKDYKRLNMLRKKF--RS-VPMMALTATAN----------PR
VQKDIQNQLEML----RPQVFTM--SFNRHNLKYDVL---PK-------------KPKRV
AM--DCLEWIKKY-HPH----DSGIIYC-----LSRHEC--------DTT---------A
AIL---QKEGLSALA-YHAGLTDSNR-DLVQKKW----INQEG-CQ----VICATIAFGM
GIDKPDVRYVIHASLPKS-VEGYYQESGRAG-RDGEMSHCLLF-YSYSDVTRLRRLI---
--------LMEKDG--NSHTR--------ETHFNNLYSM--VHYCENVVD----------
CRRI---QLLAYFGE--TNFNPNFCKDHPEV--ICDNC--SR----KKDYKSR--NVTEE
VKSIIRFVQQHCG-----QMRGMNAKRNAG-SGRYTLNMM-VD-IFLGTKSAKIQS----
------GIFGNGAAYSRHNVERLFRKLVLDKILDEDLYITAN-DQAV--AYVILGERAQA

VLNGS----L-------------Q---VEFHETESASAIRKQR----------ASVTKMS
Q--RE--E
MVKKCLSELTDTCKTLGKVFDV-HYFNIFSTSTLKKIA--ETLSSDVEVLLQIDGVTEDK
LEKYGAEIIKVMDKYSE----------------WTVPEDAACQ------SVDTAGGSTGT
PESEE-----EAGDVVT-------TSSYFCNNANQRRKRK--RPPNFRESKKKKTSSAGS
QQFRPKG---------GYSKYRRTKKPSNSKTPPSSGYSSASYSV-----------SATQ
---------------------GTVGK----------------------------------

--LGIMAPP-
--KP--------------
---------KNRQFLQPSYSLL-----------------
>R0KFM7

-----------------SAT----------NDKSGGSQKL-------------------S
NSEKNSQPEV--------DEIS-NEL------LANIEL----EEDDY-LDVVP---PSPE
EELP-------SFSPSVK---------------SISNIFKESPT-----GGRS-TANGIE
SKPEKITAKQPVAESDPRAEDADEGPHLEQELYS--------------------------
----------------------VMDDICK-------------------------------
--LVDAIPIHELKSLS----
---------------------CAKELLQQRDLR----------------------RKLLA
--SSVTLNTSSTNNTFP----RN----WKACVQQDSSAHSGTALCSGP------------
-------------NR--SFVRN--SPKSTN-----------LSSMLSGTVNSSNF-----
----------------STENNQTLD---------------------------------DL
ATSYVSKQTAQEIVCLETAELSSSKLNSKDRTSLSHPSEMSFNNSWCEKTVV-RNG----
----------EHWHLPERPITSTALK----GPSKAPAGTP-----------ASECLDV--
--NDPDFDLDNFDI---DDF--------------DEGWEENPVTVSA-------------
--PETPSTPLYQPLR--EGPPAKSLLSKII---STAKGS------------------PLV
SNPVAPKSSFLTATKN--QTEPLV-------NNPAL-----ERFRGM-----K-FSH---
------SEEMMTIFHRKFGLHTFRTNQLEAINAALLG--EDCFIL---MPT--GGGKSLC
--------YQLPA---------------------CV----SAGV-TIVISPLRSLIIDQV
QKLNT------LDI-----------------AATYLTGD---RTDTDA---SNIYMQLSK
KDP--IIKLLYVTPE-KVCAS---------------NRLMAALENLYNRKLLARFVIDEA
HCV--SQWG------HDFRKDYKRLNMLRKKF--RS-VPMMALTATAN----------PR
VQKDIQNQLEML----RPQVFTM--SFNRHNLKYDVL---PK-------------KPKRV
AM--DCLEWIKKY-HPH----DSGIIYC-----LSRHEC--------DTT---------A

```
AIL---QKEGLSALA-YHAGLTDSNR-DLVQKKW----INQEG-CQ----VICATIAFGM
GIDKPDVRYVIHASLPKS-VEGYYQESGRAG-RDGEMSHCLLF-YSYSDVTRLRRLI---
--------LMEKDG--NSHTR--------ETHFNNLYSM--VHYCENVVD----------
CRRI---QLLAYFGE--TNFNPNFCKDHPEV--ICDNC--SR----KKDYKSR--NVTEE
VKSIIRFVQQHCG-----QMRGMNAKRNAG-SGRYTLNMM-VD-IFLGTKSAKIQS----
------GIFGNGAAYSRHNVERLFRKLVLDKILDEDLYITAN-DQAV--AYVILGERAQA
VLNGS----L-------------Q---VEFHETESASAIRKQR----------ASVTKMS
Q------------------------------------------------------RE--E
MVKKCLSELTDTCKTLGKVFDV-HYFNIFSTSTLKKIA--ETLSSDVEVLLQIDGVTEDK
LEKYGAEIIKVMDKYSE----------------WTVPEDAACQ--SVDTAGGST----GT
PESEE-----EAGDVVT-------TSSYFCNNANQRRKRK--RPPNFRESKKKKTSSAGS
QQFRPKG-----------------------------------------------------
------------------------------------------------------------
------------------------------------------------------------
------------------------------------------------------------
------------------------------------------------------------
------------------------------------------------------------
------------------------------------------------------------
------------------------------------------------------------
------------------------------------------------------------
------------------------------------------------------------
---------------------------------------
>A0A1L1RX68
------------------------------------------------------------
------------------------------------------------------------
------------------------------------------------------------
------------------------------------------------------------
------------------------------------------------------------
-------------------------------------------------------M-DDF
D--LSGFEKKFSRPAVLSPKG-------PRTPQGRGLRVSKA-------------RADVG
PQEH-------------GAEVSCSQGS----SQGSLICL--------------GDA---G
APAAAVL-SDNGMEEARAAT----------DGGSGESQKL-------------------S
NGEKSSQLEP--------GDVG-NEL------LADIEL----EEDDY-LDVVP---PSPE
EELP-------SFSPSVR---------------NVSNIFKESPT-----DGRS-AVHGTE
SEPELMAPKQPAAEQDSSAEHADKGLHLEQQLYS--------------------------
----------------------VMEDICK-------------------------------
------------------------------------------LVDAIPLHELTSIS----
---------------------CAKELLQQRELR----------------------RKLLA
--DSGALNTNSVNG--P----RN----WKACVQQDPSSRPGTPLCSGP------------
-------------GRGVSSVGS--TPKSTN-----------LPPVLSRTVNSSSF-----
----------------STIRNQTLD---------------------------------KL
DTSYSSKETDQEVICLEPAALPSPKVNGKGSTSLSRPSEASFNGSWCEKPTG-RDS----
----------GNWRVPERPTASTALK----AQHTAPAGAP-----------ASGCWDV--
--NDTDFDLDHFDI---DDF--------------DEGWEDAVAPEAA-------------
--PEAPPAPQWQPLR--EG--SASLRCRLL---AAAAGS------------------APG
PHPTAPKSGCGISAKS--SSEPLV-------HNPAH-----ERFRGM-----K-FSH---
------SEEMLKIFHRKFGLHSFRTNQLEAINAALLG--EDCFIL---MPT--GGGKSLC
```

--------YQLPA---------------------CV----SAGV-TVVISPLRSLIIDQV
QKLKT------LDI-----------------AATYLTGD---ITDADA---SKTYMQLSK
KDP--IIKLLYVTPE-KVCAS---------------NRLLSALENLYDRKLLARFVIDEA
HCV--SQWG------HDFRKDYKRLNMLRKKF--HS-VPMMALTATAN----------PR
VQKDIQNQLEML----KPQVFTM--SFNRHNLKYDVL---PK-------------KPKKV
AM--DCLEWIKKY-HPH----DSGIIYC-----LSRHEC--------DTT---------A
AIL---QKEGLAALA-YHAGLTDSNR-DLVQKKW----VNQEG-CQ----VICATIAFGM
GIDKPDVRYVIHASLPKS-IEGYYQESGRAG-RDGEMSHCLLF-YSYSDVTRLRRLI---
--------LMEKDG--NSHTR--------QTHFNNLYSM--VHYCENVVD----------
CRRI---QLLAYFGE--TDFNPNFCKDHPEV--ICDNC--SR----KKDYKSR--NVTDE
VKSIIRFVQQHCG-----QMGGINGNRNTG-SGRYTLNMM-VD-IFLGAKSAKIQS----
------GIFGKGAAYSRHNVERLFRKLVLDKILDEDLYITAN-DQAV--AYVVLGEKAQA
VLNGL----L-------------Q---VEFHETENASAIRKQR----------ASVTKMS
Q--RE--E
MVKKCLGELTDTCKTLGKIFDV-HYFNIFSTSTLKKIA--ETLSSDAEVLLQIDGVTEDK
LEKYGAEIIKVMDKYSE----------------WTTPEDAACQ--SVDTAPGSA----GT
PGSEE-----EAEDDVV-------TSSYFGGNANQRRKRK--RLPNSRESKRKKTSSGGS
QQFYSKG---YV
---------------------GCG-Q----------------------------------

--LELFV---

---------QGNMVMIPELQC------------------
>A0A1D5PWU9
--------------------------------MAAVPQNNLREQL---------------

--RLHS-ARGALSKVL--PARSRPLTPGCVSCRAFT------------------------
---------FRRTHSPAAGPA--ALRDK-------------------------DVNTSLG
SGGVARPK------------------DLRVGPPGARPALPGP-----AGSSGPTAGAP--
--PGSVSVSLP--S---SGPAAYT-------------------------GDEWDDM-DDF
D--LSGFEKKFSRPAVLSPKG-------PRTPQGRGLRVSKA-------------RADVG
PQEH-------------GAEVSCSQGS----SQGSLICL--------------GDA---G
APAAAVL-SDNGMEEARAAT----------DGGSGESQKL-------------------S
NGEKSSQLEP--------GDVG-NEL------LADIEL----EEDDY-LDVVP---PSPE
EELP-------SFSPSVR---------------NVSNIFKESPT-----DGRS-AVHGTE
SEPELMAPKQPAAEQDSSAEHADKGLHLEQQLYS--------------------------
----------------------VMEDICK-------------------------------
--LVDAIPLHELTSIS----
---------------------CAKELLQQRELR----------------------RKLLA
--DSGALNTNSVNG--P----RN----WKACVQQDPSSRPGTPLCSGP------------
-------------GRGVSSVGS--TPKSTN-----------LPPVLSRTVNSSSF-----
----------------STIRNQTLD---------------------------------KL

DTSYSSKETDQEVICLEPAALPSPKVNGKGSTSLSRPSEASFNGSWCEKPTG-RDS----
----------GNWRVPERPTASTALK----AQHTAPAGAP-----------ASGCWDV--
--NDTDFDLDHFDI---DDF--------------DEGWEDAVAPEAA-------------
--PEAPPAPQWQPLR--EG--SASLRCRLL---AAAAGS------------------APG
PHPTAPKSGCGISAKS--SSEPLV-------HNPAH-----ERFRGM-----K-FSH---
------SEEMLKIFHRKFGLHSFRTNQLEAINAALLG--EDCFIL---MPT--GGGKSLC
--------YQLPA---------------------CV----SAGV-TVVISPLRSLIIDQV
QKLKT------LDV---------------------------------S---SDGFISFSR
KTT--RIRWL----A-AVCAS---------------NRLLSALENLYDRKLLARFVIDEA
HCV--SQWG------HDFRKDYKRLNMLRKKF--HS-VPMMALTATAN----------PR
VQKDIQNQLEML----KPQVFTM--SFNRHNLKYDVL---PK-------------KPKKV
AM--DCLEWIKKY-HPH----DSGIIYC-----LSRHEC--------DTT---------A
AIL---QKEGLAALA-YHAGLTDSNR-DLVQKKW----VNQEG-CQ----VICATIAFGM
GIDKPDVRYVIHASLPKS-IEGYYQESGRAG-RDGEMSHCLLF-YSYSDVTRLRRLI---
--------LMEKDG--NSHTR--------QTHFNNLYSM--VHYCENVVD----------
CRRI---QLLAYFGE--TDFNPNFCKDHPEV--ICDNC--SR----KKDYKSR--NVTDE
VKSIIRFVQQHCG-----QMGGINGNRNTG-SGRYTLNMM-VD-IFLGAKSAKIQS----
------GIFGKGAAYSRHNVERLFRKLVLDKILDEDLYITAN-DQAV--AYVVLGEKAQA
VLNGL----L-------------Q---VEFHETENASAIRKQR----------ASVTKMS
Q---RE--E
MVKKCLGELTDTCKTLGKIFDV-HYFNIFSTSTLKKIA--ETLSSDAEVLLQIDGVTEDK
LEKYGAEIIKVMDKYSE----------------WTTPEDAACQ--SVDTAPGSA----GT
PGSEE-----EAEDDVV-------TSSYFGGNANQRRKRK--RLPNSRESKRKKTSSGGS
QQFYSKG-----------ARYRRARRAPGSR-------AAAPAQS-----------SALR
---------------------GAGAR----------------------------------

--LGIMAPP-
---KP-------------
---------SSRHFLQPSYAVL-----------------
>F1P3V1
--------------------------------MAAVPQNNLREQL---------------

--RLHS-ARGALSKVL--PARSRP--------AAFT------------------------
---------FRRTHSPAAGPA--ALRDK-------------------------DVNTSLG
SGGVARPK------------------DLRVGPPGARPALPGP-----AGSSGPTAGAP--
--PGSVSVSLP--S---SGPAAYT-------------------------GDEWDDM-DDF
D--LSGFEKKFSRPAVLSPKG-------PRTPQGRGLRVSKA-------------RADVG
PQEH-------------GAEVSCSQGS----SQGSLICL--------------GDA---G
APAAAVL-SDNGMEEARAAT----------DGGSGESQKL-------------------S
NGEKSSQLEP--------GDVG-NEL------LADIEL----EEDDY-LDVVP---PSPE
EELP-------SFSPSVR---------------NVSNIFKESPT-----DGRS-AVHGTE
SEPELMAPKQPAAEQDSSAEHADKGLHLEQQLYS-------------------------

----------------------VMEDICK-------------------------------
--LVDAIPLHELTSIS----
---------------------CAKELLQQRELR----------------------RKLLA
--DSGALNTNSVNG--P----RN----WKACVQQDPSSRPGTPLCSGP------------
-------------GRGVSSVGS--TPKSTN-----------LPPVLSRTVNSSSF-----
----------------STIRNQTLD---------------------------------KL
DTSYSSKETDQEVICLEPAALPSPKVNGKGSTSLSRPSEASFNGSWCEKPTG-RDS----
----------GNWRVPERPTASTALK----AQHTAPAGAP-----------ASGCWDV--
--NDTDFDLDHFDI---DDF--------------DEGWEDAVAPEAA-------------
--PEAPPAPQWQPLR--EG--SASLRCRLL---AAAAGS------------------APG
PHPTAPKSGCGISAKS--SSEPLV-------HNPAH-----ERFRGM-----K-FSH---
------SEEMLKIFHRKFGLHSFRTNQLEAINAALLG--EDCFIL---MPT--GGGKSLC
--------YQLPA---------------------CV----SAGV-TVVISPLRSLIIDQV
QKLKT------LDI-----------------AATYLTGD---ITDADA---SKTYMQLSK
KDP--IIKLLYVTPE-KVCAS---------------NRLLSALENLYDRKLLARFVIDEA
HCV--SQWG------HDFRKDYKRLNMLRKKF--HS-VPMMALTATAN----------PR
VQKDIQNQLEML----KPQVFTM--SFNRHNLKYDVL---PK-------------KPKKV
AM--DCLEWIKKY-HPH----DSGIIYC-----LSRHEC--------DTT---------A
AIL---QKEGLAALA-YHAGLTDSNR-DLVQKKW----VNQEG-CQ----VICATIAFGM
GIDKPDVRYVIHASLPKS-IEGYYQESGRAG-RDGEMSHCLLF-YSYSDVTRLRRLI---
--------LMEKDG--NSHTR--------QTHFNNLYSM--VHYCENVVD----------
CRRI---QLLAYFGE--TDFNPNFCKDHPEV--ICDNC--SR----KKDYKSR--NVTDE
VKSIIRFVQQHCG-----QMGGINGNRNTG-SGRYTLNMM-VD-IFLGAKSAKIQS----
------GIFGKGAAYSRHNVERLFRKLVLDKILDEDLYITAN-DQAV--AYVVLGEKAQA
VLNGL----L-------------Q---VEFHETENASAIRKQR----------ASVTKMS
Q--RE--E
MVKKCLGELTDTCKTLGKIFDV-HYFNIFSTSTLKKIA--ETLSSDAEVLLQIDGVTEDK
LEKYGAEIIKVMDKYSE----------------WTTPEDAACQ--SVDTAPGSA----GT
PGSEE-----EAEDDVV-------TSSYFGGNANQRRKRK--RLPNSRESKRKKTSSGGS
QQFYSKG-----------ARYRRARRAPGSR-------AAAPAQS-----------SALR
---------------------GAGAR----------------------------------

--LGIMAPP-
--KP--------------
---------SSRHFLQPSYAVL-----------------
>Q9I920


```
------------------------------------------------------------
------------------------------------------------------------
------------MEEARAAT----------NGGSGESQKL-------------------S
NGEKSSQLEP--------GDVG-NEL------LADIEL----EEDDY-LDVVP---PSPE
EELP-------SFSPSVR---------------NVSNIFKESPT-----DGRS-AVHGTE
SEPELMAPKQPAAEQDSSAEHADKGLHLEQQLYS--------------------------
----------------------VMEDICK-------------------------------
------------------------------------------LVDAIPLHELTSIS----
---------------------CAKELLQQRELR----------------------RKLLA
--DSGALNTNSVNG--P----RN----WKACVQQDPSSRPGTPLCSGP------------
-------------GRGVSSVGS--TPKSTN-----------LPSVLSRTVNSSSF-----
----------------STIRNQTLD---------------------------------KL
DTSYSSKETDQEVICLEPAALPSPKVNGKGSTSLSRPSEASFNGSWCEKPTG-RDS----
----------GNWRVPERPTASTALK----AQHTAPAGNP-----------ASGCWDV--
--NDTDFDLDHFDI---DDF--------------DEGWEEAVAPEAA-------------
--PEAPPAPQWQPLR--EG--SASLRCRLL---AAAAGS------------------APG
PHPTAPKSGCGISAKS--SSEPLV-------HNPAH-----ERFRGM-----K-FSH---
------SEEMLKIFHRKFGLHSFRTNQLEAINAALLG--EDCFIL---MPT--GGGKSLC
--------YQLPA---------------------CV----SAGV-TVVISPLRSLIIDQV
QKLKT------LDI-----------------ASTYLTGD---ITDADA---SKTYMQLSK
KDP--IIKLLYVTPE-KVCAS---------------NRLLSALENLYNRKLLARFVIDEA
HCV--SQWG------HDFRKDYKRLNMLRKKF--HS-VPMMALTATAN----------PR
VQKDIQNQLEML----KPQVFTM--SFNRHNLKYDVL---PK-------------KPKKV
AM--DCLEWIKKY-HPH----DSGIIYC-----LSRHEC--------DTT---------A
AIL---QKEGLAALA-YHAGLTDSNR-DLVQKKW----VNQEG-CQ----VICATIAFGM
GIDKPDVRYVIHASLPKS-IEGYYQESGRAG-RDGEMSHCLLF-YSYSDVTRLRRLI---
--------LMEKDG--NSHTR--------QTHFNNLYSM--VHYCENVVD----------
CRRI---QLLAYFGE--TDFNPNFCKDHPEV--ICDNC--SR----KKDYKSR--NVTDE
VKSIIRFVQQHCG-----QVGGINGNRNTG-SGRYTLNMM-VD-IFLGAKSAKIQS----
------GIFGKGAAYSRHNVERLFRKLVLDKILDEDLYITAN-DQAV--AYVVLGEKAQA
VLNGL----L-------------Q---VEFHETENASAIRKQR----------ASVTKMS
Q------------------------------------------------------RE--E
MVKKCLGELTDTCKTLGKIFDV-HYFNIFSTSTLKKIA--ETLSSDAEVLLQIDGVTEDK
LEKYGAEIIKVMDKYSE----------------WTTPEDAACQ--SVDTAPGSA----GT
PGSEE-----EAADDVV-------TSSYFGGNANQRRKRK--RLPNSGESKRKKTSSGGS
QQFYSKG-----------ARYRRARRAPGSR-------AAAPAQS-----------SALR
---------------------GAGAR----------------------------------
------------------------------------------------------------
------------------------------------------------------------
------------------------------------------------------------
------------------------------------------------------------
------------------------------------------------------------
------------------------------------------------------------
----------------------------------------------------LGIMAPP-
--------------------------------------------KP--------------
---------SSRHFLQPSYAVL-----------------
>D3KR64
```

--------------------------------MAAVPQNNLREQL---------------

--RLHS-ARGALSKVL--PARSRP--------AAFT------------------------
---------FRRTHSPAAGPA--ALRDK-------------------------DVNTSLG
SGGVARPK------------------DLRVGPPGARPALPGP-----AGSSGPTAGAP--
--PGSVSVSLP--S---SGPAAYT-------------------------GDEWDDM-DDF
D--LSGFEKKFSRPAVLSPKG-------PRTPQGRGLRVSKA-------------RADVG
PQEH-------------GSEVSCSQGS----SQGSLICL--------------GDA---G
APAAAVL-SDNGMEEARAAT----------NGGSGESQKL-------------------S
NGEKSSQLEP--------GDVG-NEL------LADIEL----EEDDY-LDVVP---PSPE
EELP-------SFSPSVR---------------NVSNIFKESPT-----DGRS-AVHGTE
SEPELMAPKQPAAEQDSSAEHADKGLHLEQQLYS--------------------------
----------------------VMEDICK-------------------------------
--LVDAIPLHELTSIS----
---------------------CAKELLQQRELR----------------------RKLLA
--DSGALNTNSVNG--P----RN----WKACVQQDPSSRPGTPLCSGP------------
-------------GRGVSSVGS--TPKSTN-----------LPSVLSRTVNSSSF-----
----------------STIRNQTLD---------------------------------KL
DTSYSSKETDQEVICLEPAALPSPKVNGKGSTSLSRPSEASFNGSWCEKPTG-RDS----
----------GNWRVPERPTASTALK----AQHTAPAGNP-----------ASGCWDV--
--NDTDFDLDHFDI---DDF--------------DEGWEEAVAPEAA-------------
--PEAPPAPQWQPLR--EG--SASLRCRLL---AAAAGS------------------APG
PHPTAPKSGCGISAKS--SSEPLV-------HNPAH-----ERFRGM-----K-FSH---
------SEEMLKIFHRKFGLHSFRTNQLEAINAALLG--EDCFIL---MPT--GGGKSLC
--------YQLPA---------------------CV----SAGV-TVVISPLRSLIIDQV
QKLKT------LDI-----------------AATYLTGD---ITDADA---SKTYMQLSK
KDP--IIKLLYVTPE-KVCAS---------------NRLLSALENLYDRKLLARFVIDEA
HCV--SQWG------HDFRKDYKRLNMLRKKF--HS-VPMMALTATAN----------PR
VQKDIQNQLEML----KPQVFTM--SFNRHNLKYDVL---PK-------------KPKKV
AM--DCLEWIKKY-HPH----DSGIIYC-----LSRHEC--------DTT---------A
AIL---QKEGLAALA-YHAGLTDSNR-DLVQKKW----VNQEG-CQ----VICATIAFGM
GIDKPDVRYVIHASLPKS-IEGYYQESGRAG-RDGEMSHCLLF-YSYSDVTRLRRLI---
--------LMEKDG--NSHTR--------QTHFNNLYSM--VHYCENVVD----------
CRRI---QLLAYFGE--TDFNPNFCKDHPEV--ICDNC--SR----KKDYKSR--NVTDE
VKSIIRFVQQHCG-----QVGGINGNRNTG-SGRYTLNMM-VD-IFLGAKSAKIQS----
------GIFGKGAAYSRHNVERLFRKLVLDKILDEDLYITAN-DQAV--AYVVLGEKAQA
VLNGL----L-------------Q---VEFHETENASAIRKQR----------ASVTKMS
Q--RE--E
MVKKCLGELTDTCKTLGKIFDV-HYFNIFSTSTLKKIA--ETLSSDAEVLLQIDGVTEDK
LEKYGAEIIKVMDKYSE----------------WTTPEDAACQ--SVDTAPGSA----GT
PGSEE-----EAADDVV-------TSSYFGGNANQRRKRK--RLPNSGESKRKKTSSGGS
QQFYSKG-----------ARYRRARRAPGSR-------AAAPAQS-----------SALR
---------------------GAGAR----------------------------------

--LGIMAPP-
--KP--------------
---------SSRHFLQPSYAVL-----------------
>F6RXI0
--------------------------------MAVIPQNNLQQQL---------------

--ERHS-AKEIQNKLN--VLRPKS--------TGFT------------------------
---------FKKKIPPGN------------------SVTHAPVLG--------DKDVNVT
ETFPLSKAFF-NAKNQQ----TSISDSSEKTSKAQQIQKFCS------NQILLNSSKN--
--TQET-SKVS--QPPTKENCVKK----ELCKDSKILETNSSFDSI-LV-NDWDDI-DDF
D--TSGNSKTFTTP----SRTHF--TKLNNTQRSKTDKNNLS--------KT---QT---
SGKK-------------IDPELLNFPS--GKSRQVELTRKELPE---------SEE---L
NSPVICL-DDY-VSEEVRNE----------DIQESHASKTHLCKER-------------D
DNEKKKYPNG--------TEQHAADE---SLGADFAEF----NEDDFDIDFIP---PSPE
EEVFSP-----SSCSSSL---------------KYFSTLKESSTCDKEKNGTK-LSDDFL
SESEKTTSLQPIQ--ETSTKHDGKLLTLQQQLFH--------------------------
----------------------VMEDICS-------------------------------
--LVDTIPIIQLKGLD----
---------------------CGNKLLQHRDLR----------------------RKLLA
--D-ANSNKSATS-PPP----LN----WIYQAN---------SHDLSVNDMTAF------
------SSLHSFQKGKSAFTGN--LVREFK-----------SLHLPSKPVSTTDCEGNFL
DKTTRTFPNLDISTGMCSTQRTTPD----------------------------------KS
ESSS-DKRNDSERISSSS-----------------QSPEPFISSNSVEISKTEEKY----
----------KRSHLLGNFLTSTAIK----DQNKHINSV----------------NDL-R
NGFSETQDIDIFDV---DDF----D---------DSDWENPN-YNSA-------------
--EEKSSKNKYLPIR--EGQPIKSFSE-RT---PMTKTN------------------CLL
MASANQNR--SISIQN--CFDKSVQNRPS--RNPEH-----EHFKSL-----D-FPH---
------TKEMMKIFHKKFGLHHFRTNQLEAINAVLLG--EDCFVL---MPT--GGGKSLC
--------YQLPA---------------------CV----SPGV-TIVISPLRSLIVDQV
QKLTS------LDI------------------PATYLTGD---KTDSEA---ANIYLQLSK
KDP--IIKLLYVTPE-KVCAS---------------NRLISTLENLYERKLLARFVIDEA
HCV--SQWG------HDFRQDYKRMNMLRQKF--PS-VPMMALTATAN----------PR
VQKDILTQLKIL----KPQVFSM--SFNRHNLKYSVL---PK-------------KPKKV
AF--DCLEWIRKH-HPH----DSGIIYC-----LSRREC--------DTM---------A
DTL---QKDGLAALS-YHAGLSDSAR-DEVQHKW----INQDG-CQ----VICATIAFGM
GIDKPDVRYVIHSSLPKS-VEGYYQESGRAG-RDGEMSHCLLF-YNYHDVTRLKRLI---
--------LMEKDG--NSHTR--------QTHFNNLYSM--VHYCENIIE----------
CRRI---QLLSYFGE--NGFNPNFCKEYPDV--TCDNC--CK----KKDYKTR--DVTED
VKNIIKFVQEHDS-----LNGERNKKMRGS-CGRFTMNML-AD-IFLGSKSAKIQS----
------GIFGKGAAYSRHNAERLLKKLILDKILDEDLYINAN-DQAI--AYVMLGEKAQA
VLNGY----L-------------K---VDFLETENFSSMRKQK----------ALVEKTS
Q---RE--E
LVKKCLGELTEVCKGLGKVFGV-HYFNIFNTVTLKKLA--ESLSSDPEVLLQIDGVTEDK
LEKYGAEVISVLQKYSE----------------WTLPATDNDS--LWLSPHNARS---NE
E--DV-----NEEETQV-------SSHYFSNKSKNERKRR--RLPTFQKAKRKKTSH-GN

QQSYSRR---------VSSTCRKA--SSKSKSSNAVGANSASYSS-----------QMTS
---------------------GASRK----------------------------------

--LGIMAPP-
--QP--------------
---------KNRPFLQPSYSFL-----------------
>G3W1C2
--------------------------------MAIIPQNNLQQQL---------------

--ERHS-AKEIQNKLN--VLKHKS--------TGFT------------------------
---------FKKKIPSSS------------------SVTQAFVLS--------DKDVNIT
ENVPLSKPLP-NTKNQQ----ASITASSEKTSKGQQIHKFCS------NQILSDSSKN--
--THET-SCYL--QPSTNENSVKK----VLCKDSKKLELNSSFDSTSLV-SDWDDI-DDF
D--TSGNSKTFTTP----SRSNF--AKLNSTQRSKGDKNNLS--------KTRARKA---
NGKK-------------VDPELLNFPS--GKNKQAELIKKELPE---------SEE---L
SSPMICL-DDDPISEEFKNE----------DIQEKQALKTQLDDER-------------D
YSEKKKSPER--------VEQHTIDE---SLGANCEEF----NEDDFDIDFIP---PSPE
EGVF----------SSSL------------------STLKEPSICDEGKNATK-LSNYFS
SESEKTTSPQLLQ--ETSTKYDGKLLTLQQQLFH--------------------------
----------------------VMEDICS-------------------------------
--LVDTIPTYQLEGLD----
---------------------CGKKLLQHRDLR----------------------RKLLA
--D-ADLNKNGAS-SSP----LN----WTYQPN---------SHDQSVNGKTVF------
------SSLHSLQTSKSFFTGN--LVKEFK-----------SLHLPSNPVSTIDCQGNFF
DKTSRTFPNLDVSTGMHDIQRTTPE---------------------------------KS
KLSS-AKRNISESILLSS-----------------QSPEPFISSNSAEISRTGEKC----
----------KRSHLLGNFLTSTTIK----DQNKHINSV----------------NDLKR
NNFSETQDIDTFDIDDFDDF----D---------DYDWENPS-YNSV-------------
--EEK-SKNMYLPIR--EGQPLKSSSE-RT---PLSKTS------------------CLS
VVPANQNS--SLSIQN--FSDKLVQNKPS--RNPEQ-----ERFKSL-----D-FPH---
------SKEMMKIFHKKFGLHHFRTNQLEAINAVLLG--EDCFVL---MPT--GGGKSLC
--------YQLPA---------------------CV----SPGV-TIVISPLRSLIIDQV
QKLTS------LDI-----------------PATYLTGD---KTDSEA---ANIYLQLSK
KDP--IIKLLYVTPE-KVCAS---------------NRLISTLENLYERKLLARFVIDEA
HCV--SQWG------HDFRQDYKRLNMLRHKF--PS-VSMMALTATAN----------PR
VQKDILTQLKIL----NPQVFSM--SFNRHNLKYSVL---PK-------------KPKKV
AF--DCLEWIRKH-HPH----DSGIIYC-----LSRREC--------DTM---------A
DTL---QKDGLAALA-YHAGLSDSAR-DEVQHKW----INQDG-CQ----VICATIAFGM
GIDKPDVRYVIHSSLPKS-VEGYYQESGRAG-RDGEMSHCLLF-YNYHDVTRLKRLI---
--------LMEKDG--NSHTR--------QTHFNNLYSM--VHYCENIIE----------
CRRI---QLLSYFGE--SGFNPNFCKEYPDV--TCDNC--CK----KKEYKTR--DVTED
VKNIIKFVQEHDS-----LNGERNKNM-GS-CGRFTMNML-TD-IFLGSKCAKIQT----

------GIFGKGAAYSRHNAERLLKKLILDKILDEDLYINAN-DQAI--AYVMLGEKAQA
VLNGY----L-------------Q---VDFLETENLSGIRKQK----------ALVKNIS
Q--RE--E
LVKKCLGELTEVCKGLGKVFGV-HYFNIFNTVTLKKLA--ESLSSDPEVLLQIDGVTEDK
LEKYGAEVISVLQKYSE----------------WTLPDNGNDS--QRLNSHND-------
---ES-----NEEETQV-------SSHYFSNKNKNERKRR--RLPTFQRSKRKKTSH-GS
QQSSSRR---------VSNTSRKR--SSKSKSSTAFGSNSKSYNS-----------QVSS
---------------------GTSRK----------------------------------

--LGIMAPP-
--IP--------------
---------KNRPFLQPSYSFL-----------------
>G3W1C3
--------------------------------MAIIPQNNLQQQL---------------

--ERHS-AKEIQNKLN--VLKHKS--------TGFT------------------------
---------FKKKIPSSS------------------SVTQAFVLS--------DKDVNIT
ENVPLSKPLP-NTKNQQ----ASITASSEKTSKGQQIHKFCS------NQILSDSSKN--
--THET-SCYL--QPSTNENSVKK----VLCKDSKKLELNSSFDSTSLV-SDWDDI-DDF
D--TSGNSKTFTTP----SRSNF--AKLNSTQRSKGDKNNLS--------KTRARKA---
NGKK-------------VDPELLNFPS--GKNKQAELIKKE-------------------
-----------------KNE----------DIQEKQALKTQLDDER-------------D
YSEKKKSPER--------VEQHTIDE---SLGANCEEF----NEDDFDIDFIP---PSPE
EGVF----------SSSLR-------------EKCINTLKEPSICDEGKNATK-LSNYFS
SESEKTTSPQLLQ--ETSTKYDGKLLTLQQQLFH--------------------------
----------------------VMEDICS-------------------------------
--LVDTIPTYQLEGLD----
---------------------CGKKLLQHRDLR----------------------RKLLA
--D-ADLNKNGAS-SSP----LN----WTYQPN---------SHDQSVNG----------
--------------NKSFFTGN--LVKEFK-----------SLHLPSNPVSTIDCQ----
--------------GMHDIQRTTPE---------------------------------KS
KLSS-AKRNISESILLSS-----------------QSPEPFISSNSAEISRTGEKC----
----------KRSHLLGNFLTSTTIK----DQNKHINSV----------------NDLKR
NNFSETQDIDTFDIDDFDDF----D---------DYDWENPS-YNSV-------------
--EEK-SKNMYLPIR--EGQPLKSSSE-RT---PLSKTS------------------CLS
VVPANQNS--SLSIQN--FSDKLVQNKPS--RNPEQ-----ERFKSL-----D-FPH---
------SKEMMKIFHKKFGLHHFRTNQLEAINAVLLG--EDCFVL---MPT--GGGKSLC
--------YQLPA---------------------CV----SPGV-TIVISPLRSLIIDQV
QKLTS------LDI-----------------PATYLTGD---KTDSEA---ANIYLQLSK
KDP--IIKLLYVTPE-KVCAS---------------NRLISTLENLYERKLLARFVIDEA
HCV--SQWG------HDFRQDYKRLNMLRHKF--PS-VSMMALTATAN----------PR
VQKDILTQLKIL----NPQVFSM--SFNRHNLKYSVL---PK-------------KPKKV

```
AF--DCLEWIRKH-HPH----DSGIIYC-----LSRREC--------DTM---------A
DTL---QKDGLAALA-YHAGLSDSAR-DEVQHKW----INQDG-CQ----VICATIAFGM
GIDKPDVRYVIHSSLPKS-VEGYYQESGRAG-RDGEMSHCLLF-YNYHDVTRLKRLI---
--------LMEKDG--NSHTR--------QTHFNNLYSM--VHYCENIIE----------
CRRI---QLLSYFGE--SGFNPNFCKEYPDV--TCDNC--CK----KKEYKTR--DVTED
VKNIIKFVQEHDS-----LNGERNKNM-GS-CGRFTMNML-TD-IFLGSKCAKIQT----
------GIFGKGAAYSRHNAERLLKKLILDKILDEDLYINAN-DQAI--AYVMLGEKAQA
VLNGY----L-------------Q---VDFLETENLSGIRKQK----------ALVKNIS
Q------------------------------------------------------RE--E
LVKKCLGELTEVCKGLGKVFGV-HYFNIFNTVTLKKLA--ESLSSDPEVLLQIDGVTEDK
LEKYGAEVISVLQKYSE----------------WTLPGSVSLW--GMSESYSDSQ---CR
CLYES-----NEEETQV-------SSHYFSNKNKNERKRR--RLPTFQRSKRKKTSH-GS
QQSSSRR---------VSNTSRKR--SSKSKSSTAFGSNSKSYNS-----------QVSS
---------------------GTSRK----------------------------------
------------------------------------------------------------
------------------------------------------------------------
------------------------------------------------------------
------------------------------------------------------------
------------------------------------------------------------
------------------------------------------------------------
----------------------------------------------------LGIMAPP-
--------------------------------------------IP--------------
---------KNRPFLQPSYSFL-----------------
>V8P6B7
------------------------------------------------------------
------------------------------------------------------------
-------MNCLDDCASGYLGGTKP--------LALK------------------------
-----------------------------------------------------DKNINSS
LEKQS------GIKNKQ----TKINVLFETTLKVQEGKQEHS-----NDQHAKNLDCF--
-------ISSV--K---ENDADCK----VGGSEIPTPDNNCLNNSFIDLTDIWEDM-EDF
E--TTGPQKICPQL----SVSFR--DKLSVNNRKTAKKPSIS--------KS---KISST
GRDN-------------EKCNDVYI----CTNRKTQTLHEEQEE---------------G
SLPVPCLDSSNSSQRDFSNG----------EREEEKSLETFQTEKK-------------E
KSWIGQNDSG--------LPLSGNEEPDDGRDDPQVFS----ESEDY-VDFIP---PSPE
EEVLPALFGLKSTSQPSL---------------HCWCLAK------------------YL
TEPCYIS---------------------GQPLLC--------------------------
----------------------VMEDICK-------------------------------
------------------------------------------LVDSIPERELKCLL----
---------------------GGQELLRLRDCR----------------------RKLLS
--NPRNGNRTGVDTFLD----------INCKHNFDYDGSHDGSYSSQLGKMSSL------
-----------LPQGGASTPGPFSGKRLGD-----------VAPLRGLHPPPPQG-----
------------------WASADPSV----------------------------SVE-DS
TWDT-SSSAVESSGVSCP-----------------RREAGGISG--LQNPNLE--A----
----------ETRWWP-STLNGTSSE----SRRKCAPLM----------------SNV-G
SESPLATGGQKLHK---DDF-DIED---------LDDLDDAV-SASS-------------
--ARDASQGPARPAV--HTSPAAKLLL---------KEN------------------SVA
SRAGADLLHSDFSFTD--HSDTPS-------RNATL-----EPFRGL-----A-FPH---
```

```
------SNEMLKIFHKKFGLHHFRTNQLEAINAALLG--QDCFIL---MPT--GGGKSLC
--------YQLPA---------------------CV----SGGV-TIVISPLRSLIVDQV
QKLTS------MDI-----------------PATYLTGD---KTDAEA---SRIYMQLSK
KDP--IIKLLYVTPE-KVCSS---------------GRLMSALENLYQRQLLARFVIDEA
HCV--SQ----------------RLNMLRKKF--SL-VPMMALTATAN----------PR
VQKDILNQLEMH----QPQVFTM--SFNRHNLKYDVL---PK-------------KPKNV
AL--DCLQWIRKY-HPW-------------------------------------------
--------GGLSALA-YHAGLPDGTR-DLVQQKW----INQDG-CQ----VICATIAFGM
GIDKPDVRFVIHASLPKS-IEGYYQESGRSG-RDGEISHCLLF-YSYSDVTRLRRLI---
--------LMEREG--NSHTR--------QTHFNNLYSM--VHYCENIME----------
CRRI---QLLAYFGE--TGFNPRFCKEHPEV--SCDNC--SK----QQVYKLK--NVTEE
VKNIVRFVKEYCG-----EKGTRNIKRN---AGRYTLNMM-ID-IFLGSKAAKIQR----
------GLFGKGAAYSRHNAERLFRKLVLDKILDEDLYITAN-DQAI--AYIQLGEKAVA
VLNGS----L-------------Q---VEFCETESASNIRRQR----------ALDTKMS
L------------------------------------------------------RE--E
MVKKCLSELTDVCKNLGKVFNI-HYFNIFNTATLKRIA----------------------
------------------------------------------------------------
-------------------------GQIREVWSRNNRCDA--EVLYKDTTRSYKACK-SA
SS------------------------NSRSSTSCVF--------------------PGQP
---------------------GAGKR----------------------------------
------------------------------------------------------------
------------------------------------------------------------
------------------------------------------------------------
------------------------------------------------------------
------------------------------------------------------------
------------------------------------------------------------
----------------------------------------------------PGMMALP-
--------------------------------------------KP--------------
---------QSRQYLKPSYMLL-----------------
>G1KPR4
--------------------------------MASLPQNNLQEQL---------------
------------------------------------------------------------
--ELYS-AKGTLNKLA--LQKPKA--------SGFT------------------------
---------FRKKTAFDNG---KTTECL--------RGMKGGPLQ--------ENDANLS
LKKANGPLLG----PKQ----AKISELFGTTQKGQEGQQSHP-----NDPSESSLGHF--
--TFSV-KERD--A---GSQLENP-----------KADHNSSYSSIINIDDVWDDI-DDF
E--IARQEKKCSKS----PASLSSNTLLAKKSKAPKNQKPLT--------SEQLVPLVSR
QGSQ-------------LCNEGFVNNK--SNTIQIPDDDDDQEE---------NAP---G
SQSVICL--GPITSHDGSYE----------DVPANRPLGSPPGSKK-------------V
ESLEIRNDKG--------EKPGSACN------DPEVCNE--SETEDY-VDFIP---PSPE
EEEA-------PSSASSF---------------KSISYIFKESAVEKT-----------R
SPQALKSSSGASS--GSRKAQEGKPFPSGQSLYS--------------------------
----------------------VMEDICK-------------------------------
------------------------------------------LVDSIPERDLRFLS----
---------------------CGSELIRHRDYR----------------------RQLLN
--NLTNGSGRDAGIPLDISSKAL----FRYDG----------SLDSGVSFRLEE------
-------------ENVDSPPG---GMSTST-----------QPSVRKFGVFERHH-----
```

------------------NFHSTPS---------------------------------VV
PAVQGTSNVSLSRFPGNS-----------------ALENGISPL----------QN----
----------ASLSFPKRGSSGERMW----SENFMNPEMN-----------VTAESDS-L
ANRAGNFDDDNFDI---DDL---------------NELDEII-QLSG-------------
--AEQPLQASCLPA------PASSMGNCSL---ASDAGR------------------GLL
SSAVLPRANQQVHAKT--GLVRMVESPSA--RNPSL-----ERFRGC-----S-FPH---
------SNEMMKVFHKRFGLHHFRTNQQEAINAALLG--EDCFVL---MPT--GGGKSLC
--------YQLPA---------------------CI----LVGV-TIVISPLRSLIVDQV
QKLTS------MDI-----------------PATYLTGD---KTDAEA---SRIYMQLSK
KDP--IIKLLYVTPE-KVCSS---------------GRLMSTLENLYQRQLLARFVIDEA
HCV--SQWG------HDFRQDYKRLNMLRKKF--AS-VPMMALTATAN----------PR
VQKDILNQLEML----KPQVFSM--SFNRHNLKYDVL---PK-------------RPKSV
AL--DCLQWIRKY-HPY----DSGIIYC-----LSRYEC--------DSM---------A
SNL---QKAGLSALA-YHAGLPDETR-DIVQQKW----INQDG-CQ----IICATIAFGM
GIDKPDVRFVIHASLPKS-IEGYYQESGRAG-RDGERSHCLLF-YSYSDVTRLRRLI---
--------LMEKDG--NSHTR--------QTHFNNLYSM--VHYCENVVE----------
CRRI---QLLAYFGE--TGFNPKFCKEYPEV--SCDNC--CK----VQDYNMK--NVTEE
VKSIVRFVQEHCG-----EKGARGARRNG---GRYTLNMM-ID-IFLGTKSAKIQT----
------GIYGKGAAYSRHNAERLFRKLVLDKILDEELYITAN-DQAV--AYVHTGERAHV
VLSGC----L-------------Q---VEFCETESASSLRKHR----------ASVTKIS
Q--RE--E
MAKECLSELTDVCKNLGKVFNV-HYFNIFNTATLKRIA--ETLSSDPEVLLQIDGVTEDK
LEKYGAEIIAVMQKYSD----------------KTLPEE---R--QPLPTTDPGK---RH
YCAEE-----AEEEMEV-------TSGYFQSRPNRGKKRK--KPQFFKESKRKKPGP-GR
HQSHAKG---------GCNGSRKA------------PPGPSLNAS-----------PVRP
---------------------GGGRR----------------------------------

--PGFMAPP-
--KP--------------
---------QSRPYLKPSYSLL-----------------
>A0A1U8DBX3
-------------MEPPLPPMQPENGSLRGSSMATIPQNNLQKQL---------------

--ELHS-AKGACSKLS--LPKHKP--------AGFT------------------------
---------FKKKTLGNAVSVGLSGAT--------------------------NLSALKE
KDVNTSLNKPIVSLPASKDKQIKINDFFVQASKEQEIQQLCE-----------------D
QVSTTSLGCLQQISNDLPAPAKKNVSSEPCDTPSQKVDYSCSADSVITIEDDWDDI-DDF
D--LSAKQKNYSKPLVLSPKSQIVKTSKTLQISNSPTNRLSRASRTVSREQGLSKPCPSE
NGVKLQSDKLPDLSKSSLIYIDLLPDDLIPNNRKKTLCEDLQEE----------------
--SVETILDDKKKDTCTA------------DGKKQNNGKVPLGEDE-------------E
DSQSLKELDK---------------------GWVETLATTGLDEDDDYLDIIP---PSPE
EVSC------------------------------ISAPKKNNSFLKESTVEGKPAVNTTW

SEPKKTTAKQPSN--GTSTENADKCLPWEQQLYN--------------------------
----------------------VMENICK-------------------------------
--LVDAVPILELKSLS----
---------------------CGNDLLWQRDCR----------------------RKLLT
--NRTDASKSGANILLAST--------WKPCPGRDTCMSDDVLLPLGQ------------
-------------KSILSPEGNSFKFTKFTSPPSGAVNKSFLCTEKFHTLDTLDT-----
---------------SDALQHIAPN-----------------------------TNIREN
TGFCFSKLDSKERTSLSF-----------------GNDMSPLSRSWCEKPGMEKT-----
----------ASWHLSKVPVTSTALK----VQNHFSAGA------------NGRAGDHAE
TTRDIDFELDNFDI---DDF--------------DEDWDNPLNDSVP-------------
------DQPLYHPIR--EGQPAKSLSKPTFTAKGINHGS---------------------
-NPAVSKPNSFMSSRN--CSDPLL-------RNPAL-----DRFRGF-----T-FSH---
------SEEMLNVFHKKFGLHSFRTNQLEAINAALLG--EDCFIL---MPT--GGGKSLC
--------YQLPA---------------------CV----SPGV-TIVISPLRSLIVDQV
QKLNT------LDI-----------------SATYLTGD---RTDGDA---SKIYMQLSK
KDP--VIKLLYVTPE-KVCAS---------------GRLISALENLYERRLLARFIIDEA
HCV--SQWG------HDFRPDYKRLNMLRKKF--HS-VPMMALTATAN----------PR
VQKDIQNQLEML----QPQVFTM--SFNRHNLKYDVL---PK-------------KPKSV
AK--DCLEWIKKH-HPY----DSGIVYC-----LSRHEC--------DST---------A
RIL---KDGGLAALA-YHAGLRDAER-DLVQKKW----INQEG-CQ----VICATIAFGM
GIDKPDVRYVIHASLPKS-VEGYYQESGRAG-RDGETSHCLLF-YSYSDVNRLRRLI---
--------LMERDG--NSHTK--------QTHFNNLYSM--VHYCENVVE----------
CRRI---QLLAYFGE--TSFNPNFCKEHPEA--TCDNC--SR----EKDYKSR--NVTDD
VKSIIRFVQEHCG-----QGGGGRRSAGNRGSGRYTLNMM-TD-IFLGVKSAKIQT----
------GIFGKGAAYSRHNAERLFRKLVLDKILDEDLYITAN-DQAV--SYVIVGERAQA
VLNGC----L-------------E---VQFHETESASSLRKQR----------ASVAKMS
E--RE--E
MVKKCLVELTDMCKTLGKIFDV-HYFNIFNTSTLKRIA--ETLSSDTEVLLQIDGVTEDK
LEKYGAEIIKVMQKYSE----------------WTLPEEDYAT-----------------
----------AGKGPGSA------ASSYFNNKTNKGKRRK--RFPPFKQAKKKKTSQGGG
QQSYSKG---------GGSKYKKTFNSKASAAPSYSSSTFGATAA---------------
--------------------PGLSRK----------------------------------

--LGIMAPP-
--KP--------------
---------QNRQFLKPSYSLL-----------------
>A0A151MTR0
--------------------MVHSAVPVISLSMATIPQNNLQKQL---------------

--ELHS--


```
------------------------------------------------------------
---AKGACSKLSLPKHKPAGFTFKKKTLAPAKKNSDRLPDLS------------------
--------------ESSLIYIDLLPDDSIPNNRKKTLCEDLQEE---------------L
SAETILDDKKEETCTA--------------DRKKQNNGKVPLGEDE-------------E
DSQSLKDLDK---------------------GWVETLATTGLDEDDDYLDIIP---PSPE
EVSC------------------------------ISAPKKTNSFLKESTVEGKPTVNTTW
SEPKKTTAKQPSN--GTSTENADKCLPWEQQLYN--------------------------
----------------------VMENICK-------------------------------
------------------------------------------LVDAVPIHELKSLS----
---------------------CGNDLLWQRDCR----------------------RKLLT
--NCTDASKSGADILLAT------------------------------------------
-------------TWKPCPGQKFHTLDTLD-----------TSDALQHIAPNTNI-----
------------------------------------------------------------
-----------------------------------RENTGFCFSKLDSRER---------
----------TSLSFGNDVSPLN--------------------------------RDHAE
TTRDIDFELDNFDI---DDF--------------DEDWDNPLNDSVP-------------
------DQPLYHPIR--EGQPTKSLSKPSFTAKGINHGS---------------------
-NPAVSKPNSFMSSRN--CSDPLL-------RNPAL-----DRFRGF-----T-FSH---
------SEEMLNVFHKKFGLHSFRTNQLEAINAALLG--EDCFIL---MPT--GGGKSLC
--------YQLPA---------------------CV----SPGV-TIVISPLRSLIVDQV
QKLNT------LDI-----------------SATYLTGD---RTDGDA---SKIYVQLSK
KDP--VIKLLYVTPE-KVCAS---------------GRLISALENLYERRLLARFIIDEA
HCV--SQWG------HDFRPDYKRLNMLRKKF--HS-VPMMALTATAN----------PR
VQKDIQNQLEML----QPQVFTM--SFNRHNLKYDVL---PK-------------KPKSV
AK--DCLEWIKKH-HPY----DSGIVYC-----LSRHEC--------DST---------A
RIL---KDGGLAALA-YHAGLRDAER-DLVQKKW----INQEG-CQ----VICATIAFGM
GIDKPDVRYVIHASLPKS-VEGYYQESGRAG-RDGETSHCLLF-YSYSDVNRLRRLI---
--------LMERDG--NSHTK--------QTHFNNLYSM--VHYCENVVE----------
CRRI---QLLAYFGE--TSFNPNFCKEHPEA--TCDNC--SR----EKDYKSR--NVTDD
VKSIVRFVQEHCG-----QGGGGRRSAGNRGSGRYTLNMM-TD-IFLGVKSAKIQT----
------GIFGKGAAYSRHNAERLFRKLVLDKILDEDLYITAN-DQAV--SYVIVGERAQA
VLNGC--------------------LEVQFHETESASSLRKQR----------ASVAKMS
E------------------------------------------------------RE--E
MVKKCLVELTDMCKTLGKIFDV-HYFNIFNTSTLKRIA--ETLSSDTEVLLQIDGVTEDK
LEKYGAEIIKVMQKYSD----------------WTLPEEDYAT-------AGKGPGSAGM
LESDE-----ESEGTGT-------TSSYFNNKTNKGKRRK--RFPPFKQAKKKKTSQGGG
QQSYSKG---------GGSKYKKTFNSKASAAPSYSSSTYGATAV---------------
--------------------PGLSRK----------------------------------
------------------------------------------------------------
------------------------------------------------------------
------------------------------------------------------------
------------------------------------------------------------
------------------------------------------------------------
------------------------------------------------------------
----------------------------------------------------LGIMAPP-
--------------------------------------------KP--------------
---------QNRQFLKPSYSLL-----------------
```

>D3ZQW1
--------------------------------MAAVPLNNLQEQL---------------

--QRHS-ARKLKNQFS--ISKPKP--------SGFT------------------------
---------FKKKTPEGD---------V--------SVVRTPVLS--------DKDVNMS
EALSYTEPPL-HKPKQQ----SNIDGFFKNSPGGQQSKETCS------ELSLPEVVQI--
--PQDA-SCTT--P---KTPAVKK----PHVAIFKKLEFSSSADS---L-SDWADI-DDF
D--VP-ASDAFVSL----TRNPT--TRVSTAKKLSSSKRNFF--------KP---PP---
R-AS-------------AVKADLTPSS--PECLQVDLTKEQEEE---------ADC---L
SRDVICI-DTDPASEELLEK----------DVQEGLSLDARLGTER-------------V
DSAKKSHEDK--------AAFQSPQN------ALYFEH----NDNDYDIDFVP---PSP-
EEII-------STASSSL---------------KCSSMLKDLDDSDKEKDILS-TSKD-L
SKPEEMTTPKSDL--GTNEDCDAQQTSLQQQLIR--------------------------
----------------------VMEHICK-------------------------------
--LVDTVPIDELKALT----
---------------------CGDKLLQQRDIR----------------------RKLLA
--E-AGFNRNDVN-LLG----SL----WRHRPD---------SLDNTV------------
-------------QCDSCPVGH--PGKELN-----------SPYLPSHSLSTEEC-----
------------------LPTTASG---------------------------------KT
GFSA-TPKSLSERPPLNS-----------------HLQKSFVSSNWAETPRTESRT----
----------ESTYFPGSVLTSTTVK----DESKHVASG----------------RDI-E
REVQASYDIDNFNI---DDF-DDDD---------DDDWENIM-HNFS-------------
--VSKSSTAAYPPIK--EGGPVKSLSE-RI---SSAKAK------------------FLP
VASTAQNKNLSESIQN--CSDKFAQNLSS--KNPKH-----EHFQSL-----N-FPH---
------TKEMMKIFRKKFGLHNFRTNQLEAINAALLG--EDCFIL---MPT--GGGKSLC
--------YQLPA---------------------CV----SPGV-TIVISPLRSLIVDQV
QKLTS------FDI-----------------PATYLTGD---KTDSEA---ANIYLQLSK
KDP--IIKLLYVTPE-KVCAS---------------NRLISTLENLYERKLLARFVIDEA
HCV--SQWG------HDFRQDYKRMNMLRQRF--PS-VPVMALTATAN----------PR
VQKDILTQLKIL----RPQVFSM--SFNRHNLKYYVL---PK-------------KPKKV
AL--DCLEWIRKH-HPY----DSGIIYC-----LSRKEC--------DTM---------A
DTL---QREGLAALA-YHAGLSDSAR-DEVQHKW----INQDN-CQ----VICATIAFGM
GIDKPDVRFVVHASLPKS-VEGYYQESGRAG-RDGEISHCVLF-YTYHDVTRLKRLI---
--------MMEKDG--NYHTR--------ETHVNNLYSM--VHYCENITE----------
CRRI---QLLAYFGE--KGFNPDFCKKHPDV--SCDNC--CK----TKDYKTK--DVTDD
VKNIVRFVQEHSS-----SPGTRNI---GP-AGRFTLNML-VD-IFLGSKSAKVQS----
------GIFGKGSAYSRHNAERLFKKLILDKILDEDLYINAN-DQPI--AYVMLGTKAHS
VLSGH----L-------------K---VDFMETENSSSVKKQK----------AAVAHVS
Q--RD--E
AVMKCLGELADVCKLLGKVFGV-HYFNIFNTATLKKLA--ESLSSDPEVLLQIDGVTEDK
LEKYGAEIIPVLQKYAE----------------WTLPAEGGSP--G-----SRGS---RR
STPEE-----TEEEEAV-------SSHYFANQTRNERKRK--KVSASQKPKRRRTSYGGG
FR--AKG---------GSTTCRKP--SSKSKFSGVTGSRSASCAS-----------QATS
---------------------AASRK----------------------------------

--LGIMAPP-
--KP--------------
---------VNRPFLRPAYAFS-----------------
>O88700
--------------------------------MAAVPLNNLQEQL---------------

--QRHS-ARKLNNQPS--LSKPKS--------LGFT------------------------
---------FKKKTSEGD----VSVTSV--------SVVKTPALS--------DKDVNVS
EAFSFTESPL-HKPKQQ----AKIEGFFKHFPGRQQSKGTCS------EPSLPATVQT--
--AQDT-LCTT--P---KTPTAKK----LPVAVFKKLEFSSSADS---L-SDWADM-DDF
D--MS-ASDAFASL----AKNPA--TRVSTAQKMKKTKRNFF--------KP---PP---
RKAN-------------AVKTDLTPPS--PECLQVDLTKESEEEEEEEEEAEGADC---L
SRDVICI-DNDSASEELTEK----------DTQESQSLKAHLGAER-------------G
DSEKKSHEDE--------AVFHSVQN------TEYFEH----NDNDYDIDFVP---PSP-
EEII-------STASSSL---------------KCSSMLKDLDDSDKEKGILS-TSEELL
SKPEEMTTHKSDA--GTSKDCDAQQIRIQQQLIH--------------------------
----------------------VMEHICK-------------------------------
--LVDTVPTDELEALN----
---------------------CGTELLQQRNIR----------------------RKLLA
--E-AGFNGNDVR-LLG----SL----WRHRPD---------SLDNTV------------
-------------QGDSCPVGH--PNKELN-----------SPYLLSHSPSTEEC-----
------------------LPTTTPG---------------------------------KT
GFSA-TPKNLFERPLLNS-----------------HLQKSFVSSNWAETPRMENRN----
----------ESTDFPGSVLTSTTVK----AQSKQAASG----------------WNV-E
RHGQASYDIDNFNI---DDFDDDDD---------DDDWENIM-HNFP-------------
--ASKSSTATYPPIK--EGGPVKSLSE-RI---SSAKAK------------------FLP
VVSTAQNTNLSESIQN--CSDKLAQNLSS--KNPKH-----EHFQSL-----N-FPH---
------TKEMMKIFHKKFGLHNFRTNQLEAINAALLG--EDCFIL---MPT--GGGKSLC
--------YQLPA---------------------CV----SPGV-TIVISPLRSLIVDQV
QKLTS------FDI-----------------PATYLTGD---KTDSEA---ANIYLQLSK
KDP--IIKLLYVTPE-KVCAS---------------NRLISTLENLYERKLLARFVIDEA
HCV--SQWG------HDFRQDYKRMNMLRQKF--PS-VPVMALTATAN----------PR
VQKDILTQLKIL----RPQVFSM--SFNRHNLKYYVL---PK-------------KPKKV
AF--DCLEWIRKH-HPY----DSGIIYC-----LSRREC--------DTM---------A
DTL---QREGLAALA-YHAGLSDSAR-DEVQHKW----INQDN-CQ----VICATIAFGM
GIDKPDVRFVIHASLPKS-MEGYYQESGRAG-RDGEISHCVLF-YTYHDVTRLKRLI---
--------MMEKDG--NYHTK--------ETHVNNLYSM--VHYCENITE----------
CRRI---QLLAYFGE--KGFNPDFCKKYPDV--SCDNC--CK----TKDYKTK--DVTDD
VKNIIRFVQEHSS-----SPGTRNI---GP-AGRFTLNML-VD-IFLGSKSAKVKS----
------GIFGKGTTYSRHNAERLFKKLILDKILDEDLYINAN-DQPI--AYVMLGTKAHS
VLSGH----L-------------K---VDFMETENSSSIKKQK----------ALVAKVS
Q--RE--E
VVKKCLGELTEVCKLLGKVFGV-HYFNIFNTATLKKLA--ESLSSDPEVLLQIDGVTEDK
LEKYGAEVIPVLQKYSE----------------WTVPAEDGSP--G-----ARGA---PE

```
DT-EE-----EEEEAPV-------SSHYFANQTRNERKRK--KMSATHKPKRRRTSY-GG
FR--AKG---------GSTTCRKT--TSKSKFYGVTGSRSASCAS-----------QATS
---------------------SASRK----------------------------------
------------------------------------------------------------
------------------------------------------------------------
------------------------------------------------------------
------------------------------------------------------------
------------------------------------------------------------
------------------------------------------------------------
----------------------------------------------------LGIMAPP-
--------------------------------------------KP--------------
---------VNRTFLRPSYAFS-----------------
>E9PZ97
-----------------------------MRIMAAVPLNNLQEQL---------------
------------------------------------------------------------
--QRHS-ARKLNNQPS--LSKPKS--------LGFT------------------------
---------FKKKTSEGD----VSVTSV--------SVVKTPALS--------DKDVNVS
EAFSFTESPL-HKPKQQ----AKIEGFFKHFPGRQQSKGTCS------EPSLPATVQT--
--AQDT-LCTT--P---KTPTAKK----LPVAVFKKLEFSSSADS---L-SDWADM-DDF
D--MS-ASDAFASL----AKNPA--TRVSTAQKMKKTKRNFF--------KP---PP---
RKAN-------------AVKTDLTPPS--PECLQVDLTKESEEEEEEEEEAEGADC---L
SRDVICI-DNDSASEELTEK----------DTQESQSLKAHLGAER-------------G
DSEKKSHEDE--------AVFHSVQN------TEYFEH----NDNDYDIDFVP---PSP-
EEII-------STASSSL---------------KCSSMLKDLDDSDKEKGILS-TSEELL
SKPEEMTTHKSDA--GTSKDCDAQQIRIQQQLIH--------------------------
----------------------VMEHICK-------------------------------
------------------------------------------LVDTVPTDELEALN----
---------------------CGTELLQQRNIR----------------------RKLLA
--E-AGFNGNDVR-LLG----SL----WRHRPD---------SLDNTV------------
-------------QGDSCPVGH--PNKELN-----------SPYLLSHSPSTEEC-----
------------------LPTTTPG---------------------------------KT
GFSA-TPKNLFERPLLNS-----------------HLQKSFVSSNWAETPRMENRN----
----------ESTDFPGSVLTSTTVK----AQSKQAASG----------------WNV-E
RHGQASYDIDNFNI---DDFDDDDD---------DDDWENIM-HNFP-------------
--ASKSSTATYPPIK--EGGPVKSLSE-RI---SSAKAK------------------FLP
VVSTAQNTNLSESIQN--CSDKLAQNLSS--KNPKH-----EHFQSL-----N-FPH---
------TKEMMKIFHKKFGLHNFRTNQLEAINAALLG--EDCFIL---MPT--GGGKSLC
--------YQLPA---------------------CV----SPGV-TIVISPLRSLIVDQV
QKLTS------FDI-----------------PATYLTGD---KTDSEA---ANIYLQLSK
KDP--IIKLLYVTPE-KVCAS---------------NRLISTLENLYERKLLARFVIDEA
HCV--SQWG------HDFRQDYKRMNMLRQKF--PS-VPVMALTATAN----------PR
VQKDILTQLKIL----RPQVFSM--SFNRHNLKYYVL---PK-------------KPKKV
AF--DCLEWIRKH-HPY----DSGIIYC-----LSRREC--------DTM---------A
DTL---QREGLAALA-YHAGLSDSAR-DEVQHKW----INQDN-CQ----VICATIAFGM
GIDKPDVRFVIHASLPKS-MEGYYQESGRAG-RDGEISHCVLF-YTYHDVTRLKRLI---
--------MMEKDG--NYHTK--------ETHVNNLYSM--VHYCENITE----------
CRRI---QLLAYFGE--KGFNPDFCKKYPDV--SCDNC--CK----TKDYKTK--DVTDD
```

VKNIIRFVQEHSS-----SPGTRNI---GP-AGRFTLNML-VD-IFLGSKSAKVKS----
------GIFGKGTTYSRHNAERLFKKLILDKILDEDLYINAN-DQPI--AYVMLGTKAHS
VLSGH----L-------------K---VDFMETENSSSIKKQK----------ALVAKVS
Q--RE--E
VVKKCLGELTEVCKLLGKVFGV-HYFNIFNTATLKKLA--ESLSSDPEVLLQIDGVTEDK
LEKYGAEVIPVLQKYSE----------------WTVPAEDGSP--G-----ARGA---PE
DT-EE-----EEEEAPV-------SSHYFANQTRNERKRK--KMSATHKPKRRRTSY-GG
FR--AKG---------GSTTCRKT--TSKSKFYGVTGSRSASCAS-----------QATS
---------------------SASRK----------------------------------

--LGIMAPP-
--KP--------------
---------VNRTFLRPSYAFS-----------------
>L9KNT5
-----------------------------------MQMDSANRNL---------------

--PASG-AKCVVARRRGLLPRRRE--------FGYW------------------------
---------FGRFGAVCE----VTLRTVETYWCDMKQVSNSPGVI--------NVSKKHT
RSIVKIKGQEWETLDQQ----IKINEFSKNAPAGQQPSRVGS------KP----SWQI--
--PQEV-LCTL--Q---NTSVTKK----PHDAAFKKLEFSSSLDSFSTI-NDWDDM-DDF
V--TSGSSKAFVTP----SKDHF--VRVSTAQKSKRAKRNFS--------KA---EL---
PKTN-------------TEKADLNSSL--SESKQVDLTREEKED---------SEC---L
SSDVICI-SDGPLSKVLVSE----------DTHESHSLETQLRDEK-------------N
NSEEKENLEE--------AELHSTEN------VPCVEL----DDDDYDIDFVP---PSPE
EEII-------STASSSL---------------KCFSVLKDLDTSDRKKSVLS-ISDDLS
SEHEKMTTQEPHP--ETSTDYDARQLSLQQQLID--------------------------
----------------------VMDHICK-------------------------------
--LVDSISADELKALD----
---------------------CGNELLQQRNIR----------------------RKLLA
--E-ADFTRYDAS-LLN----SV----WRCRPN---------LPGSLM------------
-------------EGDSCPMEN--SIKVLY-----------FLHFPSNSFSSGDC-----
------------------LLTTTLG---------------------------------KT
GVSA-TTKNPFESLLFNS-----------------HLQKSFVSNNWAKTPRIEKRN----
----------ESSYFPGNVLTSTAVK----DQNKHTASM----------------NDL-K
SETQASYDIDNFDI---DDF--DDD---------DDEWENIM-QNLA-------------
--ANKSSTAACQPAK--EGRPIKVVSE-RF---FSAKTN------------------CLP
VASTAQNKNFPESIQN--YT--

---------------------------------------VMALTATAN----------PR

VQKDILTQLKIL----RPQVFSM--SFNRHNLKYYVL---PK-------------KPKKV
AF--DCLDWIRKH-HPY----DSGIIYC-----LSRHEC--------DTM---------A
DTL---RKDGLAALA-YHAGLSDSSR-DEVQHKW----INQDG-CQ----VICATIAFGM
GIDKPDVRFVIHASLPKS-VEGYYQESGRAG-RDGEISHCLLF-YTYHDVTRLKRLI---
--------LMEKDG--NRHTR--------ETHFNNLYSM--VHYCENITE----------
CRRI---QLLAYFGE--NGFNPDFCKEYPEV--SCDNC--CK----RKDYKTR--DVTDD
VKNIVKFVQEHSS-----SQRTKNVSS----SGRFTMNML-VD-IFLGSKSAKIQS----
------GIFGKGSAYSRHNAERLFKKLILDKILDEDLYINAN-DQAI--AYVMPGNKAQT
VLNGH----L-------------K---VEFMETENSSSVKKQK----------ALVAKVS
Q--RE--E
MVKKCLGELTEVCKSLGKVFGV-HYFNIFNTATLKKLA-----------VLQIDGVTEDK
LEKYGAEVIPILQKYSE----------------WTLPAEDSSP--RGSLCSNRST---GR
DAPTE-----LDEEAPI-------SSHYFASKTRNERKRK--RMSASQRPKRRKTGY-SG
SK--AKG---------TAFTAAEF-SEPPPRGSRALSASHKYPGGSTSQPLQGIKEEATT
---------------------EKPRG----------------------------------

--TGVTADPP
QECILGSRVPGMEEPDAGSVQGPRVGHEGCVGPHIHCPEKGGSQDP--------------
---------VTFDLLKVK---------------------
>A0A1S3ETB2

-----------------------MERICR-------------------------------
--VVDTIRVDELTSLD----
---------------------CGDELLQLRDQR----------------------RKLLA
--E-VDFTRKDVS-LLS----GV----WKHRRD---------SLGISV------------
-------------GGDSCPLE---GSVELK-----------FPYLPSNSLSIGEN-----
------------------VLSTAPG---------------------------------KT
GLSA-STKELFESPSFSS-----------------PSQKAFVSSKWAETPSRERRN----
----------ESSYFPGKALTSTTVK----DLSKHPASG----------------DNL-A
REAPTSCDVDDFDI---DDF---DD---------DDDWENIM-QNLP-------------
--ASKPSTAVYPPVR--EGGPVKSLS---------AKTH------------------CLP

AASAAQRKS-SEAVQN--YADKLAQNFTS--RNVKH-----ERFQSL-----D-FPH---
------TKEMMTIFHKKFGLHNFRTNQLEAINAALLG--EDCFIL---MPT--GGGKSLC
--------YQLPA---------------------CV----SPGV-TIVISPLRSLIVDQV
QKLTS------LDI-----------------PATYLTGD---KTDGEA---ASIYLQLSK
KDP--IIKLLYVTPE-KVCAS---------------NRLISTLENLYERKLLARFVIDEA
HCV--SQWG------HDFRQDYKRMNMLRQKF--PL-VPVMALTATAN----------PR
VQKDILTQLKIL----SPQVFSM--SFNRHNLKYYVL---PK-------------KPKKV
AF--DCLEWIRKH-HPY----DSGIIYC-----LSRREC--------DTM---------A
DTL---QKDGLAALA-YHAGLSDSAR-DEVQHKW----INQDG-CQ----VICATIAFGM
GIDKPDVRFVIHASLPKS-VEGYYQESGRAG-RDGEISHCLLF-YTYHDVTRLKRLI---
--------MMEKDG--NRHTR--------ETHFNNLYSM--VHYCENITE----------
CRRI---QLLAYFGE--NGFNPHFCKKYPDV--SCDNC--SK----TKEYKTR--DVTDD
VKNIVRFVQEHSL-----SQGMRSA---GP-AGRFTMNML-VD-IFLGSKSAKIQS----
------GMYGKGAAYSRHNAERLFKKLILDKILDEDLFINAN-DQAI--AYMMPGTNAQN
VLSGH----L-------------K---L--------------------------------

----CICPLLGFL-SLGKLHGN-RK-----------------------------------
-----------LQQFEE-----------------TKSFSGQSV--SAGRGG---------

>A0A1S3EW43
--------------------------------MAALPLNNLQEQL---------------

--ERHS-AKKLTNQSS--LSKPKP--------IGFT------------------------
---------FKKKTSSDSD---ASVASV--------SVARTPVLS--------DKDVNVS
KAFSFSQPLP-CAPRQQ----AGVGSCFKSTPAGQGTGV---------KPLSPGLLRS--
--PPAV--PSG--Q---NTPAARI----PQDAAFKKLEFSSSPDSSSSP-SHWDDR-DDF
D--TSSASKAFATP----AQGRF--VRVSTAQKSRQGKRNFV--------KA---SP---
HKAL-------------AVQVDLTSS---SESQQIDLTEEQKDE---------TES---F
RSAVICI-DEDSTSDVLLNK----------DSQESHTSETHLEDER-------------G
SRDRKQLVEG--------AEPPSAQQ------GPCIEL-----DDDYDVDFVP---PSPE
EESI-------SSATSS----------------KCLSVLKDFDASDQE--------KDLL
SKPAKIAGQEPDL--ETSVDCDARRTRLQEQLIQ--------------------------
----------------------VMERICR-------------------------------
--VVDTIRVDELTSLD----
---------------------CGDELLQLRDQR----------------------RKLLA
--E-VDFTRKDVS-LLS----GV----WKHRRD---------SLGISV------------

-------------GGDSCPLE---GSVELK-----------FPYLPSNSLSIGEN-----
------------------VLSTAPG---------------------------------KT
GLSA-STKELFESPSFSS-----------------PSQKAFVSSKWAETPSRERRN----
----------ESSYFPGKALTSTTVK----DLSKHPASG----------------DNL-A
REAPTSCDVDDFDI---DDF---DD---------DDDWENIM-QNLP-------------
--ASKPSTAVYPPVR--EGGPVKSLS---------AKTH------------------CLP
AASAAQRKS-SEAVQN--YADKLAQNFTS--RNVKH-----ERFQSL-----D-FPH---
------TKEMMTIFHKKFGLHNFRTNQLEAINAALLG--EDCFIL---MPT--GGGKSLC
--------YQLPA---------------------CV----SPGV-TIVISPLRSLIVDQV
QKLTS------LDI-----------------PATYLTGD---KTDGEA---ASIYLQLSK
KDP--IIKLLYVTPE-KVCAS---------------NRLISTLENLYERKLLARFVIDEA
HCV--SQWG------HDFRQDYKRMNMLRQKF--PL-VPVMALTATAN----------PR
VQKDILTQLKIL----SPQVFSM--SFNRHNLKYYVL---PK-------------KPKKV
AF--DCLEWIRKH-HPY----DSGIIYC-----LSRREC--------DTM---------A
DTL---QKDGLAALA-YHAGLSDSAR-DEVQHKW----INQDG-CQ----VICATIAFGM
GIDKPDVRFVIHASLPKS-VEGYYQESGRAG-RDGEISHCLLF-YTYHDVTRLKRLI---
--------MMEKDG--NRHTR--------ETHFNNLYSM--VHYCENITE----------
CRRI---QLLAYFGE--NGFNPHFCKKYPDV--SCDNC--SK----TKEYKTR--DVTDD
VKNIVRFVQEHSL-----SQGMRSA---GP-AGRFTMNML-VD-IFLGSKSAKIQS----
------GMYGKGAAYSRHNAERLFKKLILDKILDEDLFINAN-DQAI--AYMMPGTNAQN
VLSGH----L-------------K---VNFMETENSSSLKKQK----------ALVAKVS
Q--RE--E
VVKKCLGELTEVCKSLGKVFGV-HYFNIFNTATLKKLA--ESLSSDPEVLLQIDGVTEDK
LEKYGAEMIPILQKYSE----------------WTLPAEDSSL--GGGPGG-RGSGRCTP
EGLDE-----EEEEAPV-------SSHYFANQTRNERKRK--KTSASQRPKRRRTSY-GG
FK--ARG---------VSTASRRT--SSKSKPSSTAGSGPATCGS-----------RAAS
---------------------GASRK----------------------------------

--LGIMAPP-
--KP--------------
---------INRPFLKPSYALS-----------------
>A0A1S3EUP5

-----------------------MERICR-------------------------------
--VVDTIRVDELTSLD----
---------------------CGDELLQLRDQR----------------------RKLLA
--E-VDFTRKDVS-LLS----GV----WKHRRD---------SLGISV------------
-------------GGDSCPLE---GSVELK-----------FPYLPSNSLSIGEN-----
------------------VLSTAPG---------------------------------KT
GLSA-STKELFESPSFSS-----------------PSQKAFVSSKWAETPSRERRN----
----------ESSYFPGKALTSTTVK----DLSKHPASG----------------DNL-A
REAPTSCDVDDFDI---DDF---DD---------DDDWENIM-QNLP-------------
--ASKPSTAVYPPVR--EGGPVKSLS---------AKTH------------------CLP
AASAAQRKS-SEAVQN--YADKLAQNFTS--RNVKH-----ERFQSL-----D-FPH---
------TKEMMTIFHKKFGLHNFRTNQLEAINAALLG--EDCFIL---MPT--GGGKSLC
--------YQLPA---------------------CV----SPGV-TIVISPLRSLIVDQV
QKLTS------LDI-----------------PATYLTGD---KTDGEA---ASIYLQLSK
KDP--IIKLLYVTPE-KVCAS---------------NRLISTLENLYERKLLARFVIDEA
HCV--SQWG------HDFRQDYKRMNMLRQKF--PL-VPVMALTATAN----------PR
VQKDILTQLKIL----SPQVFSM--SFNRHNLKYYVL---PK-------------KPKKV
AF--DCLEWIRKH-HPY----DSGIIYC-----LSRREC--------DTM---------A
DTL---QKDGLAALA-YHAGLSDSAR-DEVQHKW----INQDG-CQ----VICATIAFGM
GIDKPDVRFVIHASLPKS-VEGYYQESGRAG-RDGEISHCLLF-YTYHDVTRLKRLI---
--------MMEKDG--NRHTR--------ETHFNNLYSM--VHYCENITE----------
CRRI---QLLAYFGE--NGFNPHFCKKYPDV--SCDNC--SK----TKEYKTR--DVTDD
VKNIVRFVQEHSL-----SQGMRSA---GP-AGRFTMNML-VD-IFLGSKSAKIQS----
------GMYGKGAAYSRHNAERLFKKLILDKILDEDLFINAN-DQAI--AYMMPGTNAQN
VLSGH----L-------------K---VNFMETENSSSLKKQK----------ALVAKVS
Q--RE--E
VVKKCLGELTEVCKSLGKVFGV-HYFNIFNTATLKKLA--ESLSSDPEVLLQIDGVTEDK
LEKYGAEMIPILQKYSE----------------WTLPAEDSSL--GGGPGG-RGSGRCTP
EGLDE-----EEEEAPV-------SSHYFANQTRNERKRK--KTSASQRPKRRRTSY-GG
FK--ARG---------VSTASRRT--SSKSKPSSTAGSGPATCGS-----------RAAS
---------------------GASRK----------------------------------

--LGIMAPP-
--KP--------------
---------INRPFLKPSYALS-----------------
>H0WSN6
--------------------------------MAGLPHNNLQEQL---------------

--ERHS-ARKLNNKLS--FSKPKS--------LGFT------------------------
---------FKKKTPSDNS---ISLTSM--------S----------------D---NVT

```
EAFSVSEPPS-HTTNQQ----TKINDFYKNAPAGQQTQRVGS------KPLLPDLVQI--
--PKEV-LCTT--P---HTSVIKK----PSDAAFKKLDFSSSSDSFGAI-DNWDDM-DDF
D--TSGTSKAFVTP----LKNHF-LLRVSTAQKSKKAKRNFS--------KE---QL---
YKTD-------------TVKAGLTTSS--SENKQVDFTKGQKDD---------SER---L
SSDVICI-DDDCISEVRINE----------DTQESHSLKTHLGDER-------------D
NSGKKKNLED--------TELHSTEK------IPYTKL-----SDDYDTDFVP---PSPE
EESI-------STASSSL---------------KCFSMLQDSDNFDREKDVLS-ISKDTL
SKPEKITAQEPNP--ETSTDYDARQISVQQQLIH--------------------------
----------------------VMDHICK-------------------------------
------------------------------------------LIDTVPADKLKALD----
---------------------CGDELLLQRNVR----------------------RKLLA
--E-VDFNRSGTS-HLG----SA----WRLRPD---------PCGSPV------------
-------------EEDSSGEN---FMKELA-----------FPYLPSNPISPGEY-----
------------------LPTTALG---------------------------------KT
GFLA-TTKNHCESMLLES-----------------SLQKSFVSNNWAETPKLEKSK----
----------ESSFFPGSVLTSTAVK----DQNKNTASI----------------NDL-E
REIQASCEVDNFDI---DDF---DD---------DDDWENIM-HNLA-------------
--ASKSSTTAHQPIR--EGRPVKSVSE-RI---SSAKTN------------------CLP
VASTAQNINFSETIQS--YTDKSEQDSTY--RNVKH-----EHFQSL-----N-FPH---
------TKEMLKIFHKKFGLHNFRTNQLEAINAALLG--EDCFIL---MPT--GGGKSLC
--------YQLPA---------------------CV----SPGV-TVVISPLRSLIVDQV
QKLTS------LDI-----------------PATYLTGD---KTDSEA---TSIYLQLSK
KDP--IIKLLYVTPE-KVCAS---------------NRLISTLENLYERKLLARFVIDEA
HCV--SQWG------HDFRQDYKRMNMLRQKF--PS-VPVMALTATAN----------PR
VQKDILTQLKIL----RPQVFSM--SFNRHNLKYYVL---PK-------------KPKKV
AF--DCLEWIRKY-HPY----DSGIIYC-----LSRREC--------DTM---------A
GTL---RKDGLAALA-YHAGLSDSER-DEVQHKW----INQDG-CQ----VICATVAFGM
GIDKPDVRFVIHASLPKS-VEGYYQESGRAG-RDGEMSHCLLF-YTYHDVTRLKRLI---
--------MMEKDG--NHHTK--------ETHFNNLYSM--VHYCENITE----------
CRRI---QLLAYFGE--NGFNPDFCKKYPDV--SCDNC--CK----RKDYKIR--DVTDD
VKSIIRFVQEHSL-----SQRARNIKHIGS-AGRFTMNML-VD-IFLGSKSAKIQL----
------GIFGKGSAYSRHNAERLFKKLILDKILDEDLYINAN-DQAI--AYIMPGDKAQT
VLSGH----L-------------K---VDFMETENSSSVKKQK----------ALVAKVS
Q------------------------------------------------------RE--E
MVKKCLGELTEVCKSLGKVFGV-HYFNIFNIVTLKKLA--ESLSSDPEVLLQIDGVTEDK
LEKYGAEVIAVLQKYSE----------------WTFPGQYSKL--GRAPAPYTKG---GG
FEPSP-----GQTALLL-------PGHQWPGWTRPARKWR--RPMSSKKVRQRRPHF-RG
ST-----------------ACRKV--SSKTKSSNVIGSSSASHDS-----------QAPS
---------------------GACRK----------------------------------
------------------------------------------------------------
------------------------------------------------------------
------------------------------------------------------------
------------------------------------------------------------
------------------------------------------------------------
------------------------------------------------------------
----------------------------------------------------LGIMAPP-
--------------------------------------------KP--------------
```

---------VNRPFLKPSYAFS-----------------
>G1T6H2
--------------------------------MAAVPQNNLQEQL---------------

--ELHS-ARKLHNRLN--LSKPKS--------SGFT------------------------
---------FKKKASSGNS---ASVTSV--------SAARTPVLS--------DKDVNVT
EGSSFSEPQA-CPAGPP----ARINDFFKNAPAGLQSRGAGS------TPLLPDVSQT--
--PQEA-SCTP--R---LAPAAKT----PRDTVFKKLDFSSSLDSLGAL-NGWDDM-DDF
D--TSGTPKAFAAS----PRKPV--ARVSTAQKSKRAKRNFC--------KA---QL---
KKAD-------------AAEADLTPAS--AASKPVELAGAGKGD---------CDG---V
GGGVICIDDDDGPVSQVLIL----------DSQESHSLETHLGGER-------------D
HPEDKKVLDT--------VELHSAEE------VPCIEL----EDDDYDIDFVP---PSPE
EEVS-------SATSSSL---------------KCSSMLKDLDTPHREKDVLS-PAQDLL
SKPEKLTPQEPDP--ETTAGCEARPSPVQRRLLQ--------------------------
----------------------VMEHICV-------------------------------
--LVDAIPAEALRALE----
---------------------GGSELLRQRDTR----------------------RKLLA
--E-ADFTRNGAS-LLG----SL----WEVGPD---------SLGSPV------------
-------------DGESCSVGD--SVKAPT-----------FPHLPSNSLSNGKC-----
------------------L-TTPPG---------------------------------KT
EFSA-PTKHLSEGPSFSS-----------------HLHKSFVSGNWADTPRTGQRN----
----------GRCYFPENVLTSTAVR----DQSKHRASA----------------SDT-A
RDARAACDVDDFDI---DDF---DD---------DDDWEDIM-HSLA-------------
--ANKSSTAAYRPIR--EGGPVRSASE-RI---SSAKTS------------------CLP
MVSTAHRRHISESVQN--DADKPAQSLAS--GNLKH-----ERFQSL-----N-FPH---
------TKDMMKIFHKKFGLHNFRTNQLEAINAALLG--EDCFIL---MPT--GGGKSLC
--------YQLPA---------------------CI----SPGV-TIVISPLRSLIVDQV
QKLTS------LDI-----------------PATYLTGD---KTDSEA---ASIYLQLSK
KDP--IIKLLYVTPE-KVCAS---------------NRLISTLENLYERKLLARFVIDEA
HCV--SQWG------HDFRQDYKRMNMLRQKF--PS-VPVMALTATAN----------PR
VQKDILTQLKIV----RPQVFSM--SFNRHNLKYYVL---PK-------------KPKKV
AF--DCLEWIRKH-HPY----DSGIIYC-----LSRREC--------DTM---------A
DTL---QKDGLAALA-YHAGLSDSAR-DEVQHKW----INQDG-CQ----VICATIAFGM
GIDKPDVRFVIHASLPKS-VEGYYQESGRAG-RDGEISHCVLF-YTYHDVTRLKRLI---
--------LMEKDG--NQHTR--------ETHFNNLYSM--VHYCENITE----------
CRRI---QLLAYFGE--NGFNPNFCKKYSDV--SCDNC--CK----TKDFKTR--DVTDE
VKNIVRFVQEHSS-----SQGARPARPLGP-SGRFTMNML-VD-IFLGSKSAKIQS----
------GIFGKGSAYSRHNAERLFKKLILDKILDEELYINAN-DQAI--AYVMPGNKAQT
LLSGH----L-------------K---VDFMETENSSSVKKQK----------ALVAKVS
Q--RE--E
VVKKCLGELTEVCKSLGKVFGV-HYFNIFSTATLRRLA--ESLSSDPEVLLQIDGVTEDK
LEKYGAEVIPILQKYSE----------------WTLPAEDASP--RPSPPSRRDS---GR
SAPEA-----PEEDAPV-------SSRYFANTARNERRKR--KLPAPQRAKRRRTSH-GG
CP--AKG---------GSTARRKP--PSKTKPS-----CAAPRGS-----------PAPT
---------------------GGPGK----------------------------------

--LGLLAPP-
--KP--------------
---------VNRPFLRPSFAVC-----------------
>L5LJG2

------------MDIS--LRAAEE--------AG--------------------------
---------------------------------------EAPPTA---------------
AAFASCQPLL-------------LAE-WRSCPLWKHTDHQGA------------------
-------APAL--S---VFPLVRG----RERDR---------------------EL-ESM
R--EKHQSAASCTP----PTRDVPATKVHALDRNRTWDLSVG--------RPTLYPL---
SQTG-------------FGHRIMAAVP--QNNLQEQLERHSARK---------------L
NNKLNLS-KPKPSSEELTNE----------DTQNSHSSKTHLGEER-------------D
NSEKEKHLEE--------TEFHSLEK------SPCVQL----DEDDYDIDFIP---PSPE
EITT-------SASSSSL---------------KCF------------------------
-----------------------RQVSLQQQLIQ--------------------------
----------------------VMDRICN-------------------------------
--LVDAIPDDELKALD----
---------------------CGDDLLQQRNRR----------------------RKLLA
--E-ADFNPSDAS-VLG----SM----WRCRPD---------ALGDPMEDMASV------
------SLPYAFSKGDSCPTG---SSDELH-----------FPPIPSNSISSGGC-----
------------------LV-TPPG---------------------------------KT
GFSV-TKKN-FERPLFNS-----------------HLQKSFVSSNWAETPRTEKRS----
----------ERSHFPGSVLTSTAVK----DQNKHTAST----------------DDL-E
REIQASCEIDNFDI---DDL---DD---------DDEWENIM-HGLA-------------
--TNKSSTAAYPPIK--EGRPVKSVSK-RI---SPTKTD------------------CLP
VTPTAQNKNFSGSIQKCRFGDKLRRAISGLVELTVW-----VSHLGL-----N-FPH---
------TKEMMKIFHKKFGLHHFRTNQLEAINAALLG--EDCFIL---MPT--GGGKSLC
--------YQLPA---------------------CV----SPGV-TIVISPLRSLIVDQV
QKLTS------LDI-----------------PATYLTGD---KTDSEA---TSIYLQLSK
KDP--IIKLLYVTPE-KVCAS---------------NRLISTLENLYERKLLARFVIDEA
HCV--SQWG------HDFRQDYKRMNMLRQKF--PS-VPVMALTATAN----------PR
VQKDILTQLKIL----RPQVFTM--SFNRHNLKYYVL---PK-------------KPKKV
AF--DCLEWIRKH-HPH----DSGIIYC-----LSRREC--------DTM---------A
DTL---QKNGLPALA-YHAGLSDSAR-DEVQHKW----INQDG-CQ----VICATIAFGM
GIDKPDVRFVIHASLPKS-VEGYYQESGRAG-RDGEISHCLLF-YTYYDVTRLKRLI---
--------LMEKDG--NHHTR--------ETHFNNLYSM--VHYCENITE----------
CRRI---QLLAYFGE--IGFNPDFCKKHPEV--SCDNC--CQ----SKDYKTR--DVTGD
VKNIVRFVQEHSS-----SQGMGNRNHGGP-CGRFTMNML-VD-IFLGSKSAKIQS----
------GIFGKGSTYSRHNAERLFKKLILDKILDEDLFINAN-DQPI--AYVMLGSKAQA
LLNGH----L-------------K---VDFMETENSSRIKKQK----------ALVTKVS
Q--RE--E
VVKKCLGELTEVCKSLGKVFGV-HYFNIFNTVTLKKLA--ESLSSDPEVLLQIDGVTEDK

```
LEKYGAEVIPILQKYSE----------------WTLPVEDSSP--QMSPSSSRSA----R
NGSEE-----CDEETPI-------SSHYFANKTKNERKRK--RMPACQRSKRRRTG--GD
FK--TKG---------RSTTCRRI--SPKTKSSSIPGPTSTARGS-----------QAAA
---------------------AVSRK----------------------------------
------------------------------------------------------------
------------------------------------------------------------
------------------------------------------------------------
------------------------------------------------------------
------------------------------------------------------------
------------------------------------------------------------
----------------------------------------------------LGIMAPP-
--------------------------------------------KP--------------
---------VNRPFLRPTYAFS-----------------
>W5PTL9
--------------------------------MAAVPQNNLQEQL---------------
------------------------------------------------------------
--ERHS-ARRLNNKLS--LSKPKS--------SGFT------------------------
---------FKKKVPSAND---VSVTSV--------SVAKTPVLS--------DKDVNIT
EAFSFNEPLP-HTTNQQ----MRIND-FESAPSGPQTRKVGS------KPLLPSLSQA--
--PQEV-SCTA--Q---NTPVIKQ----SHDATFKKLEFSSSSDSLIPF-NDWDDM-DDF
D--TSGNSKAFVTP----YKSHF--IRVSTAQKSKKSKRNLV--------NA---QL---
NKTN-------------TGKTDLAPSS--SESKQEHLTEKQEDD---------SES---L
SSDVICI-DDDPDFEELINE----------DTQENHSLKTHLGAER-------------G
NCEKKKNLEE--------TELHSVEK------SSCVEL----IEDDYDIDFVP---PSPE
EGMT-------STSSSSL---------------KCSSMWKDLDTSDKK-DGLS-TS-D-L
SKLKEMATQQPNR--ETSTSCEARQTSLQQQLIH--------------------------
----------------------VMDRICK-------------------------------
------------------------------------------LVDAVPDDELRALD----
---------------------CGDELLQQRDIR----------------------RKLLA
--E-AAFDPIDAS-VLS----SM----WGCRPD---------PLESPLGDSASA------
------SLPYAFPKGDSCPAGN--AVKEFT-----------LPHLPSNSMSTRER-----
------------------LLTPLLG---------------------------------KT
GVSA-TTKSPFERPLFSS-----------------HLQKSFVSSNWAETPRAEKRN----
----------ESPNFPRNVLTSTAVK----DQNKYTTSV----------------NDL-E
RELQAACEIDNFDI---DDF--DDD---------DDDWENLM-QNLA-------------
--ASKSSTAAYPPIK--EGRPVKSVSE-RP---SSAKTD------------------CLP
GASTTQNKNFSESIPN--YPDKMEQNLAS--RNPKH-----ERFQSL-----N-FPH---
------TKEMMKIFHKKFGLHNFRTNQLEAINAALLG--EDCFIL---MPT--GGGKSLC
--------YQLPA---------------------CV----SPGV-TIVISPLRSLIVDQV
QKLTS------LDI-----------------PATYLTGD---KTDSEA---TSIYLQLSK
KDP--IIKLLYVTPE-KVCAS---------------NRLISTLENLYERGLLARFVIDEA
HCV--SQWG------HDFRPDYKRMNILRQKF--PS-VPVMALTATAN----------PR
VQKDILTQLKIL----RPQVFSM--SFNRHNLKYYVL---PK-------------KPKKV
AF--DCLEWIRKY-HPH----DSGIIYC-----LSRREC--------DTM---------A
ETL---QNDGLAALA-YHAGLSDSAR-DEVQHKW----INQDG-CQ----VICATIAFGM
GIDKPDVRFVIHASLPKS-VEGYYQESGRAG-RDGEISHCILF-YAYHDVTRLKRLI---
--------LMEKDG--NRHTR--------ETHFNNLYSM--VHYCENITE----------
```

CRRI---QLLAYFGE--NEFNPHFCKKYPDV--SCDNC--CK----TKDFKTK--DVTDD
VKNIVRFVQEHSS-----SQGTRNTKHIGP-SGRFTMNML-VD-IFLGSKSAKIQS----
------GIFGKGSTYSRHNAERLFKKLILDRILDEDLYINAN-DQPI--AYVMLGSKAQT
VLNGH----L-------------K---VDFMETENSSSVKKQK----------ALVAKVS
Q--RE--E
VVKKCLRELTEVCKSLGKVFGV-HYFNIFNTVTLKKLA--ESLSSDPEVLLQIDGVTEDK
LEKYGAEVIPILQKYSE----------------WTLPAEDTSP--RRSPSSSRGT---GK
NTPEE-----SDEEASV-------SSRYFANKTRNERKRK--RMSASQRAKRRRTGF-GG
SR--TKG---------GSNTCRRI--PSKTKPSNVIGPSSTLPGS-----------QAAS
---------------------GVSRK----------------------------------

--LGIMAPP-
--KP--------------
---------INRPFLKPSYAFS-----------------
>E1BQ04
--------------------------------MAAVPQNNLQEQL---------------

--ERHS-ARKLNNKLG--LSKPKS--------SGFT------------------------
---------FKKKVPSANN---VSVTSV--------SVAKTPVLS--------DKDVNIT
EAFSFSEPLP-HTTNQQ----TGIND-FKSAPSGPQTKRVGS------KPLLPSLSQV--
--PQEV-SCTA--Q---NTPVIKQ----SLNATFKKLEFSSSSDSLIAF-NDWDDM-DDF
D--TSGNSKAFVTP----CKNHF--VRVSTAQKSKKSKRNLV--------KA---QL---
NKTH-------------TGETDLAPSS--SESKQEHLTEKQKDD---------SEW---L
SSDVICI-DDDPDPEELINE----------DTQENHSLKTHLGAER-------------G
NCEKKKNLEE--------TELRSVEK------SSCIEL----DEDDYDIDFVP---PSPE
EGMT-------STSSSSL---------------KCSSMLKDLDTSDKK-HSLS-TSED-L
SKLKEMATQQPNQ--ETSTSCEARQTSLQQQLIR--------------------------
----------------------VMDRICK-------------------------------
--LVDAVPDDELKALD----
---------------------CGNELLQQRDIR----------------------RKLLA
--E-ADFDPSDAS-VLS----AM----WGCRPD---------PLESPV------------
-------------KGDSCLAGN--AVKEFT-----------LPHLPSNSMSTRER-----
------------------LLTPPLG---------------------------------KT
GVLA-TTKSPFERPLFSS-----------------HLQKSFVSSNWAETPRAEKRN----
----------ESPDFPGNVLTSTAVK----DQNKYTTSI----------------NDL-E
RELQAACEIDNFDI---DDF--DDD---------DDDWENLM-QNLA-------------
--ASKSSTAAYPPIK--EGRPVKSVSE-RP---SSAKTD------------------CLP
GASTTQNKNFSESIPN--YPDKMEQNLAS--RNPKH-----ERFQSL-----N-FPH---
------TKEMMKIFHKKFGLHNFRTNQLEAINAALLG--EDCFIL---MPT--GGGKSLC
--------YQLPA---------------------CV----SPGV-TIVISPLRSLIVDQV
QKLTS------LDI-----------------PATYLTGD---KTDSEA---TSIYLQLSK
KDP--IIKLLYVTPE-KVCAS---------------NRLISTLENLYERKLLARFVIDEA

HCV--SQWG------HDFRPDYKRMNILRQKF--PS-VPVMALTATAN----------PR
VQKDILTQLKIL----RPQVFSM--SFNRHNLKYYVL---PK-------------KPKKV
AF--DCLEWIRKH-HPH----DSGIIYC-----LSRREC--------DTM---------A
ETL---QNDGLAALA-YHAGLSDSAR-DEVQHKW----INQDG-CQ----VICATIAFGM
GIDKPDVRFVIHASLPKS-VEGYYQESGRAG-RDGEISHCILF-YTYHDVTRLKRLI---
--------LMEKDG--NRHTR--------ETHFNNLYSM--VHYCENITE----------
CRRI---QLLAYFGE--NEFNPHFCKKYPDV--SCDNC--YK----TKDFKTK--DVTED
VKNIVRFVQEHSS-----SQGTRNIKHIGP-SGRFTMNML-VD-IFLGSKSAKIQS----
------GIFGKGSTYSRHNAERLFKKLILDRILDEDLYINAN-DQAI--AYVMLGSKAQT
VLNGH----L-------------K---VDFMETENSSSVKKQK----------ALVAKVS
Q--RE--E
VVKKCLGELTEVCKSLGKVFGV-HYFNIFNTVTLKKLA--ESLSSDPEVLLQIDGVTEDK
LEKYGAEVIPILQKYSE----------------WTLPAEDTSP--RRSPSSSRGT---RK
NTPEE-----SDEGAPI-------SSHYFANKTRNERKRK--RMPASQKAKRRRTGF-SG
SR--TKG---------GSNTCRRI--PSKTKPSNIIGPSSTSHSS-----------QAAS
---------------------GVSRK----------------------------------

--LGIMAPP-
--KP--------------
---------INRPFLKPSYAFS-----------------
>L8J1R8
--------------------------------MAAVPQNNLQEQL---------------

--ERHS-ARKLNNKLS--LSKPKS--------SGFT------------------------
---------FKKKVPSAND---VSVTSV--------SVAKTPVLS--------DKDVNIT
EAFSFSEPLP-HTTNQQ----TGIND-FKSAPSGPQTKRVGS------KPLLPSLSQV--
--PQEV-SCTA--Q---NTPVIKQ----SLNATFKKLEFSSSSDSLIAF-NDWDDM-DDF
D--TSGNSKAFVTP----CKNHF--VRVSTAQKSKKSKRNLV--------KA---QL---
NKTH-------------TGETDLAPSS--SESKQELLTEKQKDD---------SEW---L
SSDVICI-DDDPDPEELINE----------DTQENHSLKTHLGAER-------------G
NCEKKKNLEE--------TELHSVEK------SSCIEL----DEDDYDIDFVP---PSPE
EGMT-------STSSSSL---------------KCSSMLKDLDTSDKK-HSLS-TSED-L
SKPKEMATQQPNR--ETSTSCEARQTSLQQQLIR--------------------------
----------------------VMDRICK-------------------------------
--LVDAVPDDELKALD----
---------------------CGNELLQQRDIR----------------------RKLLA
--E-ADFDPSDAS-VLS----AM----WGCRPD---------PLESPVKDLASV------
------SLPYAFRKGDSCLAGN--AVKEFT-----------LPHLPSNSMSTRER-----
------------------LLTPPLG---------------------------------KT
GVLA-TTKSPFERPLFSS-----------------HLQKSFVSSNWAETPRAEKRN----
----------ESPDFPGNVLTSTAVK----DQNKHTTSI----------------NDL-E
RELQAACEIDNFDI---DDF--DDD---------DDDWENLM-QNLA-------------

--ASKSSTAAYPPIK--EGRPVKSVSE-RP---SSAKTD------------------CLP
GASTTQNKNFSESIPN--YPDKMEQNLAS--RNPKH-----ERFQSL-----N-FPH---
------TKEMMKIFHKKFGLHNFRTNQLEAINAALLG--EDCFIL---MPT--GGGKSLC
--------YQLPA---------------------CV----SPGV-TIVISPLRSLIVDQV
QKLTS------LDI-----------------PATYLTGD---KTDSEA---TSIYLQLSK
KDP--IIKLLYVTPE-KVCAS---------------NRLISTLENLYERKLLARFVIDEA
HCV--SQWG------HDFRPDYKRMNILRQKF--PS-VPVMALTATAN----------PR
VQKDILTQLKIL----RPQVFSM--SFNRHNLKYYVL---PK-------------KPKKV
AF--DCLEWIRKH-HPH----DSGIIYC-----LSRREC--------DTM---------A
ETL---QNDGLAALA-YHAGLSDSAR-DEVQHKW----INQDG-CQ----VICATIAFGM
GIDKPDVRFVIHASLPKS-VEGYYQESGRAG-RDGEISHCILF-YTYHDVTRLKRLI---
--------LMEKDG--NRHTR--------ETHFNNLYSM--VHYCENITE----------
CRRI---QLLAYFGE--NEFNPHFCKKYPDV--SCDNC--YK----TKDFKTK--DVTED
VKNIVRFVQEHSS-----SQGTRNIKHIGP-SGRFTMNML-VD-IFLGSKSAKIQS----
------GIFGKGSTYSRHNAERLFKKLILDRILDEDLYINAN-DQAI--AYVMLGSKAQT
VLNGH----L-------------K---VDFMETENSSSVKKQK----------ALVAKVS
Q--RE--E
VVKKCLGELTEVCKSLGKVFGV-HYFNIFNTVTLKKLA--ESLSSDPEVLLQIDGVTEDK
LEKYGAEVIPILQKYSE----------------WTLPAEDTSP--RRSPSSSRGT---RK
NTPEE-----SDEEAPI-------SSHYFANKTRNERKRK--RMPASQKAKRRRTGF-SG
SR--TKG---------GSNTCRRI--PSKTKPSNIIGPSSTSHSS-----------QAAS
---------------------GVSRK----------------------------------

--LGIMAPP-
--KP--------------
---------INRPFLKPSYAFS-----------------
>A0A1S3W399
--------------------------------MAAIPQNNLQEQL---------------

--ERHS-ARRLNNTLG--LSKPKS--------SGFT------------------------
---------FRKKVSSASS--------V--------SVARTPVLS--------DKDVNAA
EAFSSSELSP-HAWRQQ----AALSG-FSHSPAGPQTPKAGS------EPSSADLLHT--
--PQGA-FCTT--Q---NTPAVKA----SRGSTMKKLEVSSSSDSFLLP-NEWDDM-DDF
D--TSGNSKAFVTP----PKNHF--VRVSTAQKSKKSKRNFF--------KA---PR---
PKAN-------------KVEINLTLS---SESSHIHSAKEQKGA---------LEW---S
HSGVICI-DDDSQHEEFLKE----------ETRGSRSPETHSEIER-------------G
RSEKKNTELE-------------FEE------SSCVEL----DEEDYDIDFVP---PSPE
EEII-------SSSSSSL---------------HCISILKDLDTSEGKKD----TAED-L
SKPEQTTSQQFTQ--DTSTDNNARQTSLQQQLIC--------------------------
----------------------VMEHICN-------------------------------
--LVDAIPAGELRALG----
---------------------CGDELLQQRDIR---------------------RKLLA

--E-VDLGTCDAS-VPD----AV----WRCRPR---------PHGSPVEDVVSAS-----
------SSPFAFSGGKYCPTGR--SAKEFN-----------SPHLALTPLPASES-----
------------------LLTITPA---------------------------------KT
GFSATTTKTLPERPLLGS-----------------HLQKSLVSDSWAETPSTDQRS----
----------ERSYFPGKVLTSTAVK----DQNKHNASM----------------NDS-E
TELQACCDIDNFDI---DGF---DD---------DDDWEDIM-HNLT-------------
--ANKSSTAAYHPIK--EGRPVKSVSE-RI---SSAKTN------------------CLP
VIATTQSKNFSESIQN--YPDKSTQNLAS--KNLKH-----EHFQSL-----S-LPH---
------TKEMMKIFHKKFGLHHFRTNQLEAINAALLG--EDCFIL---MPT--GGGKSLC
--------YQLPA---------------------CV----SPGV-TIVISPLRSLIVDQV
QKLTS------LDI-----------------PATYLTGD---RTDSEA---TSIYLQLSK
KEP--IIKLLYVTPE-KVCAS---------------NRLISTLENLYERKLLARFVIDEA
HCV--SQWG------HDFRQDYKRMNMLRQKF--PS-VPVMALTATAN----------PR
VQKDILTQLKIL----TPQVFSM--SFNRHNLKYYVL---PK-------------KPKKV
AF--DCLEWIRKH-HPH----DSGIIYC-----LSRREC--------DTM---------A
DTL---QKDGLAALA-YHAGLSDAAR-DEVQHKW----INQDG-CQ----VICATIAFGM
GIDKPDVRFVIHASLPKS-VEGYYQESGRAG-RDGEMSHCLLF-YTYHDVTRLKRLI---
--------LMEKDG--NHHTR--------QTHFNNLYSM--VHYCENITE----------
CRRI---QLLAYFGE--NGFNPDFCKKYPEV--SCDNC--CK----TKDFKTR--DVTDD
VKNIVQFVKEHSS-----SQERGNIMHVGP-AGRFTMNML-VD-IFLGSKSAKVQS----
------GIFGKGSTYSRHNAERLFKKLVLDNILDEDLHINAN-DQAI--AYVMPGSKAQA
VLSGH----L-------------K---VDFMETENSSSVKKQK----------TLVAKVS
K--RE--E
IVKKCLGELTEVCKSLGKVFGV-HYFNIFNTATLKKLA--ESLSSDPEVLLQIDGVTEDK
LEKYGAEVIPILQKYSE----------------WTLPAEDACP--RVSPPSSRGS---RR
NDPEE-----SDEESPV-------SSHYFTNRTKNERKRK--RIPAFQKSKRRKSSL-GG
SR--TKG---------GSTTCRKS--SSRTKPSSISGPSSAVHGS-----------QAVS
---------------------GANRK----------------------------------

--LGIMAPP-
--KP--------------
---------LNRPFLKPSFTLL-----------------
>G1PQL6
--------------------------------MAAVPQNNLQEQL---------------

--ERHS-ARKLNNKLN--LSKPKP--------SCFT------------------------
---------FKKKVSSANN---VSVTCV--------SVAKTPVLS--------DKDVNVS
EDFSFSETLP-HTMNPQ----TRISD-FKNAAAGQQAKRIGS------KPLLPDSSQI--
--PREV-SLAT--Q---STPSRKE----FRDATFKKLEYSSSSD--------WDDM-DDF
D--TSGNSKAFVTP----LKNHS--VRVSTAQ--KKSKRNFL--------KA---QL---
QKED-------------TVKVDLTTPP--SECKQVDLTEEQKAD---------SEWLSSL
SSGVICI-DDGPSSEELANE----------DTQNSHSLKTHLGEER------------D

NSEKEKHLGE--------TEFHSLKK------SPPVQL----DEDDYDIDFIP---PSPE
EITT-------SASSSSL---------------KCFSMLKELDTCDRKKDGLS-TPED-L
SKPEKMPTQQSNQ--ETSRDCDGRQISLQQQLIQ--------------------------
----------------------VMDHICN-------------------------------
--LVDAIPDDELKALD----
---------------------CGNDLLQQRNIR----------------------RKLLA
--E-ADFNPSDAS-VLG----SM----WRCRPD---------ALGNPME-----------
--------------GDSCPTA---SSDELH-----------FPPIPSNSISSGGC-----
------------------LL-TPPG---------------------------------KT
GFSV-TKKNLFERPLFNS-----------------HLQKSFVSSNWAETPRTEKRN----
----------ESSHFPGSVLTSTAVK----DQNKHTVST----------------DDL-E
REIQASCEIDNFDI---DDL---DD---------DDEWENIM-HGLA-------------
--TNKSSTAAYPPIK--EGRPVKSVSE-RI---SPTKTN------------------CLP
VTPAAQNKNFSGSMQK--CKYQSTQNLPS--RNLNH-----EHFQRL-----N-FPH---
------TKEMMKIFHKKFGLHHFRTNQLEAINAALLG--EDCFIL---MPT--GGGKSLC
--------YQLPA---------------------CV----SPGV-TIVISPLRSLIVDQV
QKLTS------LDI-----------------PATYLTGD---KTDSEA---TSIYLQLSK
KDP--IIKLLYVTPE-KVCAS---------------NRLISTLENLYERKLLARFVIDEA
HCV--SQWG------HDFRQDYKRMNMLRQKF--PS-VPVMALTATAN----------PR
VQKDILTQLKIL----RPQVFTM--SFNRHNLKYYVL---PK-------------KPKKV
AF--DCLEWIRKH-HPH----DSGIIYC-----LSRREC--------DTM---------A
DTL---QRNGLPALA-YHAGLSDSAR-DEVQHKW----INQDG-CQ----VICATIAFGM
GIDKPDVRFVIHASLPKS-VEGYYQESGRAG-RDGEISHCLLF-YTYYDVTRLKRLI---
--------LMEKDG--NHHTR--------ETHFNNLYSM--VHYCENITE----------
CRRI---QLLAYFGE--IGFNPDFCKKYSEV--SCDNC--CQ----SKDYKTR--DVTDD
VKNIVRFVQEHSS-----SQGTGNRNHIGP-CGRFTMNML-VD-IFLGSKSAKIQS----
------GIFGKGSTYSRHNAERLFKKLILDKILDEDLFINAN-DQPI--AYVMLGSKAQA
LLNGH----L-------------K---VDFMETENSSRIKKQK----------ALVTKVS
Q--RE--E
VVKKCLGELTEVCKSLGKVFGV-HYFNIFNTVTLKKLAGTESLSSDPEVLLQIDGVTEDK
LEKYGAEVIPILQKYSE----------------WTLPVEDSSP--QMSPASSRSA----R
NGSEE-----CDEETPI-------SSHYFANKTKNERKRK--RMPACQRSKRRRTGV-GD
FK--TKG---------RSTTCRKI--SPKTKSSSIPGPTSTARGS-----------QAAA
---------------------GISRK----------------------------------

--LGIMAPP-
--KP--------------
---------VNRPFLRPTYAFS-----------------
>S7ND63
----------MQIREAGNSPGSGLGRSLQPRIMAAVPQNNLQEQL---------------

--ERHS-ARKLNNKLN--LSKPKP--------SCFT------------------------

```
---------FKKKVSSANN---VSVTCV--------SVAKTPVLS--------DKDVNVS
EDFSFSETLP-HTMNPQ----TRVKD-FKNAAAGQQAKRIGS------KPLLPDSLQI--
--PREV-SLAT--Q---STPSRKE----FRDATFKKLEYSSSSD--------WDDM-DDF
D--TSGNSKAFVTP----LKNHS--VRVSTAQ--KKSKRNFL--------KA---QL---
QKED-------------TVKVDLTTPS--SECKQVDLTEEQKAD---------SEWLSSL
SSGVICI-DDGPSSEELKNE----------DTQNSHSLKTHLGEER-------------D
NSEKEKHLGE--------TEFHSLKK------SPCVQL----DEDEYDIDFIP---PSPE
EITT-------SASSSSL---------------KCFSMLKELDTCDRKKDGLS-TPED-L
SKPEKMPTQQSNQ--ETSRDCDGRQISLQQQLIQ--------------------------
----------------------VMDHICN-------------------------------
------------------------------------------LVDAIPDDELKALD----
---------------------CGNDLLQQRNIR----------------------RKLLA
--E-ADFNPSDAN-VLG----SM----WRCRPD---------ALGDPMEDMPSV------
------SLPYAFSKGDSCPTG---SSDELH-----------FPPIPSNSISSGGC-----
------------------LV-TPPG---------------------------------KT
GFSV-TKKNLFERPLFNS-----------------HLQKSFVSSNWAETPRTEKRN----
----------ESSRFPGSVLTSTAVK----DQNKHTVST----------------DDL-E
REIQASCEIDNFDI---DDL---DD---------DDEWENIM-HGLA-------------
--TNKSSTAAYPPIK--EGRPVKSVSE-RI---SPTKTN------------------CLP
VTPAAQNKNFSGSMQ----KYQSTQNLPS--RNLNH-----EHFQRL-----N-FPH---
------TKEMMKIFHKKFGLHHFRTNQLEAINAALLG--EDCFIL---MPT--GGGKSLC
--------YQLPA---------------------CV----SPGV-TIVISPLRSLIVDQV
QKLTS------LDI-----------------PATYLTGD---KTDSEA---TSIYLQLSK
KDP--IIKLLYVTPE-KVCAS---------------NRLISTLENLYERKLLARFVIDEA
HCV--SQWG------HDFRQDYKRMNMLRQKF--PS-VPVMALTATAN----------PR
VQKDILTQLKIL----RPQV----------------------------------------
------------------------------------REC--------DTM---------A
DTL---QKNGLPALA-YHAGLSDSAR-DEVQHKW----INQDG-CQ----VICATIAFGM
GIDKPDVRFVIHASLPKS-VEGYYQESGRAG-RDGEISHCLLF-YTYYDVTRLKRLI---
--------LR--------------------------------------------------
------------------------------------------------------------
------------------------------------------------SKSAKIQS----
------GIFGKGSTYSRHNAERLFKKLILDKILDEDLFINAN-DQPI--AYVMLGSKAQA
LLNGH----L-------------K---VDFMETENSSRIKKQK----------ALVTKVS
Q------------------------------------------------------RE--E
VVKKCLGELTEVCKSLGKVFGV-HYFNIFNTVTLKKLA--ESLSSDPEVLLQIDGVTEDK
LEKYGAEVIPILQKYSE----------------WTLPVEDSSP--QMSPSSSRSA----R
NGSEE-----CDEETPI-------SSHYFANKTKNERKRK--RMPACQRSKRRRTGV-GD
FK--TKG---------RSTTCRKI--SPKTKSSSIPGPTSTARGS-----------QAAA
---------------------GISRK----------------------------------
------------------------------------------------------------
------------------------------------------------------------
------------------------------------------------------------
------------------------------------------------------------
------------------------------------------------------------
------------------------------------------------------------
----------------------------------------------------LGIMAPP-
```

--KP--------------
---------VNRPFLRPTYAFS-----------------
>H0UWZ2
--------------------------------MAAVPLNNLQEQL---------------

--ERHS-ARKLNNQSS--LSKPKS--------CGFT------------------------
---------FKKKTSSDND---ASITSV--------SVAKAPVLS--------DKDVNVS
EVFSFTEPPP-HTPNQQ----TKVNGFFKNATSGQQGQGLGS------KPLLVDLLKT--
--SQEA-LPAT--R---NTSAIKK----PYDAVLKKLEFSSSQDSFCTM-DDWDDL-DDF
D--TSETSKVFATP----PKNHL--IRVSTAQKSKKAKRIFV--------KA---PP---
HKTS-------------IVKADLTQSE--SQP-VVDLTKEEKHD---------LKW---L
KSSMICI-DDDSVPQVLTSE----------DTQESKSLN-RLGDER-------------D
NNE-KHNEED--------VELYSTEK------ATCVEH----NDDDY-IDFVP---PSPE
EQSI-------SASSSSL---------------KCFSTLKDLDASDHE-ENVN-TSKDFL
SKPEKLTTQEPNP--EISMDCDARPISLQQQLLL--------------------------
----------------------VMEHICK-------------------------------
--LVDTIPLDELKALD----
---------------------CGDELLQQRNRR----------------------RKLLS
--E-ADFARNDAN--LD----SV----WKHNPD---------ILGSPV------------
-------------EDDCCPAES--SIKGPH-----------VPHLPSNSLFSREC-----
------------------LLNTTPG---------------------------------KT
GFSA-TSSTPFERPLFSS-----------------HLQKSFVSSNWAETPRVEKRN----
----------ESSYFPGNVLTSTAVK----DQKKHAASV----------------NNL-E
RESQASPDVDNFNI---DDF---DD---------DDDWEKIM-QDLT-------------
--ANKTSTAACQPIQ--KGGPLKSISE-KI---ALAKMH------------------SLP
GASV---------VPH--HTDKLTQNLAS--RNTSQ-----ERFQSL-----S-FPH---
------TKEMMKIFHKKFGLHNFRTNQLEAINATLLG--EDCFIL---MPT--GGGKSLC
--------YQLPA---------------------CV----LPGV-TIVISPLRSLIVDQV
QKLTS------LDI-----------------PATYLTGD---KTDSEA---ASIYLQLSK
KDP--IIKLLYVTPE-KVCAS---------------NRLISILENLYERKLLARFVIDEA
HCV--SQWG------HDFRPDYKRMNMLRQKF--PS-VPVMALTATAN----------SR
VQTDILTQLKIL----RPQVFSM--SFNRHNLKYYVL---PK-------------KPKKV
AF--DCLEWIRKH-HPY----DSGIIYC-----LSRWEC--------DTM---------A
DTL---QKDGLAALA-YHAGLSDAAR-DEVQHKW----INQDG-CQ----VICATIAFGM
GIDKPDVRFVIHASLPKS-VEGYYQESGRAG-RDGEISHCLLF-YTYHDVTRLKRLI---
--------MMEKDG--NRHTK--------ETHFNNLYSM--VHYCENITE----------
CRRI---QLLAYFGE--NGFNPEFCRKHPDV--SCDNC--CK----TKDYKTR--DVTGD
VKNIIKFIQEHSS-----LQGARSAKYVGPSSGRFTMNML-VD-IFLGSKSAKVQS----
------GIFGKGSTYSRHNAERLFKKLILDKILDEDLYINAN-DQPV--AYLMLGTKAPA
VLNGQ----L-------------K---VDFMETENSSSIKKQK----------ALEAKVS
Q--RE--E
VVKKCLGELTEVCKSLGKVFGV-HYFNIFNTATLKKLA--ESLSSDPEVLLQIDGVTEDK
LEKYGAEVIPVLQKYSQ----------------WTLPAEDTSP--GISLSGNSGS---RR
KAPEE-----LEDEIPV-------SSHYFANRARNERKRK--KMPASQRPKRRKTSF-GG
FK--AKG---------GSTTCRRM--SSKSKFSNTIGSSSALHSS-----------QAAS
---------------------GTSRK----------------------------------

```
------------------------------------------------------------
------------------------------------------------------------
------------------------------------------------------------
------------------------------------------------------------
------------------------------------------------------------
----------------------------------------------------LGIMAPP-
--------------------------------------------KP--------------
---------INRPFLRPSYAFS-----------------
>G1M5K3
--------------------------------MAAVPQNNLQEQL---------------
------------------------------------------------------------
--ERHS-ARKLNNKLS--PSKPKS--------LGFT------------------------
---------FKKKITSANN---VSVTSV--------SVAKTPVLN--------DKDVNVA
EAVSFSGPPP-LTTSQQ----TRPSD-FRHAPAGQQTRRAVS------KPLSPDVLQV--
--QRGV-LGNT--Q---NTPTVKK----SSNATFKKLEFSSSSDSFVTI-NDWDDM-DDF
D--TSGNSKAFVTP----CRNHF--VRVSTAQKSKKSKRNFL--------KA---QL---
PEAN-------------TVTADLTPSS--SESKQACLTKKQNND---------SEW---F
SNDVICI-DDDPNPEELIHG----------DAQESQPLKTHLGEER-------------D
GSE-KKNLGE--------TELHSVVK------SPCVEL----DEDDYETDFVP---PSPE
EEVI-------SASSSSL---------------KCFSMLKDLDSS-----GPS-TSED-L
PTPEGM-APQPDQ--EASTDCDARQTSLQQQLIH--------------------------
----------------------VMDQICK-------------------------------
------------------------------------------LVDTIPDDELKALD----
---------------------CGDELRQQRDIR----------------------RNLLA
--D-ANFNTSDAS-RLA----SV----WRCWPD---------SLGSPVDDT---------
--------------GESCPAGS--SVQELN-----------FPHLPSHCGTTREC-----
------------------LLATTPG---------------------------------KT
GFSA-TPQNPFERPLFSP-----------------HLQKSFVSSNWAETPRIEKRN----
----------ESSYFPGNVLTSTAVK----DQNKRAAPI----------------DDL-E
RETQAACDIDNFDI---DGF-DDDD---------DDDWENII-HDLA-------------
--ASKSSTAASQPIK--EGRPVKPVSE-RI---SSAKAN------------------CLP
VASTAQDKN-SESVQN--YTDKSAQNLAS--RNLKH-----ERFQSL-----S-FPH---
------TKEMMKIFHKKFGLHNFRTNQLEAINAALLG--EDCFIL---MPT--GGGKSLC
--------YQLPA---------------------CV----SPGV-TIVISPLRSLIVDQV
QKLTS------LDI-----------------PATYLTGD---KTDSEA---TSIYLQLSK
KDP--IIKLLYVTPE-KVCAS---------------NRLISTLENLYERKLLARFVIDEA
HCV--SQWG------HDFRQDYKRMNMLRQKF--PS-VPVMALTATAN----------PR
VQKDILTQLKIL----QPQVFSM--SFNRHNLKYYVL---PK-------------KPKKV
AF--DCLEWIRKH-HPH----DSGIIYC-----LSRREC--------DTM---------A
DTL---QKDGLAALA-YHAGLSDSAR-DEVQHKW----INQDG-CQ----VICATIAFGM
GIDKPDVRFVIHASLPKS-VEGYYQESGRAG-RDGEISHCLLF-YTYHDVTRLKRLI---
--------LMEKEG--NHHTR--------ETHINNLYSM--VHYCENITE----------
CRRI---QLLAYFGE--KGFNPDFCKKYPDV--SCDNC--CR----TKDYKTR--DVTND
VKNIVRFVQEHSS-----SQGIRNKNHPGP-SGRFTMNML-VD-IFLGSKSAKIQS----
------GIFGKGSTYSRHNAERLFKKLILDKILDEDLYINAN-DQPI--AYVMPGNKAQT
VLSGH----L-------------K---VDFMETENSTSVKKQK----------ALVAKIS
Q------------------------------------------------------RE--E
```

```
VVKKCLGELTEVCKSLGKVFGV-HYFNIFNTVTLKKLA--ESLSSDPEVLLQIDGVTEDK
LEKYGAEVIPILQKYSE----------------WTLPAADGSP--RRSPGSSRAT----R
SAPEE-----FDEETPV-------ASHYFANKTKNERKRK--RMSASQRPKRRRTSF-GG
SK--TKG---------GSTTCRRT--SSKSKASNIFGPHSSVLGS-----------QAAS
---------------------GATRK----------------------------------
------------------------------------------------------------
------------------------------------------------------------
------------------------------------------------------------
------------------------------------------------------------
------------------------------------------------------------
------------------------------------------------------------
----------------------------------------------------LGIMAPP-
--------------------------------------------KP--------------
---------INRPFLKPSYAFS-----------------
>J9PB86
--------------------------------MAAVPQNNLQEQL---------------
------------------------------------------------------------
--ERHS-ARKLSNKLS--PSKPKS--------SVFT------------------------
---------FKKK-TSANN---VSVTSV--------SVAKTPVLN--------DKDVNVT
EAFPFSEPLP-HATSQQ----TRPSD-LKNTPAGQQTRRTVS------KPLLPDLSLV--
--SR-V-LCNT--Q---NTPIVKK----SSNATFKKLEFSSSSDSFVTI-NDWDDM-DDF
D--TSGNSK-LVTP----CKNHF--VRVSTAQKSKKPKRNFL--------KA---QL---
PQAN-------------TVKADLTPSS--FESKQACLTKKQNDD---------SEW---F
SNDVICI-DDDPNPEELIHG----------DPQESHSLKTHLEEER-------------D
-SE-KKNWEE--------TELCSLEK------SPCVEL----DEDDYDTDFVP---PSP-
-EVI-------SASSSSL---------------KCFSMLKDLDASGTE-DGPS-TSED-L
PTPKGMTTQQPNQ--ETSTNCDARQPSLQQQLIH--------------------------
----------------------VMDQICK-------------------------------
------------------------------------------LVDTIPDDELKALT----
---------------------CGNALHQQRDIR----------------------RKLLA
--E-ADFNTSDAS-VLA----SV----WTRGPD---------SLGSPVEDMASL------
------SAPDVFPKGNSHPTGN--SVKELN-----------FPHLPSHSSSTGEC-----
------------------LLATTPG---------------------------------KT
GFSG-TMKNPSERLLFSP-----------------HLQKSFVSSNWAETPRIEKRN----
----------ESSYFLGNVLTSTAVR----DQNKRAAPI----------------NDL--
-EIQAACDIDNFDI---DGF---DD---------DDDWEDIM-HDLA-------------
--ASRSSTAACLPIK--EGRPVKPVSE-RI---SSAKTN------------------CLP
VASTAQNKN-SESIQN--YTDKSAQNLAS--RNLKH-----ERFQSL-----S-FPH---
------TKEMMKIFHKKFGLHNFRTNQLEAINAALLG--EDCFIL---MPT--GGGKSLC
--------YQLPA---------------------CV----SPGV-TIVISPLRSLIVDQV
QKLTS------LDI-----------------PATYLTGD---KTDSEA---TSIYLQLSK
KDP--IIKLLYVTPE-KVCAS---------------NRLLSTLENLYERKLLARFVIDEA
HCV--SQWG------HDFRQDYKRMNMLRQKF--PS-VPVMALTATAN----------PR
VQKDILTQLKIL----QPQVFSM--SFNRHNLKYYVL---PK-------------KPKKV
AF--DCLEWIRKY-HPH----DSGIIYC-----LSRREC--------DTM---------A
DTL---QKDGLAALA-YHAGLSDSAR-DEVQHKW----INQDG-CQ----VICATIAFGM
GIDKPDVRFVIHASLPKS-VEGYYQESGRAG-RDGEISHCLLF-YTYHDVTRLKRLI---
```

```
--------LMEKDG--NRHTR--------ETHFNNLYSM--VHYCENITE----------
CRRI---QLLAYFGE--SGFNPDFCKKYPDV--SCDNC--CK----TKDYKTR--DVTDD
VKNIVRFVQEHSS-----SQGTRIKNQLGP-SGRFTMNML-VD-IFLGSKSAKIQS----
------GIFGQGSTYSRHNAERLFKKLILDKILDEDLYINAN-DQPI--AYVMPGNKAQT
VLNGH----L-------------K---VDFIETENSTSIKKQKA---------ALVAKMS
Q------------------------------------------------------RE--E
VVKKCLGELTEVCKSLGKVFGV-HYFNIFNTVTLKKLA--ESLSSDPEVLLQIDGVTEDK
LEKYGAEVIPILQKYSE----------------WTLPADDGSP--RISPSSSRST---RR
SAPEE-----FDEETSV-------SSHYFANKTKNERKRK--RMSATQRSKRRRTGF-GG
SK--TKG---------GSTACRKT--SSKSKSSNIFGPHSSLHGS-----------QAAS
---------------------GAARK----------------------------------
------------------------------------------------------------
------------------------------------------------------------
------------------------------------------------------------
------------------------------------------------------------
------------------------------------------------------------
------------------------------------------------------------
----------------------------------------------------LGIMAPP-
--------------------------------------------KP--------------
---------INRPFLKPSYAFS-----------------
>E2RS76
--------------------------------MAAVPQNNLQEQL---------------
------------------------------------------------------------
--ERHS-ARKLSNKLS--PSKPKS--------SVFT------------------------
---------FKKK-TSANN---VSVTSV--------SVAKTPVLN--------DKDVNVT
EAFPFSEPLP-HATSQQ----TRPSD-LKNTPAGQQTRRTVS------KPLLPDLSLV--
--SR-V-LCNT--Q---NTPIVKK----SSNATFKKLEFSSSSDSFVTI-NDWDDM-DDF
D--TSGNSK-LVTP----CKNHF--VRVSTAQKSKKPKRNFL--------KA---QL---
PQAN-------------TVKADLTPSS--FESKQACLTKKQNDD---------SEW---F
SNDVICI-DDDPNPEELIHG----------DPQESHSLKTHLEEER-------------D
-SE-KKNWEE--------TELCSLEK------SPCVEL----DEDDYDTDFVP---PSP-
-EVI-------SASSSSL---------------KCFSMLKDLDASGTE-DGPS-TSED-L
PTPKGMTTQQPNQ--ETSTNCDARQPSLQQQLIH--------------------------
----------------------VMDQICK-------------------------------
------------------------------------------LVDTIPDDELKALT----
---------------------CGNALHQQRDIR----------------------RKLLA
--E-ADFNTSDAS-VLA----SV----WTRGPD---------SLGSPVE-----------
--------------GNSHPTGN--SVKELN-----------FPHLPSHSSSTGEC-----
------------------LLATTPG---------------------------------KT
GFSG-TMKNPSERLLFSP-----------------HLQKSFVSSNWAETPRIEKRN----
----------ESSYFLGNVLTSTAVR----DQNKRAAPI----------------NDL--
-EIQAACDIDNFDI---DGF---DD---------DDDWEDIM-HDLA-------------
--ASRSSTAACLPIK--EGRPVKPVSE-RI---SSAKTN------------------CLP
VASTAQNKN-SESIQN--YTDKSAQNLAS--RNLKH-----ERFQSL-----S-FPH---
------TKEMMKIFHKKFGLHNFRTNQLEAINAALLG--EDCFIL---MPT--GGGKSLC
--------YQLPA---------------------CV----SPGV-TIVISPLRSLIVDQV
QKLTS------LDI-----------------PATYLTGD---KTDSEA---TSIYLQLSK
```

KDP--IIKLLYVTPE-KVCAS---------------NRLLSTLENLYERKLLARFVIDEA
HCV--SQWG------HDFRQDYKRMNMLRQKF--PS-VPVMALTATAN----------PR
VQKDILTQLKIL----QPQVFSM--SFNRHNLKYYVL---PK-------------KPKKV
AF--DCLEWIRKY-HPH----DSGIIYC-----LSRREC--------DTM---------A
DTL---QKDGLAALA-YHAGLSDSAR-DEVQHKW----INQDG-CQ----VICATIAFGM
GIDKPDVRFVIHASLPKS-VEGYYQESGRAG-RDGEISHCLLF-YTYHDVTRLKRLI---
--------LMEKDG--NRHTR--------ETHFNNLYSM--VHYCENITE----------
CRRI---QLLAYFGE--SGFNPDFCKKYPDV--SCDNC--CK----TKDYKTR--DVTDD
VKNIVRFVQEHSS-----SQGTRIKNQLGP-SGRFTMNML-VD-IFLGSKSAKIQS----
------GIFGQGSTYSRHNAERLFKKLILDKILDEDLYINAN-DQPI--AYVMPGNKAQT
VLNGH----L-------------K---VDFIETENSTSIKKQKA---------ALVAKMS
Q--RE--E
VVKKCLGELTEVCKSLGKVFGV-HYFNIFNTVTLKKLA--ESLSSDPEVLLQIDGVTEDK
LEKYGAEVIPILQKYSE----------------WTLPADDGSP--RISPSSSRST---RR
SAPEE-----FDEETSV-------SSHYFANKTKNERKRK--RMSATQRSKRRRTGF-GG
SK--TKG---------GSTACRKT--SSKSKSSNIFGPHSSLHGS-----------QAAS
---------------------GAARK----------------------------------

--LGIMAPP-
--KP--------------
---------INRPFLKPSYAFS-----------------
>B0M1M9
--------------------------------MAAVPQNNLQEQL---------------

--ERHS-ARKLNNKLS--LSKPKS--------SGFT------------------------
---------FKKKAPSAKD---LSITSV--------SVAKTPVLS--------DKDVNVT
EAFSFSEPLS-HTTDQH----KRIND-FKNTPAGQQTKRVSS-----QQPLLPDLSQV--
--PQEV-LCTS--Q---NTPVIKE----SRDATFKKLEFSSSSDSCITF-NDWDDM-DDF
D--TSGNSKTFVTP----LRNNF--VRVSTAQKSKKSKRNFL--------KS---QF---
DKIN-------------TVKTDLIPPS--SESKQAYLTKKQKDD---------SEW---L
SSDVICIDDDDPNSEEHINE----------DTQENRSLKTHLEEKR-------------D
NCEKEKSLEE--------TELHSVEK------SSCIEL----NEDDYDIDYVP---PSPE
EGMT-------STSSSSL---------------KCSSMLKDLDTSDRR-DGLG-TSED-L
SEPEKMTSQQPNP--ETSTDCEAGQINVQQQLIR--------------------------
----------------------VMDHICK-------------------------------
--LLDTIPDDKLKALD----
---------------------CGNELLQQRDMR----------------------RKLLA
--E-ADFNTCDAS-ALA----LL----WRCRPA---------SLGSPV---ASV------
------SSPSAFPNGSSCPTGN--SVKELA-----------FPHCLSNSFSSGEH-----
------------------LLTTTPG---------------------------------KT
GFSA-TTKTVFERPPFNS-----------------HLQKSFVSSNWAETPRVEKRN----
----------ESSYFPGNVLTSTAVK----DQNKHTTSI----------------NDL-E

REIQASCDIDNFDI---DDF--DDD---------DDDWENMM-HNLA-------------
--ASKSSTAVYQPIK--EGRPVKSVSE-RI---SSAKTD------------------SLP
VASTAQNKNFSESIHN--YTDKSEQNLTS--RNLKH-----KHFQSL-----N-FPH---
------TKEMMKIFHKKFGLHNFRTNQLEAINAALLG--EDCFIL---MPT--GGGKSLC
--------YQLPA---------------------CV----SSGV-TIVISPLRSLIVDQV
QKLTS------LDI------------------PATYLTGD---KTDSEA---TSIYLQLSK
KDP--IIKLLYVTPE-KVCAS---------------NRLISTLENLYNRKLLARFVIDEA
HCV--SQWG------HDFRPDYKRMNMLRQKF--PS-VPVMALTATAN----------PR
VQKDILTQLKIL----RPQVFTM--SFNRHNLKYYVL---PK-------------KPKKV
AF--DCLEWIRKH-HPH----DSGIIYC-----LSRREC--------DTM---------A
DTL---QKDGLAALA-YHAGLSDSAR-DEVQHKW----INQDG-CQ----VICATIAFGM
GIDKPDVRFVIHASLPKS-VEGYYQESGRAG-RDGEISQCLLF-YTYHDVTRLKRLI---
--------LMEKDG--NHHTK--------ETHFNNLYSM--VHYCENIAE----------
CRRI---QLLAYFGE--NEFNPNFCKKYPDV--SCDNC--CK----TKDYKTR--DVTDD
VKNIVRFVQEHSS-----SRGARNI---GS-SGRFTMNML-VD-IFLGSKSAKLQS----
------GIFGKGSAYSRHNAERLFKKLILDKILDEDLYINAN-DQPI--AYIMLGDKAQA
VLNGH----L-------------K---VDFMETENSSSVKKQK----------ALVAKVS
Q---RE--E
MVKKCLGELTEVCKSLGKVFGV-HYFNIFNTTTLKKLA--ESLSSDPEVLLQIDGVTEDK
LEKYGAEVIPILQKYSE----------------WTVPAEDNSP--RRSPSSCRGS---GR
NAPEE-----LDEETLI-------SSHYFANKTKNERKRK--RIPASQRSKRRKTSF-GG
SK--AKG---------GSGTSRRI--SSKTKPSNVFGSRSALLGS-----------QAAS
---------------------GASRK----------------------------------

--LGIMAPP-
---KP---------------
---------INRPFLKPSYAFS-----------------
>A0A287AKX6
-------------------------MRETGIMAAIPQNNLQEQL---------------

--ERHS-ARKLNNKLS--LSKPKS--------SGFT------------------------
---------FKKKAPSAKD---LSITSV--------SVAKTPVLS--------DKDVNVT
EAFSFSEPLS-HTTDQH----KRIND-FKNTPAGQQTKRVSS-----QQPLLPDLSQV--
--PQEV-LCTS--Q---NTPVIKE----SRDATFKKLEFSSSSDSCITF-NDWDDM-DDF
D--TSGNSKTFVTP----LRNNF--VRVSTAQKSKKSKRNFL--------KS---QF---
DKIN-------------TVKTDLIPPS--SESKQAYLTKKQKDD---------SEW---L
SSDVICIDDDDPNSEEHINE----------DTQENRSLKTHLEEKR-------------D
NCEKEKSLEE--------TELHSVEK------SSCIEL----NEDDYDIDYVP---PSPE
EGMT-------STSSSSL---------------KCSSMLKDLDTSDRR-DGLG-TSED-L
SEPEKMTSQQPNP--ETSTNCEAGQINVQQQLIR-------------------------
---------------------VMDHICK-------------------------------
--LLDTIPDDKLKALD----

---------------------CGNELLQQRDMR----------------------RKLLA
--E-ADFNTCDAS-ALA----LL----WRCRPA---------SLGSPV---ASV------
------SSPSAFPNGNSCPTGN--SVKELA-----------FPHCLSNSFSSGEH-----
------------------LLTTTPG---------------------------------KT
GFSA-TTKTVFERPPFNS-----------------HLQKSFVSSNWAETPRVEKRN----
----------ESSYFPGNVLTSTAVK----DQNKHTTSI----------------NDL-E
REIQASCDIDNFDI---DDF--DDD---------DDDWENMM-HNLA-------------
--ASKSSTAVYQPIK--EGRPVKSVSE-RI---SSAKTD------------------SLP
VASTAQNKNFSESIHN--Y---
--T--GGGKSLC
--------YQLPA---------------------CV----SSGV-TIVISPLRSLIVDQV
QKLTS------LDI-----------------PATYLTGD---KTDSEA---TSIYLQLSK
KDP--IIKLLYVTPE-KVCAS---------------NRLISTLENLYNRKLLARFVIDEA
HCV--SQWG------HDFRPDYKRMNMLRQKF--PS-VPVMALTATAN----------PR
VQKDILTQLKIL----RPQVFTM--SFNRHNLKYYVL---PK-------------KPKKV
AF--DCLEWIRKH-HPH----DSGIIYC-----LSRREC--------DTM---------A
DTL---QKDGLAALA-YHAGLSDSAR-DEVQHKW----INQDG-CQ----VICATIAFGM
GIDKPDVRFVIHASLPKS-VEGYYQESGRAG-RDGEISQCLLF-YTYHDVTRLKRLI---
--------LMEKDG--NHHTK--------ETHFNNLYSM--VHYCENIAE----------
CRRI---QLLAYFGE--NEFNPNFCKKYPDV--SCDNC--CK----TKDYKTR--DVTDD
VKNIVRFVQEHSS-----SRGARNI---GS-SGRFTMNML-VD-IFLGSKSAKLQS----
------GIFGKGSAYSRHNAERLFKKLILDKILDEDLYINAN-DQPI--AYIMLGDKAQA
VLNGH----L-------------K---VDFMETENSSSVKKQK----------ALVAKVS
Q--RE--E
MVKKCLGELTEVCKSLGKVFGV-HYFNIFNTTTLKKLA--ESLSSDPEVLLQIDGVTEDK
LEKYGAEVIPILQKYSE----------------WTVPAEDNSP--RRSPSSCRGS---GR
NAPEE-----LDEETLI-------SSHYFANKTKNERKRK--RIPASQRSKRRKTSF-SG
SK--AKG---------GSGTSRRI--SSKTKPSNVFGSRSALLGS-----------QAAS
---------------------GASRK----------------------------------

--LGIMAPP-
--KP--------------
---------INRPFLKPSYAFS-----------------
>F1RMJ3
--------------------------MRETGIMAAIPQNNLQEQL---------------

--ERHS-ARKLNNKLS--LSKPKS--------SGFT------------------------
---------FKKKAPSAKD---LSITSV--------SVAKTPVLS--------DKDVNVT
EAFSFSEPLS-HTTDQH----KRIND-FKNTPAGQQTKRVSS-----QQPLLPDLSQV--
--PQEV-LCTS--Q---NTPVIKE----SRDATFKKLEFSSSSDSCITF-NDWDDM-DDF
D--TSGNSKTFVTP----LRNNF--VRVSTAQKSKKSKRNFL--------KS---QF---
DKIN-------------TVKTDLIPPS--SESKQAYLTKKQKDD---------SEW---L

```
SSDVICIDDDDPNSEEHINE----------DTQENRSLKTHLEEKR-------------D
NCEKEKSLEE--------TELHSVEK------SSCIEL----NEDDYDIDYVP---PSPE
EGMT-------STSSSSL---------------KCSSMLKDLDTSDRR-DGLG-TSED-L
SEPEKMTSQQPNP--ETSTNCEAGQINVQQQLIR--------------------------
----------------------VMDHICK-------------------------------
------------------------------------------LLDTIPDDKLKALD----
---------------------CGNELLQQRDMR----------------------RKLLA
--E-ADFNTCDAS-ALA----LL----WRCRPA---------SLGSPV---ASV------
------SSPSAFPNGNSCPTGN--SVKELA-----------FPHCLSNSFSSGEH-----
------------------LLTTTPG---------------------------------KT
GFSA-TTKTVFERPPFNS-----------------HLQKSFVSSNWAETPRVEKRN----
----------ESSYFPGNVLTSTAVK----DQNKHTTSI----------------NDL-E
REIQASCDIDNFDI---DDF--DDD---------DDDWENMM-HNLA-------------
--ASKSSTAVYQPIK--EGRPVKSVSE-RI---SSAKTD------------------SLP
VASTAQNKNFSESIHN--YTDKSEQNLTS--RNLKH-----KHFQSL-----N-FPH---
------TKEMMKIFHKKFGLHNFRTNQLEAINAALLG--EDCFIL---MPT--GGGKSLC
--------YQLPA---------------------CV----SSGV-TIVISPLRSLIVDQV
QKLTS------LDI-----------------PATYLTGD---KTDSEA---TSIYLQLSK
KDP--IIKLLYVTPE-KVCAS---------------NRLISTLENLYNRKLLARFVIDEA
HCV--SQWG------HDFRPDYKRMNMLRQKF--PS-VPVMALTATAN----------PR
VQKDILTQLKIL----RPQVFTM--SFNRHNLKYYVL---PK-------------KPKKV
AF--DCLEWIRKH-HPH----DSGIIYC-----LSRREC--------DTM---------A
DTL---QKDGLAALA-YHAGLSDSAR-DEVQHKW----INQDG-CQ----VICATIAFGM
GIDKPDVRFVIHASLPKS-VEGYYQESGRAG-RDGEISQCLLF-YTYHDVTRLKRLI---
--------LMEKDG--NHHTK--------ETHFNNLYSM--VHYCENIAE----------
CRRI---QLLAYFGE--NEFNPNFCKKYPDV--SCDNC--CK----TKDYKTR--DVTDD
VKNIVRFVQEHSS-----SRGARNI---GS-SGRFTMNML-VD-IFLGSKSAKLQS----
------GIFGKGSAYSRHNAERLFKKLILDKILDEDLYINAN-DQPI--AYIMLGDKAQA
VLNGH----L-------------K---VDFMETENSSSVKKQK----------ALVAKVS
Q------------------------------------------------------RE--E
MVKKCLGELTEVCKSLGKVFGV-HYFNIFNTTTLKKLA--ESLSSDPEVLLQIDGVTEDK
LEKYGAEVIPILQKYSE----------------WTVPAEDNSP--RRSPSSCRGS---GR
NAPEE-----LDEETLI-------SSHYFANKTKNERKRK--RIPASQRSKRRKTSF-SG
SK--AKG---------GSGTSRRI--SSKTKPSNVFGSRSALLGS-----------QAAS
---------------------GASRK----------------------------------
------------------------------------------------------------
------------------------------------------------------------
------------------------------------------------------------
------------------------------------------------------------
------------------------------------------------------------
------------------------------------------------------------
----------------------------------------------------LGIMAPP-
--------------------------------------------KP--------------
---------INRPFLKPSYAFS-----------------
>F1RMJ2
--------------------------MRETGIMAAIPQNNLQEQL---------------
------------------------------------------------------------
```

```
--ERHS-ARKLNNKLS--LSKPKS--------SGFT------------------------
---------FKKKAPSAKD---LSITSV--------SVAKTPVLS--------DKDVNVT
EAFSFSEPLS-HTTDQH----KRIND-FKNTPAGQQTKRVSS-----QQPLLPDLSQV--
--PQEV-LCTS--Q---NTPVIKE----SRDATFKKLEFSSSSDSCITF-NDWDDM-DDF
D--TSGNSKTFVTP----LRNNF--VRVSTAQKSKKSKRNFL--------KS---QF---
DKIN-------------TVKTDLIPPS--SESKQAYLTKKQKDD---------SEW---L
SSDVICIDDDDPNSEEHINE----------DTQENRSLKTHLEEKR-------------D
NCEKEKSLEE--------TELHSVEK------SSCIEL----NEDDYDIDYVP---PSPE
EGMT-------STSSSSL---------------KCSSMLKDLDTSDRR-DGLG-TSED-L
SEPEKMTSQQPNP--ETSTNCEAGQINVQQQLIR--------------------------
----------------------VMDHICK-------------------------------
------------------------------------------LLDTIPDDKLKALD----
---------------------CGNELLQQRDMR----------------------RKLLA
--E-ADFNTCDAS-ALA----LL----WRCRPA---------SLGSPV---ASV------
------SSPSAFPNGNSCPTGN--SVKELA-----------FPHCLSNSFSSGEH-----
------------------LLTTTPG---------------------------------KT
GFSA-TTKTVFERPPFNS-----------------HLQKSFVSSNWAETPRVEKRN----
----------ESSYFPGNVLTSTAVK----DQNKHTTSI----------------NDL-E
REIQASCDIDNFDI---DDF--DDD---------DDDWENMM-HNLA-------------
--ASKSSTAVYQPIK--EGRPVKSVSE-RI---SSAKTD------------------SLP
VASTAQNKNFSESIHN--YTDKSEQNLTS--RNLKH-----KHFQSL-----N-FPH---
------TKEMMKIFHKKFGLHNFRTNQLEAINAALLG--EDCFIL---MPT--GGGKSLC
--------YQLPA---------------------CV----SSGV-TIVISPLRSLIVDQV
QKLTS------LDI-----------------PATYLTGD---KTDSEA---TSIYLQLSK
KDP--IIKLLYVTPE-KVCAS---------------NRLISTLENLYNRKLLARFVIDEA
HCV--SQWG------HDFRPDYKRMNMLRQKF--PS-VPVMALTATAN----------PR
VQKDILTQLKIL----RPQVFTM--SFNRHNLKYYVL---PK-------------KPKKV
AF--DCLEWIRKH-HPH----DSGIIYC-----LSRREC--------DTM---------A
DTL---QKDGLAALA-YHAGLSDSAR-DEVQHKW----INQDG-CQ----VICATIAFGM
GIDKPDVRFVIHASLPKS-VEGYYQESGRAG-RDGEISQCLLF-YTYHDVTRLKRLI---
--------LMEKDG--NHHTK--------ETHFNNLYSM--VHYCENIAE----------
CRRI---QLLAYFGE--NEFNPNFCKKYPDV--SCDNC--CK----TKDYKTR--DVTDD
VKNIVRFVQEHSS-----SRGARNI---GS-SGRFTMNML-VD-IFLGSKSAKLQS----
------GIFGKGSAYSRHNAERLFKKLILDKILDEDLYINAN-DQPI--AYIMLGDKAQA
VLNGH----L-------------K---VDFMETENSSSVKKQK----------ALVAKVS
Q------------------------------------------------------RE--E
MVKKCLGELTEVCKSLGKVFGV-HYFNIFNTTTLKKLA--ESLSSDPEVLLQIDGVTEDK
LEKYGAEVIPILQKYSE----------------WTVPAEDNSP--RRSPSSCRGS---GR
NAPEE-----LDEETLI-------SSHYFANKTKNERKRK--RIPASQRSKRRKTSF-SG
SK--AKG-----------------------------------------------------
------------------------------------------------------------
------------------------------------------------------------
------------------------------------------------------------
------------------------------------------------------------
------------------------------------------------------------
------------------------------------------------------------
------------------------------------------------------------
```


>M3W592
--------------------------------MAAIPQNNLQEQL---------------

--ERHS-ARKLNNGLS--LAKPKS--------SGFT------------------------
---------FKKKISSAND---VSVTSV--------SVAKAPVLS--------DKSVNVT
ATFSFSEPSP-HITSQQ----TRVND-FKNAPAGQQTRRAVS------KPSLPDLLQV--
--PRGV-FCNT--R---NTSVVKK----SSNATFKKLEFSSSSDSFVTV-DDWDDM-DDF
D--ISGNSEAFVTP----CKSHV--VRVSTAQKSKKSKRNFF--------QA---QR---
SQAN-------------TIKADVTPAS--SASEQACVTKEQNNY---------SEW---L
SNDVICI-DDDPIPEELLGG----------DAQDGHPVKTHLGKER-------------E
SSE-KKISEA--------TELHPVEK------SPCVEL----NKEDYDADFIP---PSP-
EEVT-------SASSSAL---------------NCLSILKDLDASDTK-DGPS-TSED-L
AKPRETTMWQPDQ--ETSTDYEARQTSLQQQLIR--------------------------
----------------------VMDRICK-------------------------------
--LVDTIPDDELTALD----
---------------------CGQELRQHRDIR----------------------RKLLA
--D-TDSNTSGAR-VLG----SV----WRCGPE---------ALGGPR------------
-------------EDDSCPTGSCVFVKELH-----------FPHLPSNSNAAREC-----
------------------VLATTPG---------------------------------KT
GFSA-PTKNPFERPLFSP-----------------HLQKSFVSSNWAETPRIEKRN----
----------ESSYFPGNVLTSTAVK----DQNKHTASL----------------NDL-E
REIQASCDVDNFDI---DDF---DD---------DDDWENIM-HNLA-------------
--ASKSSTAAYQPIK--EGRPVKPVSE-RI---SSAKTN------------------CLP
LASAAQIKN-SESIQN--YTDKSAQNLAS--RNLKH-----ERFQSL-----S-FPH---
------TKEMMKIFHKKFGLHNFRTNQLEAINAALLG--EDCFIL---MPT--GGGKSLC
--------YQLPA---------------------CV----SPGV-TIVISPLRSLIVDQV
QKLTS------LDI-----------------PATYLTGD---KTDSEA---TSIYLQLSK
KDP--IIKLLYVTPE-KVCAS---------------NRLISTLENLYERKLLARFVIDEA
HCV--SQWG------HDFRQDYKRMNMLRQKF--PS-VPVMALTATAN----------PR
VQKDILTQLKIL----QPQVFSM--SFNRHNLKYYVL---PK-------------KPKKV
AF--DCLEWIRKH-HPH----DSGIIYC-----LSRREC--------DTM---------A
DTL---QKDGLAALA-YHAGLSDSAR-DEVQHKW----INQDG-CQ----VICATIAFGM
GIDKPDVRFVIHASLPKS-VEGYYQESGRAG-RDGEISHCLLF-YTYHDVTRLKRLI---
--------LMEKDG--NRHTR--------ETHFNNLYSM--VHYCENITE----------
CRRI---QLLAYFGE--NGFNPDFCKKYPDV--SCDNC--CK----TKDCKTR--DVTDD
VKDIVRFVQEHSS-----AQGTQNKPPAGP-SGRFTMNML-VD-IFLGSKSAKIQS----
------GIFGKGSTYSRHNAERLFKKLILDKILDEDLYINAN-DQPI--AYMVPGNKAET
VLNGH----L-------------K---VDFMETENSSSVKKQK----------ALVAKMS
Q--RE--E
IVKKCLGELTEVCKSLGKVFGV-HYFNIFNTVTLKKLA--ESLSSDPEVLLQIDGVTEDK
LEKYGAEVIPILQKYSE----------------WTLPADDGSP--RVSPGSSRGT---RR
NAPVE-----SDEEMPV-------SSHYFANKTRNERKRK--RMPASQRSKRRKTGF-GG
SK--TKG---------GSTTCRRI--SAKSKSSNIFGPRSPLPGP-----------QAAP
---------------------GASRK----------------------------------

--LGIMAPP-
--KP--------------
---------INRPFLKPSYAFS-----------------
>M3YW37
-----------------------MDASGSTWIMAAVPQNNLQEQL---------------

--ERHS-ARKLSNKLS--PSKPKP--------SGFT------------------------
---------FKKKVTLANN---VSVTGV--------SVAKTPALN--------DKDVNVA
EAFSFSGPPP-LPASQQ----TSPSD-FRNAPAGQQTRRAVS------KPLLPDVLQV--
--PQGV-LCNT--Q---NTPIVKK----SSNATFKKLEFSSSSDSFVTI-NDWDDM-DDF
D--TSGNSKAFVTP----CRNHF--VRVSTAQKSKKSKRNVL--------KA---QL---
PKEN-------------TVKADLTLSS--SESKQACLTKKQNDD---------SEW---F
NNDVIYI-DEDPGPEDLIPG----------SAQESQPSKTHLGVER-------------D
SSE-KKNLEE--------IEFLSVKR------SPCIEL----DEDDYDTDFIP---PSPE
EEMI-------SASSSSL---------------KCSSMLKDLDTSDTK-GGPS-TSED-F
PTPEGTTTQPPDQ--ETSTDCDARQTSVQQQLIH--------------------------
----------------------VMDQICK-------------------------------
--LVDTIPDHELKALD----
---------------------CGNELHQQRDIR----------------------RKLLA
--E-ADFNTSDAS-ILA----SW----WRCRPA---------SHGNPVEDMASV------
------SAPCAFPKGDSCPTGN--SVKELN-----------FPHLPSNPSSATEC-----
------------------LVAATPG---------------------------------KT
GFTA-TTKNPSERPLFNP-----------------HLQKSFVSSNWADTPRVEKRN----
----------ESSYFPGRVLTSTAVK----DQNKRAAPV----------------IDL-E
REFQAACDIDSFDI---DGF-DDDD---------DDDWENIM-HSLA-------------
--ASKSSTAACQPIK--EGWPVKPVPE-RS---SSTKTN------------------CLP
MASAAQNKN-SESIQK--NTDKSAQNLAS--RNLKH-----ERFRSL-----S-FPH---
------TKEMMKIFHKKFGLHNFRTNQLEAINAALLG--EDCFIL---MPT--GGGKSLC
--------YQLPA---------------------CV----SPGV-TIVISPLRSLIVDQV
QKLTS------LDI-----------------PATYLTGD---KTDSEA---TSIYLQLSK
KDP--IIKLLYVTPE-KVCAS---------------NRLISTLENLYERKLLARFVIDEA
HCV--SQWG------HDFRQDYKRMNMLRQKF--PS-VPVMALTATAN----------PR
VQKDILTQLKIL----QPQVFSM--SFNRHNLKYYVL---PK-------------KPKKV
AF--DCLEWIRKH-HPH----DSGIIYC-----LSRREC--------DTM---------A
DTL---QKDGLAALA-YHAGLSDSAR-DEVQHKW----VNQDG-CQ----VICATIAFGM
GIDKPDVRFVIHASLPKS-VEGYYQESGRAG-RDGETSHCLLF-YTYHDVTRLKRLI---
--------LMEKDG--NHHTR--------ETHFNNLYSM--VHYCENITE----------
CRRI---QLLAYFGE--NGFNPDFCKKYPDV--SCDNC--CK----TKDYKTR--DVTDD
VKNIVRFVQEHSS-----SQGTRNKNHMGP-SGRFTMNML-VD-IFLGSKSAKIQS----
------GIFGKGSTYSRHNAERLFKKLILDKILDEDLYINAN-DQPI--AYVMPGNKAQT
VLNGH----L------------K---VGYF-----------------------------

```
------------------------------------------------------------
---KCL----------------------FYLIILQ-------------------------
------------------------------------------------------------
------------------------------------------------------------
------------------------------------------------------------
------------------------------------------------------------
------------------------------------------------------------
------------------------------------------------------------
------------------------------------------------------------
------------------------------------------------------------
------------------------------------------------------------
------------------------------------------------------------
------------------------------------------------------------
------------------------------------------------------------
---------------------------------------
>U6D7S1
------------------------------------------------------------
------------------------------------------------------------
------------------------------------------------------------
------------------------------------------------------------
------------------------------------------------------------
------------------------------------------------------------
------------------------------------------------------------
---------------------DLTLSS--SESKQACLTKKQNDD---------SEW---F
GNDVICI-DDDPGPEDLIPG----------SAQESQPSKTHLGVER-------------D
SSE-KKNLEE--------TEFLSVKR------SPCIEF----DEDEYDTDFIP---PSPE
EKMI-------SASSSSL---------------KCSSMLKDLDTSDTR-GGPS-TSED-L
PTPEGATTQPPDQ--ETSTDCDARRTSVQQQLIH--------------------------
----------------------VMDQICK-------------------------------
------------------------------------------LVDTIPDHELKALD----
---------------------CGNELHQQRDIR----------------------RKLLA
--E-ADFSTSDAS-ILA----SW----WRCRPA---------SHGNPVEDMASV------
------SAPCTFPKGDSCPTGN--SVKELN-----------FPHLPSNPSSTTEC-----
------------------LVAATPG---------------------------------KT
GFTA-TTKNPSERPLFNP-----------------HLQKSFVSSNWADTPRVEKRN----
----------ESSYFPGSVLTSTAVK----DQNKRSAPI----------------IDL-E
REFQAACDIDSFDI---DGF-DDDD---------DDDWENIM-HSLA-------------
--ASKSSTAACQPIK--EGRPIKPVPE-RS---SSTKTN------------------CLP
MASAAQNKN-SESIQK--YTDKSAQNLAS--RNLKH-----ERFRSL-----S-FPH---
------TKEMMKIFHKKFGLHNFRTNQLEAINAALLG--EDCFIL---MPT--GGGKSLC
--------YQLPA---------------------CV----SPGV-TIVISPLRSLIVDQV
QKLTS------LDI-----------------PATYLTGD---KTDSEA---TSIYLQLSK
KDP--IIKLLYVTPE-KVCAS---------------NRLISTLENLYERKLLARFVIDEA
HCV--SQWG------HDFRQDYKRMNMLRQKF--PS-VPVMALTATAN----------PR
VQKDILTQLKIL----QPQVFSM--SFNRHNLKYYVL---PK-------------KPKKV
AF--DCLEWIRKH-HPH----DSGIIYC-----LSRREC--------DTM---------A
DTL---QKDGLAALA-YHAGLSDSAR-DEVQHKW----VNQDG-CQ----VICATIAFGM
```

GIDKPDVRFVIHASLPKS-VEGYYQESGRAG-RDGETSHCLLF-YTYHDVTRLKRLI---
--------LMEKDG--NHHTR--------ETHFNNLYSM--VHYCENITE----------
CRRI---QLLAYFGE--NGFNPDFCKKYPDV--SCDNC--CK----TKDYKTR--DVTDD
VKNIVRFVQEHSS-----SQGTRNKNHVGP-SGRFTMNML-VD-IFLGSKSAKIQS----
------GIFGKGSTYSRHNAERLFKKLILDKILDEDLYINAN-DQPI--AYVMPGNKAQT
VLNGH----L-------------K---VDFMETENSSSVKKQK----------ALEAKIS
Q--RE--E
VVKKCLGELTEVCKSLGKVFGV-HYFNIFNTVTLKKLA--ESLSSDPEVLLQIDGVTEDK
LEKYGAEVISVLQKYSG----------------WTLPADDGSP--LRSPGSSRGT---RK
NGPEE-----FDEETPI-------SSHYFTDKTKNERKRK--RMSASQRPKRRKTGF-GG
SK--TKG---------GSTACRRT--SSKSRSSNVFGPHSSVPGS-----------QAAS
---------------------GAPRR----------------------------------

--LGIMAPP-
--KP--------------
---------INRPFLKPSYAFS-----------------
>A0A1U7QP15
--------------------------------MAAVPLNNLQEQL---------------

--QRHS-ARKLNNKSS--LAKPKS--------SGFT------------------------
---------FKKKTSEGD----VSITSV--------SVVKTPALS--------DKDVIVS
EALPSTESPPLHTPKQQ----PGTDGFFKTSLGGQQTK--GA------KPSQLDLLQI--
--PQEV-LCTT--P---KTPAVKK----PHVAVFKKLEFSSSPDS---V-NDWADL-DDF
D--VS-ASNAFVSL----NRNPA--VRVSTAQKPKKPNRNFF--------KP---PP---
HKAN-------------VVKAALTLSS--PESSQVDLTKEQKDE---------SEC---L
SSDVICI-DSDPGSEELVKK----------DTQESQSLETQLGVEK-------------D
NSEKKNHVDE--------AKLQSTEK------ALYFQHD--DDDDDDDIDFVP---PSPE
EEII-------SPASSSS---------------KCCSMLKDLDDSDKEKDILS-TSKDFL
SKPEEMPIQKPDP--RTNTDCDDKQMSVQQQLMR--------------------------
----------------------VMEHICE-------------------------------
--LVDTVPIDELKALN----
---------------------CGNELLQQRNIR----------------------RELLA
--K-AGFNGKDVS-LLS----SL----WRCRPD---------SHGNTV------------
-------------QGDSCPVGH--PSKELN-----------SPSFPSHSFSTEEC-----
------------------LPTTTPG---------------------------------KT
GFSA-TTKNLLERPLLNS-----------------HLQKSFVSSNWAETLRTEKRN----
----------ESTSFPGNVLTSTTVK----DQNKHAALV----------------NNL-E
REVQASYDIDNFNI---DDF-DDDD---------DDDWENIM-HSFS-------------
--ASKSSTAAYPPIK--EGGPIKSLSE-RI---SSAKTK------------------SLP
VVSTAQNKMLSESIQN--YTDKLEQNLSS--KNLKH-----EHFQSL-----N-FPH---
------TKEMMKIFHKKFGLHNFRTNQLEAINAALLG--EDCFIL---MPT--GGGKSLC
--------YQLPA---------------------CV----SPGV-TIVISPLRSLIVDQV

```
QKLTS------FDI-----------------PATYLTGD---KTDSEA---ANIYLQLSK
KDP--IIKLLYVTPE-KVCAS---------------NRLISTLENLYERKLLARFVIDEA
HCV--SQWG------HDFRQDYKRMNMLRQKF--PS-VPVMALTATAN----------PR
VQKDILTQLKIL----RPQVFSM--SFNRHNLKYYVL---PK-------------KPKKV
AF--DCLEWIRKH-HPY----DSGIIYC-----LSRREC--------DTM---------A
DTL---QRDGLAALA-YHAGLSDSAR-DEVQNKW----INQDG-CQ----VICATIAFGM
GIDKPDVRFVIHASLPKS-VEGYYQESGRAG-RDGEISHCLLF-YTYHDVTRLKRLI---
--------MMEKDG--NYHTK--------ETHFNNLYSM--VHYCENITE----------
CRRI---QLLAYFGE--KGFNPDFCKKYPDV--SCDNC--CK----TKDYKTK--DVTDD
VKSIIRFVKEHSS-----SPGTRNI---GP-SGRFTLNML-VD-IFLGSKSAKIQS----
------GIFGKGSSYSRHNAERLFKKLILDKILDEDLYINAN-DQPI--AYMMLGTKAQN
VLNGH----L-------------K---VDFMETENSSSIKKQK----------ALVAKVS
Q------------------------------------------------------RE--E
VVKKCLGELTEVCKLLGKVFGV-HYFNIFNTATLKKLA--ESLSSDPEVLLQIDGVTEDK
LEKYGAEIIPVLQKYSE----------------WTLPAENSSP--GITLTGGRGT---GR
STPEE-----PDEEEPV-------SSHYFTNQTRNERKRK--KMPASQRLKRRRTSY-GG
FK--AKG---------VSTASRKT--SSKSKFSGVTGSSSASCAS-----------QAAS
---------------------VASRK----------------------------------
------------------------------------------------------------
------------------------------------------------------------
------------------------------------------------------------
------------------------------------------------------------
------------------------------------------------------------
------------------------------------------------------------
----------------------------------------------------LGIMAPP-
--------------------------------------------KP--------------
---------VNRAFLRPSYAVS-----------------
>I3M5A7
--------------------------------MAAVPLNNLQEQL---------------
------------------------------------------------------------
--ERHS-ARKLNSKLS--LSKPKS--------SGFT------------------------
---------FKKKLSSDNDASVASVTSV--------SVAKTPVLS--------DKDVNVS
EAFSFNESLP-HNPNQQ----TTINDFFKNVPAGQQTKATSS------KSLLPNMLQI--
--PQEV-LCTT--Q---NTPAIKT----PCNAVFKKLEFSSSPDSFSVM-NDWDDM-DDF
D--T---SKTFSTP----PENHF--VRVSTAQKSKKAKRNFI--------KP---QP---
HKTN-------------TVKTDLTPSS--SENQQIDSAKDQKDD---------SEW---L
SSGVICI-DDDPVSETLIND----------NTQESYSLKTHLGDER-------------G
NGEKKKNVEE--------VELLSTEK------LPHIDF----DDDDYDIDFVP---PSPE
EEII-------SGASSSL---------------KSFSMLKDLDASDREKDVLS-ASKDVL
SKPEKMTTQEPNP--ETNEDFDARQINLKQQLIH--------------------------
----------------------VMDHICK-------------------------------
------------------------------------------LVDMIPVDDLKALN----
---------------------CGNELLQQRNIR----------------------RKLLA
--E-ADFNGNDVS-LFG----SV----WRHRPV---------SLDNPV------------
-------------ESDFCPGGN--SVKELN-----------FPHLPSNSLSTGKC-----
------------------LLTTTPE---------------------------------KT
ESSD-TLKNFFERPLFTS-----------------HLQKSFVSGNWAETPRIEKRN----
```

----------E-----RNVLTSTAIK----NQNKNIASK----------------NYL-E
GEIQASYDIDNFDI---DDF---DD---------DEDWENIM-HNLT-------------
--ASKSSTAAYHPIK--EGRPIKSVSE-KI---SSAKTN-------------------CLS
VSSTAQTKSFSESIQN--HTDNLAQNLAS--TNLRH-----ERFQGL-----S-FSH---
------TKEMMKIFHKKFGLHNFRTNQLEAINATLLG--EDCFIL---MPT--GGGKSLC
--------YQLPA---------------------CV----SPGV-TIVISPLRSLIIDQV
QKLTS------LDI------------------PATYLTGD---KTDSEA---TSIYLQLSK
KDP--IIKLLYVTPE-KVCAS---------------NRLISTLENLYERKLLARFVIDEA
HCV--SQWG------HDFRQDYKRMNMLRQKF--PS-VPVMALTATAN----------PR
VQKDILTQLKIV----KPQVFSM--SFNRHNLKYYVL---PK-------------KPKKV
AF--DCLEWIRKH-HPY----DSGIIYC-----LSRREC--------DTM---------A
DTL---QKNGLAALA-YHAGLSDSAR-DEVQHKW----INQDG-CQ----VICATIAFGM
GIDKPDVRFVIHASLPKS-VEGYYQESGRAG-RDGEISHCLLF-YTYHDVTRLKRLI---
--------MMEKDG--NHHTR--------ETHFNNLYSM--VHYCENITE----------
CRRI---QLLAYFGE--NGFNPDFCKKYPDV--SCDNC--CK----TKDYKTR--DVTDD
VKNIVSFVQEYSS-----SQGTRNIKHVGP-SGRFTMNML-VD-IFLGSKSAKIQS----
------GIFGKGSAYSRHNAERLFKKLILEKILDEDLYINAN-DQPI--AYIMLGTKAQS
VLNGH----L-------------K---VDFIEMENFSNLKKQK----------ALVAKVS
E---RE--E
MVKKCLGELTEVCKSLGKVFGV-HYFNIFNTATLKKLA--ESLSSDPEVLLQIDGVTEDK
LEKYGAEVIPILQKYSE----------------WTLPAKDSSP--GISLPGNRGP---RK
NDPEE-----LDEEEPV-------SSHYFTNKTRNERKRK--KMPISQKSKRRKSSY-GG
FK--PRG---------GTTTCRKI--SSKSKSSNIMGSSSTLHGS-----------QATS
---------------------GARRK----------------------------------

--LGIMAPP-
--KP--------------
---------INRPFLRPSYAFS-----------------
>A0A091DWF8
--------------------------------MAAVPLNNLQEQL---------------

--ERHS-ARKLNNKSG--LSKPKS--------CGFT------------------------
---------FKKKTTSDND---ASVISV--------SIAKTPVLS--------DKDVNVS
EAFSFTEPPP-HTPSQQ----PRINGFFKNAAAGQQGQGTGS------KLLLPDLLQI--
--PQEASLPPT--Q---NTSAVKK----PHESILKKLEFSSSSDSFHTM-NNWDDM-DDF
D--TSETSKIFATP----PKNHL--VRVSTAQKSKKAKRNFF--------KANFLQP---
HKTG--------------TVKADLTL----SESQPVDLTKEEKDD---------SEW---L
KSGVICI-DDDSIPQVHTNE----------DTQESQSLKIHLGDGR-------------D
NNE-KHNEED--------VELHSSEK------ATCIEL----NDDDY-IDFVP---PSPE
EESI-------SASSSSL---------------KCFSTLKDLDASDREEDVLS-TSKDLL
SKPEKMTTQEPNP--EISTDCDARQISLQQQLLH--------------------------
----------------------VMEHICK-------------------------------

--LVDTIPVDELKALD----
---------------------CGEELLQQRNRR----------------------RKLLA
--E-AGFTRNDAS-LLG----SV----WKYRPD---------SLGSFV------------
-------------ESDSCPT-S--FVKELN-----------VPPLPSHSLSTREH-----
------------------LLTTSPG---------------------------------KT
GFSA-TTSTL-ERPLFNS-----------------HLQKSFVNSNWAETPRIEKRN----
----------ESSYFLGNVLTSTAVK----DQNKHAASV----------------NDL-E
GESQASYDVDNFDI---DDF--DDD---------DDDWENIM-HDLA-------------
--ASKPSTAAYQPIK--KGGPLKSVSE-KI---SAGKAH------------------SLP
GASVA---------QH--HTDKFTQNLAS--RNTKL-----ERFRSH-----S-FPH---
------TKEMMKIFHKKFGLHNFRTNQLEAINAALLG--EDCFIL---MPT---------

--------------------DYKRMSILRQKF--PS-VPVMALTATAN----------PR
VQTDILTQLKIL----RPQVFSM--SFNRHNLKYYVL---PK-------------KPKKV
AF--DCLDWIRKH-HPY----DSGIIYC-----LSRREC--------DTM---------A
DTL---HRDGLAALA-YHAGLSDAAR-DEVQHKW----INQDG-CQ----VICATIAFGM
GIDKPDVRFVIHASLPKS-VEGYYQESGRAG-RDGEMSHCLLF-YTYHDVTRLKRLI---
--------MR--

--SKSAKVQS----
------GIFGKGSAYSRHNAERLFKKLILDKILDEDLYINAN-DQAI--AYVMLGIKAQA
ILNGH----L-------------K---VDFMETENSSSMKKQK----------ALEAKVS
Q--RE--E
MVKKCLGELTEVCKSLGKVFGV-HYFNIFNTATLKKLA--AEEASPP-------------
---VGTRGP---RR
NAPEE-----SEEEMPV-------SSHYFANRTRSERKRK---MPASQRSKRRKTSF-GG
FK--AKG---------ASTTCRRM--SSKSKFSSTVGSSSALPSS-----------QATS
---------------------GASKK----------------------------------

--LGIMAPP-
--KP--------------
---------INRQFLRPSYAFS-----------------
>F6ZL78
--------------------------------MAAVPLNNLQEQL---------------

--ERHS-ARKLNNKLS--LSKPKS--------SGFT------------------------
---------FKKKISSAND---VSVTSV--------SVAKTPVLS--------DKDVNVT
EARSFTEPLP-PPPNQQ----TSISD-FKKAPGGQQTKRIGS------KPLLPDLLQF--
--PQEV-LCTT--Q---KTPVVKK----SRDATFKKLEFSSSSDSFITI-NDWDDM-DDF
D--TSGSSKAFVTP----SRNHS--VRVSTAQKCKKSKRTFL--------KA---QL---

```
PKAN-------------SVKADLTPSS--SEIEQVDLTKKQ-DD---------SEW---L
SSDVICI-DDGPNSKELINE----------DMQESHS----LGEER-------------D
SGEKKKNLEE--------KELHSIEK------SPCVEF----DEDDYDMDFVP---PSPE
EGMI-------SGSSSSL---------------KCLSVLKDLDTPDRK-DGLS-TSED-L
SKAEKMTVQQPHQ--ETSTDCDVRQISLQQQLIH--------------------------
----------------------VMDHICK-------------------------------
------------------------------------------LVDTIPDAELKALN----
---------------------CGNELLQQRNIR----------------------RKLLA
--E-VDFNTSDAG-VLG----ST----WRGRPD---------SLGSPV------------
------------EDAGSCPTEN--SV-EFN-----------FPHLPSNSISAGEC-----
------------------LLTTTPG---------------------------------KT
GFSA-TTKNLFERPLFNS-----------------HLQKSFVSSNWAETPRIEKRN----
----------ESSYFPGNVLTSTAVK----DQNKHTASV----------------NDL-E
REIQASREIDNFDI---DDF---DD---------DDDWENIM-HNLA-------------
--ASKSSTAAYQPVK--EGGPVKSVSE-RI---SSAKTN------------------CLP
VASTAQNKNFSESIQN--CTVKSAQNLAS--GNLKH-----ERFQSL-----S-FPH---
------TKEMMKIFHKKFGLHHFRTNQLEAINAALLG--EDCFIL---MPT--GGGKSLC
--------YQLPA---------------------CV----SPGV-TIVISPLRSLIVDQV
QKLTS------LDI-----------------PATYLTGD---KTDSEA---TSIYLQLSK
KDP--IIKLLYVTPE-KVCAS---------------NRLITTLENLYERKLLARFVIDEA
HCV--SQWG------HDFRQDYKRMNMLRQKF--PS-VPVMALTATAN----------PR
VQKDILTQLKIL----SPQVFSM--SFNRHNLKYYVL---PK-------------KPKKV
AF--DCLEWIRKH-HPY----DSGIIYC-----LSRREC--------DTM---------A
DTL---QKDGLAALA-YHAGLSDSAR-DAVQHKW----INQDG-CQ----VICATIAFGM
GIDKPDVRFVIHASLPKS-VEGYYQESGRAG-RDGEISHCLLF-YTYHDVTRLKRLI---
--------LMEKDG--NHHTK--------ETHFNNLYSM--VHYCENIAE----------
CRRI---QLLAYFGE--HGFNPDFCKKYPDV--SCDNC--CK----TKDYKTR--DVTED
VKNIVRFVQEHSS-----SQGMRNIKHLGP-SGRFTMNML-VD-IFLGNKSAKIQS----
------GIFAKGSAYSRHNAERLFKKLILDKILDEDLYINAN-DQPI--AYVMPGNKAQT
VLNGH----L-------------K---VDFMETENYSSVKKQK----------ALVAKVS
Q------------------------------------------------------RE--E
MVKKCLGELTEVCKALGKVFGV-HYFNIFNTVTLKKLA--ESLSSDPEILLQIDGVTEDK
LEKYGAEVIPILQKYSE----------------WTLPAEDNSP--RISPSSSRGT---GR
NAPEE-----FDEETPI-------SSHYFANKTKNERKRK--RISASQRSKRRKTGF-SG
SK--TKG---------GSTASRKL--SSKTKSSSITGPS--LHGS-----------QAAS
---------------------GATRK----------------------------------
------------------------------------------------------------
------------------------------------------------------------
------------------------------------------------------------
------------------------------------------------------------
------------------------------------------------------------
------------------------------------------------------------
----------------------------------------------------LGIMAPP-
--------------------------------------------KP--------------
---------VNRPFLKPSYALS-----------------
>L5L4C6
--------------------------------MAAVPQNNLQEQL---------------
```

```
------------------------------------------------------------
--ERHS-ARKLNNKLS--FSKPKS--------SGFT------------------------
---------FKKKISAAND---VSVTSV--------SVAKTPVLS--------DKDINVT
EAFFFSEPLA-HTTSQQ----TEIDD-FQNAPARQQTKRAGS------KPLLPDLVQ---
----EV-LHTT--Q---STPIIKE----SRDPAFKKLEFSSSSDSFIAI-NNWDDM-DDF
D--TSGNSKAFVTP----LRNHF--VRVSTAQKSKKSKRNFF--------KA---HL---
HKAN-------------TVKVDLTSSS--SESNQVYSTKEQKAD---------SKW---L
NSGVICI-DDSPNSQELINE----------DSQDSHSLKTHLREEG-------------D
NSEKKKNFEE--------TELYSIEK------SPCIEL----DEDDYDIDFVP---PSPE
EEMT-------SASSSSL---------------KCFSMLKDLDTCDQKKESFG-TSED-L
TKPEKMPTQQSNE--ESSTDCDAKQINLQQQLIR--------------------------
----------------------VMDNICN-------------------------------
------------------------------------------LVDTIPDNELKSLD----
---------------------CGNNLLQQRNIR----------------------RKLLA
--E-ADFNPGDAG-VLG----VV----WRCRPD---------ALGSPMGDVASV------
------SSPYAFPKGDFCPRGN--S-DEFN-----------FPLVPSNSISTGEC-----
------------------LVTTTPG---------------------------------RA
GFSA-TRKNLTEGPSFIS-----------------HVQQCFVSSDQADTPRIEKRN----
----------ESSHFPRNVLTSTAMT----DQNEHIASV-----------------DL-E
REIQASYDTDNFDI---DGF---DD---------DDEWEDIM-HNLA-------------
--ASKSSTAVYQPIK--EGRPVKSVSE-RV---SPSKTN------------------CLP
VASTPQNKNFSESIQN--YTDESAQNLPS--RHLKH-----ERFQSL-----S-FPH---
------TREMMKIFHKKFGLHNFRTNQLEAINAALLG--EDCFIL---MPT--GGGKSLC
--------YQLPA---------------------CV----SPGV-TIVISPLRSLIVDQV
QKLTS------LDI-----------------PATYLTGD---KTDSEA---TSIYLQLSK
KDP--IIKLLYVTPE-KVCAS---------------NRLISTLENLYERNLLARFVIDEA
HCV--SQWG------HDFRQDYKRMNMLRQKF--PS-VPVMALTATAN----------LR
VQKDILTQLKIL----RPQV----------------------------------------
------------------------------------REC--------DTM---------A
DTL---QKNGLAALA-YHAGLSDSAR-DEVQHKW----INQDG-CQ----VICATVAFGM
GIDKPDVRFVIHASLPKS-VEGYYQESGRAG-RDGEVSHCLLF-YTYYDVTRLKRLI---
--------LMEKDG--NHHTK--------ETHFNNLYSM--VHYCENITE----------
CRRI---QLLAYFGE--IGFNPDFCKKYPDV--SCDNC--CK----TKDYKTR--DVTDV
TKNIIRFVQEHSS-----SQGTRNIKHVGP-CGRFTMNML-VD-IFLGSKSAKIQS----
------GLFGKGSTYSRHNAERLFKKLILDKILDEDLYINAN-DQPI--AYVMTGSKAQT
VLNGH----L-------------K---VDFMETENSSSIKKQK----------ALVTKVS
Q------------------------------------------------------RE--E
VVKKCLGELTEVCKALGKVFGV-HYFNIFNTATLKKLA--ESLSSDPEVLLQIDGVTEDK
LEKYGAEVIPVLQKYSE----------------WTLPGK---------------------
------------------------------------------------------------
------------------------------------------------------------
------------------------------------------------------------
------------------------------------------------------------
------------------------------------------------------------
------------------------------------------------------------
------------------------------------------------------------
------------------------------------------------------------
```

```
------------------------------------------------------------
---------------------------------------------------------VW-
--------------------------------------------QD--------------
---------RDHPISLENVALK-----------------
>A0A1U7TXA0
--------------------------------MAAVPQNNLQEQL---------------
------------------------------------------------------------
--ERHS-ARRLNNKLS--LSKPKS--------SGFT------------------------
---------FKKKTSSESD---VSLTSV--------SVAKMPVLS--------NKDVNVT
EDFSFSEPLS-HIKNQS----TRISDVFKNAPEGQQTQRLAS------KP----SLQI--
--PKEV-LCTT--Q---NTPVIKK----SRDAAFKTLEFSSSSDSFNAI-DDWDDM-DDF
D--TTGTSKAFVTP----SRNHF--VRVSTAQKSKKAKRNFL--------KA---QL---
YKTN-------------TVNIDLTSS---SESKQVDLTKEQKDD---------SEW---L
SSDVICI-DDGPVFKVPTSE----------DTQKSHSLETHLGDER-------------D
NSEKKNNLEE--------AELHSTEK------VPCIEF----DDDDYNIDFVP---PSPE
EEIN-------STTSSSL---------------KCFSMLKDLDTSEEKKDVLS-TSEDLL
SAPENVPTQKPHP--EASTDCDAGQKSLQQQLIQ--------------------------
----------------------VMDRICK-------------------------------
------------------------------------------LVDTIPADKLIALD----
---------------------CGNELLQQRNIR----------------------RKLLA
--E-ADCNTNDAS-LLG----WI----WRRRPD---------SLCSPV------------
-------------EGDSYPREI--SMKELN-----------SSHLSLDSVSPGEC-----
------------------LLTTTPG---------------------------------KT
GFSA--RKDLFERPLFNS-----------------HLQESFVSSNWAETPRLQKRN----
----------ESSYFPGNVLTSTAVK----DQNKHTASI----------------SDL-E
RETQASYDIDNFDI---DDF---DD---------DDDWENIM-HNLA-------------
--ASKSSTAAYQPIK--EGPPIKSVTE-RI---ASAKTN------------------CLP
VASTAQNINSSESIQS--YTDKSAQNLAS--RNLKH-----ERFQSL-----S-FPH---
------TKEMMKIFHKKFGLHNFRTNQLEAINAALLG--EDCFIL---MPT--GGGKSLC
--------YQLPA---------------------CI----SPGV-TVVISPLRSLIVDQV
QKLTS------LDI-----------------PATYLTGD---KTDSEA---TTIYLQLSK
KDP--IIKLLYVTPE-KVCAS---------------NRLISTLENLYERKLLARFVIDEA
HCV--SQWG------HDFRQDYKRMNMLRQKF--PC-VPVMALTATAN----------PR
VQKDILTQLKIL----SPQVFSM--SFNRHNLKYYVL---PK------------KPKKV
AF--DCLEWIRKH-HPY----DSGIIYC-----LSRREC--------DTM---------A
DTL---QKGSLAALA-YHAGLTDSAR-DEVQHKW----INQDG-CQ----VICATIAFGM
GIDKPDVRFVIHASLPKS-VEGYYQESGRAG-RDGELSHCLLF-YTYHDATRLKRLI---
--------MMEKDG--NHHTR--------ETHLNNLYSM--VHYCENITE----------
CRRI---QLLAYFGE--HGFNPDFCKKHPDA--SCDNC--CK----TKDYKTR--DVTDD
VKNIVRFVQEHSL-----SQGTRNIKHVGP-SGRFTLNML-VD-IFLGSKSAKIQS----
------GIFGKGSAYSRHNAERLFKKLILDKILDEDLYINAN-DQAI--AYVMLGNKAQS
VLNGH----L-------------K---VDFMETENSSSIKKQK----------ALVAKVS
Q------------------------------------------------------RE--E
MVKKCLGELTEVCKSLGKVFGV-HYFNIFNTVTLKKLA--ESLSSDPEVLLQIDGVTEDK
LEKYGAEVISVLQKYSA----------------RTLPAEDSSP--GTSLASNRGP---GR
NAPEE-----LNEDIPV-------SSHYFANKARNERKRR--KMPASQKAKRRKTGC-GG
SK--AKG---------GSTTCRKI--ASKTKSSSVIGTSSASHGS-----------QATL
```

```
---------------------GASRK----------------------------------
------------------------------------------------------------
------------------------------------------------------------
------------------------------------------------------------
------------------------------------------------------------
------------------------------------------------------------
------------------------------------------------------------
----------------------------------------------------LGIMAPP-
--------------------------------------------KP--------------
---------INRPFLKPSYAFS-----------------
>A0A1U7TMS8
--------------------------------MAAVPQNNLQEQL---------------
------------------------------------------------------------
--ERHS-ARRLNNKLS--LSKPKS--------SGFT------------------------
---------FKKKTSSESD---VSLTSV--------SVAKMPVLS--------NKDVNVT
EDFSFSEPLS-HIKNQS----TRISDVFKNAPEGQQTQRLAS------KP----SLQI--
--PKEV-LCTT--Q---NTPVIKK----SRDAAFKTLEFSSSSDSFNAI-DDWDDM-DDF
D--TTGTSKAFVTP----SRNHF--VRVSTAQKSKKAKRNFL--------KA---QL---
YKTN-------------TVNIDLTSS---SESKQVDLTKEQKDD---------SEW---L
SSDVICI-DDGPVFKVPTSE----------DTQKSHSLETHLGDER-------------D
NSEKKNNLEE--------AELHSTEK------VPCIEF----DDDDYNIDFVP---PSPE
EEIN-------STTSSSL---------------KCFSMLKDLDTSEEKKDVLS-TSEDLL
SAPENVPTQKPHP--EASTDCDAGQKSLQQQLIQ--------------------------
----------------------VMDRICK-------------------------------
------------------------------------------LVDTIPADKLIALD----
---------------------CGNELLQQRNIR----------------------RKLLA
--E-ADCNTNDAS-LLG----WI----WRRRPD---------SLCSPV------------
-------------EGDSYPREI--SMKELN-----------SSHLSLDSVSPGEC-----
------------------LLTTTPG---------------------------------KT
GFSA--RKDLFERPLFNS-----------------HLQESFVSSNWAETPRLQKRN----
----------ESSYFPGNVLTSTAVK----DQNKHTASI----------------SDL-E
RETQASYDIDNFDI---DDF---DD---------DDDWENIM-HNLA-------------
--ASKSSTAAYQPIK--EGPPIKSVTE-RI---ASAKTN------------------CLP
VASTAQNINSSESIQS--YTDKSAQNLAS--RNLKH-----ERFQSL-----S-FPH---
------TKEMMKIFHKKFGLHNFRTNQLEAINAALLG--EDCFIL---MPT--GGGKSLC
--------YQLPA---------------------CI----SPGV-TVVISPLRSLIVDQV
QKLTS------LDI-----------------PATYLTGD---KTDSEA---TTIYLQLSK
KDP--IIKLLYVTPE-KVCAS---------------NRLISTLENLYERKLLARFVIDEA
HCV--SQWG------HDFRQDYKRMNMLRQKF--PC-VPVMALTATAN----------PR
VQKDILTQLKIL----SPQVFSM--SFNRHNLKYYVL---PK-------------KPKKV
AF--DCLEWIRKH-HPY----DSGIIYC-----LSRREC--------DTM---------A
DTL---QKGSLAALA-YHAGLTDSAR-DEVQHKW----INQDG-CQ----VICATIAFGM
GIDKPDVRFVIHASLPKS-VEGYYQESGRAG-RDGELSHCLLF-YTYHDATRLKRLI---
--------MMEKDG--NHHTR--------ETHLNNLYSM--VHYCENITE----------
CRRI---QLLAYFGE--HGFNPDFCKKHPDA--SCDNC--CK----TKDYKTR--DVTDD
VKNIVRFVQEHSL-----SQGTRNIKHVGP-SGRFTLNML-VD-IFL-------------
------------------------------------------------------------
```


--ESLSSDPEVLLQIDGVTEDK
LEKYGAEVISVLQKYSA----------------RTLPAEDSSP--GTSLASNRGP---GR
NAPEE-----LNEDIPV-------SSHYFANKARNERKRR--KMPASQKAKRRKTGC-GG
SK--AKG---------GSTTCRKI--ASKTKSSSVIGTSSASHGS-----------QATL
---------------------GASRK----------------------------------

--LGIMAPP-
--KP--------------
---------INRPFLKPSYAFS-----------------
>F7GNB7

--MHNLA-------------
--ASKSSTAAYQPIK--EGRPIKSVSEQL----SSAKTN------------------CLP
VASTAQNKNFSESMQN--YTDKSTQNLAS--RNLKH-----ERFQGL-----S-FPH---
------TKEMMKIFHKKFGLHNFRTNQLEAINAALLG--EDCFIL---MPT--GGGKSLC
--------YQLPA---------------------CV----SPGV-TIVISPLRSLIVDQV
QKLTS------LDI-----------------PATYLTGD---KTDSEA---TNIYLQLSK
KDP--IIKLLYVTPE-KICAS---------------NRLISTLENLYERRLLARFVIDEA
HCV--SQWG------HDFRQDYKRMNMLRQKF--PS-VPVMALTATAN----------PR
VQKDILTQLKIL----RPQVFSM--SFNRHNLKYYVL---PK-------------KPKKV
AF--DCLEWIRKH-YPY----DSGIIYC-----LSRREC--------DTM---------A

DTL---QRDGLSALA-YHAGLSDSAR-DEIQQKW----INQDG-CQ--------------
-------------------------------------CDSFIY------LIQIRCIM---

-------SLISF------AFISGY---------LCNNC----------------------
--IWNGD-----------

>A0A1D5R519

--MHNLA-------------
--ASKSSTAAYQPIK--EGRPIKSVSERL----SSAKTN------------------CLP
VASTAQNINFSESIQN--YTDKSAQNLAS--RNLKH-----ERFQSL-----S-FPH---
------TKEMMKIFHKKFGLHNFRTNQLEAINAALLG--EDCFIL---MPT--GGGKSLC

--------YQLPA---------------------CV----SPGV-TIVISPLRSLIVDQV
QKLTS------LDI-----------------PATYLTGD---KTDSEA---TNIYLQLSK
KDP--IIKLLYVTPE-KICAS---------------NRLISTLENLYERKLLARFVIDEA
HCV--SQWG------HDFRQDYKRMNMLRQKF--PS-VPVMALTATAN----------PR
VQKDILTQLKIL----RPQVFSM--SFNRHNLKYYVL---PK-------------KPKKV
AF--DCLEWIRKH-YPY----DSGIIYC-----LSRREC--------DTM---------A
DTL---QRDGLAALA-YHAGLSDSAR-DEVQQKW----INQDG-CQ----------SFAL

SPRL-----------------------------QCSGG----------------------
---LS-----------------

-----------------------TYFY---------------------------------
---------------------------------LHLPGSNDSP-----------------
------------------------ASAS--------------------------------

>Q4R3T8


```
------------------------------------------------------------
------------------------------------------------------------
------------------------------------------MHNLA-------------
--ASKSSTAAYQPIK--EGRPIKSVSERL----SSAKTN------------------CLP
VASTAQNINFSESIQN--YTDKSAQNLAS--RNLKH-----ERFQSL-----S-FPH---
------TKEMMKIFHKKFGLHNFRTNQLEAINAALLG--EDCFIL---MPT--GGGKSLC
--------YQLPA---------------------CV----SPGV-TIVISPLRSLIVDQV
QKLTS------LDI-----------------PATYLTGD---KTDSEA---TNIYLQLSK
KDP--IIKLLYVTPE-KICAS---------------NRLISTLENLYERKLLARFVIDEA
HCV--SQWG------HDFRQDYKRMNMLRQKF--PS-VPVMALTATAN----------PR
VQKDILTQLKIL----RPQVFSM--SFNRHNLKYYVL---PK-------------KPKKV
AF--DCLEWIRKH-YPY----DSGIIYC-----LSRREC--------DTM---------A
DTL---QRDGLAALA-YHAGLSDSAR-DEVQQKW----INQDG-CQ--------------
-----RQSFALSPRLQCS----------------GGLSTYFYL-----------------
------------------------------------------------------------
-------HLPGY---------------------LCDNC----------------------
--------------------------------------------IWNGD-----------
------------------------------------------------------------
------------------------------------------------------------
------------------------------------------------------------
------------------------------------------------------------
------------------------------------------------------------
------------------------------------------------------------
------------------------------------------------------------
------------------------------------------------------------
------------------------------------------------------------
------------------------------------------------------------
------------------------------------------------------------
------------------------------------------------------------
------------------------------------------------------------
------------------------------------------------------------
------------------------------------------------------------
------------------------------------------------------------
---------------------------------------
&gt;F7ES97
--------------------------------MAAVPQNNLQEQL---------------
------------------------------------------------------------
--ERHS-ARKLNNKLS--LSKPKF--------SGFT------------------------
---------FKKKTSSDNN---VSLTNV--------SVAKTPVLR--------NKDVNVA
EHFSFSEPLP-PTTDQQ-----RGKDFSKNVPAGQQTQRVGS------KPLLPDFLQT--
--PKEV-LCTT--Q---NTPTLEK----SRDTAFKKLEFSSSPDSLSTI-NDWDDM-DDF
D--TSGTSKAFVTP----PQSHF--VRVSTAQKSKKAKRNFF--------KA---QL---
CATN-------------TVKADLPPS---SESKQIDLTVEQKDD---------SEW---F
SSDVICI-DDGPIAEVHVNE----------DAQESDSLKTHLQDER-------------D
NNEKKKNVEE--------AELHSTEK------VPCIEF----DDDDYDIDFVP---PSP-
EEII-------SASSSSS---------------KCFSVSKDLDTSDRREDVLG-TSKDLL
SKPEKMTMQEPNP--ETSTDCDARRISLQQQLIH--------------------------
```

----------------------VMDRICK-------------------------------
--LVDIIPADKLKLLD----
---------------------CGNELLQQRNIR----------------------RKLLT
--E-VDLNKSDAS-LLG----PV----WRCRPD---------SLDEPV------------
-------------EGDSCPTEN--SMKGLN-----------FLHLSSDSVSPGEC-----
------------------LLTTTPG---------------------------------KT
GFSA-TRENLFERPLFNT-----------------HLQKSFVSSNWAETPRLEKRN----
----------ESPYFPGNVLTSTAVK----DWNKHTASI----------------NDL-E
RESQPSCDIGNFDI---DDF---DD---------DDDWEDIM-HNLA-------------
--ASKSSTAAYQPIK--EGRPIKSVSE-QL---SSAKTN------------------CLP
VASTAQNKNFSESMQN--YTDKSTQNLAS--RNLKH-----ERFQGL-----S-FPH---
------TKEMMKIFHKKFGLHNFRTNQLEAINAALLG--EDCFIL---MPT--GGGKSLC
--------YQLPA---------------------CV----SPGV-TIVISPLRSLIVDQV
QKLTS------LDI-----------------PATYLTGD---KTDSEA---TNIYLQLSK
KDP--IIKLLYVTPE-KICAS---------------NRLISTLENLYERRLLARFVIDEA
HCV--SQWG------HDFRQDYKRMNMLRQKF--PS-VPVMALTATAN----------PR
VQKDILTQLKIL----RPQVFSM--SFNRHNLKYYVL---PK-------------KPKKV
AF--DCLEWIRKH-YPY----DSGIIYC-----LSRREC--------DTM---------A
DTL---QRDGLSALA-YHAGLSDSAR-DEIQQKW----INQDG-CQ----VICATIAFGM
GIDKPDVRFVIHASLPKS-MEGYYQESGRAG-RDGEISHCLLF-YTYHDVTRLKRLI---
--------MMEKDG--NHHTR--------ETHFNNLYSM--VHYCENITE----------
CRRI---QLLAYFGE--NGFNPDFCKKHADV--SCDNC--CK----TKDYKTR--DVTDD
VKSIIRFVQEHSS-----SQGARNIKHIGP-SGRFTMNML-VD-IFLGSKSAKIQS----
------GIFGKGSAYSRHNAERLFKKLILDKILDEDLYINAN-DQAI--AYMMLGNKAQG
VLNGN----L-------------K---VDFMETENSSSVKKQK----------ALVAKVS
Q--RE--E
MVKKCLGELTEVCKSLGKVFGV-HYFNIFNTVTLKKLA--ESLSSDPEVLLQIDGVTEDK
LEKYGAEVIAVLQKYSE----------------WTLPAEDSSP--GISLSGSRGP---GK
SATEK-----LDEEIPV-------SSHYFANNTGNERKRK--RMPASQRSKRRKTAS-GG
SK--AKG---------GSATRRKM--SSRTKSS-IAGSSSASHAS-----------QATS
---------------------GANRK----------------------------------

--LGIMAPP-
--KP--------------
---------INRPFLKPSYAFA-----------------
>F7HZ92

-----MTMQEPNP--ETSTDCDARRISLQQQLIH--------------------------
----------------------VMDRICK-------------------------------
--LVDIIPADKLKLLD----
---------------------CGNELLQQRNIR----------------------RKLLT
--E-VDLNKSDAS-LLG----PV----WRCRPD---------SLDEPV------------
-------------EGDSCPTEN--SMKGLN-----------FLHLSSDSVSPGEC-----
------------------LLTTTPG---------------------------------KT
GFSA-TRENLFERPLFNT-----------------HLQKSFVSSNWAETPRLEKRN----
----------ESPYFPGNVLTSTAVK----DWNKHTASI----------------NDL-E
RESQPSCDIGNFDI---DDF---DD---------DDDWEDIM-HNLA-------------
--ASKSSTAAYQPIK--EGRPIKSVSE-QL---SSAKTN------------------CLP
VASTAQNKNFSESMQN--YTDKSTQNLAS--RNLKH-----ERFQGL-----S-FPH---
------TKEMMKIFHKKFGLHNFRTNQLEAINAALLG--EDCFIL---MPT--GGGKSLC
--------YQLPA---------------------CV----SPGV-TIVISPLRSLIVDQV
QKLTS------LDI-----------------PATYLTGD---KTDSEA---TNIYLQLSK
KDP--IIKLLYVTPE-KICAS---------------NRLISTLENLYERRLLARFVIDEA
HCV--SQWG------HDFRQDYKRMNMLRQKF--PS-VPVMALTATAN----------PR
VQKDILTQLKIL----RPQVFSM--SFNRHNLKYYVL---PK-------------KPKKV
AF--DCLEWIRKH-YPY----DSGIIYC-----LSRREC--------DTM---------A
DTL----------------------------QKW----INQDG-CQ----VICATIAFGM
GIDKPDVRFVIHASLPKS-MEGYYQESGRAG-RDGEISHCLLF-YTYHDVTRLKRLI---
--------MMEKDG--NHHTR--------ETHFNNLYSM--VHYCENITE----------
CRRI---QLLAYFGE--NGFNPDFCKKHADV--SCDNC--CK----TKDYKTR--DVTDD
VKSIIRFVQEHSS-----SQGARNIKHIGP-SGRFTMNML-VD-IFLGSKSAKIQS----
------GIFGKGSAYSRHNAERLFKKLILDKILDEDLYINAN-DQAI--AYMMLGNKAQG
VLNGN----L-------------K---VDFMETENSSSVKKQK----------ALVAKVS
Q--RE--E
MVKKCLGELTEVCKSLGKVFGV-HYFNIFNTVTLKKLA--ESLSSDPEVLLQIDGVTEDK
LEKYGAEVIAVLQKYSE----------------WTLPAEDSSP--GISLSGSRGP---GK
SATEK-----LDEEIPV-------SSHYFANNTGNERKRK--RMPASQRSKRRKTAS-GG
SK--AKG---------GSATRRKM--SSRTKSS-IAGSSSASHAS-----------QATS
---------------------GANRK----------------------------------

--LGIMAPP-
--KP--------------
---------INRPFLKPSYAFA-----------------
>F7CZ93

```
------------------------------------------------------------
------------------------------------------------------------
------------------------------------------------------------
------------------------------------------------------------
------------------------------------------------------------
------------------------------------------------------------
------------------------------------------------------------
------------------------------------------------------------
------------------------------------------------------------
------------------------------------------------------------
------------------------------------------------------------
------------------------------------------------------------
------------------------------------------------------------
------------------------------------------------------------
------------------------------------------------------------
------------------------------------------------------------
------------------------------------------------------------
------------------------------------------------------------
------------------------------------------------------------
------------------------------------------------------------
------------------------------------------------------------
------------------------------------------------------------
------------------------------------------------------------
---------MMKIFHKKFGLHHFRTNQLEAINAALLG--EDCFIL---MPT--GGGKSLC
--------YQLPA---------------------CI----SPGV-SIVISPLRSLIVDQV
QKLTS------LDI-----------------PATYLTGD---KTDSEA---ASIYLQLSK
KDP--IIKLLYVTPE-KVCAS---------------NRLISTLENLYERKLLARFVIDEA
HCV--SQWG------HDFRQDYKRMNVLRQKF--PS-VPVMALTATAN----------PR
VQKDILTQLKIL----KPQVFSM--SFNRHNLKYDVL---PK-------------KPKKV
AF--DCLSWIRKH-HPY----DSGIIYC-----LSRREC--------DTM---------A
LTL---RKDGLAALA-YHAGLSDSAR-DEVQHKW----INQDG-CQ----VICATIAFGM
GIDKPDVRYVIHASLPKS-VEGYYQESGRAG-RDGEISHCLLF-YSYHDVIRLKRLI---
--------LMEKDG--NSHTR--------QTHFNNLYSM--VHYCENIAE----------
CRRI---QLLSYFGE--NGFNPNFCKEHLDV--ACDNC--CQ----KKDYKSR--DVTED
VKNIVRFVQELDS-----LQGRRNMRNITS-PGRFTMNML-VD-IFLGSKSAKVQS----
------GIFGKGSAYSRHNAERLFKKLVLDKILDEDLFINAN-DQPI--AYIVAGERAQA
VLHGY----S-------------K---VDFIETENASSIRKQK----------ALVAKVS
E------------------------------------------------------RE--E
VVKKCLGELTEVCKALGKVFGV-HYFNIFNTVTLKKVA--ESLSSDPEVLLQIDGVTEDK
LEKYGAEVIGVLQKYSE----------------WTLPAGGDDK--DPSWLSPHEEEFGGR
SVSQE-----DGEEVHV-------ASRYFGSKTERGTKRK--RTLAFQRAKRKKTGP-GG
QKSNAKG---------GPNTCRKT-PSSKIKASRSFGSQSTSDGF-----------QTAA
---------------------GSGRK----------------------------------
------------------------------------------------------------
------------------------------------------------------------
------------------------------------------------------------
------------------------------------------------------------
```

```
------------------------------------------------------------
------------------------------------------------------------
----------------------------------------------------LGLMALP-
--------------------------------------------KP--------------
---------KNRQFLQPSYTFL-----------------
>G5B1S9
--------------------------------MAAIPLNNLQEQL---------------
------------------------------------------------------------
--ERHS-ARKLNNKSD--LSKPKP--------CGFT------------------------
---------FKKKTTSDND---ASVTSV--------SIAKTPVLS--------DKDVNVS
EVFSFTEPPP-HTPSQQ----PRINGFFKNAAVGQQGQRTGS------KLLLTGLLQI--
--PQEA-LPTT--Q---NISAVKK----PHNAILKKLEFSSSSDSFHTM-NDWDDM-DDF
D--TSETSKVFATP----PKNHL--VRVSTAQKSKKAKRNFF--------KA---QP---
HKTN-------------TVKADLTP----SESQPVDLTKEEKDD---------SEW---L
KSGVICI-DDDSIPQVHTDE----------DTQERHSLKIHLGDER-------------D
NNE-KHNEED--------VELLSSEK------ATCVEL----NDDDY-IDFVP---PSPE
EESI-------SASSSSL---------------KCFSALKDLDASDQEEDVLS-TSKDLL
SKPEKMTTQEPNP--EISTDCDARQISLQQQLLH--------------------------
----------------------VMEHICK-------------------------------
------------------------------------------LVDTIPVDELKALD----
---------------------CGEELLQQQNRR----------------------RKLLA
--E-AGFTRNDAS-LLG----SV----WRYRPG---------SLSSLV------------
-------------ETDSYPTRS--SVKDLS-----------VLPLPSHSLSTREC-----
------------------LLTTTPG---------------------------------KT
GFLA-TMSTLPERPLFSS-----------------HLQKSFVSNNWAETPRIEKRN----
----------ESSYFPGNVLTSTAVK----DQNKHAASV----------------NDL-E
RENQASHDVDNFNI---DDF--DDD---------DDDWENIM-HDLA-------------
--ASKPSTAAYQPIK--KGGPLKSVSE-KI---SAAKTH------------------CLP
GASVA---------QH--HTDKLTQNLAS--RNIKQ-----ECFQSL-----S-FPH---
------TKEMMKIFHKKFGLHNFRTNQLEAINAALLG--EDCFIL---MPT--GGGKSLC
--------YQLPA---------------------CV----SPGV-TIVISPLRSLIVDQV
QKLTS------LDI-----------------PATYLTGD---KTDSEA---ASIYLQLSK
KDP--IIKLLYVTPE-KVCAS---------------NRLISTLENLYERKLLARFVIDEA
HCV--SQWG------HDFRQDYKRMNMLRQKF--PS-VPMMALTATAN----------PR
VQTDILTQLKIL----RPQV----------------------------------------
------------------------------------------------------------
------------------------------------------------------------
------------------------------------------------------------
------------------------------------------------------------
------------------------------------------------------------
------------------------------------------------------------
------------------------------------------------------------
------------------------------------------------------------
------------------------------------------------------------
------------------------------------------------------------
------------------------------------------------------------
------------------------------------------------------------
```

```
------------------------------------------------------------
------------------------------------------------------------
------------------------------------------------------------
------------------------------------------------------------
------------------------------------------------------------
------------------------------------------------------------
------------------------------------------------------------
------------------------------------------------------------
------------------------------------------------------------
------------------------------------------------------------
---------------------------------------
>H2NP72
--------------------------------MAAVPQNNLQEQL---------------
------------------------------------------------------------
--ERHS-ARTLNNKLS--LSKPKF--------SGFT------------------------
---------FKKKTSLDNN---VSVTNV--------SVAKTPVLR--------NKDVNVT
EDFSFSEPLP-NTTNQQ-----RIKDFFKNAPAGQQTQRVES------KPLLPDFLQT--
--PKEV-LCTT--Q---NTPTVKK----SWDTALKRLEFSSSPDSLSTI-NDWDDM-DDF
D--TSETSKSFVTP----PQSHF--VRVSTAQKSKKGKRNFF--------KA---QL---
YTTN-------------TVKADLPPPS--SESEQIDLTEEQKDD---------SEW---L
SSDVICI-DDGPIAEVHINE----------DAQESDSLKTHLEDER-------------D
NSKKKKNLEE--------AELHSTEK------VPCIEF----DDDDYDTDFVP---PSP-
EEII-------SASSSSS---------------KCLSMLKDLDTSDRKEDVLS-TSKDLL
SKPEKMSMQEPNP--ETSTDYDVGQISLQQQLIY--------------------------
----------------------VMEHICK-------------------------------
------------------------------------------LIDTIPDDKLKLLD----
---------------------CGNELLQQRNIR----------------------RKLLT
--E-VDFNKSDAS-LLG----SM----WRCRPD---------SLDGPM------------
-------------EGDSCPTGN--SMKELN-----------FSHLPSNAVSLGTV-----
------------------TDYHXLG---------------------------------RQ
DL-C-HRKNLFDRPLF-I-----------------PIYRSLVSSNWAETPRLG-KN----
----------ESSYFPGNVLTSTAVK----DQNKHTASI----------------NDL-E
RETQPSYDIDNFDI---DDF---DD---------DDDWENIM-HNLA-------------
--ASKSSTAAYQPIK--EGRPIKSVSE-RL---SSAKTN------------------CLP
VASTAQNINFSESIQN--YTDKSAQNLAS--RNLKH-----ERFQSL-----S-FPH---
------TKEMMKIFHKKFGLHNFRTNQLEAINAALLG--EDCFIL---MPT--GGGKSLC
--------YQLPA---------------------CV----SPGV-TIVISPLRSLIVDQV
QXXXX------XXI-----------------PATYLTGD---KTDSEA---TNIYLQLSK
KDP--IIKLLYVTPE-KICAS---------------NRLVSTLENLYERKLLARFVIDEA
HCV--SQWG------HDFRQDYKRMNMLRQKF--PS-VPVMALTATAN----------PR
VQKDILTQLKIL----RPQVFSM--SFNRHNLKYYVL---PK-------------KPKKV
AF--DCLEWIRKH-HPY----DSGIIYC-----LSRREC--------DTM---------A
DTL---QRDGLAALA-YHAGLSDSAR-DEVQQKW----INQDD-CQ----VICATIAFGM
GIDKPDVRFVIHASLPKS-MEGYYQESGRAG-RDGEMSHCLLF-YTYHDVTRLKRLI---
--------MMEKDG--NHHTR--------ETHFNNLYSM--VHYCENITE----------
CRRI---QLLAYFGE--NGFNPDFCKKHPDV--SCDNC--CK----TKDYKTR--DVTDD
VKSIVRFVQEHSS-----SQGTRNIKHVGP-SGRFTMNML-VD-IFLGSKSAKLQS----
```

```
------GIFGKGSAYSRHNAERLFKKLILDKILDEDLYINAN-DQAI--AYVMLGDKAQT
VLNGA----L-------------K---VDFMETENSSSVKKQK----------ALVAKVS
Q------------------------------------------------------RE--E
MVKKCLGELTEVCKSLGKVFGV-HYFNIFNTVTLKKLA--ESLSSDPEVLLQIDGVTEDK
LEKYGAEVISVLQKYSE----------------WTSPAEDSSP--GISLSSSRGP---GR
SATEE-----LDEEIPV-------SSHYFASKTRNERKRK--KMPASQRSKRRKTAS-SG
SK--AKG---------GSATCRKI--SSKMKSSSIIGSSSASHTS-----------QATS
---------------------GANSK----------------------------------
------------------------------------------------------------
------------------------------------------------------------
------------------------------------------------------------
------------------------------------------------------------
------------------------------------------------------------
------------------------------------------------------------
----------------------------------------------------LGIMAPP-
--------------------------------------------KP--------------
---------INRPFLKPSYAFS-----------------
>A0A0D9RWU3
--------------------------------MAAVPQNNLQEQL---------------
------------------------------------------------------------
--ERHS-ARTLNNKLS--LSKPKF--------SGFT------------------------
---------FKKKTSSDNN---VSVTNV--------SVAKTPVLRNKDVNVTENKDVNVT
EDFSFSEPPP-DTTNQQ-----RVKDFFKNAPAGQQTQRVGS------KPLLPDFLQT--
--PKEV-LCTT--Q---NTPTVKT----SQNTALKKLEFSSSPDSFRTI-NDWDDM-DDF
D--TSGTSKAFVTP----PQSHF--VRGSTAQKSKKAKRNFF--------KA---QL---
YTTN-------------TVKADLPPSF--SESEQIDLTKEQKDD---------SEW---L
SSDVICI-DDGPIGEVHINE----------DAQESDSLKTHLEDER-------------D
NSE-KKNLEE--------AELHSTEK------VPCIEF----DDDDYDIDFVP---PSP-
EE-I-------SASYSSS---------------KCPSMLKDLDTSDRKEDVLS-TSKDLL
SKPEKMSTQEPNP--ETSTDCDARQINLQQQLIH--------------------------
----------------------VMDHICK-------------------------------
------------------------------------------LIDTIPADKLKLLD----
---------------------CGNELLQQRNIR----------------------RKLLT
--E-VDFNKSDAS-LLG----SV----WRCRPD---------SLDGPV------------
-------------EGDSCSAGN--SMKELN-----------FSHLPSNSVSPGDY-----
------------------LLTTTLG---------------------------------KT
GFSA-TRENLFERPSFNS-----------------PLQKSFVSSNWAETPRLGKKN----
----------ESSYFPGNVLTSTAVK----DQNKHTASI----------------NDL-E
RETQPSYDIDNFDI---DDF---DD---------DDDWENIM-HNLA-------------
--ASKSSTAAYQPIK--EGRPVKSVSE-RL---SSAKTN------------------CLP
VASTAQNINFSESIQN--YTDKSAQNLAS--RNLKH-----ERFQSL-----S-FPH---
------TKEMMKIFHKKFGLHNFRTNQLEAINAALLG--EDCFIL---MPT--GGGKSLC
--------YQLPA---------------------CV----SPGV-TIVISPLRSLIVDQV
QKLTS------LDI-----------------PATYLTGD---KTDSEA---TNIYLQLSK
KDP--IIKLLYVTPE-KICAS---------------NRLISTLENLYERKLLARFVIDEA
HCV--SQWG------HDFRQDYKRMNMLRQKF--PS-VPVMALTATAN----------PR
VQKDILTQLKIL----RPQVFSM--SFNRHNLKYYVL---PK-------------KPKKV
```

AF--DCLEWIRKH-YPY----DSGIIYC-----LSRREC--------DTM---------A
DTL---QRDGLAALA-YHAGLSDSAR-DEVQQKW----INQDG-CQ----VICATIAFGM
GIDKPDVRFVIHASLPKS-MEGYYQESGRAG-RDGEISHCLLF-YTYHDVTRLKRLI---
--------IMEKDG--NHHTR--------ETHFNNLYSM--VHYCENITE----------
CRRI---QLLAYFGE--NGFNPDFCKKHPDV--SCDNC--CK----TKDYKTR--DVTDD
VKSIIRFVQEHSS-----SQGTRNIKHVGP-SGRFTMNML-VD-IFLGSKSAKIQS----
------GIFGKGSAYSRHNAERLFKKLILDKILDEDLYINAN-DQAI--AYVMLGNKAQT
VLNGN----L-------------K---VDFMETENSSSVKKQK----------ALVTKVS
Q--RE--E
MVKKCLGELTEVCKSLGKVFGV-HYFNIFNTVTLKKLA--ESLSSDPEVLLQIDGVTEDK
LEKYGAEVISVLQKYSE----------------WTSPAEDSSP--GMSLSSSRGP---GR
STAEE-----LNEEISV-------SSHYFTSKTRNERKRK--KMPASQRPKRRKTAS-SG
SK--AKG---------GSATCRKI--SSKTKSSSIIGSSSASHTS-----------QVTS
---------------------GANRK----------------------------------

--LGIMAPP-
--KP--------------
---------INRPFLKPSYAFS-----------------
>A0A096NNB3
--------------------------------MAAVPQNNLQEQL---------------

--ERHS-ARTLNNKLS--LSKPKF--------SGFT------------------------
---------FKKKTSSDNN---VSVTNV--------SVAKTPVLR--------NKDVNVT
EDFSFSEPPP-DTTNQQ-----RVKDFFKNAPAGQQTQRVGS------KPLLPDFLQT--
--PKEV-LCTT--Q---NTPTVKK----SRNTALKKLEFSSSPDSFRTI-NDWDDM-DDF
D--TSGTSKAFVTP----PQSHF--VRVSTAQKSKKAKRNFF--------KA---QL---
YTTN-------------TVKADLPPSF--SESEQIDLTKEQKDD---------SEW---L
SSDVICI-DDGPIGEVHINE----------DAQESDSLKTHLEDER-------------D
NSEKKKNLEE--------AELHSTEK------VPCIEF----DDDDYDIDFVP---PSP-
EE-I-------SASYSSS---------------KCLSMLKDLDTSDRKEDVLS-TSKDLL
SKPEKMSTQEPNP--ETSTDCDARQINLQQQLIH--------------------------
----------------------VMDHICK-------------------------------
--LIDTIPADKLKLLD----
---------------------CGNELLQQRNIR----------------------RKLLT
--E-VDFNKSDAS-LLG----SV----WRCRPD---------SLDGPV------------
-------------EGDSCSAGN--SMKELN-----------FSHLPSNSVSPGDY-----
------------------LLTTTLG---------------------------------KT
GFSA-TRENLFERPSFNT-----------------PLQKSFVSSNWAETPRLGKKN----
----------ESSYFPGNVLTSTAVK----DQNKHTASI----------------NDL-E
RETQPSYDIDNFDI---DDF---DD---------DDDWENIM-HNLA-------------
--ASKSSTAAYQPIK--EGRPIKSVSE-RL---SSAKTN------------------CLP
VASTAQNINFSESIQN--HTDKSAQNLAS--RNLKH-----ERFQSL-----S-FPH---

------TKEMMKIFHKKFGLHNFRTNQLEAINAALLG--EDCFIL---MPT--GGGKSLC
--------YQLPA---------------------CV----SPGV-TIVISPLRSLIVDQV
QKLTS------LDI-----------------PATYLTGD---KTDSEA---TNIYLQLSK
KDP--IIKLLYVTPE-KICAS---------------NRLISTLENLYERKLLARFVIDEA
HCV--SQWG------HDFRQDYKRMNMLRQKF--PS-VPVMALTATAN----------PR
VQKDILTQLKIL----RPQVFSM--SFNRHNLKYYVL---PK-------------KPKKV
AF--DCLEWIRKH-YPY----DSGIIYC-----LSRREC--------DTM---------A
DTL---QRDGLAALA-YHAGLSDSAR-DEVQQKW----INQDG-CQ----VICATIAFGM
GIDKPDVRFVIHASLPKS-MEGYYQESGRAG-RDGEISHCLLF-YTYHDVTRLKRLI---
--------IMEKDG--NHHTR--------ETHFNNLYSM--VHYCENITE----------
CRRI---QLLAYFGE--NGFNPDFCKKHPDV--SCDNC--CK----TKDYKTR--DVTDD
VKSIIRFVQEHSS-----SQGTRNIKHVGP-SGRFTMNML-VD-IFLGSKSAKIQS----
------GIFGKGSAYSRHNAERLFKKLILDKILDEDLYINAN-DQAI--AYVMLGNKAQT
VLNGN----L-------------K---VDFMETENSSSVKKQK----------ALVAKVS
Q--RE--E
MVKKCLGELTEVCKSLGKVFGV-HYFNIFNTVTLKKLA--ESLSSDPEVLLQIDGVTEDK
LEKYGAEVISVLQKYSE----------------WTSPAEDSSP--GMSLSGSRGP---GR
STAEE-----LNEEISV-------SSHYFTSKTRNERKRK--KMPASQRPKRRKTAS-SG
SK--AKG---------GSATCRKI--SSKTKSSSIIGSSSASHTS-----------QVTS
---------------------GANRK----------------------------------

--LGIMAPP-
--KP--------------
---------INRPFLKPSYAFS-----------------
>G7MVY7
--------------------------------MAAVPQNNLQEQL---------------

--ERHS-ARTLNNKLS--LSKPKF--------SGFT------------------------
---------FKKKTSSDNN---VSVTNV--------SVAKTPVLR--------NKDVNVT
EDFSFSEPPP-DTTNQQ-----RVKDFFKNAPAGQQTQRVGS------KPLLPDFLQT--
--PKEV-LCTT--Q---NTPTVKK----SQNTALKKLEFSSSPDSFRTI-NDWDDM-DDF
D--TSRTSKAFVTP----PQSHF--VRVSTAQKSKKAKRNFF--------KA---QL---
YTTN-------------TVKADLPPSF--SESEQIDLTKEQKDD---------SEW---L
SSDVICI-DDGPIGEVHINE----------DAQESDSLKTHLEDER-------------D
NSEKKKNLEE--------AELHSTEK------VPCIEF----DDDDYDIDFVP---PSP-
EE-I-------SASSSSS---------------KCLSMLKDLDTSDRKEDVLS-TSKDLL
SKPEKMSTQEPNP--ETSTDCDARQINLQQQLIH--------------------------
----------------------VMDHICK-------------------------------
--LIDNIPADKLKLLD----
---------------------CGNELLQQRNIR----------------------RKLLT
--E-VDFNKSDAS-LLG----SV----WRCRPD---------SLDGPV------------
-------------EGDSCSAGN--SMKELN-----------FSHLPSNSVSPGDY-----

------------------LLTTTLG---------------------------------KT
GFSA-TRENLFERPSFNT-----------------PLQKSFVSSNWAETPRLGKKN----
----------ESSYFPGNVLTSTAVK----DQNKHTASI----------------NDL-E
RETQPSYDIDNFDI---DDF---DD---------DDDWENIM-HNLA-------------
--ASKSSTAAYQPIK--EGRPIKSVSE-RL---SSAKTN------------------CLP
VASTAQNINFSESIQN--YTDKSAQNLAS--RNLKH-----ERFQSL-----S-FPH---
------TKEMMKIFHKKFGLHNFRTNQLEAINAALLG--EDCFIL---MPT--GGGKSLC
--------YQLPA---------------------CV----SPGV-TIVISPLRSLIVDQV
QKLTS------LDI-----------------PATYLTGD---KTDSEA---TNIYLQLSK
KDP--IIKLLYVTPE-KICAS---------------NRLISTLENLYERKLLARFVIDEA
HCV--SQWG------HDFRQDYKRMNMLRQKF--PS-VPVMALTATAN----------PR
VQKDILTQLKIL----RPQVFSM--SFNRHNLKYYVL---PK-------------KPKKV
AF--DCLEWIRKH-YPY----DSGIIYC-----LSRREC--------DTM---------A
DTL---QRDGLAALA-YHAGLSDSAR-DEVQQKW----INQDG-CQ----VICATIAFGM
GIDKPDVRFVIHASLPKS-MEGYYQESGRAG-RDGEISHCLLF-YTYHDVTRLKRLI---
--------IMEKDG--NHHTR--------ETHFNNLYSM--VHYCENITE----------
CRRI---QLLAYFGE--NGFNPDFCKKHPDV--SCDNC--CK----TXDYKTR--DVTDD
VKSIIRFVQEHSS-----SQGMRNVKHVGP-SGRFTMNML-VD-IFLGSKSAKIQS----
------GIFGKGSAYSRHNAERLFKKLILDKILDEDLYINAN-DQAI--AYVMLGNKAQT
VLNGN----L-------------K---VDFMETENSSSVKKQK----------ALVAKVS
Q--RE--E
MVKKCLGELTEVCKSLGKVFGV-HYFNIFNTVTLKKLA--ESLSSDPEVLLQIDGVTEDK
LEKYGAEVISVLQKYSE----------------WTSPAEDNSP--GMSLSGSRGP---GR
STAEE-----LDEEISV-------SSHYFTSKTRNERKRK--KMPASQRPKRRKTAS-SG
SK--AKG---------GSATCRKI--SSKTKSSSIIGSSSASHTS-----------QVTS
---------------------GANRK----------------------------------

--LGIMAPP-
--KP--------------
---------INRPFLKPSYAFS-----------------
>F7CS38
--------------------------------MAAVPQNNLQEQL---------------

--ERHS-ARTLNNKLS--LSKPKF--------SGFT------------------------
---------FKKKTSSDNN---VSVTNV--------SVAKTPVLR--------NKDVNVT
EDFSLSEPPP-DTTNQQ-----RVKDFFKNAPAGQQTQRVGS------TPLLPDFLQT--
--PKEV-LCTT--Q---NTPTVKK----SQNTALKKLEFSSSPDSFRTI-NDWDDM-DDF
D--TSGTSKAFVTP----PQSHF--VRVSTAQKSKKAKRNFF--------KA---QL---
YTTN-------------TVKADLPPSF--SESEQIDLTKEQKDD---------SEW---L
SSDVICI-DDGPIGEVHINE----------DAQESDSLKTHLEDER-------------D
NSEKKKNLEE--------AELHSTEK------VPCIEF----DDDDYDIDFVP---PSP-
EE-I-------SASSSSS---------------KCLSMLKDLDTSDRKEDVLS-TSKDLL

SKPEKMSTQEPNP--ETSTDCDARQINLQQQLIH--------------------------
----------------------VMDHICK-------------------------------
--LIDTIPADKLKLLD----
---------------------CGNELLQQRNIR----------------------RKLLT
--E-VDFNKSDAS-LLG----SV----WRCRPD---------SLDGPV------------
-------------EGDSCSAGN--SMKELN-----------FSHLPSNSVSPGDY-----
------------------LLTTTLG---------------------------------KT
GFSA-TRENLFERPSFNT-----------------PLQKSFVSSNWAETPRLGKKN----
----------ESSYFPGNVLTSTAVK----DQNKHTASI----------------NDL-E
RETQPSYDIDNFDI---DDF---DD---------DDDWENIM-HNLA-------------
--ASKSSTAAYQPIK--EGRPIKSVSE-RL---SSAKTN------------------CLP
VASTAQNINFSESIQN--YTDKSAQNLAS--RNLKH-----ERFQSL-----S-FPH---
------TKEMMKIFHKKFGLHNFRTNQLEAINAALLG--EDCFIL---MPT--GGGKSLC
--------YQLPA---------------------CV----SPGV-TIVISPLRSLIVDQV
QKLTS------LDI-----------------PATYLTGD---KTDSEA---TNIYLQLSK
KDP--IIKLLYVTPE-KICAS---------------NRLISTLENLYERKLLARFVIDEA
HCV--SQWG------HDFRQDYKRMNMLRQKF--PS-VPVMALTATAN----------PR
VQKDILTQLKIL----RPQVFSM--SFNRHNLKYYVL---PK-------------KPKKV
AF--DCLEWIRKH-YPY----DSGIIYC-----LSRREC--------DTM---------A
DTL---QRDGLAALA-YHAGLSDSAR-DEVQQKW----INQDG-CQ----VICATIAFGM
GIDKPDVRFVIHASLPKS-MEGYYQESGRAG-RDGEISHCLLF-YTYHDVTRLKRLI---
--------IMEKDG--NHHTR--------ETHFNNLYSM--VHYCENITE----------
CRRI---QLLAYFGE--NGFNPDFCKKHPDV--SCDNC--CK----TKDYKTR--DVTDD
VKSIIRFVQEHSS-----SQGMRNVKHVGP-SGRFTMNML-VD-IFL-------------

--ESLSSDPEVLLQIDGVTEDK
LEKYGAEVISVLQKYSE----------------WTSPAEDNSP--GMSLSGSRGP---GR
STAEE-----LDEEISV-------SSHYFTSKTRNERKRK--KMPASQRPKRRKTAS-SG
SK--AKG---------GSATCRKI--SSKTKSSSIIGSSSASHTS-----------QVTS
---------------------GANRK----------------------------------

--LGIMAPP-
--KP--------------
---------INRPFLKPSYAFS-----------------
>A0A1D5R692
--------------------------------MAAVPQNNLQEQL---------------

--ERHS-ARTLNNKLS--LSKPKF--------SGFT------------------------
---------FKKKTSSDNN---VSVTNV--------SVAKTPVLR--------NKDVNVT
EDFSLSEPPP-DTTNQQ-----RVKDFFKNAPAGQQTQRVGS------TPLLPDFLQT--

```
--PKEV-LCTT--Q---NTPTVKK----SQNTALKKLEFSSSPDSFRTI-NDWDDM-DDF
D--TSGTSKAFVTP----PQSHF--VRVSTAQKSKKAKRNFF--------KA---QL---
YTTN-------------TVKADLPPSF--SESEQIDLTKEQKDD---------SEW---L
SSDVICI-DDGPIGEVHINE----------DAQESDSLKTHLEDER-------------D
NSEKKKNLEE--------AELHSTEK------VPCIEF----DDDDYDIDFVP---PSP-
EE-I-------SASSSSS---------------KCLSMLKDLDTSDRKEDVLS-TSKDLL
SKPEKMSTQEPNP--ETSTDCDARQINLQQQLIH--------------------------
----------------------VMDHICK-------------------------------
------------------------------------------LIDTIPADKLKLLD----
---------------------CGNELLQQRNIR----------------------RKLLT
--E-VDFNKSDAS-LLG----SV----WRCRPD---------SLDGPV------------
-------------EGDSCSAGN--SMKELN-----------FSHLPSNSVSPGDY-----
------------------LLTTTLG---------------------------------KT
GFSA-TRENLFERPSFNT-----------------PLQKSFVSSNWAETPRLGKKN----
----------ESSYFPGNVLTSTAVK----DQNKHTASI----------------NDL-E
RETQPSYDIDNFDI---DDF---DD---------DDDWENIM-HNLA-------------
--ASKSSTAAYQPIK--EGRPIKSVSE-RL---SSAKTN------------------CLP
VASTAQNINFSESIQN--YTDKSAQNLAS--RNLKH-----ERFQSL-----S-FPH---
------TKEMMKIFHKKFGLHNFRTNQLEAINAALLG--EDCFIL---MPT--GGGKSLC
--------YQLPA---------------------CV----SPGV-TIVISPLRSLIVDQV
QKLTS------LDI-----------------PATYLTGD---KTDSEA---TNIYLQLSK
KDP--IIKLLYVTPE-KICAS---------------NRLISTLENLYERKLLARFVIDEA
HCV--SQWG------HDFRQDYKRMNMLRQKF--PS-VPVMALTATAN----------PR
VQKDILTQLKIL----RPQVFSM--SFNRHNLKYYVL---PK-------------KPKKV
AF--DCLEWIRKH-YPY----DSGIIYC-----LSRREC--------DTM---------A
DTL---QRDGLAALA-YHAGLSDSAR-DEVQQKW----INQDG-CQ----VICATIAFGM
GIDKPDVRFVIHASLPKS-MEGYYQESGRAG-RDGEISHCLLF-YTYHDVTRLKRLI---
--------IMEKDG--NHHTR--------ETHFNNLYSM--VHYCENITE----------
CRRI---QLLAYFGE--NGFNPDFCKKHPDV--SCDNC--CK----TKDYKTR--DVTDD
VKSIIRFVQEHSS-----SQGMRNVKHVGP-SGRFTMNML-VD-IFLGSKSAKIQS----
------GIFGKGSAYSRHNAERLFKKLILDKILDEDLYINAN-DQAI--AYVMLGNKAQT
VLNGN----L-------------K---VDFMETENSSSVKKQK----------ALVAKVS
Q------------------------------------------------------RE--E
MVKKCLGELTEVCKSLGKVFGV-HYFNIFNTVTLKKLA--ESLSSDPEVLLQIDGVTEDK
LEKYGAEVISVLQKYSE----------------WTSPAEDNSP--GMSLSGSRGP---GR
STAEE-----LDEEISV-------SSHYFTSKTRNERKRK--KMPASQRPKRRKTAS-SG
SK--AKG---------GSATCRKI--SSKTKSSSIIGSSSASHTS-----------QVTS
---------------------GANRK----------------------------------
------------------------------------------------------------
------------------------------------------------------------
------------------------------------------------------------
------------------------------------------------------------
------------------------------------------------------------
------------------------------------------------------------
----------------------------------------------------LGIMAPP-
--------------------------------------------KP--------------
---------INRPFLKPSYAFS-----------------
```

>H9ZC96
--------------------------------MAAVPQNNLQEQL---------------

--ERHS-ARTLNNKLS--LSKPKF--------SGFT------------------------
---------FKKKTSSDNN---VSVTNV--------SVAKTPVLR--------NKDVNVT
EDFSLSEPPP-DTTNQQ-----RVKDFFKNAPAGQQTQRVGS------KPLLPDFLQT--
--PKEV-LCTT--Q---NTPTVKK----SQNTALKKLEFSSSPDSFRTI-NDWDDM-DDF
D--TSGTSKAFVTP----PQSHF--VRVSTAQKSKKAKRNFF--------KA---QL---
YTTN-------------TVKADLPPSF--SESEQIDLTKEQKDD---------SEW---L
SSDVICI-DDGPIGEVHINE----------DAQESDSLKTHLEDER-------------D
NSEKKKNLEE--------AELHSTEK------VPCIEF----DDDDYDIDFVP---PSP-
EE-I-------SASSSSS---------------KCLSMLKDLDTSDRKEDVLS-TSKDLL
SKPEKMSTQEPNP--ETSTDCDARQINLQQQLIH--------------------------
----------------------VMDHICK-------------------------------
--LIDTIPADKLKLLD----
---------------------CGNELLQQRNIR----------------------RKLLT
--E-VDFNKSDAS-LLG----SV----WRCRPD---------SLDGPV------------
-------------EGDSCSAGN--SMKELN-----------FSHLPSNSVSPGDY-----
------------------LLTTTLG---------------------------------KT
GFSA-TRENLFERPSFNT-----------------PLQKSFVSSNWAETPRLGKKN----
----------ESSYFPGNVLTSTAVK----DQNKHTASI----------------NDL-E
RETQPSYDIDNFDI---DDF---DD---------DDDWENIM-HNLA-------------
--ASKSSTAAYQPIK--EGRPIKSVSE-RL---SSAKTN------------------CLP
VASTAQNINFSESIQN--YTDKSAQNLAS--RNLKH-----ERFQSL-----S-FPH---
------TKEMMKIFHKKFGLHNFRTNQLEAINAALLG--EDCFIL---MPT--GGGKSLC
--------YQLPA---------------------CV----SPGV-TIVISPLRSLIVDQV
QKLTS------LDI-----------------PATYLTGD---KTDSEA---TNIYLQLSK
KDP--IIKLLYVTPE-KICAS---------------NRLISTLENLYERKLLARFVIDEA
HCV--SQWG------HDFRQDYKRMNMLRQKF--PS-VPVMALTATAN----------PR
VQKDILTQLKIL----RPQVFSM--SFNRHNLKYYVL---PK-------------KPKKV
AF--DCLEWIRKH-YPY----DSGIIYC-----LSRREC--------DTM---------A
DTL---QRDGLAALA-YHAGLSDSAR-DEVQQKW----INQDG-CQ----VICATIAFGM
GIDKPDVRFVIHASLPKS-MEGYYQESGRAG-RDGEISHCLLF-YTYHDVTRLKRLI---
--------IMEKDG--NHHTR--------ETHFNNLYSM--VHYCENITE----------
CRRI---QLLAYFGE--NGFNPDFCKKHPDV--SCDNC--CK----TKDYKTR--DVTDD
VKSIIRFVQEHSS-----SQGMRNVKHVGP-SGRFTMNML-VD-IFLGSKSAKIQS----
------GIFGKGSAYSRHNAERLFKKLILDKILDEDLYINAN-DQAI--AYVMLGNKAQT
VLNGN----L-------------K---VDFMETENSSSVKKQK----------ALVAKVS
Q--RE--E
MVKKCLGELTEVCKSLGKVFGV-HYFNIFNTVTLKKLA--ESLSSDPEVLLQIDGVTEDK
LEKYGAEVISVLQKYSE----------------WTSPAEDNSP--GMSLSGSRGP---GR
STAEE-----LDEEISV-------SSHYFTSKTRNERKRK--KMPASQRPKRRKTAS-SG
SK--AKG---------GSATCRKI--SSKTKSSSIIGSSSASHTS-----------QVTS
---------------------GANRK----------------------------------


```
------------------------------------------------------------
------------------------------------------------------------
------------------------------------------------------------
----------------------------------------------------LGIMAPP-
--------------------------------------------KP--------------
---------INRPFLKPSYAFS-----------------
>G7P9G6
------------------MADSKSGAKKCTRIMAAVPQNNLQEQL---------------
------------------------------------------------------------
--ERHS-ARTLNNKLS--LSKPKF--------SGFT------------------------
---------FKKKTSSDNN---VSVTNV--------SVAKTPVLR--------NKDVNVT
KDFSFSEPPP-DTTNQQ-----RVKDFFKNAPAGQQTQRVGS------TPLLPDFLQT--
--PKEV-LCTT--Q---NTPTVKK----SQNTALKKLEFSSSPDSFRTI-NDWDDM-DDF
D--TSGTSKAFVTP----PQSHF--VRVSTAQKSKKAKRNFF--------KA---QL---
YTTN-------------TVKADLPPSF--SESEQIDLTKEQKDD---------SEW---L
SSDVICI-DDGPIGEVHINE----------DAQESDSLKTHLEDERVVNKSTFINCFTVD
NSEKKNLEE---------AELHSTEK------VPCIEF----DDDDYDIDFVP---PSP-
EE-I-------SASSSSS---------------KCLSMLKDLDTSDRKEDVLS-TSKDLL
SKPEKMSTQEPNP--ETSTDCDARQINLQQQLIH--------------------------
----------------------VMDHICK-------------------------------
------------------------------------------LIDTIPADKLKLLD----
---------------------CGNELLQQRNIR----------------------RKLLT
--E-VDFNKSDAS-LLG----SV----WRCRPD---------SLDGPV------------
-------------EGDSCSAGN--SMKELN-----------FSHLPSNSVSPGDY-----
------------------LLTTTLG---------------------------------KT
GFSA-TRENLFERPSFNT-----------------PLQKSFVSSNWAETPRLGKKN----
----------ESSYFPGNVLTSTAVK----DQNKHTASI----------------NDL-E
RETQPSYDIDNFDI---DDF---DD---------DDDWENIM-HNLA-------------
--ASKSSTAAYQPIK--EGRPIKSVSE-RL---SSAKTN------------------CLP
VASTAQNINFSESIQN--YTDKSAQNLAS--RNLKH-----ERFQSL-----S-FPH---
------TKEMMKIFHKKFGLHNFRTNQLEAINAALLG--EDCFIL---MP----------
------------------------------------------------------------
------------------------------------TGD---KTDSEA---TNIYLQLSK
KDP--IIKLLYVTPE-KICAS---------------NRLISTLENLYERKLLARFVIDEA
HCV--SQWG------HDFRQDYKRMNMLRQKF--PS-VPVMALTATAN----------PR
VQKDILTQLKIL----RPQVFSM--SFNRHNLKYYVL---PK-------------KPKKV
AF--DCLEWIRKH-YPY----DSGIIYC-----LSRREC--------DTM---------A
DTL---QRDGLAALA-YHAGLSDSAR-DEVQQKW----INQDG-CQ----VICATIAFGM
GIDKPDVRFVIHASLPKS-MEGYYQESGRAG-RDGEISHCLLF-YTYHDVTRLKRLI---
--------IMEKDG--NHHTR--------ETHFNNLYSM--VHYCENITE----------
CRRI---QLLAYFGE--NGFNPDFCKKHPDV--SCDNC--CK----TKDYKTR--DVTDD
VKSIIRFVQEHSS-----SQGMRNVKHVGP-SGRFTMNML-VD-IFLGSKSAKIQS----
------GIFGKGSAYSRHNAERLFKKLILDKILDEDLYINAN-DQAI--AYVMLGNKAQT
VLNGN----L-------------K---VDFMETENSSSVKKQK----------ALVAKVS
Q------------------------------------------------------RE--E
MVKKCLGELTEVCKSLGKVFGV-HYFNIFNTVTLKKLA--ESLSSDPEVLLQIDGVTEDK
LEKYGAEVISVLQKYSE----------------WTSPAEDNSP--GMSLSGSRGP---GR
```

STAEE-----LDEEISV-------SSHYFTSKTRNERKRK--KMPASQRPKRRKTAS-SG
SK--AKG---

>H9F6Y6

------------------------QNLAS--RNLKH-----ERFQSL-----S-FPH---
------TKEMMKIFHKKFGLHNFRTNQLEAINAALLG--EDCFIL---MPT--GGGKSLC
--------YQLPA---------------------CV----SPGV-TIVISPLRSLIVDQV
QKLTS------LDI-----------------PATYLTGD---KTDSEA---TNIYLQLSK
KDP--IIKLLYVTPE-KICAS---------------NRLISTLENLYERKLLARFVIDEA
HCV--SQWG------HDFRQDYKRMNMLRQKF--PS-VPVMALTATAN----------PR
VQKDILTQLKIL----RPQVFSM--SFNRHNLKYYVL---PK-------------KPKKV
AF--DCLEWIRKH-YPY----DSGIIYC-----LSRREC--------DTM---------A
DTL---QRDGLAALA-YHAGLSDSAR-DEVQQKW----INQDG-CQ----VICATIAFGM
GIDKPDVRFVIHASLPKS-MEGYYQESGRAG-RDGEISHCLLF-YTYHDVTRLKRLI---
--------IMEKDG--NHHTR--------ETHFNNLYSM--VHYCENITE----------
CRRI---QLLAYFGE--NGFNPDFCKKHPDV--SCDNC--CK----TKDYKTR--DVTDD

VKSIIRFVQEHSS-----SQGMRNVKHVGP-SGRFTMNML-VD-IFLGSKSAKIQS----
------GIFGKGSAYSRHNAERLFKKLILDKILDEDLYINAN-DQAI--AYVMLGNKAQT
VLNGN----L-------------K---VDFMETENSSSVKKQK----------ALVAKVS
Q--RE--E
MVKKCLGELTEVCKSLGKVFGV-HYFNIFNTVTLKKLA--ESLSSDPEVLLQIDGVTEDK
LEKYGAEVISVLQKYSE----------------WTSPAEDNSP--GMSLSGSRGP---GR
STAEE-----LDEEISV-------SSHYFTSKTRNERKRK--KMPASQRPKRRKTAS-SG
SK--AKG---------GSATCRKI--SSKTKSSSIIGSSSASHTS-----------QVTS
---------------------GANRK----------------------------------

--LGIMAPP-
--KP--------------
---------INRPFLKPSYAFS-----------------
>G1RNC4
ARSEPALGAAAVVAAREVWILVPSARSLHARIMAAVPQNNLQEQL---------------

--ERHS-ARTLNNKLS--LSKPKF--------SGFT------------------------
---------FKKKTSSDNN---VSVTNV--------SVAKTPVLR--------NKDVNVT
EDFSFSEPLP-NTTNQQ-----RVKDFFKNAPEGQQTQRVGS------KPLLPDFLQT--
--PKEV-LCAT--Q---NTPTVKK----SRDTVLKKLEFSSSPDSLSTI-NDWDDM-DDF
D--TSGTSKSFVTP----PQSHF--VRVSTAQKSKKGKRNFF--------KA---QL---
YTTN-------------TVKADLPPPS--SESEQIDLTEEQKDD---------SEW---L
SSDVICI-DDGPIAEVHINE----------DAQESDSLKTHLEDER-------------D
NSEKKKNLEE--------AELHSAEK------VPCIEF----DDDDYDTDFVP---PSP-
EEII-------SASSSSS---------------KCLSMLKDLDTSDRKEDVLS-TSKDLL
PKPEKMSMQDPNP--ETSTDCDARQISLQQQLIH--------------------------
----------------------VMEHICK-------------------------------
--LIDTIPDDKLKLLD----
---------------------CGNELLQQRNIR----------------------RKLLT
--E-VEFNKSDAS-LLG----LM----WRCRPD---------SLDGPM------------
-------------EGDSCPTGN--SMKELN-----------FSHLPSNSVSPGDC-----
------------------LLTTTLG---------------------------------KT
GFSA-TRENLFERPLFNT-----------------HLQKSFVSSNWAETPRLGKKN----
----------ESSYFPGNVLTSTAVK----DQNKHTASI----------------NDL-E
RETQPSYDIDNFDI---DDF---DD---------DDDWENIM-HNLA-------------
--ASKSSTAAYQPIK--EGRPIKSVSE-RL---SSAKTN------------------CLP
VASTAQNINFSESIQN--YTNKSAQNLAS--RNLKH-----ERFQSL-----S-FPH---
------TKEMMKIFHKKFGLHNFRTNQLEAINAALLG--EDCFIL---MPT--GGGKSLC
--------YQLPA---------------------CV----SPGD-TIEISPLRSLIVDPV
QKLTS------LDI-----------------PATYLTGD---KTDSET---TNIYLQLSK
KDP--IIKLLYVTPE-KICAS---------------NRLISTLENLYERKLLARFVIDEA
HCV--SQWG------HDFRQDYKRMNILRQKF--PS-VPVMALTATAN----------PR

VQKDILTQLKIL----RPQVFSM--SFNRHNLKYYVL---PK-------------KPKKV
AF--DCLEWIRKH-HPY----DSGIIYC-----LSRREC--------DTM---------A
DTL---QRDGLAALA-YHAGLSDSAR-DEVQQKW----INQDG-CQ----VICATIAFGM
GIDKPDVRFVIHASLPKS-VEGYYQESGRAG-RDGEISHCLLF-YTYHDVTRLKRLI---
--------MMEKDG--NHHTR--------ETHFNNLYSM--VHYCENITE----------
CRRI---QLLAYFGE--NGFNPDFCKKHPDV--SCDNC--CK----TKDYKTR--DVTDD
VKSIIRFVQEHSS-----SQGTRNIKHVGP-SGRFTMNML-VD-IFLGSKSAKLQS----
------GIFGKGSAYSRHNAERLFKKLILDKILDEDLYINAN-DQAI--AYVMLGNKAQT
VLNGN----L-------------K---VDFMETENSSSVKKQK----------ALVAKVS
Q--RE--E
MVKKCLGELTEVCKSLGKVFGV-HYFNIFNTVTLKKLA--ESLSSDPEVLLQIDGVTEDK
LEKYGAEVISVLQKYSE----------------WTSPAEDSSP--GISLSSSRVP---GR
SATEE-----LDKEIPV-------SSHYFASKTRNERKRK--KMPASQRSKRRKTAS-SG
SK--AKG---------GSATCSKI--SSKTKSSSIIGSSSASHTS-----------QATS
---------------------GANSK----------------------------------

--LGIMAPP-
--KP--------------
---------INRPFLKPSYAFS-----------------
>G3RSA8
--------------------------------MAAVPQNNLQEQL---------------

--ERHS-ARTLNNKLS--LSKPKF--------SGFT------------------------
---------FKKKTSSDNN---VSVTNV--------SVAKTPVLR--------NKDVNVT
KDFSFSEPLS-NTTNQQ-----RVKDFFKNAPAGQQTQRVES------KSLLPDFLQT--
--PKEV-LCTT--Q---NTPTIKK----SRDTALKKLEFSSSPDSLSTI-NDWDDM-DDF
D--TSETSKSFVTP----PQSHF--VRVSTAQKSKKGKRNFF--------KA---QL---
YTTN-------------TVKTDLPPP---SESEQIDLTEEQKDD---------SEW---L
SSDVICI-DDGPIAEVHINE----------DAQESDSLKTHLEDER-------------D
NSE-KKNLEE--------AELHSTEK------VPCIEF----DDDDYDTDFVP---PSP-
EEII-------SASSSSS---------------KCLSVLRDLDTSDRKEDVLS-TSKDLL
SKPEKMSMQELNP--ETSTDCDARQISLQQQLIH--------------------------
----------------------VMEHICK-------------------------------
--LIDTIPDDKLKLLD----
---------------------CGNELLQQRNIR----------------------RKLLT
--E-VDFNKSDAS-LLG----SM----WRCRPD---------SLDGPM------------
-------------EGDSWPTGN--SMKELN-----------FSHLPSNSVSPGDC-----
------------------LLTTTLG---------------------------------KT
GFSA-TRKNLFERPLFNT-----------------HLQKSFVNSNWAETPRLGKKN----
----------ESSYFPGNVLTSTAVK----DQNKHTASI----------------NDL-E
RETQPSYDIDNFDI---DDF---DD---------DDDWEDIM-HNLA-------------
--ASKSSTAAYQPIK--EGRPIKSVSE-RL---SSAKTN------------------CLP

VASTAQNINFSESIQN--YTDKSAQNLAS--RNLKH-----ERFQSL-----S-FPH---
------TKEMMKIFHKKFGLHNFRTNQLEAINAALLG--EDCFIL---MPT--GGGKSLC
--------YQLPA---------------------CV----SPGV-TIVISPLRSLIVDQV
QKLTS------LDF-----------------SFPYILKC---IHPCMP---ARIYLFFSW
LSL--RNKFIFILFT-KICAS---------------NRLISTLENLYERKLLARFVIDEA
HCV--SQWG------HDFRQDYKRMNMLRQKF--PS-VPVMALTATAN----------PR
VQKDILTQLKIL----RPQVFSM--SFNRHNLKYYVL---PK-------------KPKKV
AF--DCLEWIRKH-HPY----DSGIIYC-----LSRREC--------DTM---------A
DTL---QRDGLAALA-YHAGLSDSAR-DEVQQKW----INQDG-CQ----VICATIAFGM
GIDKPDVRFVIHASLPKS-VEGYYQESGRAG-RDGEISHCLLF-YTYHDVTRLKRLI---
--------MMEKDG--NHHTR--------ETHFNNLYSM--VHYCENITE----------
CRRI---QLLAYFGE--NGFNPDFCKKHPDV--SCDNC--CK----TKDYKSR--DVTED
VKSIVRFVQEHSS-----SQGMRNIKHVGP-SGRFTMNML-VD-IFLGSKSAKIQS----
------GIFGKGSAYSRHNAERLFKKLILDKILDEDLYINAN-DQAI--AYVMLGNKAQT
VLNGN----L-------------K---VDFMETENSSSVKKQK----------ALVAKVS
Q--RE--E
MVKKCLGELTEVCKSLGKVFGV-HYFNIFNTVTLKKLA--ESLSSDPEVLLQIDGVTEDK
LEKYGAEVISVLQKYSE----------------WTSPAEDSSP--GISLSSSRGP---GR
GAAEE-----LDEEIPV-------SSHYFASKTRNERKRK--KMPASQRSKRRKTAS-SG
SK--AKG---------GSATCRKI--SSKTKSSSIIGSSSASHTS-----------QATS
---------------------GANSK----------------------------------

--LGIMAPP-
--KP--------------
---------INRPFLKPSYAFS-----------------
>G3QXK2
--------------------------------MAAVPQNNLQEQL---------------

--ERHS-ARTLNNKLS--LSKPKF--------SGFT------------------------
---------FKKKTSSDNN---VSVTNV--------SVAKTPVLR--------NKDVNVT
KDFSFSEPLS-NTTNQQ-----RVKDFFKNAPAGQQTQRVES------KSLLPDFLQT--
--PKEV-LCTT--Q---NTPTIKK----SRDTALKKLEFSSSPDSLSTI-NDWDDM-DDF
D--TSETSKSFVTP----PQSHF--VRVSTAQKSKKGKRNFF--------KA---QL---
YTTN-------------TVKTDLPPP---SESEQIDLTEEQKDD---------SEW---L
SSDVICI-DDGPIAEVHINE----------DAQESDSLKTHLEDER-------------D
NSE-KKNLEE--------AELHSTEK------VPCIEF----DDDDYDTDFVP---PSP-
EEII-------SASSSSS---------------KCLSVLRDLDTSDRKEDVLS-TSKDLL
SKPEKMSMQELNP--ETSTDCDARQISLQQQLIH--------------------------
----------------------VMEHICK-------------------------------
--LIDTIPDDKLKLLD----
---------------------CGNELLQQRNIR----------------------RKLLT
--E-VDFNKSDAS-LLG----SM----WRCRPD---------SLDGPM------------

```
-------------EGDSWPTGN--SMKELN-----------FSHLPSNSVSPGDC-----
------------------LLTTTLG---------------------------------KT
GFSA-TRKNLFERPLFNT-----------------HLQKSFVNSNWAETPRLGKKN----
----------ESSYFPGNVLTSTAVK----DQNKHTASI----------------NDL-E
RETQPSYDIDNFDI---DDF---DD---------DDDWEDIM-HNLA-------------
--ASKSSTAAYQPIK--EGRPIKSVSE-RL---SSAKTN------------------CLP
VASTAQNINFSESIQN--YTDKSAQNLAS--RNLKH-----ERFQSL-----S-FPH---
------TKEMMKIFHKKFGLHNFRTNQLEAINAALLG--EDCFIL---MPT--GGGKSLC
--------YQLPA---------------------CV----SPGV-TIVISPLRSLIVDQV
QKLTS------LD-----------------------------------------------
-----------------ICAS---------------NRLISTLENLYERKLLARFVIDEA
HCV--SQWG------HDFRQDYKRMNMLRQKF--PS-VPVMALTATAN----------PR
VQKDILTQLKIL----RPQVFSM--SFNRHNLKYYVL---PK-------------KPKKV
AF--DCLEWIRKH-HPY----DSGIIYC-----LSRREC--------DTM---------A
DTL---QRDGLAALA-YHAGLSDSAR-DEVQQKW----INQDG-CQ----VICATIAFGM
GIDKPDVRFVIHASLPKS-VEGYYQESGRAG-RDGEISHCLLF-YTYHDVTRLKRLI---
--------MMEKDG--NHHTR--------ETHFNNLYSM--VHYCENITE----------
CRRI---QLLAYFGE--NGFNPDFCKKHPDV--SCDNC--CK----TKDYKSR--DVTED
VKSIVRFVQEHSS-----SQGMRNIKHVGP-SGRFTMNML-VD-IFLGSKSAKIQS----
------GIFGKGSAYSRHNAERLFKKLILDKILDEDLYINAN-DQAI--AYVMLGNKAQT
VLNGN----L-------------K---VDFMETENSSSVKKQK----------ALVAKVS
Q------------------------------------------------------RE--E
MVKKCLGELTEVCKSLGKVFGV-HYFNIFNTVTLKKLA--ESLSSDPEVLLQIDGVTEDK
LEKYGAEVISVLQKYSE----------------WTSPAEDSSP--GISLSSSRGP---GR
GAAEE-----LDEEIPV-------SSHYFASKTRNERKRK--KMPASQRSKRRKTAS-SG
SK--AKG---------GSATCRKI--SSKTKSSSIIGSSSASHTS-----------QATS
---------------------GANSK----------------------------------
------------------------------------------------------------
------------------------------------------------------------
------------------------------------------------------------
------------------------------------------------------------
------------------------------------------------------------
------------------------------------------------------------
----------------------------------------------------LGIMAPP-
--------------------------------------------KP--------------
---------INRPFLKPSYAFS-----------------
>B2RAN0
--------------------------------MAAVPQNNLQEQL---------------
------------------------------------------------------------
--ERHS-ARTLNNKLS--LSKPKF--------SGFT------------------------
---------FKKKTSSDNN---VSVTNV--------SVAKTPVLR--------NKDVNVT
EDFSFSEPLP-NTTNQQ-----RVKDFFKNAPAGQETQRGGS------KSLLPDFLQT--
--PKEV-VCTT--Q---NTPTVKK----SRDTALKKLEFSSSPDSLSTI-NDWDDM-DEF
D--TSETSKSFVTP----PQSHF--VRVSTAQKSKKGKRNFF--------KA---QL---
YTTN-------------TVKTDLPPPS--SESEQIDLTEEQKDD---------SEW---L
SSDVICI-DDGPIAEVHINE----------DAQESDSLKTHLEDER-------------D
NSEKKKNLEE--------AELHSTEK------VPCIEF----DDDDYDTDFVP---PSP-
```

EEII-------SASSSSS---------------KCLSTLKDLDTSDRKEDVLS-TSKDLL
SKPEKMSMQELNP--ETSTDCDARQISLQQQLIH--------------------------
----------------------VMEHICK-------------------------------
--LIDTIPDDKLKLLD----
---------------------CGNELLQQRNIR----------------------RKLLT
--E-VDFNKSDAS-LLG----SL----WRYRPD---------SLDGPM------------
-------------EGDSCPTGN--SMKELN-----------FSHLPSNSVSPGDC-----
------------------LLTTTLG---------------------------------KT
GFSA-TRKNLFERPLFNT-----------------HLQKSFVSSNWAETPRLGKKN----
----------ESSYFPGNVLTSTAVK----DQNKHTASI----------------NDL-E
RETQPSYDIDNFDI---DDF---DD---------DDDWEDIM-HNLA-------------
--ASKSSTAAYQPIK--EGRPIKSVSE-RL---SSAKTD------------------CLP
VSSTAQNINFSESIQN--YTDKSAQNLAS--RNLKH-----ERFQSL-----S-FPH---
------TKEMMKIFHKKFGLHNFRTNQLEAINAALLG--EDCFIL---MPT--GGGKSLC
--------YQLPA---------------------CV----SPGV-TVVISPLRSLIVDQV
QKLTS------LDI-----------------PATYLTGD---KTDSEA---TNIYLQLSK
KDP--IIKLLYVTPE-KICAS---------------NRLISTLENLYERKLLARFVIDEA
HCV--SQWG------HDFRQDYKRMNMLRQKF--PS-VPVMALTATAN----------PR
VQKDILTQLKIL----RPQVFSM--SFNRHNLKYYVL---PK-------------KPKKV
AF--DCLEWIRKH-HPY----DSGIIYC-----LSRREC--------DTM---------A
DTL---QRDGLAALA-YHAGLSDSAR-DEVQQKW----INQGG-CQ----VICATIAFGM
GIDKPDVRFVIHASLPKS-VEGYYQESGRAG-RDGEISHCLLF-YTYHDVTRLKRLI---
--------MMEKDG--NHHTR--------ETHFNNLYSM--VHYCENITE----------
CRRI---QLLAYFGE--NGFNPDFCKKHPDV--SCDNC--CK----TKDYKTR--DVTDD
VKSIVRFVQEHSS-----SQGMRNIKHVGP-SGRFTMNML-VD-IFLGSKSAKIQS----
------GIFGKGSAYSRHNAERLFKKLILDKILDEDLYINAN-DQAI--AYVMLGNKAQT
VLNGN----L-------------K---VDFMETENSSSVKKQK----------ALVAKVS
Q--RE--E
MVKKCLGELTEVCKSLGKVFGV-HYFNIFNTVTLKKLA--ESLSSDPEVLLQIDGVTEDK
LEKYGAEVISVLQKYSE----------------WTSPAEDSSP--GISLSSSRGP---GR
SAAEE-----LDEEIPV-------SSHYFASKTRNERKRK--KMPASQRSKRRKTAS-SG
SK--AKG---------GSATCRKI--SSKTKSSSIIGSSSASHTS-----------QATS
---------------------GANSK----------------------------------

--LGIMAPP-
--KP--------------
---------INRPFLKPSYAFS-----------------
>B7ZKN7

-----------------------MEHICK-------------------------------
--LIDTIPDDKLKLLD----
---------------------CGNELLQQRNIR----------------------RKLLT
--E-VDFNKSDAS-LLG----SL----WRYRPD---------SLDGPM------------
-------------EGDSCPTGN--SMKELN-----------FSHLPSNSVSPGDC-----
------------------LLTTTLG---------------------------------KT
GFSA-TRKNLFERPLFNT-----------------HLQKSFVSSNWAETPRLGKKN----
----------ESSYFPGNVLTSTAVK----DQNKHTASI----------------NDL-E
RETQPSYDIDNFDI---DDF---DD---------DDDWEDIM-HNLA-------------
--ASKSSTAAYQPIK--EGRPIKSVSE-RL---SSAKTD------------------CLP
VSSTAQNINFSESIQN--YTDKSAQNLAS--RNLKH-----ERFQSL-----S-FPH---
------TKEMMKIFHKKFGLHNFRTNQLEAINAALLG--EDCFIL---MPT--GGGKSLC
--------YQLPA---------------------CV----SPGV-TVVISPLRSLIVDQV
QKLTS------LDI-----------------PATYLTGD---KTDSEA---TNIYLQLSK
KDP--IIKLLYVTPE-KICAS---------------NRLISTLENLYERKLLARFVIDEA
HCV--SQWG------HDFRQDYKRMNMLRQKF--PS-VPVMALTATAN----------PR
VQKDILTQLKIL----RPQVFSM--SFNRHNLKYYVL---PK-------------KPKKV
AF--DCLEWIRKH-HPY----DSGIIYC-----LSRREC--------DTM---------A
DTL---QRDGLAALA-YHAGLSDSAR-DEVQQKW----INQDG-CQ----VICATIAFGM
GIDKPDVRFVIHASLPKS-VEGYYQESGRAG-RDGEISHCLLF-YTYHDVTRLKRLI---
--------MMEKDG--NHHTR--------ETHFNNLYSM--VHYCENITE----------
CRRI---QLLAYFGE--NGFNPDFCKKHPDV--SCDNC--CK----TKDYKTR--DVTDD
VKSIVRFVQEHSS-----SQGMRNIKHVGP-SGRFTMNML-VD-IFLGSKSAKIQS----
------GIFGKGSAYSRHNAERLFKKLILDKILDEDLYINAN-DQAI--AYVMLGNKAQT
VLNDN----L-------------K---VDFMETENSSSVKKQK----------ALVAKVS
Q--RE--E
MVKKCLGELTEVCKSLGKVFGV-HYFNIFNTVTLKKLA--ESLSSDPEVLLQIDGVTEDK
LEKYGAEVISVLQKYSE----------------WTSPAEDSSP--GISLSSSRGP---GR
SAAEE-----LDEEIPV-------SSHYFASKTRNERKRK--KMPASQRSKRRKTAS-SG
SK--AKG---------GSATCRKI--SSKTKSSSIIGSSSASHTS-----------QATS
---------------------GANSK----------------------------------

--LGIMAPP-
--KP--------------

```
---------INRPFLKPSYAFS-----------------
>H0YNU5
--------------------------------MAAVPQNNLQEQL---------------
------------------------------------------------------------
--ERHS-ARTLNNKLS--LSKPKF--------SGFT------------------------
---------FKKKTSSDNN---VSVTNV--------SVAKTPVLR--------NKDVNVT
EDFSFSEPLP-NTTNQQ-----RVKDFFKNAPAGQETQRGGS------KSLLPDFLQT--
--PKEV-VCTT--Q---NTPTVKK----SRDTALKKLEFSSSPDSLSTI-NDWDDM-DDF
D--TSETSKSFVTP----PQSHF--VRVSTAQKSKKGKRNFF--------KA---QL---
YTTN-------------TVKTDLPPPS--SESEQIDLTEEQKDD---------SEW---L
SSDVICI-DDGPIAEVHINE----------DAQESDSLKTHLEDER-------------D
NSEKKKNLEE--------AELHSTEK------VPCIEF----DDDDYDTDFVP---PSP-
EEII-------SASSSSS---------------KCLSTLKDLDTSDRKEDVLS-TSKDLL
SKPEKMSMQELNP--ETSTDCDARQISLQQQLIH--------------------------
----------------------VMEHICK-------------------------------
------------------------------------------LIDTIPDDKLKLLD----
---------------------CGNELLQQRNIR----------------------RKLLT
--E-VDFNKSDAS-LLG----SL----WRYRPD---------SLDGPM------------
-------------EGDSCPTGN--SMKELN-----------FSHLPSNSVSPGDC-----
------------------LLTTTLG---------------------------------KT
GFSA-TRKNLFERPLFNT-----------------HLQKSFVSSNWAETPRLGKKN----
----------ESSYFPGNVLTSTAVK----DQNKHTASI----------------NDL-E
RETQPSYDIDNFDI---DDF---DD---------DDDWEDIM-HNLA-------------
--ASKSSTAAYQPIK--EGRPIKSVSE-RL---SSAKTD------------------CLP
VSSTAQNINFSESIQN--YTDKSAQNLAS--RNLKH-----ERFQSL-----S-FPH---
------TKEMMKIFHKKFGLHNFRTNQLEAINAALLG--EDCFIL---MPT--GGGKSLC
--------YQLPA---------------------CV----SPGV-TVVISPLRSLIVDQV
QKLTS------LDI-----------------PATYLTGD---KTDSEA---TNIYLQLSK
KDP--IIKLLYVTPE-KICAS---------------NRLISTLENLYERKLLARFVIDEA
HCV--SQWG------HDFRQDYKRMNMLRQKF--PS-VPVMALTATAN----------PR
VQKDILTQLKIL----RPQVFSM--SFNRHNLKYYVL---PK-------------KPKKV
AF--DCLEWIRKH-HPY----DSGIIYC-----LSRREC--------DTM---------A
DTL---QRDGLAALA-YHAGLSDSAR-DEVQQKW----INQDG-CQ----VICATIAFGM
GIDKPDVRFVIHASLPKS-VEGYYQESGRAG-RDGEISHCLLF-YTYHDVTRLKRLI---
--------MMEKDG--NHHTR--------ETHFNNLYSM--VHYCENITE----------
CRRI---QLLAYFGE--NGFNPDFCKKHPDV--SCDNC--CK----TKDYKTR--DVTDD
VKSIVRFVQEHSS-----SQGMRNIKHVGP-SGRFTMNML-VD-IFL-------------
------------------------------------------------------------
------------------------------------------------------------
------------------------------------------------------------
----------------------------------------ESLSSDPEVLLQIDGVTEDK
LEKYGAEVISVLQKYSE----------------WTSPAEDSSP--GISLSSSRGP---GR
SAAEE-----LDEEIPV-------SSHYFASKTRNERKRK--KMPASQRSKRRKTAS-SG
SK--AKG---------GSATCRKI--SSKTKSSSIIGSSSASHTS-----------QATS
---------------------GANSK----------------------------------
------------------------------------------------------------
------------------------------------------------------------
```


---LGIMAPP-
---KP-------------
---------INRPFLKPSYAFS-----------------
>K7D4C4
--------------------------------MAAVPQNNLQEQL---------------

--ERHS-ARTLNNKLS--LSKPKF--------SGFT------------------------
---------FKKKTSSDNN---VSVTNV--------SVAKTPVLR--------NKDVNVT
EDVSFSEPLP-NTTNQQ-----RAKDFFKNAPAGQQTQRVGS------KSLLPDFLQT--
--PKEV-LCTT--Q---NTPTVKK----SRDTALKKLEFSSSPDSLSTI-NDWDDM-DDF
D--TSETSKSFVTP----PQSHF--VRVSTAQKSKKGKRNFF--------KA---QL---
YTTN-------------TVKTDFPPPS--SESEQIDLTEEQKDD---------SEW---L
SSDVICI-DDGPIAEVHINE----------DAQESDSLKTHLEDER-------------D
NSKKKKNLEE--------AELHSTEK------VPCIEF----DDDDYDTDFVP---PSP-
EEII-------SSSSSSS---------------KCLSMLKDLDTSDRKEDVLS-TSKDLL
SKPEKMSTQELNP--ETSTDCDARQISLQQQLIH--------------------------
----------------------VMEHICK-------------------------------
--LIDTIPDDKLKLLD----
---------------------CGNELLQQRNIR----------------------RKLLT
--E-VDFNKSDAS-LLG----SM----WRCRPD---------SLDGPM------------
-------------KGDSCPTGN--SMKELN-----------FSHLPSNSVSPGDC-----
------------------LLTTTLG---------------------------------KT
GFSA-TRKNLFERPLFNT-----------------HLQKSFVSSNWAETPRLGKKN----
----------ESSYFPGNVLTSTAVK----DQNKHTASI----------------NDL-E
RETQPSYDIDNFDI---DDF---DD---------DDDWEDIM-HNLA-------------
--ASKSSTAAYQPIK--EGRPIKSVSE-RL---SSAKTN------------------CLP
VSSTAQNINFSESIQN--YTDKSAQNLAS--GNLKH-----ERFQSL-----S-FPH---
------TKEMMKIFHKKFGLHNFRTNQLEAINAALLG--EDCFIL---MPT--GGGKSLC
--------YQLPA---------------------CV----SPGV-TVVISPLRSLIVDQV
QKLTS------LDI------------------PATYLTGD---KTDSEA---TNIYLQLSK
KDP--IIKLLYVTPE-KICAS---------------NRLISTLENLYERKLLARFVIDEA
HCV--SQWG------HDFRQDYKRMNMLRQKF--PS-VPVMALTATAN----------PR
VQKDILTQLKIL----RPQVFSM--SFNRHNLKYYVL---PK-------------KPKKV
AF--DCLEWIRKH-HPY----DSGIIYC-----LSRREC--------DTM---------A
DTL---QRDGLAALA-YHAGLSDSAR-DEVQQKW----INQDG-CQ----VICATIAFGM
GIDKPDVRFVIHASLPKS-MEGYYQESGRAG-RDGEISHCLLF-YTYHDVTRLKRLI---
--------MMEKDG--NHHTR--------ETHFNNLYSM--VHYCENITE----------
CRRI---QLLAYFGE--NGFNPDFCKKHPDV--SCDNC--CK----TKDYKTR--DVTDD
VKSIVRFVQEHSS-----SQGMRNIKHVGP-SGRFTMNML-VD-IFLGSKSAKIQS----
------GIFGKGSAYSRHNAERLFKKLILDKILDEDLYINAN-DQAI--AYVMLGNKAQT
VLNGN----L-------------K---VDFMETENSSSVKKQK----------ALVAKVS
Q---RE--E
MVKKCLGELTEVCKSLGKVFGV-HYFNIFNTVTLKKLA--ESLSSDPEVLLQIDGVTEDK

LEKYGAEVISVLQKYSE----------------WTSPAEDSSP--GISLSSSRGP---GR
SAAEE-----LDEEIPV-------SSHYFASKTRNERKRK--KMPASQRSKRRKAAS-SG
SK--AKG---------GSATCRKI--SSKTKSSSIIGSSSASHTC-----------QATS
---------------------GTNSK----------------------------------

--LGIMAPP-
--KP--------------
---------INRPFLKPSYAFS-----------------
>K7BX88
--------------------------------MAAVPQNNLQEQL---------------

--ERHS-ARTLNNKLS--LSKPKF--------SGFT------------------------
---------FKKKTSSDNN---VSVTNV--------SAAKTPVLR--------NKDVNVT
EDVSFSEPLP-NTTNQQ-----RAKDFFKNAPAGQQTQRVGS------KSLLPDFLQT--
--PKEV-LCTT--Q---NTPTVKK----SRDTALKKLEFSSSPDSLSTI-NDWDDM-DDF
D--TSETSKSFVTP----PQSHF--VRVSTAQKSKKGKRNFF--------KA---QL---
YTTN-------------TVKTDLPPPS--SETEQIDLTEEQKDD---------SEW---L
SSDVICI-DDGPIAEVHINE----------DAQESDSLKTHLEDER-------------D
NSKKKKNLEE--------AELHSTEK------VPCIEF----DDDDYDTDFVP---PSP-
EEII-------SSSSSSS---------------KCLSMLKDLDTSDRKEDVLS-TSKDLL
SKPEKTSTQELNP--ETSTDCDARQISLQQQLIH--------------------------
----------------------VMEHICK-------------------------------
--LIDTIPDDKLKLLD----
---------------------CGNELLQQRNIR----------------------RKLLT
--E-VDFNKSDAS-LLG----SM----WRCRPD---------SLDGPM------------
-------------KGDSCPTGN--SMKELN-----------FSHLPSNSVSPGDC-----
------------------LLTTTLG---------------------------------KT
GFSA-TRKNLFERPLFNT-----------------HLQKSFVSSNWAETPRLGKKN----
----------ESSYFPGNVLTSTAVK----DQNKHTASI----------------NDL-E
RETQPSYDIDNFDI---DDF---DD---------DDDWEDIM-HNLA-------------
--ASKSSTAAYQPIK--EGRPIKSVSE-RL---SSAKTN------------------CLP
VSSTAQNINFSESIQN--YTDKSAQNLAS--GNLKH-----ERFQSL-----S-FPH---
------TKEMMKIFHKKFGLHNFRTNQLEAINAALLG--EDCFIL---MPT--GGGKSLC
--------YQLPA---------------------CV----SPGV-TVVISPLRSLIVDQV
QKLTS------LDI-----------------PATYLTGD---KTDSEA---TNIYLQLSK
KDP--IIKLLYVTPE-KICAS---------------NRLISTLENLYERKLLARFVIDEA
HCV--SQWG------HDFRQDYKRMNMLRQKF--PS-VPVMALTATAN----------PR
VQKDILTQLKIL----RPQVFSM--SFNRHNLKYYVL---PK-------------KPKKV
AF--DCLEWIRKH-HPY----DSGIIYC-----LSRREC--------DTM---------A
DTL---QRDGLAALA-YHAGLSDSAR-DEVQQKW----INQDG-CQ----VICATIAFGM
GIDKPDVRFVIHASLPKS-MEGYYQESGRAG-RDGEISHCLLF-YTYHDVTRLKRLI---
--------MMEKDG--NHHTR--------ETHFNNLYSM--VHYCENITE----------

CRRI---QLLAYFGE--NGFNPDFCKKHPDV--SCDNC--CK----TKDYKTR--DVTDD
VKSIVRFVQEHSS-----SQGMRNIKHVGP-SGRFTMNML-VD-IFLGSKSAKIQS----
------GIFGKGSAYSRHNAERLFKKLILDKILDEDLYINAN-DQAI--AYVMLGNKAQT
VLNGN----L-------------K---VDFMETENSSSVKKQK----------ALVAKVS
Q--RE--E
MVKKCLGELTEVCKSLGKVFGV-HYFNIFNTVTLKKLA--ESLSSDPEVLLQIDGVTEDK
LEKYGAEVISVLQKYSE----------------WTSPAEDSSP--GISLSSSRGP---GR
SAAEE-----LDEEIPV-------SSHYFASKTRNERKRK--KMPASQRSKRRKAAS-SG
SK--AKG---------GSATCRNI--SSKTKSSSIIGSSSASHTS-----------QATS
---------------------GANSK----------------------------------

--LGIMAPP-
--KP--------------
---------INRPFLKPSYAFS-----------------
>H2QA33
--------------------------------MAAVPQNNLQEQL---------------

--ERHS-ARTLNNKLS--LSKPKF--------SGFT------------------------
---------FKKKTSSDNN---VSVTNV--------SAAKTPVLR--------NKDVNVT
EDVSFSEPLP-NTTNQQ-----RAKDFFKNAPAGQQTQRVGS------KSLLPDFLQT--
--PKEV-LCTT--Q---NTPTVKK----SRDTALKKLEFSSSPDSLSTI-NDWDDM-DDF
D--TSETSKSFVTP----PQSHF--VRVSTAQKSKKGKRNFF--------KA---QL---
YTTN-------------TVKTDLPPPS--SETEQIDLTEEQKDD---------SEW---L
SSDVICI-DDGPIAEVHINE----------DAQESDSLKTHLEDER-------------D
NSKKKKNLEE--------AELHSTEK------VPCIEF----DDDDYDTDFVP---PSP-
EKII-------SSSSSSS---------------KCLSMLKDLDTSDRKEDVLS-TSKDLL
SKPEKMSTQELNP--ETSTDCDARQISLQQQLIH--------------------------
----------------------VMEHICK-------------------------------
--LIDTIPDDKLKLLD----
---------------------CGNELLQQRNIR----------------------RKLLT
--E-VDFNKSDAS-LLG----SM----WRCRPD---------SLDGPM------------
-------------KGDSCPTGN--SMKELN-----------FSHLPSNSVSPGDC-----
------------------LLTTTLG---------------------------------KT
GFSA-TRKNLFERPLFNT-----------------HLQKSFVSSNWAETPRLGKKN----
----------ESSYFPGNVLTSTAVK----DQNKHTASI----------------NDL-E
RETQPSYDIDNFDI---DDF---DD---------DDDWEDIM-HNLA-------------
--ASKSSTAAYQPIK--EGRPIKSVSE-RL---SSAKTN------------------CLP
VSSTAQNINFSESIQN--YTDKSAQNLAS--GNLKH-----ERFQSL-----S-FPH---
------TKEMMKIFHKKFGLHNFRTNQLEAINAALLG--EDCFIL---MPT--GGGKSLC
--------YQLPA---------------------CV----SPGV-TVVISPLRSLIVDQV
QKLTS------LDI-----------------PATYLTGD---KTDSEA---TNIYLQLSK
KDP--IIKLLYVTPE-KICAS---------------NRLISTLENLYERKLLARFVIDEA

```
HCV--SQWG------HDFRQDYKRMNMLRQKF--PS-VPVMALTATAN----------PR
VQKDILTQLKIL----RPQVFSM--SFNRHNLKYYVL---PK-------------KPKKV
AF--DCLEWIRKH-HPY----DSGIIYC-----LSRREC--------DTM---------A
DTL---QRDGLAALA-YHAGLSDSAR-DEVQQKW----INQDG-CQ----VICATIAFGM
GIDKPDVRFVIHASLPKS-MEGYYQESGRAG-RDGEISHCLLF-YTYHDVTRLKRLI---
--------MMEKDG--NHHTR--------ETHFNNLYSM--VHYCENITE----------
CRRI---QLLAYFGE--NGFNPDFCKKHPDV--SCDNC--CK----TKDYKTR--DVTDD
VKSIVRFVQEHSS-----SQGMRNIKHVGP-SGRFTMNML-VD-IFLGSKSAKIQS----
------GIFGKGSAYSRHNAERLFKKLILDKILDEDLYINAN-DQAI--AYVMLGNKAQT
VLNGN----L-------------K---VDFMETENSSSVKKQK----------ALVAKVS
Q------------------------------------------------------RE--E
MVKKCLGELTEVCKSLGKVFGV-HYFNIFNTVTLKKLA--ESLSSDPEVLLQIDGVTEDK
LEKYGAEVISVLQKYSE----------------WTSPAEDSSP--GISLSSSRGP---GR
SAAEE-----LDEEIPV-------SSHYFASKTRNERKRK--KMPASQRSKRRKAAS-SG
SK--AKG---------GSATCRNI--SSKTKSSSIIGSSSASHTC-----------QATS
---------------------GANSK----------------------------------
------------------------------------------------------------
------------------------------------------------------------
------------------------------------------------------------
------------------------------------------------------------
------------------------------------------------------------
------------------------------------------------------------
----------------------------------------------------LGIMAPP-
--------------------------------------------KP--------------
---------INRPFLKPSYAFS-----------------
```
