# Supplementary material for: Investigating the pathogenic SNPs in BLM helicase and their biological consequences by computational approach
Source: Sci Rep. 2020 Jul 23;10:12377. doi: 10.1038/s41598-020-69033-8 (PMC7378827; doi:10.1038/s41598-020-69033-8)
Supplement: Supplementary file 2 — Supplementary Figures [file 41598_2020_69033_MOESM2_ESM.pdf]

Investigating the pathogenic SNPs in BLM helicase and their biological consequences by computational approach

Contributors of the manuscript in terms of data generation, manuscript drafting, editing

Faisal A. Alzahrani[#1,2], Firoz Ahmed[#*3,4], Monika Sharma[#5], Mohd Rehan[6,7], Maryam Mahfuz[8], Mohammed N. Baeshen[9], Yousef Hawsawi[10,] Ahmed Almatrafi[11], Suliman Abdallah Alsagaby[12], Mohammad Azhar Kamal[3,4], Mohiuddin Khan Warsi[3,4], Hani Choudhry[13], Mohammad Sarwar Jamal[*6,7,14]

# Equal Contribution
**** Corresponding Author***

1. Department of Biochemistry, Faculty of Science, stem cells Unit, King Fahd Medical Research Center, King Abdulaziz University, Jeddah 21589, Saudi Arabia.
2. Aston Medical Research Institute, Aston Medical School, Aston University, Birmingham B4 7ET, UK.
3. Department of Biochemistry, College of Science, University of Jeddah, PO Box 80327, Jeddah, Saudi Arabia.
4. University of Jeddah Centre for Scientific and Medical Research (UJ-CSMR), University of Jeddah, Jeddah, Saudi Arabia.
5. Department of Chemical Sciences, Indian Institute of Science Education and Research (IISER), Mohali, India
6. King Fahd Medical Research Center, King Abdulaziz University, Jeddah, Saudi Arabia.
7. Department of Medical Laboratory Technology, Faculty of Applied Medical Sciences, King Abdulaziz University, Jeddah, Saudi Arabia
8. Department of Computer Science, Jamia Millia Islamia, New Delhi, Delhi, India
9. Department of Biology, College of Science, University of Jeddah, PO Box 80327, Jeddah, Saudi Arabia.
10. Department of Genetics, Research Center, King Faisal Specialist Hospital, and Research Center, MBC-03, PO Box 3354, Riyadh, 11211, Kingdom of Saudi Arabia.
11. Department of Biology, Faculty of Science, University of Taibah, Medinah, Saudi Arabia
12. Department of Medical Laboratories, Central Biosciences Research Laboratories, College of Science in Al Zulfi, Majmaah University, Kingdom of Saudi Arabia
13. Department of Biochemistry, Cancer Metabolism and Epigenetic Unit, Faculty of Science; Cancer and Mutagenesis Unit, King Fahd Center for Medical Research; King Abdulaziz University, Jeddah, Saudi Arabia.
14. Integrative Biosciences Center, Wayne State University, Detroit, MI 48202, USA

@ Corresponding Author:
Dr. Mohammad Sarwar Jamal (Email: sarwar4u@gmail.com & hd2149@wayne.edu)
Dr. Firoz Ahmed (Email: fahmed1@uj.edu.sa)

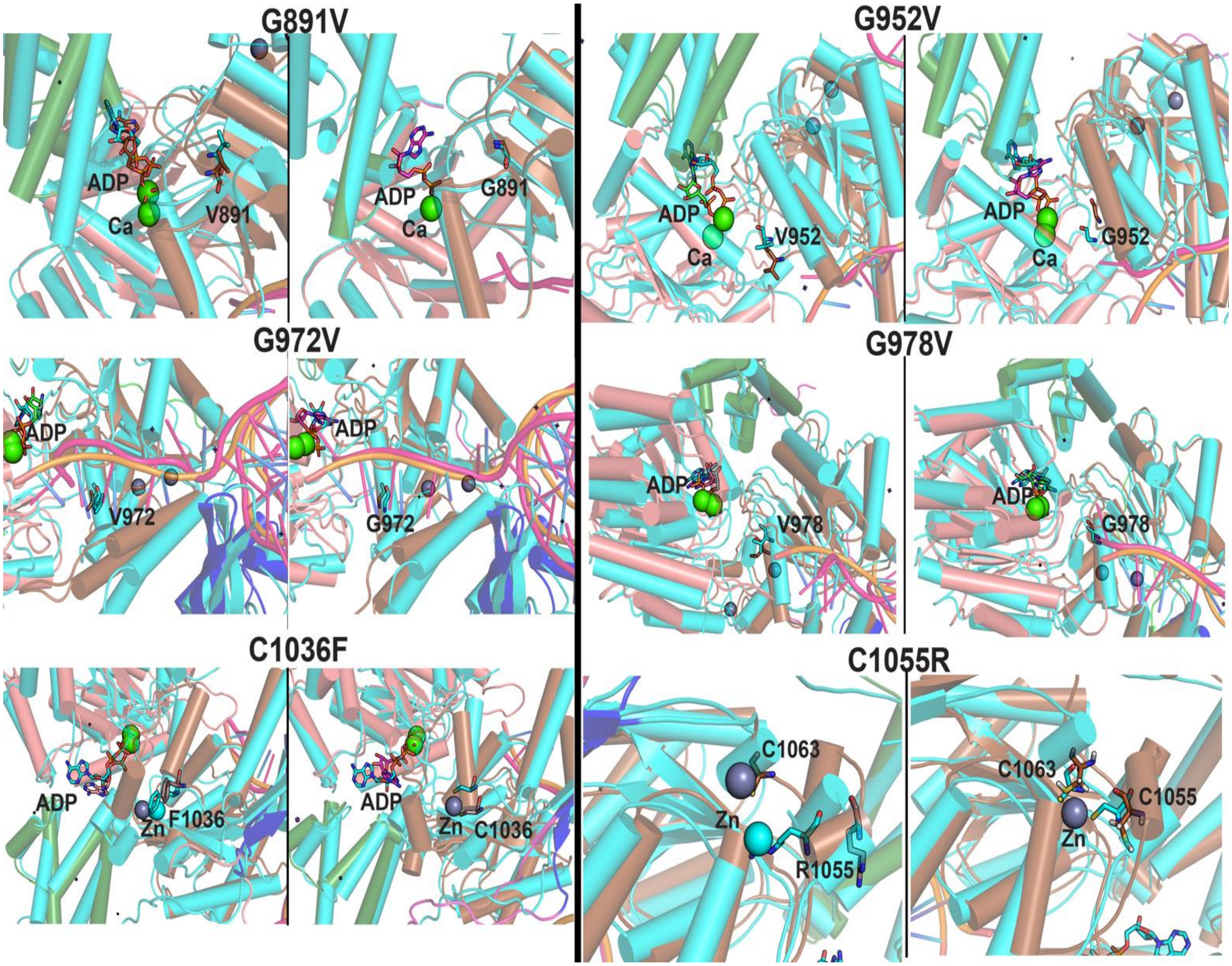

Figure S1: Comparative analysis of initial structure and final structures obtained after 20ns sampling for mutants with higher rmsd values with respect to native state. For each mutant analysis, two snapshots are provided; one on left is for mutant, and one on right is for native structure. The final structures obtained at the end of sampling for mutant or native structure are shown in cartoon colored according to Figure X, and the initial structure in both cases is shown in cyan colored cartoon.

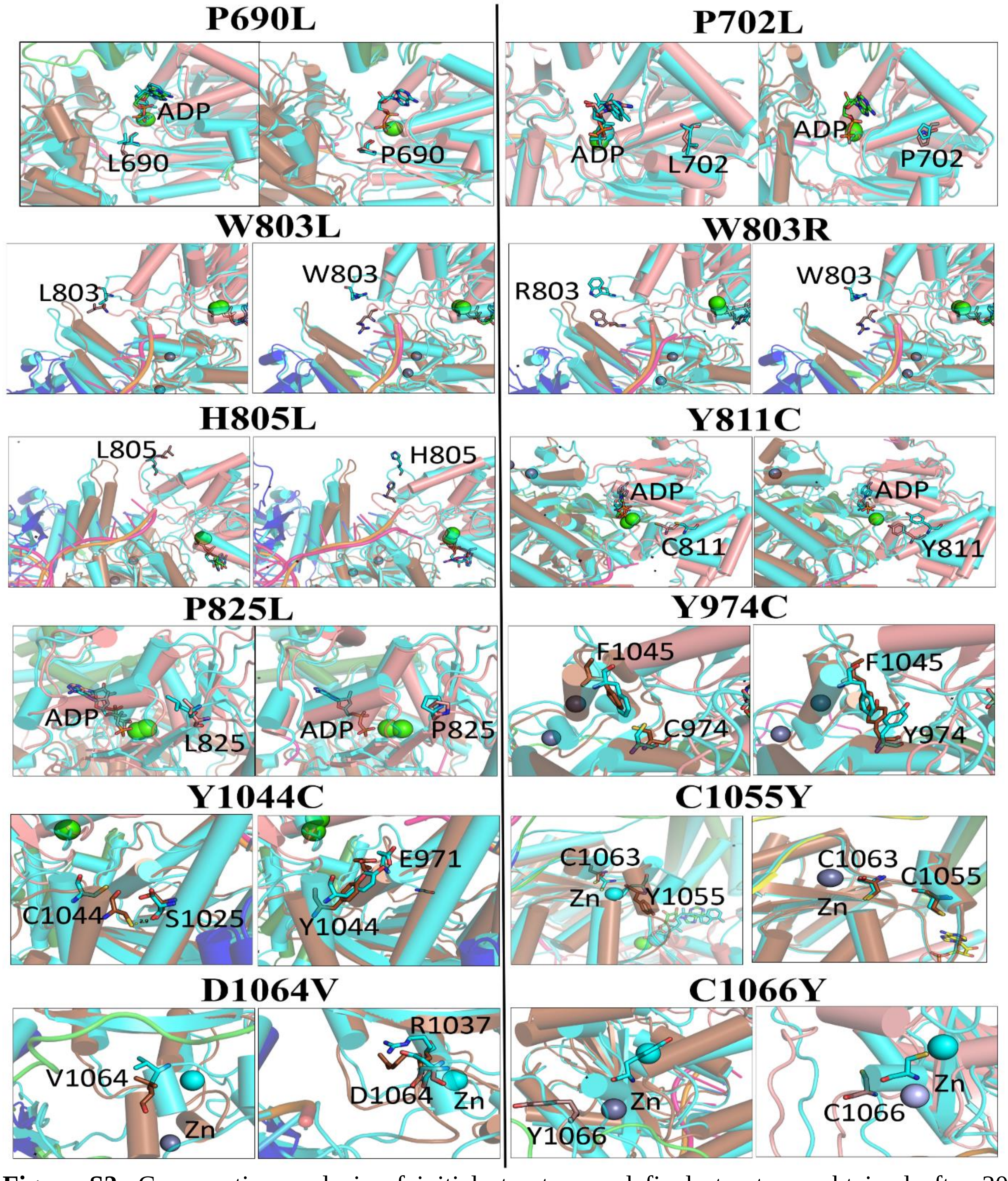

Figure S2: Comparative analysis of initial structure and final structures obtained after 20ns sampling for mutants with similar rmsd values with respect to native state. For each mutant analysis, two snapshots are provided; one on left is for mutant, and one on right is for native structure. The final structures obtained at the end of sampling for mutant or native structure are shown in cartoon colored according to Figure X, and the initial structure in both cases is shown in cyan colored cartoon.

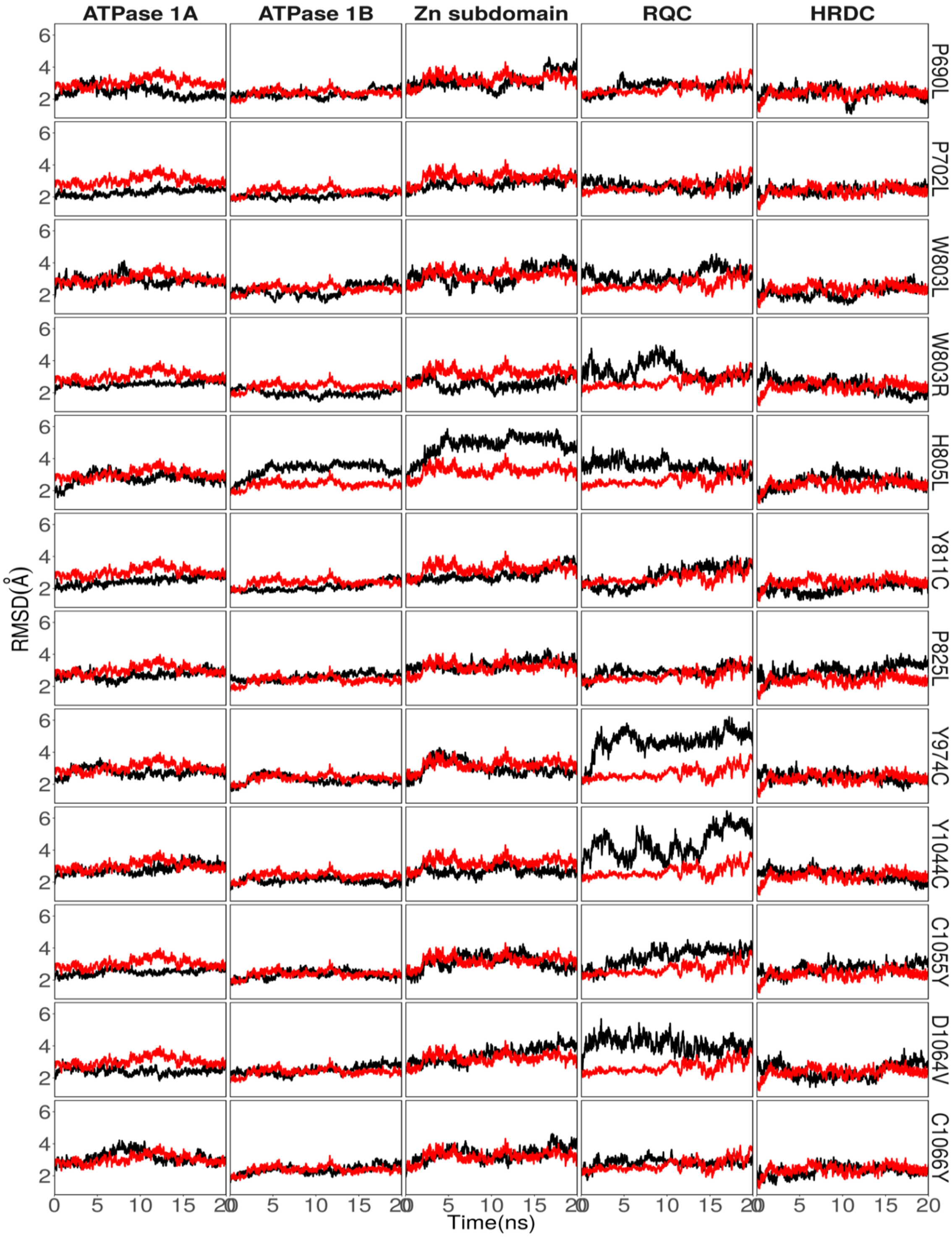

Figure S3: Domain wise rmsd plots for rest of the mutants. Black line corresponds for mutants and red line corresponds for WT/native structure.

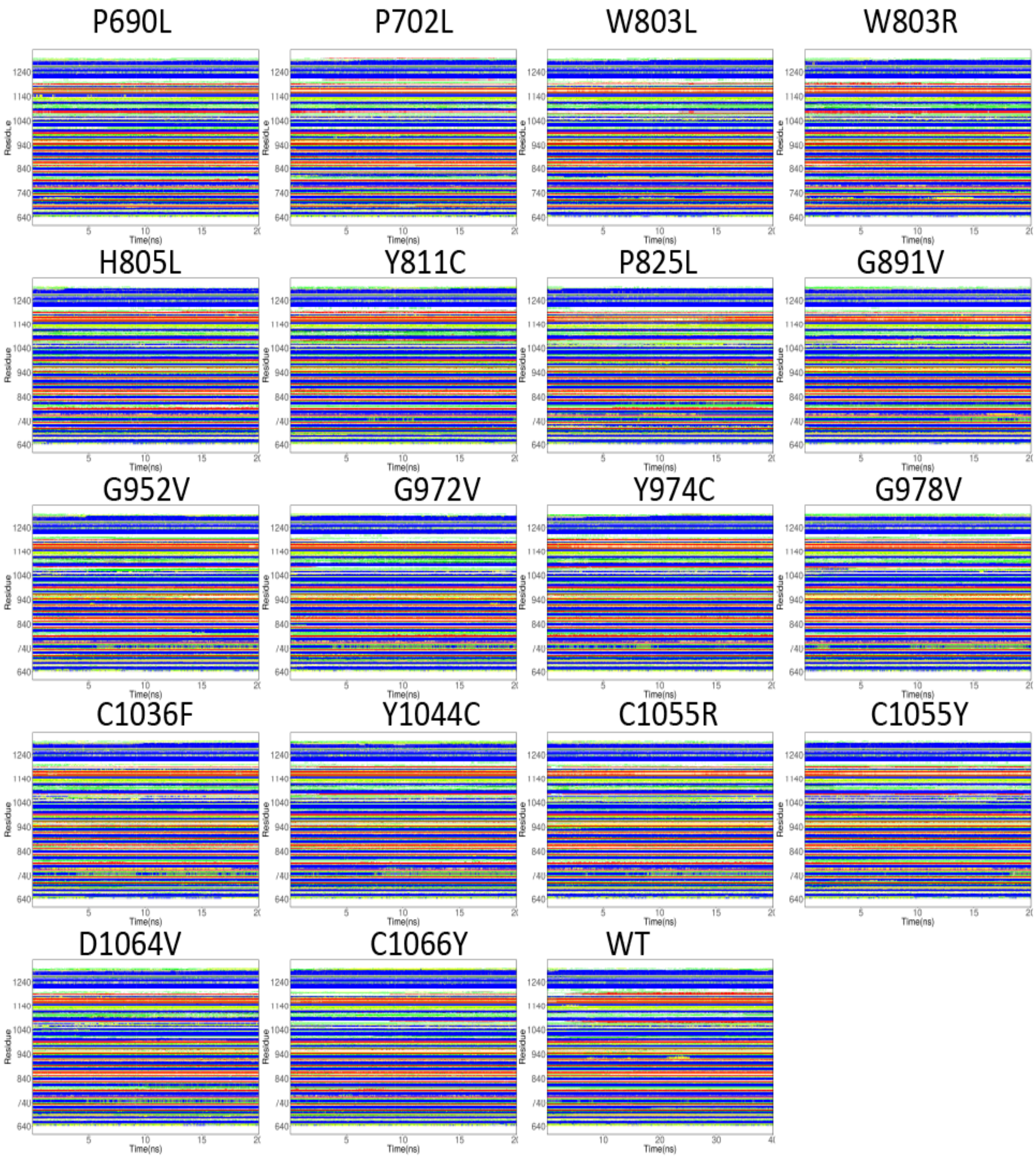

Figure S4: Variation in secondary structure for mutants and WT with respect to time as observed by DSSP algorithm. α-Helices are blue, beta sheets are red, beta bridges are black, bends are green, turns are yellow, 3_{10} helices are grey; and coils are white.
